# New molecular insights on the response of the green alga *Tetraselmis suecica* to nitrogen starvation

**DOI:** 10.1038/s41598-019-39860-5

**Published:** 2019-03-04

**Authors:** Chiara Lauritano, Daniele De Luca, Mariano Amoroso, Salvatore Benfatto, Simone Maestri, Claudia Racioppi, Francesco Esposito, Adrianna Ianora

**Affiliations:** 1 Marine Biotechnology Department, Stazione Zoologica Anton Dohrn, Villa Comunale, 80121 Napoli, Italy; 2 Integrative Marine Ecology Department, Stazione Zoologica Anton Dohrn, Villa Comunale, 80121 Napoli, Italy; 3 Present Address: Università degli Studi di Verona, Ca’ Vignal 1, Strada Le Grazie 15, 37134 Verona, Italy; 4 Center for Developmental Genetics, Department of Biology, College of Arts and Science, New York University, New York, USA

## Abstract

Microalgae are currently considered one of the most promising resources for biofuel production, aquaculture feedstock and new pharmaceuticals. Among them, green algae of the genus *Tetraselmis* are extensively studied for their lipid accumulation in nutrient-starvation conditions. In this paper, we present the full-transcriptome of *Tetraselmis suecica* and differential expression analysis between nitrogen-starved and -repleted conditions (at stationary phase) focusing not only on lipid metabolism but giving new insights on nutrient starvation responses. Transcripts involved in signal transduction pathways, stress and antioxidant responses and solute transport were strongly up-regulated when *T*. *suecica* was cultured under nitrogen starvation. On the contrary, transcripts involved in amino acid synthesis, degradation of sugars, secondary metabolite synthesis, as well as photosynthetic activity were down-regulated under the same conditions. Among differentially expressed transcripts, a polyketide synthase and three lipoxygenases (involved in the synthesis of secondary metabolites with antipredator, anticancer and anti-infective activities) were identified, suggesting the potential synthesis of bioactive compounds by this microalga. In addition, the transcript for a putative nitrilase, enzyme used in nitrile bioremediation, is here reported for the first time for *T. suecica*. These findings give new insights on *T. suecica* responses to nutrient starvation and on possible biotechnological applications for green algae.

## Introduction

Nitrogen is the second most important nutrient, after carbon, in phytoplankton and is generally considered the major element limiting phytoplankton growth in the marine environment^1^. Nitrogen coupling with carbon is essential for the biosynthesis of nucleic acids, proteins and chlorophylls, sharing of energy and organic compounds via glycolysis, the tricarboxylic acid (TCA) cycle, the mitochondrial electron transport chain and photosynthesis^2^. Various studies have shown that several microalgae such as *Desmodesmus* sp., *Chlorella* sp. and *Chlamydomonas reinhardtii* (Chlorophyceae), *Nannochloropsis oculata* (Eustigmatophyceae) and *Porphyridium cruentum* (Rhodophyceae) increased lipid accumulation when cultured in nitrogen starvation (N starvation) and, for this reason, they have been proposed as promising feedstock for biodiesel production^3–6^. N starvation induced an increase in glycolytic and TCA cycle enzymes in the marine diatom *Thalassiosira pseudonana* (Mediophyceae^7^) and *de novo* biosynthesis of triacylglycerols, decrease of chloroplast galactolipids and reorganization of the photosynthetic apparatus in the flagellate *Nannochloropsis gaditana*^8^. However, cellular responses triggered by N starvation are not completely clarified.

Among microalgae, green algae, with more than 7000 species growing in a variety of habitats^9^, have been frequently studied for energy purposes^10^, but also as sources of bioactive extracts/compounds^11,12^. *Tetraselmis* spp. (green algae) are widely harvested as feed for molluscs, shrimp larvae and rotifers^13^, for their antimicrobial activity^14^, as sources of vitamins for animal and human consumption^15^ and for biodiesel production^16^. *T. suecica* clone CCMP906 raw extracts did not show any antimicrobial, antioxidant, anticancer and anti-diabetes activities^17^, but the purified carotenoid extract had a strong antioxidant and repairing activity in the human lung cancer cell line (A549) and on reconstructed human epidermal tissue cells (EpiDerm^TM^ ^12^). These data suggest that this species has cosmeceutical activity and potential interesting biotechnological applications.

In this paper, we present for the first time the full-transcriptome of the green alga *Tetraselmis suecica* (CCMP906) and differential expression analysis between N-starved and –repleted (control) conditions focusing not only on lipid metabolism but giving new insights on N starvation responses and possible biotechnological applications for this species.

Even in the absence of a fully sequenced and annotated genome, transcriptomic analysis by RNA-sequencing can provide a powerful tool to improve our understanding of physiological networks that allow microalgae to respond to various environmental cues^18^. Regarding *Tetraselmis*, transcriptome sequencing has been done for *Tetraselmis* sp. GSL018 (MMETSP0419), *T. chuii* PLY429 (MMETSP0491), *T. astigmatica* CCMP880 (MMETSP0804) and *T. striata* LANL1001 (MMETSP0817, MMETSP0818, MMETSP0819, MMETSP0820). In addition, Adarme-Vega *et al*. studied some specific genes involved in lipid metabolism in the clone *Tetraselmis* sp. M8^19^ and, recently, Lim *et al*. have sequenced the transcriptome of *Tetraselmis* sp. M8 clone in nitrogen depletion in order to study lipid-related pathways that lead to triacylglyceride accumulation in oleaginous microalgae^20^. Our study focuses on N starvation-induced metabolic changes and new insights on *Tetraselmis* responses to low concentrations of this nutrient.

## Materials and Methods

### Cell culturing and harvesting, RNA extraction and cDNA synthesis

*Tetraselmis suecica* (CCMP906) was cultured in Guillard’s f/2 medium^21^ without silicic acid. Experimental culturing for both control and nitrogen starvation conditions was performed in 2 litre polycarbonate bottles (each experiment was performed in triplicate) constantly bubbled with air filtered through 0.2 µm membrane filters. For the N starvation experiment the medium was prepared with low concentrations of nitrogen (30 mM of NO_3_^−^; N starvation condition). Cultures were kept in a climate chamber at 19 °C on a 12:12 h light:dark cycle at 100 µmol photons m^−2^ s^−1^. Initial cell concentrations were about 5000 cells/mL for each experiment and net growth was monitored^22^. Aliquots of 50 mL were sampled during the stationary phase (day 7) and centrifuged for 15 minutes at 4 °C at 1900 g (Eppendorf, 5810 R). Cell concentration was ~2 × 10^6^ cells ml^−1^ for the control condition and ~2 × 10^5^ cells ml^−1^ for N-starved cells. For RNA extractions, both RNA sequencing (RNAseq) and reverse transcription-quantitative PCR (RT-qPCR) pellets (triplicates for each condition and for each technique) were re-suspended in 500 µL of TRIZOL© (Invitrogen, Carlsbad, CA), incubated for 2–3 min at 60 °C until completely dissolved and kept at −80 °C^23^.

RNA was extracted as in Lauritano *et al*.^18^ using TRIZOL® manufacturer’s instructions. For RT-qPCR, 500 ng/replicate were retrotranscribed into cDNA with the iScriptTM cDNA Synthesis Kit (BIORAD, Hercules, CA) following the manufacturer’s instructions.

### Library preparation, sequencing and assembly

RNA-Seq libraries were prepared from 2.5 µg of total RNA using the Illumina TruSeq® Stranded mRNA kit (Illumina Inc., San Diego, CA, USA) according to the manufacturer’s instructions. Paired-end sequencing (2 × 100 bp) was performed with the HiSeq1000 Illumina platform. Adaptor trimming of the reads was performed using CutAdapt (ver. 1.6), followed by duplicates removal and then abundance normalization with khmer software (ver. 1.1). *De novo* assembly was performed with the Oases/Velvet assembler (v. 0.2.08) with multiple k-mers (from k-mer 25 to k-mer 85 with steps of 10^24,25^). Clustering and redundancy removal was performed by using EvidentialGene (ver. 2013-07-27). To evaluate coverage depth on the obtained transcriptome, the filtered RNA-seq reads were mapped against it using BWA mem (ver. 0.7.5a-r405) and data were filtered to eliminate transcripts with less than 100 alignments.

### Functional annotation and differential expression analysis

Functional annotation of non-redundant contigs was performed using Blast2GO software (Version 4.1.9) with NCBI-NR database and default parameters^26^. Expression abundances were quantified using RSEM (version 1.1.21) with default settings^27^. Differentially expressed genes (FDR ≤ 0.001; |Log2(FC)| ≥ 2) were identified by using the R package DESeq^28^. Raw read counts were transformed to FPKM (fragments per kilobase of exon per million fragments mapped). Analysis with BUSCO software (v3.0.0) was performed using the eukaryote dataset (odb9). In addition, transcript functional categories were also deeply investigated by using the Kyoto Encyclopedia of Genes and Genomes (KEGG) annotation^29^. Raw reads and assembled transcripts have been deposited in GenBank (GEO database^30^; series entry GSE109461).

### Reverse transcription-quantitative polymerase chain reaction (RT-qPCR)

Primers for putative reference genes (RGs) and genes of interest (GOI) were designed using the software Primer3 v. 0.4.0 (http://frodo.wi.mit.edu/primer3/) and optimized as in Lauritano *et al*.^31^. Supplementary Table S1 lists selected RGs and GOI, their functions, primers’ sequences and efficiencies. To normalize expression levels of the selected GOI, a panel of putative RGs (i.e. actin, alpha and beta tubulins, glyceraldehyde 3-phosphate dehydrogenase, histone 1 and 4) was first screened in the 2 experimental conditions: control and N starvation conditions. The best RGs (i.e. histone 1, actin and β tubulin, see Supplementary Table S2) were identified by using the software BestKeeper^32^, geNorm^33^ and NormFinder^34^. Primer reaction efficiency (E) and correlation factor (R^2^) were calculated using the equation E = 10^−1/slope^. GOI were selected between the most up- or down-regulated DEGs with functional annotation: Calcium/calmodulin-dependent protein kinase type 1 (CAMK), Protein phosphatase 2c family protein (PP2C), Squamosa promoter binding protein (SBP), Ammonium transporter (AMT), ATP-binding cassette protein transporter (ABC), Glutathione-S-transferase (GST), Catalase (CAT), Heat shock protein 20 (HSP20), Phosphoenolpyruvate carboxylase kinase (PPCK), Lipoxygenase (LPX), Polyketide synthase (PKS), Nitrilase (NIT1), 3,5-cyclic nucleotide phosphodiesterase (PDE) and Elmo-domain-containing protein 3 (ELMOD3). RT-qPCR was performed as in Lauritano *et al*.^18^ in a Viia7 real-time PCR system (Applied Biosystem) and using the Relative Expression Software Tool^35^ for expression level analyses. Control condition was represented by microalgae cultured in normal repleted-medium. Statistical analysis was performed using GraphPad Prim statistic software, V4.00 (GraphPad Software). Normality of data was tested by using the Anderson-Darling test^36^ with the PAST software (v.3.15^37^).

### Protein structure prediction of selected transcripts

We selected some transcripts (lipoxygenases, nitrilase and polyketide synthase) with known potential biotechnological applications to further investigate their structure at protein level. Their nucleotide sequence was first translated into the corresponding amino acid one from the first methionine to the first stop codon using the Translate tool of ExPASy^38^ (https://web.expasy.org/translate/); then, functional domains of the protein sequences were annotated using the webserver InterProScan (available at https://www.ebi.ac.uk/interpro/search/sequence-search) and protein structures were predicted using the Phyre2 web server (http://www.sbg.bio.ic.ac.uk/~phyre2/html/page.cgi?id=index). For PKS, separate analyses were done for each domain identified by InterProScan (https://www.ebi.ac.uk/interpro/search/sequence-search).

### Phylogenetic analysis of nitrilase

In order to assess the evolutionary relationships among the nitrilase found in our transcriptome and the others from closely related organisms, we inferred a phylogenetic analysis retrieving homologous sequences from different databases as eggNOG v. 4.5 (available at http://eggnogdb.embl.de/#/app/home), the UniRef90 database of the BLAST tool available at UniProt server (http://www.uniprot.org/blast/) and the genome database for red algae (realDB, http://realdb.algaegenome.org/P.e.a.html#). The latter database was used to include homologous sequences of distantly related taxa that are at the basis of the evolutionary lineage that led to green algae and land plants. We also downloaded green algae transcriptomes from the Marine MicroEukaryote Transcriptome Sequencing Project (MMETSP) to increase the number of nitrilase sequences to be analysed. Finally, the sequences of land plants nitrilases by Howden *et al*.^39^ were added into our analysis to assess the evolutionary relationships with our sequence. All sequences were aligned in ClustalX2^40^ and then edited manually. A Maximum Likelihood (ML) tree was built using RAxML^41^ under the substitution model LG + G suggested by PartitionFinder v.1.1.1^42^ using the AICc criterion. Support to branches was inferred via bootstrap analysis using the autoMRE option of RAxML. The resulting tree was visualised and graphically edited in FigTree v1.4.3 (http://tree.bio.ed.ac.uk/software/figtree/).

## Results and Discussion

### Transcriptome sequencing, *de novo* assembly and functional annotation

RNA-sequencing (RNA-seq) from samples cultured in control culturing condition and in nitrogen starvation (N starvation) yielded 26,550,078 and 5,005,719 total raw or normalized fragments, respectively, per sample on average (Table 1). As no available reference genome of *T. suecica* was available, normalised RNA-seq reads have been assembled with *de novo* approach producing 621,424 putative transcripts. In order to evaluate the assembled transcriptome, general statistics have been computed (Table 2). Clustering and redundancy removal resulted in a transcriptome of 31,352 main transcripts with an average length of 962.6 bp (Table 1). Of these, 24,399 transcripts were supported by sufficient RNA-seq reads (>100) and 15,027 were further associated to a known protein, based on the NCBI database. Analysis with the Benchmarking Universal Single-Copy Orthologs (BUSCO) software identified 94.4% of 303 BUSCOs reference transcripts (81.5% completed and 12.9% fragmented, see Supplementary Table S3) demonstrating that the large majority of the transcriptome has been reconstructed with mostly full length transcripts.

**Table 1:** Total, filtered and normalized fragments obtained from the RNA-sequencing analysis performed using 100 nt paired-end reads. Each experimental condition was analysed in triplicate.

	Total fragments (100 × 2)	Filtered fragments	Normalized fragments
Control 1	27,029,365	22,666,543	5,696,910
Control 2	24,675,025	18,897,676	5,319,434
Control 3	30,219,523	25,188,451	5,987,306
Nitrogen starvation 1	25,175,982	18,601,502	4,778,109
Nitrogen starvation 2	26,652,748	20,280,597	4,273,429
Nitrogen starvation 3	25,547,826	18,029,006	3,979,124

**Table 2:** *De novo* assembly statistics.

Parameter	
Assembly length	30,123,488 bp
Minimum length of contigs	165 bp
Maximum length of contigs	13,961 bp
Average length of contigs	962.6 bp
N50	1,215 bp
N90	486 bp

Functional annotation using Gene Ontology (GO) assigned molecular function, biological process and cellular localization to 46% of the putative transcripts (see Supplementary Fig. S1). The Kyoto Encyclopedia of Genes and Genomes (KEGG) annotation (see Supplementary Table S4), identified the presence of 134 metabolic pathways. Of these, the biosynthesis of the antibiotic pathway (Pathway ID map01130), was the one with the highest number of enzymes associated to it (112 enzymes). Other highly represented pathways were purine metabolism (Pathway ID map00230), pyruvate metabolism (Pathway ID map00620), amino sugar and nucleotide sugar metabolism (Pathway ID map00520), glycolysis/gluconeogenesis (Pathway ID map00010), starch and sucrose metabolism (Pathway ID map00500) and alanine, aspartate and glutamate metabolism (Pathway ID map00250).

### Differential expression analysis

**Figure 1:**
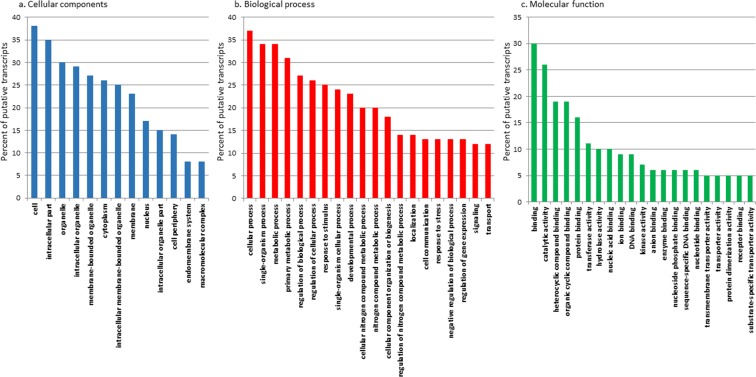
Histograms of GO classifications showing sequence distribution of the differentially expressed genes within cellular component (**a**), biological process (**b**) and molecular function (**c**). The y-axis indicates the percentage of sequences for each category.

**Figure 2:**
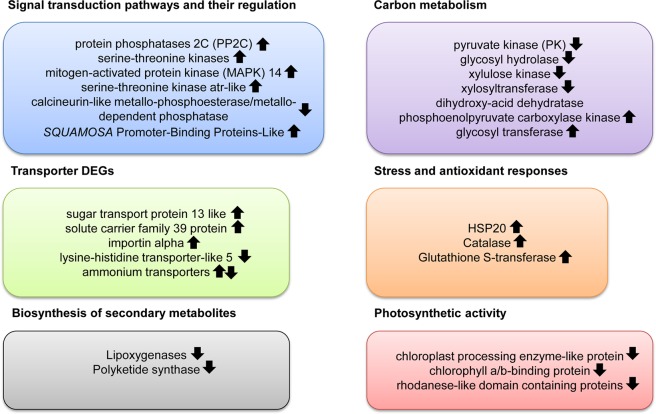
Summary of the main results. Up-regulated transcripts were mainly involved in signal transduction pathways, stress and antioxidant responses and solute transport while transcripts involved in amino acid synthesis, degradation of sugars, secondary metabolite synthesis and photosynthetic activity were down-regulated in nitrogen starvation condition.

Differential expression analysis identified 319 genes with significant expression variations (|LogFC| > 2; P value adjusted ≤0.01) in N starvation condition relative to control (i.e. *T. suecica* cultured in complete K medium). GO annotation was used to identify major categories of genes differentially expressed between the two experimental conditions and percentage of sequences for each GO term within cellular component, biological process and molecular function are reported in Fig. 1. Among the 319 differential expressed genes (DEGs; of which 189 were up-regulated and 130 down-regulated), 166 transcripts had no NCBI NR assignment (of which 107 were up-regulated and 59 down-regulated), while the remaining 153 included 82 up-regulated and 71 down-regulated genes. The full list of DEGs, log2 x-fold change, adjusted P value (padj), and their GO annotation are reported in the Supplementary Table S5. Among the DEGs, the ones showing the highest expression in N starvation conditions were an extracellular ligand-binding receptor (padj = 4,32E-64), the abc transporter substrate-binding protein (padj = 4,73E-181), the elmo domain-containing protein 3-like (padj = 3,72E-15) and the 3,5-cyclic nucleotide phosphodiesterase (padj = 3,30E-05). Conversely, N starvation induced a strong down-regulation of polyketide synthase (PKS; padj = 8,17E-20). Figure 2 summarizes the main results while details are reported in the following paragraphs. Up-regulated transcripts were mainly involved in signal transduction pathways, stress and antioxidant responses and solute transport while transcripts involved in amino acid synthesis, degradation of sugars, secondary metabolite synthesis and photosynthetic activity were down-regulated when cultured in N starvation.

### DEGs involved in signal transduction pathways and their regulation

In N starvation condition, *T. suecica* activated several signal transduction pathways involving protein kinases and phosphatases. Some of these are protein phosphatases 2 C (PP2C), involved in mitogen-activated protein kinase (MAPK) signalling^43^, and serine-threonine kinases, playing a central role in cell-cycle regulation by transmitting DNA damage signals to downstream effectors of cell-cycle progression^44^. Eukaryotic MAPK cascades transduce environmental and developmental cues into intracellular responses^45,46^. To date, the activation of MAPKs in response to N starvation has been observed in the yeast *Saccharomyces cerevisiae*^47,48^ and in the ascomycete fungus *Fusarium proliferatum*^49^, but no information exists in microalgae. In the present study, we found a 3 fold up-regulation of MAPK 14 during N starvation. MAPK signalling is regulated by the action of phosphatases^50^ and we observed a 5.4 fold up-regulation of PP2C^51,52^. Interactions between PP2C and MAPK have been observed in *S. cerevisiae* in response to osmotic stress^53^ and in *A. thaliana* during stress responses^54,55^. Since we found a 5.4 up-regulation of PP2C, together with a significant expression of MAPK in N starvation, we suggest a possible role of this signalling pathway in the response to N starvation in *T. suecica*.

We also found a transcript coding for a serine-threonine kinase atr-like which was 4.5 fold up-regulated and a calcineurin-like metallo-phosphoesterase/metallo-dependent phosphatase that was 2.6 fold down-regulated. The serine-threonine kinase atr is a kinase which can be involved in stress responses (https://www.uniprot.org/uniprot/Q13535). The function of calcineurins are less known compared to other members of this family in plants, while there is no information regarding microalgae. Our study suggests a possible role in microalgal response to N starvation.

Three transcripts coding for putative *SQUAMOSA* Promoter-Binding Proteins-Like (SPL) were significantly up-regulated as well (4.9, 3.9, and 2.2 fold, respectively). These are transcription factors that are involved in the regulation of other transcription factors and metabolic processes^56^. A recent phylogenetic analysis revealed nine major SPL gene lineages in higher plants, each of which is described in terms of function and diversification^57^, but an extensive knowledge in their closest relatives, the green algae, is still missing. Our study indicates a possible role during nitrogen starvation and calls for further investigations.

### Transporter DEGs

A significant differential expression of several genes involved in the transport of different metabolites was observed in our experiment. In particular, the sugar transport protein 13 like (for sugar transport), the solute carrier family 39 protein (generic transporter of solutes) and importin alpha (involved in the import of proteins into the nucleus) were 2.4, 4.1 and 3.9 fold up-regulated, respectively. On the contrary, lysine-histidine transporter-like 5 was significantly 4.5 fold down-regulated. The decrease of transcripts involved in the carriage of amino acids is compatible with the same trend observed in their production as a consequence of N starvation. In contrast, the increase of sugar and solute carriers is probably due to the altered metabolic state of the cell and consequently need of reallocating such compounds.

Two transcripts referring to ammonium transporters were also found in our transcriptome, whose encoded amino acid sequences shared a 40% similarity. One was 2.7 fold up-regulated and the other one 4.4 fold down-regulated. Ammonium transporter genes (AMTs) have been found in several phytoplankton species including diatoms^58^, green algae^59,60^, haptophytes^61^ and prasinophytes^62^. A study conducted in the marine diatom *Cylindrotheca fusiformis* also revealed the occurrence of two types of AMT that were differentially expressed under N starvation conditions^63^. Both studies suggest that different AMT isoforms of the same species may not be activated under the same experimental conditions (as also observed in higher plants^64^).

### DEGs involved in stress and antioxidant responses

Microalgae are constantly exposed to both physical and chemical stressors which they react to by activating a series of defense mechanisms. The most common defense strategies include the activation of heat shock proteins (HSPs) and antioxidant enzymes^65,66^. HSPs are molecular chaperones that can be involved in protein folding and unfolding, and degradation of mis-folded or aggregated proteins^65^. The antioxidant enzymes detoxify reactive or toxic intermediates (e.g. reactive oxygen species) which can be damaging to DNA, RNA and proteins^66^. In this study, N starvation induced the activation of HSP20 (3.7 fold up-regulation) and the antioxidant enzymes catalase (CAT; 2.5 fold up-regulation) and glutathione S-transferase (GST; 2.07 fold up-regulation). HSP20 was highly up-regulated also in a recent paper on the diatom *P. tricornutum*^67^ and the plant *A. thaliana* cultured in N starvation^68^. CAT enzymatic activity increased in the chlorophytes *Chlorella sorokiniana* and *Coccomyxa* sp.^69,70^ and GST also increased in *C. reinhardtii* exposed to N depletion^71^. Both CAT and GST were not differentially regulated in other green algae exposed to N starvation/depletion. These data highlight a connection between nutrient deprivation, oxidative stress and detoxification of free radicals; however, the responses were slightly different depending on the studied species.

### DEGs involved in carbon metabolism

Several transcripts involved in glycolytic pathways, like pyruvate kinase (PK), glycosyl hydrolase, xylulose kinase and xylosyltransferase, were down-regulated. PK plays an important role in the initiation of *de novo* amino acid synthesis providing carbons to the tricarboxylic acid (TCA) cycle^2^. The down-regulation of this transcript is in agreement with the decrease in the number of transcripts of enzymes involved in the biosynthesis of several amino acids such as dihydroxy-acid dehydratase for valine-leucine-isoleucine biosynthesis (2.3 fold down-regulation). On the contrary, in the green alga *Chlamydomonas reinhardtii* no drastic changes were observed in pyruvate kinase expression after N starvation^72^. The phosphoenolpyruvate carboxylase kinase (PPCK), responsible for phosphoenolpyruvate carboxylase phosphorylation and activation, was strongly up-regulated (5.225 fold up-regulated). Phosphoenolpyruvate carboxylase (PPC) catalyses the fixation of CO_2_ to yield oxaloacetate, playing several key roles in the central metabolism of plants (e.g. regulation of carbon fixation and TCA cycle^73^). Hence, the increased transcription of PPCK indicates a possible cellular signalling for the regulation of carbon fixation and TCA cycle upon nitrogen starvation.

Glycosyl hydrolases (also called glycosylases) are a family of enzymes mainly involved in the degradation of complex sugars such as cellulose, hemicellulose, and starch^74^. Together with glycosyl transferases, which are involved in the establishment of glycoside linkages, they form the major catalytic machinery of sugar bonds. We found a 2.2 fold down-regulation of a glycosyl hydrolase transcript and a 2.2 up-regulation of a glycosyl transferase, indicating that during N starvation there is a trend in the accumulation of carbon compounds rather than in their degradation or *de novo* biosynthesis. Other transcripts related to carbohydrate metabolism, such as xylulose kinase and xylosyltransferase, were 2 and 2.2 fold down-regulated, respectively. Xylulose kinase is a key enzyme for arabinose and xylose metabolism in the green alga *Chlorella protothecoides*^75^, xylose utilisation in *S. cerevisiae*^76^ and biosynthesis of plastidial isoprenoids in *A. thaliana*^77^. Furthermore, this enzyme is involved in the phosphorylation of xylulose to xylulose 5-phosphate, which plays an important role in the regulation of glucose metabolism and lipogenesis^75^. Xylosyltransferase is an enzyme involved in the biosynthesis of glycosaminoglycans, which are known to have anticoagulant and anti-inflammatory properties, as well as tissue repairing properties^78^. Hence, the presence of this enzyme also suggests the possible production of other tissue-repairing compounds.

Transcripts involved in lipid metabolism (such as 3-ketoacyl-ACP synthase and Glycerol kinase) were affected in *Tetraselmis* sp. M8 during N starvation (in early-stationary growth phase in nitrogen depletion)^20^ but were not differentially regulated in our study (in stationary growth phase in nitrogen starvation). Summarizing, our data suggest that lipid metabolism was not the most affected pathway by N starvation in *T. suecica*, whereas transcripts involved in sugar degradation were strongly down-regulated.

### Biosynthesis of secondary metabolites

**Figure 3:**
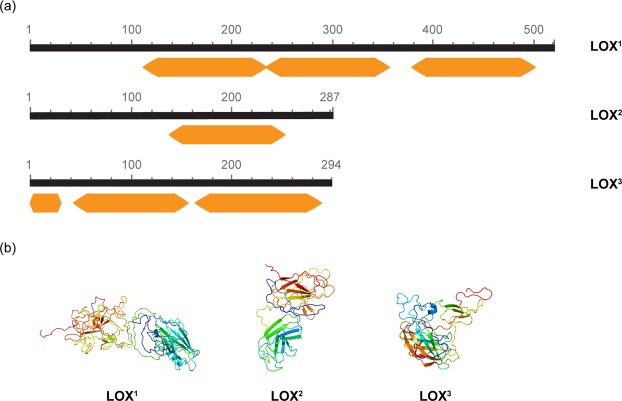
(**a**) Domain annotation of transcripts coding for lipoxygenase according to InterProScan. Query length is in black. Orange arrows refer to the PLAT/LH2 (Polycystin-1, Lipoxygenase, Alpha-Toxin/Lipoxygenase homology) domains. (**b**) Protein 3D structure predicted by Phyre2. Models are coloured by rainbow from N to C terminus. Helices in the secondary structure represent α-helices, arrows indicate β-strands and faint lines indicate coils. ^1,2^ and ^3^ stand for LOX transcript down-regulated by 2.46, 2.51 and 3.07, respectively, and reported in the DEG list.

Lipoxygenases (LOX), which are enzymes involved in fatty acid metabolism and biosynthesis of secondary metabolites with anti-proliferative activities^79^ (i.e. polyunsaturated aldehydes and other non-volatile oxylipins), were down-regulated in this study. Three transcripts including the PLAT (Polycystin-1, Lipoxygenase, Alpha-Toxin)/LH2 (Lipoxygenase homology) domains were found (2.458, 2.506 and 3.065 fold down-regulation, respectively). Domain assignment using InterProScan and structure prediction by Phyre2 confirmed that the three transcripts belong to the family of lipoxygenases (Fig. 3). For the first transcript (2.458 down-regulated; LOX^1^), three PLAT/LH2 domains were identified, at aa positions 112–227, 234–356 and 380–496, respectively. The structure was predicted at >90% accuracy based on the 47% of residues (aa positions 112–227). The second transcript (2.506 down-regulated; LOX^2^) contained only one PLAT/LH2 domain at aa positions 137–255 and its structure was predicted at >90% accuracy based on the 61% of residues. The third transcript (3.065 down-regulated; LOX^3^) contained three PLAT/LH2 domains (aa positions 1–35, 43–158 and 166–286). The 3D model was inferred at >90% accuracy using the 87% of residues (Fig. 3). However, our data suggest that this fatty acid metabolic pathway is affected by N starvation, and only future chemical analyses may confirm oxylipin production by *T. suecica*.

**Figure 4:**
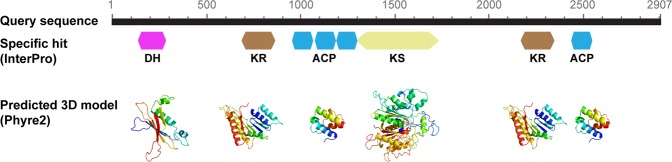
Detected domains and homology models for PKS transcript. Coloured blocks indicate the amino acid positions of the following domains: dehydratase (DH), keto-reductase (KR), phosphopantetheine-binding acyl carrier protein (ACP), β-ketoacyl synthase (KS), keto-reductase (KR) and phosphopantetheine-binding acyl carrier protein (ACP). Predicted 3D models are coloured by rainbow from N to C terminus. Helices in the secondary structure represent α-helices, arrows indicate β-strands and faint lines indicate coils. For the three consecutive domains of ACP the predicted protein model has been shown once.

In this study, a transcript for polyketide synthase I (PKS), involved in the synthesis of polyketides (compounds known to have antipredator, antimicrobial, anticancer and sometimes toxic activities^80^), was identified as well. Type I PKS are large multifunctional proteins, comprising several essential domains: acyltransferase (AT), β-ketosynthase (KS), acyl carrier protein (ACP), β-ketoacyl reductase (KR), enoyl reductase (ER), methyl transferases, thioesterases (TE) and dehydrogenase (DH) domains^81^. The InterProScan analysis of the transcript coding for PKS revealed the occurrence of the following domains: dehydratase (DH, aa positions 227–356), keto-reductase (KR, aa positions 692–875), phosphopantetheine-binding acyl carrier protein (ACP, aa positions 986–1060), β-ketoacyl synthase (KS, aa positions 1296–1735), keto-reductase (KR, aa positions 2179–2356) and phosphopantetheine-binding acyl carrier protein (ACP, aa positions 2467–2542). (Fig. 4). Protein structure prediction analysis confirmed the identification of all the domains (Fig. 4) with a coverage between 99.8 and 100%. In previous studies *T. suecica* showed antioxidant and protective activity on human cells^12^ without any cytotoxicity^17^ and this activity was associated to a pool of carotenoids. However, the presence of PKS may suggest the production of secondary metabolites that can be active as well. In N starvation, PKS was strongly down-regulated (5.77 fold down-regulation), as also found for the dinoflagellate *Amphidinium carterae*^18^ and for several fungi^82^.

Our data suggest that both lipoxygenase and PKS metabolism are affected by N starvation, thus reducing the possible secondary metabolite production they are involved in. This regulatory mechanism is known in fungi as *nitrogen metabolite repression*^82^ and this is the first study reporting this type of repression also in green algae.

### Photosynthetic activity related DEGs

Among the genes related to photosynthetic activity whose expression was significantly different between control and N-starved conditions, there were the chlorophyll a/b-binding protein, the chloroplast processing enzyme-like protein and two rhodanese-like domain containing proteins which were significantly down-regulated (3.3, 2.1, 2.4 and 4.0 fold respectively). Light-harvesting chlorophyll a/b-binding (LHCB) proteins are found in the antenna complex of the light-harvesting complex of photosystem II (PSII) and their expression at the gene level is considered to be an important mechanism to modulate chloroplast functions^83,84^. A significant decrease in chlorophyll a/b-binding protein was also observed in *A. thaliana* plants grown with low nitrogen concentrations^85^. Chloroplast processing enzymes are known to be involved in the cleavage of the precursor of the light-harvesting chlorophyll a/b-binding protein of photosystem II (LHCPII) and in the production of mature protein^86^. Rhodanases catalyse the transfer of a sulfane sulfur atom from thiosulfate to cyanide *in vitro*, and in vascular plants are involved in many processes including leaf senescence^87^, immune response^88^, and tethering of ferredoxin NADP+ oxidoreductase in electron transfer chains in photosynthesis^89,90^.

The down-regulation of these transcripts in N starvation can be interpreted as a way for algal cells to balance the alteration of C:N ratio due to N starvation. As a general pattern, photosynthesis has been demonstrated to be down-regulated in many organisms cultured under N starvation^7,72,91–93^. In some cases, major changes involving photosynthetic enzymes and apparatus were observed^94^; in others, our case included, only a decrease in photosynthetic enzymes (e.g. enzymes involved in maturation of photosystems, electron transfer chain) was found.

### DEG coding for putative nitrilase

Nitrilases (EC 3.5.5.1) catalyse the hydrolysis of nitriles to carboxylic acids and ammonia^95^. Nitrile converting enzymes have attracted substantial interest in several fields because nitriles (used as solvents, synthetic rubber, starting material for pharmaceuticals and herbicides^96^) are highly toxic, carcinogenic^96^ and cause of hazardous environmental pollution^97^. Likely, nitrilases are very important for nitrile biodegradation (enzymatic bioremediation). In addition, enzymes of the nitrilase superfamily have been shown to play different roles in the cell, such as vitamin and co-enzyme metabolism^98^, detoxification of small molecules^99,100^, synthesis of signalling mediators^101^ and post-translational modification of proteins^102^.

Nitrilase and nitrilase-like enzymes have been identified in several macro and microorganisms, but nitrilases were considered absent in algae^103^. We did not find publications reporting nitrilases in microalgae but, using our sequence as query, we found homologs in red and green algae as well as land plants, with a percentage of identity of about 60% and labelled as putative nitrilase, nitrilase-like protein 2 or carbon-nitrogen hydrolase (see Supplementary Table S6).

**Figure 5:**
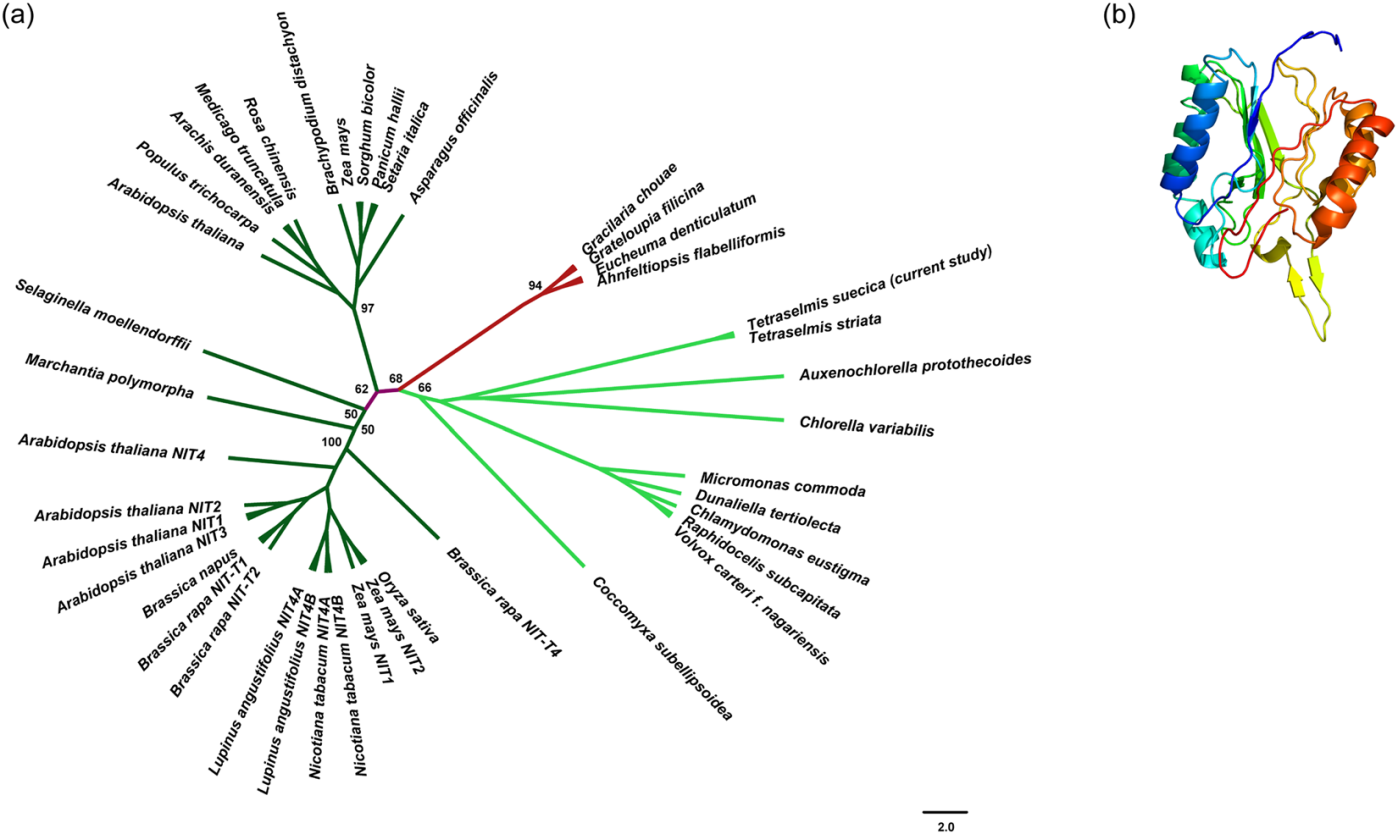
(**a**) Phylogenetic tree showing the evolutionary relationships of enzymes belonging to the nitrilase superfamily in the plant lineage. In red are highlighted red algae (Rhodophyta), in green the green algae (Chlorophyta) and in dark green the land plants (Spermatophyta). Bootstrap values were reported only for internal and basal nodes. (**b**) Predicted 3D structure of the enzyme using Phyre2. Colours are by rainbow from N to C terminus. Helices in the secondary structure represents α-helices, arrows indicate β-strands and faint lines indicate coils.

Our transcript was significantly down-regulated in N starvation conditions (3.48 fold down-regulation) suggesting that nutrient starvation affects its function and indicating a putative role in nitrogen metabolism. The cladogram in Fig. 5 showed that it clusters together with other putative nitrilases of green algae (in light green) and is not strictly related to the nitrilases characterized in land plants by Howden *et al*.^39^ (in dark green). However, further investigations at the biochemical and molecular levels are needed to unravel their function in the cell.

### Data validation by Reverse transcription-quantitative PCR

**Figure 6:**
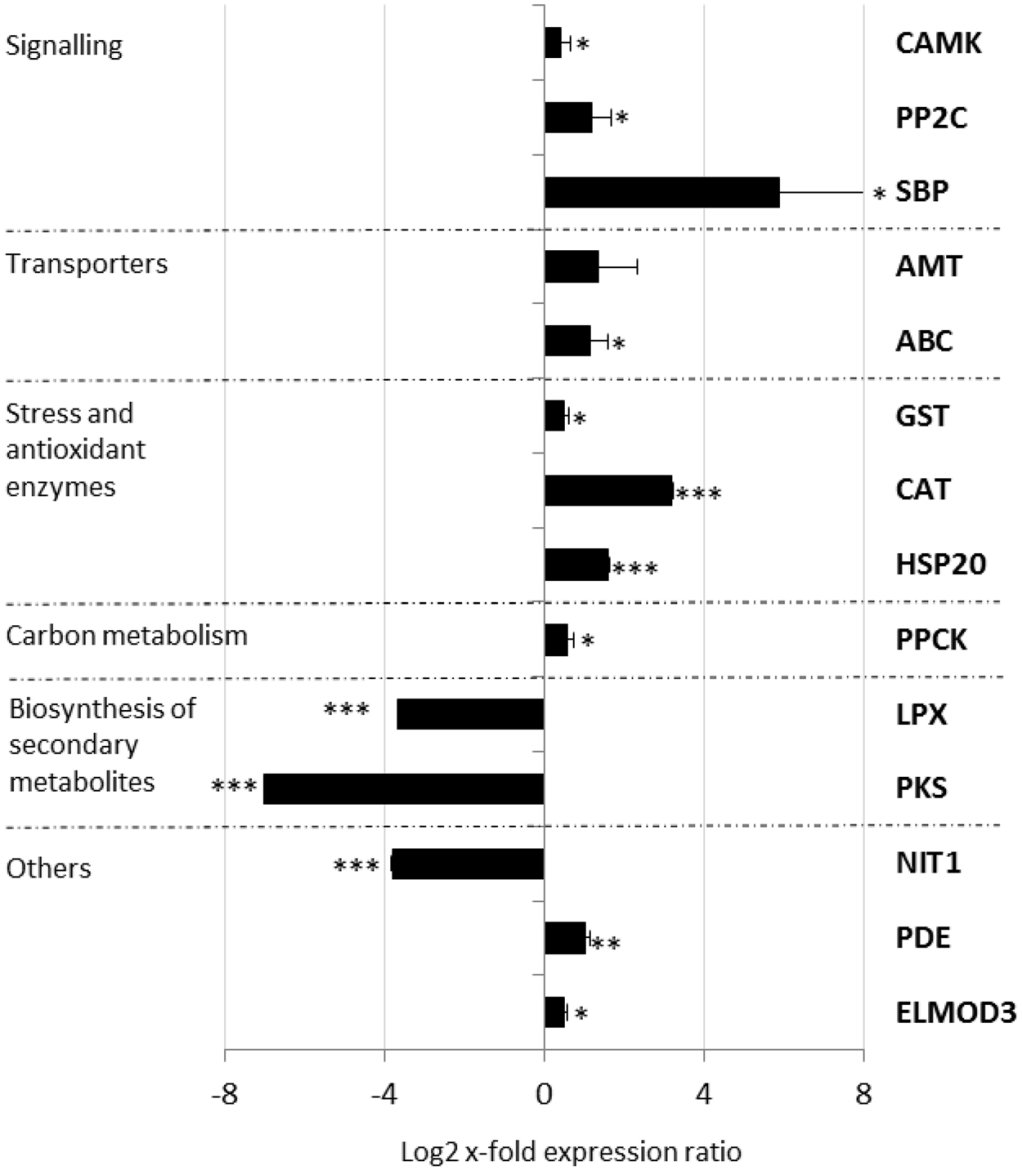
Expression levels of selected genes in *Tetraselmis suecica* cells cultured in nitrogen starvation compared to control conditions (i.e. culturing in complete medium; represented in the figure by the x-axis). Data are represented as log2 x-fold expression ratio ± SD (n = 3). Gene abbreviations are: Calcium/calmodulin-dependent protein kinase type 1 (CAMK), Protein phosphatase 2c family protein (PP2C), Squamosa promoter binding protein (SBP), Ammonium transporter (AMT), ATP-binding cassette protein transporter (ABC), Glutathione-S-transferase (GST), Catalase (CAT), Heat shock protein 20 (HSP20), Phosphoenolpyruvate carboxylase kinase (PPCK), Lipoxygenase (LPX), Polyketide synthase (PKS), Nitrilase (NIT1), 3,5-cyclic nucleotide phosphodiesterase (PDE) and Elmo-domain-containing protein 3 (ELMOD3).

Reverse transcription-quantitative PCR (RT-qPCR) of 14 selected transcripts, between the most up- and down-regulated DEGs with functional annotation (see Supplementary Table S1), showed a good correlation with RNAseq data (R = 0.81, p value < 0.0005; Fig. 6). In particular, transcripts involved in signaling pathways (CAMK, PP2C and SBP), transport (AMT and ABC), stress and antioxidant responses (GST, CAT and HSP20), and carbon metabolism (PPCK) were up-regulated (p < 0.05 for CAMK, PP2C, SBP, GST and PPCK; p < 0.001 for CAT and HSP20). The 3,5-cyclic nucleotide phosphodiesterase (PDE), involved in purine metabolism, and the Elmo-domain-containing protein 3 (ELMOD3), which acts as a GTPase-activating protein, were up-regulated as well (p < 0.05 for ELMOD3 and p < 0.01 for PDE).

On the contrary, lipoxygenase (the LPX sequence which was down-regulated by 3.065 log2 x-fold in the transcriptome differential expression analysis was selected for primer design for the RT-qPCR) and polyketide synthase, both involved in the synthesis of secondary metabolites, and nitrilase, involved in nitrile bioremediation, were significantly down-regulated (p < 0.001 for all). RT-qPCR data further confirmed the up/down regulation of these transcripts in N starvation (Fig. 6) thus demonstrating the reliability of the high-throughput results.

## Conclusion

Summarizing, what makes this study interesting is the fact that we give a series of details regarding the molecular response of *T. suecica* providing a bigger picture of the nitrogen starvation story. For example, *T. suecica* does not activate transcripts involved in lipid biosynthesis under nitrogen starvation as other green algae^4,5^ such as *C. reinhardtii*^72^ and *Tetraselmis* sp. M8^20^. *T. suecica* is also able to activate stress and antioxidant transcripts as well as signalling and solute transporter transcripts indicating the activation of a series of defense and adaptation strategies to maintain cellular homeostasis and survival.

Our study also identifies enzymes that have never been reported before in *T. suecica*, such as nitrilase and various PKS domains. Nitrilase, involved in nitrile detoxification^95,96^, has potential enzymatic bioremediation applications to clean up hazardous environmental pollutants^97^. Many of the known nitrilases possess various disadvantages, such as insufficient stability, selectivity or low specific activities, preventing their application, and there is therefore a constant demand for new nitrilases. Our results suggest a new possible source of nitrilase.

On the other hand, PKS enzymes are known to be involved in the synthesis of compounds with anti-infective and antiproliferative activities, with possible pharmaceutical applications. The presence of PKS domains in this species suggests the production of still unknown polyketides. Until now most of the known polyketides have been identified in dinoflagellates^80^ whereas our study indicates that they may be widely spread in other microalgal groups as well. This study confirms that transcriptomic approaches are not only useful for physiological studies but also have the power to discover gene clusters that can be involved in the production of novel metabolites.

## Supplementary information

Supplementary information

## Data Availability

Data are available and sequences are deposited in GenBank.

## References

[1] Falkowski P (1994). The role of phytoplankton photosynthesis in global biogeochemical cycles. Photosynth Res..

[2] Huppe HC, Turpin DH (1994). Integration of carbon and nitrogen metabolism in plant and algal cells. Annu. Rev. Plant Biol..

[3] Hong SJ (2017). Enhanced production of fatty acids in three strains of microalgae using a combination of nitrogen starvation and chemical inhibitors of carbohydrate synthesis. Biotechnol. Bioprocess Eng..

[4] Kamalanathan M, Pierangelini M, Shearman LA, Gleadow R, Beardall J (2016). Impacts of nitrogen and phosphorus starvation on the physiology of *Chlamydomonas reinhardtii*. J. Appl. Phycol..

[5] Rios LF, Klein BC, Luz LF, Maciel Filho R, Wolf Maciel MR (2015). Nitrogen starvation for lipid accumulation in the microalga species *Desmodesmus* sp. Appl. Biochem. Biotechnol..

[6] Wang X, Shen Z, Miao X (2016). Nitrogen and hydrophosphate affects glycolipids composition in microalgae. Sci. Rep..

[7] Hockin NL, Mock T, Mulholland F, Kopriva S, Malin G (2012). The response of diatom central carbon metabolism to nitrogen starvation is different from that of green algae and higher plants. Plant Physiol..

[8] Simionato D (2013). The response of *Nannochloropsis gaditana* to nitrogen starvation includes *de novo* biosynthesis of triacylglycerols, a decrease of chloroplast galactolipids, and reorganization of the photosynthetic apparatus. Eukaryot Cell..

[9] Buckingham, J., Cooper, C. M. & Purchase, R. Natural Products Desk Reference. (Ed. Buckingham, J.) (CRC Press, Taylor & Francis group, 2015).

[10] Pérez-López P (2014). Life cycle assessment of the production of bioactive compounds from *Tetraselmis suecica* at pilot scale. J. Clean. Prod..

[11] Romano G (2016). Marine microorganisms as a promising and sustainable source of bioactive molecules. Mar. Environ. Res..

[12] Sansone C (2017). The green microalga *Tetraselmis suecica* reduces oxidative stress and induces repairing mechanisms in human cells. Scientific Reports.

[13] Muller-Feuga, A., Robert, R., Cahu, C., Robin, J. & Divanach, P. Uses of microalgae in aquaculture, Live Feeds in Marine Aquaculture (Eds Stottrup, J. G. & McEvoy, L. A.) 253–299 (Blackwell, Oxford; 2003).

[14] Austin B, Baudet E, Stobie M (1992). Inhibition of bacterial fish pathogens by *Tetraselmis suecica*. J. Fish. Dis..

[15] Carballo-Cárdenas EC, Tuan PM, Janssen M, Wijffels RH (2003). Vitamin E (α-tocopherol) production by the marine microalgae *Dunaliella tertiolecta* and *Tetraselmis suecica* in batch cultivation. Biomol. Eng..

[16] Montero MF, Aristizábal M, Reina GG (2011). Isolation of high-lipid content strains of the marine microalga *Tetraselmis suecica* for biodiesel production by flow cytometry and single-cell sorting. J. Appl. Phycol..

[17] Lauritano C (2016). Bioactivity screening of microalgae for antioxidant, anti-inflammatory, anticancer, anti-diabetes and antibacterial activities. Front. Mar. Sci..

[18] Lauritano C (2017). *De novo* transcriptome of the cosmopolitan dinoflagellate *Amphidinium carterae* to identify enzymes with biotechnological potential. Sci. Rep..

[19] Adarme-Vega TC, Thomas-Hall SR, Lim DK, Schenk PM (2014). Effects of long chain fatty acid synthesis and associated gene expression in microalga *Tetraselmis* sp. Mar. Drugs.

[20] Lim DKY (2017). RNA-Seq and metabolic flux analysis of *Tetraselmis* sp. M8 during nitrogen starvation reveals a two-stage lipid accumulation mechanism. Bioresour. Technol..

[21] Guillard, R. R. L. Culture of phytoplankton for feeding marine invertebrates, in Culture of Marine Invertebrate Animals, (eds Smith, W. L. & Chanley, M. H.), 26–60 (New York, NY:USA:Plenum Press, 1975).

[22] Escalera L (2010). Bloom dynamics of *Dinophysis acuta* in an upwelling system: *In situ* growth versus transport. Harmful Algae.

[23] Lauritano C, Orefice I, Procaccini G, Romano G, Ianora A (2015). Key Genes as Stress Indicators in the Ubiquitous Diatom *Skeletonema marinoi*. BMC Genomics.

[24] Grabherr MG (2011). Full-length transcriptome assembly from RNA-Seq data without a reference genome. Nat. Biotechnol..

[25] Schulz MH, Zerbino DR, Vingron M, Birney E (2012). Oases: Robust *de novo* RNA-seq assembly across the dynamic range of expression levels. Bioinformatics.

[26] Conesa A (2005). Blast2GO: a universal tool for annotation, visualization and analysis in functional genomics research. Bioinformatics.

[27] Li B, Dewey CN (2011). RSEM: accurate transcript quantification from RNA-Seq data with or without a reference genome. BMC Bioinform..

[28] Anders S, Huber W (2010). Differential expression analysis for sequence count data. Genome Biol..

[29] Ogata H (1999). KEGG: Kyoto Encyclopedia of Genes and Genomes. Nucleic Acids Res..

[30] Barrett T (2013). NCBI GEO: archive for functional genomics data sets–update. Nucleic Acids Res..

[31] Lauritano C (2011). First molecular evidence of diatom effects in the copepod *Calanus helgolandicus*. J. Exp. Mar. Biol. Ecol..

[32] Pfaffl MW, Tichopad A, Prgomet C, Neuvians TP (2004). Determination of stable housekeeping genes, differentially regulated target genes and sample integrity: BestKeeper–Excel-based tool using pair-wise correlations. Biotechnol. Lett..

[33] Vandesompele, J. *et al*. Accurate normalization of real-time quantitative RT-PCR data by geometric averaging of multiple internal control genes. *Genome Biol*. **3**, RESEARCH0034; PMC126239 (2002). 10.1186/gb-2002-3-7-research0034 PMC126239 12184808

[34] Andersen CL, Jensen JL, Orntoft TF (2004). Normalization of real-time quantitative reverse transcription-PCR data: a model-based variance estimation approach to identify genes suited for normalization, applied to bladder and colon cancer data sets. Cancer Res..

[35] Pfaffl, M. W., Horgan, G. W. & Dempfle, L. Relative expression software tool (REST (c)) for group-wise comparison and statistical analysis of relative expression results in real-time PCR. *Nucleic Acids Res*. **3**0, e36, PMC113859 (2002). 10.1093/nar/30.9.e36 PMC113859 11972351

[36] Stephens, M. A. Tests based on edf statistics in Goodness-of-Fit Techniques (eds D’Agostino, R. B. & Stephens, M. A.) 97–194 (Marcel Dekker, 1986).

[37] Hammer Ø, Harper DAT, Ryan PD (2001). PAST: Paleontological statistics software package for education and data analysis. Palaeontol. Electron..

[38] Gasteiger E (2003). ExPASy: the proteomics server for in-depth protein knowledge and analysis. Nucleic Acids Res..

[39] Howden AJ, Harrison CJ, Preston GM (2009). A conserved mechanism for nitrile metabolism in bacteria and plants. Plant J..

[40] Larkin MA (2007). Clustal W and Clustal X version 2.0. Bioinformatics.

[41] Stamatakis A (2006). RAxML-VI-HPC: maximum likelihood-based phylogenetic analyses with thousands of taxa and mixed models. Bioinformatics.

[42] Lanfear R, Calcott B, Ho SY, Guindon S (2012). PartitionFinder: combined selection of partitioning schemes and substitution models for phylogenetic analyses. Mol. Biol. Evol..

[43] Bartels S, Besteiro MAG, Lang D, Ulm R (2010). Emerging functions for plant MAP kinase phosphatases. Trends Plant Sci..

[44] Roitinger E (2015). Quantitative phosphoproteomics of the ataxia telangiectasia-mutated (ATM) and ataxia telangiectasia-mutated and rad3-related (ATR) dependent DNA damage response in *Arabidopsis thaliana*. Mol. Cell. Proteomics.

[45] Suarez MC, Petersen M, Mundy J (2010). Mitogen-activated protein kinase signaling in plants. Annu. Rev. Plant Biol..

[46] Theodosiou A, Ashworth A (2002). MAP kinase phosphatases. Genome Biol..

[47] Cook JG, Bardwell L, Thorner J (1997). Inhibitory and activating functions for MAPK Kss1 in the *S. cerevisiae* filamentous-growth signalling pathway. Nature.

[48] Chen RE, Thorner J (2007). Function and regulation in MAPK signaling pathways: lessons learned from the yeast *Saccharomyces cerevisiae*. Biochim. Biophys. Acta.

[49] Kohut G, Ádám AL, Fazekas B, Hornok L (2009). N-starvation stress induced FUM gene expression and fumonisin production is mediated via the HOG-type MAPK pathway in *Fusarium proliferatum*. Int. J. Food Microbiol..

[50] Keyse SM (2008). The regulation of stress-activated MAP kinase signalling by protein phosphatases. Top. Curr. Genet..

[51] Rodriguez PL (1998). Protein phosphatase 2C (PP2C) function in higher plants. Plant Mol. Biol..

[52] Moorhead GBG, Trinkle-Mulcahy L, Ulke-Lemée A (2007). Emerging roles of nuclear protein phosphatases. Nat. Rev. Mol. Cell Biol..

[53] González A, Ruiz A, Serrano R, Ariño J, Casamayor A (2006). Transcriptional profiling of the protein phosphatase 2C family in yeast provides insights into the unique functional roles of Ptc1. J. Biol. Chem..

[54] Schweighofer A (2007). The PP2C-type phosphatase AP2C1, which negatively regulates MPK4 and MPK6, modulates innate immunity, jasmonic acid, and ethylene levels in Arabidopsis. Plant Cell.

[55] Umbrasaite J (2010). MAPK phosphatase AP2C3 induces ectopic proliferation of epidermal cells leading to stomata development in *Arabidopsis*. PloS one.

[56] Chen X (2010). SQUAMOSA promoter‐binding protein‐like transcription factors: Star players for plant growth and development. J. Integr. Plant. Biol..

[57] Preston JC, Hileman LC (2013). Functional evolution in the plant Squamosa-promoter binding protein-like (SPL) gene family. Front. Plant Sci..

[58] Kang LK, Chang J (2014). Sequence diversity of ammonium transporter genes in cultured and natural species of marine phytoplankton. J. Mar. Sci. Tech..

[59] Gonzalez-Ballester D, Camargo A, Fernández E (2004). Ammonium transporter genes in *Chlamydomonas*: the nitrate-specific regulatory gene Nit2 is involved in Amt1; 1 expression. Plant Mol. Biol..

[60] Song T, Gao Q, Xu Z, Song R (2011). The cloning and characterization of two ammonium transporters in the salt-resistant green alga. Dunaliella viridis. Mol. Biol. Rep..

[61] Kang LK (2007). Influences of nitrogen deficiency on the transcript levels of ammonium transporter, nitrate transporter and glutamine synthetase genes in *Isochrysis galbana* (Isochrysidales, Haptophyta). Phycol..

[62] McDonald SM, Plant JN, Worden AZ (2010). The mixed lineage nature of nitrogen transport and assimilation in marine eukaryotic phytoplankton: a case study of *Micromonas*. Mol. Biol. Evol..

[63] Hildebrand M (2005). Cloning and functional characterization of ammonium transporters from the marine diatom *Cylindrotheca fusiformis* (Bacillariophyceae). J. Phycol..

[64] Howitt SM, Udvardi MK (2000). Structure, function and regulation of ammonium transporters in plants. Biochim. Biophys. Acta.

[65] Sorensen JG, Kristensen TN, Loeschcke V (2003). The evolutionary and ecological role of heat shock proteins. Ecol. Lett..

[66] Lauritano C, Procaccini G, Ianora A (2012). Gene Expression Patterns and Stress Response in Marine Copepods. Mar. Environ. Res..

[67] Longworth J, Wu D, Huete-Ortega M, Wright PC, Vaidyanathan S (2016). Proteome response of *Phaeodactylum tricornutum*, during lipid accumulation induced by nitrogen depletion. Algal Res..

[68] Safi, A. *et al*. HRS1/HHOs GARP transcription factors and reactive oxygen species are regulators of *Arabidopsis* nitrogen starvation response. *bioRxiv*, 164277, 10.1101/164277 (2018).

[69] Zhang YM, Chen H, He CL, Wang Q (2013). Nitrogen starvation induced oxidative stress in an oil-producing green alga *Chlorella sorokiniana* C3. PloS one.

[70] Ruiz-Dominguez MC (2014). Lipid accumulation and antioxidant activity in the eukaryotic acidophilic microalga *Coccomyxa* sp. (strain *onubensis*) under nutrient starvation. J. Appl. Phycol..

[71] Velmurugan N (2014). Systematically programmed adaptive evolution reveals potential role of carbon and nitrogen pathways during lipid accumulation in *Chlamydomonas reinhardtii*. Biotechnol. Biofuels.

[72] Miller R (2010). Changes in transcript abundance in *Chlamydomonas reinhardtii* following nitrogen deprivation predict diversion of metabolism. Plant Physiol..

[73] Hartwell J (1999). Phosphoenolpyruvate carboxylase kinase is a novel protein kinase regulated at the level of expression. Plant J..

[74] Bourne Y, Henrissat B (2001). Glycoside hydrolases and glycosyltransferases: families and functional modules. Curr. Opin. Struct. Biol..

[75] Mu J (2015). Enhanced biomass and oil production from sugarcane bagasse hydrolysate (SBH) by heterotrophic oleaginous microalga *Chlorella protothecoides*. Bioresour. Technol..

[76] Pival SL, Birner-Gruenberger R, Krump C, Nidetzky B (2011). d-Xylulose kinase from *Saccharomyces cerevisiae*: Isolation and characterization of the highly unstable enzyme, recombinantly produced in *Escherichia coli*. Protein Expr. Purif..

[77] Hemmerlin A (2006). A cytosolic Arabidopsis D-xylulose kinase catalyzes the phosphorylation of 1-deoxy-D-xylulose into a precursor of the plastidial isoprenoid pathway. Plant Physiol..

[78] de Jesus Raposo MF, de Morais AMB, de Morais RMSC (2015). Marine polysaccharides from algae with potential biomedical applications. Mar. Drugs.

[79] Miralto A (1999). The insidious effect of diatoms on copepod reproduction. Nature.

[80] Kobayashi J (2008). Amphidinolides and its related macrolides from marine dinoflagellates. J. Antibiot..

[81] Shelest E, Heimerl N, Fichtner M, Sasso S (2015). Multimodular type I polyketide synthases in algae evolve by module duplications and displacement of AT domains in trans. BMC Genomics.

[82] Tudzynski B (2014). Nitrogen regulation of fungal secondary metabolism in fungi. Front. Microbiol..

[83] Nott A, Jung HS, Koussevitzky S, Chory J (2006). Plastid-to nucleus retrograde signaling. Ann. Rev. Plant Biol..

[84] Pruneda-Paz JL, Kay SA (2010). An expanding universe of circadian networks in high plants. Trends Plant Sci..

[85] Martin T, Oswald O, Graham IA (2002). *Arabidopsis* seedling growth, storage lipid mobilization, and photosynthetic gene expression are regulated by carbon: nitrogen availability. Plant Physiol..

[86] Richter S, Lamppa GK (1998). A chloroplast processing enzyme functions as the general stromal processing peptidase. Proc. Natl. Acad. Sci. USA.

[87] Azumi Y, Watanabe A (1991). Evidence for a senescence-associated gene induced by darkness. Plant Physiol..

[88] Caplan JL, Mamillapalli P, Burch-Smith TM, Czymmek K, Dinesh-Kumar SP (2008). Chloroplastic protein NRIP1 mediates innate immune receptor recognition of a viral effector. Cell.

[89] Jurić S (2009). Tethering of ferredoxin: NADP+ oxidoreductase to thylakoid membranes is mediated by novel chloroplast protein TROL. Plant J..

[90] Aliverti A, Pandini V, Pennati A, de Rosa M, Zanetti G (2008). Structural and functional diversity of ferredoxin-NADP+ reductases. Arch. Biochem. Biophys..

[91] Wase N, Black PN, Stanley BA, Di Russo CC (2014). Integrated quantitative analysis of nitrogen stress response in *Chlamydomonas reinhardtii* using metabolite and protein profiling. J. Proteome Res..

[92] Machado M (2016). Comprehensive metabolic reprograming in freshwater *Nitzschia palea* strains undergoing nitrogen starvation is likely associated with its ecological origin. Algal Res..

[93] Zhao LS (2017). Nitrogen starvation impacts the photosynthetic performance of *Porphyridium cruentum* as revealed by chlorophyll a fluorescence. Sci. Rep..

[94] Juergens MT (2015). The regulation of photosynthetic structure and function during nitrogen deprivation in *Chlamydomonas reinhardtii*. Plant physiol..

[95] Gupta V, Gaind S, Verma PK, Sood N, Srivastava AK (2010). Purification and characterization of intracellular nitrilases from *Rhodococcus* sp. - potential role of periplasmic nitrilase. Afr. J. Microbiol. Res..

[96] Ramakrishna C, Dave H, Ravindranathan M (1999). Microbial metabolism of nitriles and its biotechnological potential. J. Sci. Ind. Res..

[97] Brandao PFB, Bull AT (2003). Nitrile hydrolyzing activities of deep-sea and terrestrial mycolate actinomycetes. Antonie van Leeuwenhoek.

[98] Nakada Y, Jiang Y, Nishijyo T, Itoh Y, Lu CD (2001). Molecular characterization and regulation of theaguBA operon, responsible for agmatine utilization in *Pseudomonas aeruginosa* PAO1. J. Bacteriol..

[99] Bestwick LA, Groning LM, James DC, Bones A, Rossiter JT (1993). Purification and characterization of a nitrilase from *Brassica napus*. Physiol. Plant.

[100] Piotrowski M, Schonfelder S, Weiler ER (2001). The *Arabidospsis thaliana* isogene NIT4 and its orthologs in Tobacco encode b-cyano-L-alanine hydratase ⁄ nitrilase. J. Biol. Chem..

[101] Kutz A (2002). A role for nitrilase 3 in the regulation of root morphology in sulphur‐starving *Arabidopsis thaliana*. Plant J..

[102] Brenner C (2002). Catalysis in the nitrilase superfamily. Curr. Opin. Struct. Biol..

[103] Piotrowski M (2008). Primary or secondary? Versatile nitrilases in plant metabolism. Phytochemistry..

