# Supplementary material for: New molecular insights on the response of the green alga Tetraselmis suecica to nitrogen starvation
Source: Sci Rep. 2019 Mar 4;9:3336. doi: 10.1038/s41598-019-39860-5 (PMC6399242; doi:10.1038/s41598-019-39860-5)
Supplement: Supplementary file 1 — Supplementary information [file 41598_2019_39860_MOESM1_ESM.pdf]

New molecular insights on the response of the green alga *Tetraselmis suecica* to nitrogen starvation

Chiara Lauritano[1*], Daniele De Luca[2], Mariano Amoroso[1], Salvatore Benfatto[3], Simone Maestri[3], Claudia Racioppi[1,4], Francesco Esposito[1], Adrianna Ianora[1*]

[1] Marine Biotechnology Department, Stazione Zoologica Anton Dohrn, Villa Comunale, 80121, Napoli, Italy

[2] Integrative Marine Ecology Department, Stazione Zoologica Anton Dohrn, Villa Comunale, 80121, Napoli, Italy

[3] Università degli Studi di Verona, Ca' Vignal 1 Strada Le Grazie 15, 37134, Verona, Italy

[4] Center for Developmental Genetics, Department of Biology, College of Arts and Science, New York University, New York, USA

[*]Corresponding authors:

Chiara Lauritano, Marine Biotechnology Department, Stazione Zoologica Anton Dohrn, Villa Comunale, 80121, Napoli, Italy; chiara.lauritano@szn.it; Tel: 00395833221; Fax: 0039 0817641355

Adrianna Ianora, Marine Biotechnology Department, Stazione Zoologica Anton Dohrn, Villa Comunale, 80121, Napoli, Italy; adrianna.ianora@szn.it; Tel: 00395833246; Fax: 0039 0817641355

Supplementary information

Figure S1 Histograms of GO classifications showing sequence distribution of total transcripts within cellular component (a), biological process (b) and molecular function (c). The y-axis indicates the percentage of sequences for each category.

Table S1 List of selected reference genes (RG) and genes of interest (GOI) with their abbreviations (Abbr), functions, primer's sequences and efficiencies.

Table S2 Reference genes ranking from the most to the least stable according to NormFinder, BestKeeper and Genorm softwares.

Table S3 BUSCO analysis results. Lineage dataset eukariota_odb9 was used. Out of 303 BUSCOs 68.4% complete entries were identified (44.6% single and 23.8% duplicated) and 4.0% were detected as fragments.

Table S4 List of KEGG pathways associated to contigs with assigned enzyme codes in differentially regulated genes. The table contains pathway names, pathway ID, number of enzymes found for each pathway, enzyme codes and sequence names.

Table S5 DEG id, log2 Fold Change, adjusted P value (padj), sequence description and enzyme codes ($|LogFC| > 2$, padj ≤ 0.01).

Table S6 List of nitrilase and nitrilase-like sequences retrieved from public repositories utilised to generate the ML tree.

Figure S1. Histograms of GO classifications showing sequence distribution of total transcripts within cellular component (a), biological process (b) and molecular function (c). The y-axis indicates the percentage of sequences for each category.

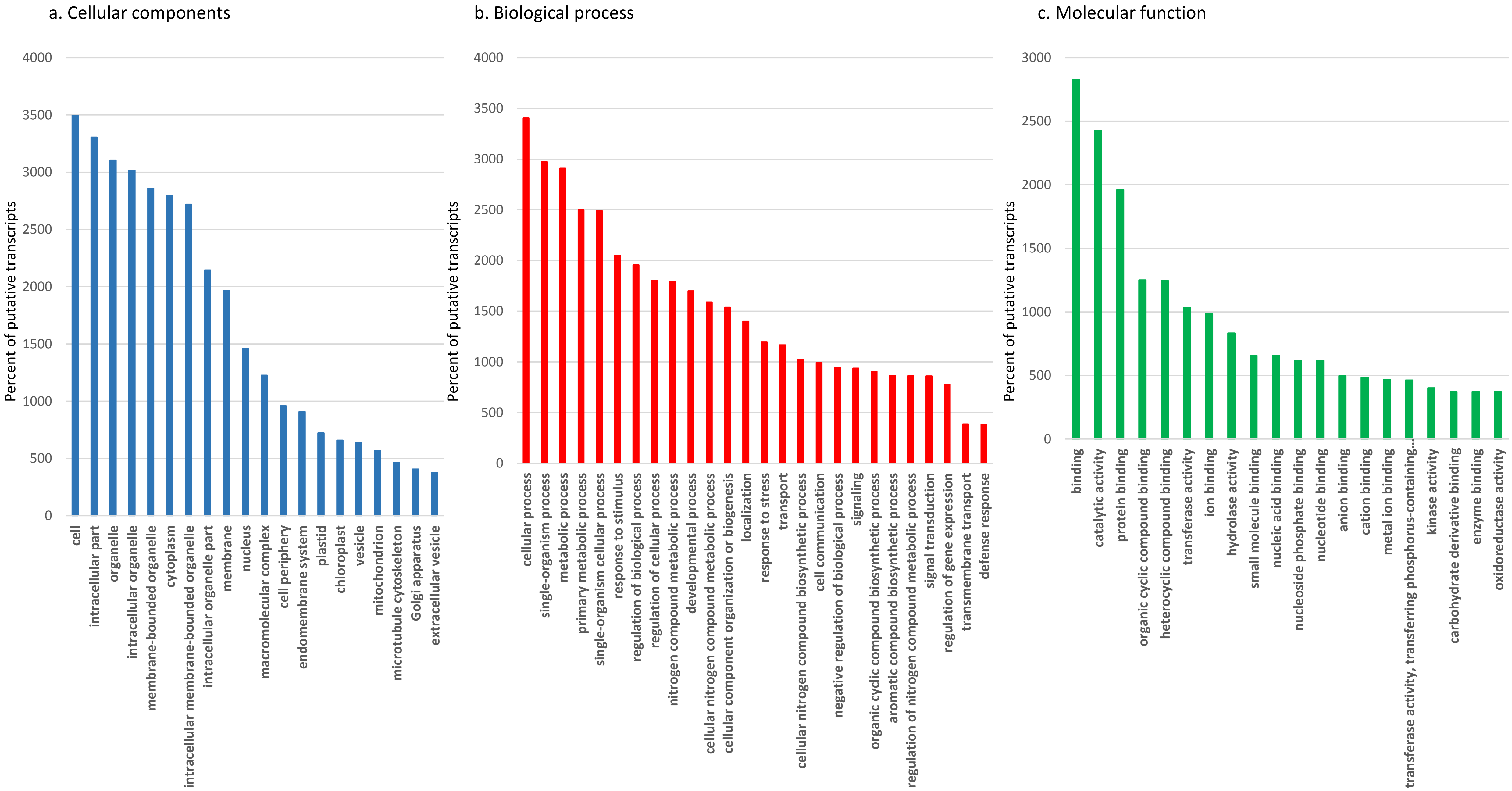

Table S1. List of selected reference genes (RG) and genes of interest (GOI) with their abbreviations (Abbr), functions, primer's sequences and efficiencies.

Gene Name	Abbr	Function	Primer F	Primer R	E
Reference Genes (RGs)					
Actin	ACT	Cytoskeleton microfilaments	CACATCTGCTGGAAGGTCGA	GCATTGCGGACCGTATGAAC	2
α tubulin	ATUB	Microtubule subunit	AAATGGATGCGTGGGTAGGG	CCCACGTACACCAACCTGAA	2
β tubulin	BTUB	Microtubule subunit	AATGCAGACGAGGAAAGGGG	TTATCTCCGCCGTCATGTCG	2
Glyceraldehyde 3-phosphate dehydrogenase	GAPDH	Glycolysis	GGCGTGTACTGTGGTCATCA	TGTGATGGGTGTGAACGAGG	2
Histone 1	H1	Packaging DNA in nucleosomes	GCTGAACCGCCAAAACCTTT	TCACCCCAAGTACGAGGTCA	2
Histone 4	H4	Packaging DNA in nucleosomes	TGGATGGCGCAAAGGTTAGT	TCTACCGAGCTCCTCATCCG	2
Genes Of Interest (GOI)					
Polyketide synthase	PKS	Polyketide synthesis	CCCAAACGGTCCATCACAGA	AGCAAGACTTGACTGAGCCC	2
Lipoxygenase	LPX	Lipid metabolism	CTCGACAAGAACCACGACGA	CGCTCGAAGTTATCCCTGCT	2
Squamosa promoter binding protein	SBP	Transcription factor	CAGAGTGTGCGTCGAGTGTA	GTCCCGTCAAAGTCACCGAT	2
Elmo-domain-containing protein 3	ELMOD3	Acts as a GTPase-activating protein (GAP)	GGTAGAGGAGCTGCATGAGG	ACTTCAAGATCCTGTCGGCC	2
3 5-cyclic nucleotide phosphodiesterase	PDE	Purine metabolism	CGTTGAGGCTGTGCATTTCC	TGTTAACGCGCTCAACTTGC	2
ATP-binding cassette protein transporter	ABC	Transporter	GGCTGTACTCCGATCCCAAG	TGATCTCGTGCATGCTCCAG	2
Protein phosphatase 2c family	PP2C	Serine/threonine specific	AGCCTGTTCTGTCGCATGAT	CCGCACGTCACACAGGTTAA	2

protein		protein phosphatases			
Calcium/calmodulin-dependent protein kinase type 1	CAMK	Calcium-triggered CaMKK-CaMK1 signaling cascade	TCGAAAGGCTGCCGTATTGT	GCACGAGAGATTATCCGCCA	2
Phosphoenolpyruvate carboxylase kinase	PPCK	Light-dependent phosphoenolpyruvate carboxylase phosphorylation	TTCCTTGGTCGCCGTGTATC	GCACGGCGACATAAAACCTG	2
Heat shock protein 20	HSP20	Stress response	CTGCCCTTCGTTCGTCATCT	GAAGGACGAGGATGGCGAG	2
Ammonium transporter	AMT	Transporter	AAGCAATCCTGTCGCCAAGA	TTCGAGCCCTACATAGCCCT	2
Catalase	CAT	Antioxidant	AGGTCCACGAACTTCACAGC	GTGACCTCCGCTGATATGGG	2
Glutathione-S-transferase	GST	Antioxidant	CACCGTGTCGTACAGCTTCT	GAGCATCTTCGACTCGCAGT	2
Nitrilase	NIT1	Catalyse the hydrolysis of nitriles to carboxylic acids and ammonia	TAGCTCTGCGTCTCGATTGC	TGCTATGACCTTCGCTTCCC	2

Table S2. Reference genes ranking from the most to the least stable according to NormFinder, BestKeeper and Genorm softwares. The best RGs identified are represented in bold.

Ranking	NormFinder	Genorm	BestKeeper
1	**Histone 1**	**Actin**	**Actin**
2	β tubulin	**β tubulin**	Histone 4
3	GAPDH	Histone 1	β tubulin
4	Actin	GAPDH	Histone 1
5	α tubulin	Histone 4	GAPDH
6	Histone 4	α tubulin	α tubulin

Table S3. BUSCO analysis results. Lineage dataset eukariota_odb9 was used. Out of 303 BUSCOs 68.4% complete entries were identified (44.6% single and 23.8% duplicated) and 4.0% were detected as fragments.

# Busco id	Status	Sequence	Score	Length
EOG0937004M	Complete	Sample3_Locus_11118_Transcript_1_2_Confidence_0.667_Length_3004	1440.8	826
EOG09370082	Complete	Sample1_Locus_20518_Transcript_3_3_Confidence_0.000_Length_3552	1705.5	1046
EOG093700N7	Fragmented	SampleTS1_Locus_17730_Transcript_3_4_Confidence_0.400_Length_1023	462.6	295
EOG0937017X	Complete	Sample3_Locus_4090_Transcript_10_14_Confidence_0.353_Length_4125	842.8	1281
EOG0937018Z	Complete	Sample6_Locus_980_Transcript_11_12_Confidence_0.214_Length_3072	1098.3	798
EOG0937019B	Complete	Sample1_Locus_13436_Transcript_4_7_Confidence_0.200_Length_2137	1188.9	662
EOG093701EE	Complete	Sample1_Locus_2485_Transcript_2_3_Confidence_0.333_Length_3529	730.2	740
EOG093701IQ	Duplicated	Sample1_Locus_17374_Transcript_1_2_Confidence_0.333_Length_997	361.8	246
EOG093701IQ	Duplicated	Sample6_Locus_10491_Transcript_7_7_Confidence_0.125_Length_4037	326.3	394
EOG093701IQ	Duplicated	Sample6_Locus_6166_Transcript_4_5_Confidence_0.400_Length_3519	357.9	362
EOG093701S0	Complete	Sample6_Locus_19560_Transcript_2_3_Confidence_0.333_Length_2582	1053.6	703
EOG093701SQ	Complete	Sample6_Locus_722_Transcript_2_13_Confidence_0.026_Length_1793	924.1	537
EOG093701V7	Fragmented	Sample1_Locus_6950_Transcript_1_1_Confidence_0.000_Length_844	333.5	223
EOG0937029K	Complete	Sample6_Locus_1890_Transcript_5_7_Confidence_0.143_Length_4516	298.5	602
EOG093702AT	Fragmented	Sample6_Locus_3889_Transcript_3_5_Confidence_0.375_Length_822	192.3	134
EOG093702DP	Fragmented	SampleTS1_Locus_3621_Transcript_2_4_Confidence_0.143_Length_620	155.5	174
EOG093702MJ	Complete	Sample1_Locus_1240_Transcript_5_7_Confidence_0.125_Length_2907	258.8	433
EOG093702PE	Fragmented	SampleTS1_Locus_1308_Transcript_2_3_Confidence_0.400_Length_666	57.1	85
EOG0937033T	Complete	Sample6_Locus_1784_Transcript_26_30_Confidence_0.000_Length_2728	119.2	345
EOG0937036L	Fragmented	SampleTS1_Locus_24681_Transcript_1_2_Confidence_0.333_Length_976	269.1	244
EOG093703C8	Complete	Sample6_Locus_10055_Transcript_3_5_Confidence_0.400_Length_2109	718.8	496
EOG093703DY	Complete	Sample6_Locus_15394_Transcript_3_8_Confidence_0.091_Length_1439	174.1	260
EOG093703N5	Fragmented	SampleTS2_Locus_15206_Transcript_1_1_Confidence_0.000_Length_646	65.4	107
EOG093703UX	Complete	Sample6_Locus_7364_Transcript_2_3_Confidence_0.333_Length_2406	889	592
EOG093703W1	Fragmented	Sample6_Locus_7766_Transcript_4_9_Confidence_0.111_Length_1615	122.4	275

EOG093703WD	Complete	Sample1_Locus_20679_Transcript_7_7_Confidence_0.143_Length_2696	549.9	586
EOG093703YA	Complete	Sample3_Locus_23468_Transcript_1_4_Confidence_0.100_Length_1497	530	412
EOG0937049Y	Fragmented	SampleTS1_Locus_21779_Transcript_1_1_Confidence_0.000_Length_378	43.1	73
EOG093704GD	Duplicated	Sample3_Locus_9915_Transcript_1_3_Confidence_0.667_Length_653	153.5	177
EOG093704GD	Duplicated	Sample6_Locus_825_Transcript_2_4_Confidence_0.500_Length_878	137.9	182
EOG093704HC	Complete	Sample1_Locus_10788_Transcript_1_1_Confidence_0.000_Length_1565	729.9	396
EOG093704J7	Missing			
EOG093704Q0	Complete	SampleTS3_Locus_2622_Transcript_10_32_Confidence_0.074_Length_1553	327.6	270
EOG093704UY	Missing			
EOG0937050C	Complete	Sample1_Locus_15155_Transcript_4_5_Confidence_0.286_Length_1354	312.8	346
EOG093705DM	Complete	Sample3_Locus_7619_Transcript_2_4_Confidence_0.400_Length_1120	335.8	281
EOG093705E5	Complete	Sample6_Locus_18230_Transcript_3_7_Confidence_0.364_Length_1443	227.1	342
EOG093705EY	Complete	Sample1_Locus_1466_Transcript_4_5_Confidence_0.286_Length_2081	611.1	478
EOG093705KN	Complete	SampleTS1_Locus_4695_Transcript_2_2_Confidence_0.000_Length_1914	459	394
EOG093705U4	Complete	Sample3_Locus_2613_Transcript_4_6_Confidence_0.333_Length_970	172.5	190
EOG093705VV	Complete	SampleTS2_Locus_9621_Transcript_1_1_Confidence_0.000_Length_1253	421.8	332
EOG093705YA	Fragmented	Sample3_Locus_11212_Transcript_2_4_Confidence_0.500_Length_1034	332.1	272
EOG093705YE	Fragmented	SampleTS1_Locus_20485_Transcript_2_6_Confidence_0.143_Length_517	32.6	63
EOG093705Z6	Complete	SampleTS2_Locus_13865_Transcript_1_2_Confidence_0.333_Length_1158	638.7	329
EOG0937060I	Duplicated	Sample6_Locus_12501_Transcript_1_2_Confidence_0.333_Length_2191	493	587
EOG0937060I	Duplicated	SampleTS1_Locus_8184_Transcript_3_5_Confidence_0.400_Length_3710	562.4	721
EOG0937066G	Complete	Sample3_Locus_4849_Transcript_2_3_Confidence_0.333_Length_1992	628.7	408
EOG0937068H	Complete	Sample3_Locus_10257_Transcript_10_12_Confidence_0.455_Length_6913	161.8	439
EOG093706PM	Complete	Sample3_Locus_11553_Transcript_2_6_Confidence_0.167_Length_2250	335.7	328
EOG093706RN	Fragmented	Sample3_Locus_10779_Transcript_4_5_Confidence_0.143_Length_1310	210.6	337
EOG093706X0	Complete	SampleTS3_Locus_20404_Transcript_5_8_Confidence_0.667_Length_1756	251.7	296
EOG09370762	Complete	Sample1_Locus_2510_Transcript_3_5_Confidence_0.667_Length_1885	797.5	487
EOG093707B7	Fragmented	SampleTS2_Locus_20902_Transcript_1_1_Confidence_0.000_Length_474	99.2	101
EOG093707HK	Fragmented	Sample1_Locus_26365_Transcript_2_3_Confidence_0.333_Length_947	280.7	224

EOG093707LO	Complete	Sample6_Locus_12744_Transcript_3_4_Confidence_0.200_Length_2047	304.3	402
EOG093707RF	Complete	Sample6_Locus_2442_Transcript_4_16_Confidence_0.038_Length_1846	281	331
EOG093707VN	Fragmented	Sample1_Locus_20248_Transcript_1_1_Confidence_0.000_Length_738	278.5	182
EOG093707VQ	Duplicated	Sample6_Locus_5293_Transcript_2_14_Confidence_0.074_Length_2711	280	522
EOG093707VQ	Duplicated	SampleTS1_Locus_8872_Transcript_7_7_Confidence_0.250_Length_2752	321	558
EOG0937084T	Complete	SampleTS1_Locus_15589_Transcript_3_4_Confidence_0.286_Length_1029	204.3	255
EOG0937085E	Complete	SampleTS1_Locus_1272_Transcript_7_12_Confidence_0.250_Length_1964	450.4	414
EOG093708IM	Fragmented	Sample6_Locus_19850_Transcript_2_5_Confidence_0.375_Length_1242	303.6	223
EOG093708OP	Complete	Sample1_Locus_9565_Transcript_1_1_Confidence_0.000_Length_1596	604.9	369
EOG093708TP	Complete	Sample6_Locus_24894_Transcript_2_4_Confidence_0.750_Length_1312	217.4	237
EOG0937091Y	Complete	SampleTS3_Locus_3183_Transcript_1_2_Confidence_0.333_Length_2485	556.3	520
EOG0937092T	Complete	SampleTS3_Locus_5343_Transcript_3_5_Confidence_0.286_Length_2471	227.7	338
EOG0937092W	Complete	Sample6_Locus_6313_Transcript_3_3_Confidence_0.000_Length_2171	334.5	423
EOG093709BZ	Complete	SampleTS1_Locus_1827_Transcript_15_16_Confidence_0.043_Length_2672	596.9	437
EOG093709HP	Complete	SampleTS3_Locus_6625_Transcript_3_3_Confidence_0.000_Length_1806	369.8	366
EOG093709JG	Complete	Sample1_Locus_15708_Transcript_1_9_Confidence_0.055_Length_1007	281.4	246
EOG09370A22	Complete	Sample1_Locus_15645_Transcript_2_4_Confidence_0.333_Length_1001	404.3	255
EOG09370ADO	Complete	Sample6_Locus_891_Transcript_1_8_Confidence_0.375_Length_1651	603.2	367
EOG09370AI8	Complete	Sample1_Locus_7596_Transcript_1_1_Confidence_0.000_Length_1647	228.6	248
EOG09370AJP	Complete	Sample1_Locus_3414_Transcript_3_5_Confidence_0.600_Length_1809	545.7	398
EOG09370AL3	Missing			
EOG09370ARO	Complete	SampleTS2_Locus_1325_Transcript_1_7_Confidence_0.667_Length_1724	316.8	339
EOG09370AS9	Complete	Sample6_Locus_9177_Transcript_2_3_Confidence_0.333_Length_793	182.5	199
EOG09370AV1	Complete	Sample1_Locus_3265_Transcript_2_2_Confidence_0.000_Length_1716	415.8	362
EOG09370AWB	Complete	Sample6_Locus_3336_Transcript_4_5_Confidence_0.400_Length_1554	392.7	338
EOG09370B1L	Complete	SampleTS3_Locus_3272_Transcript_6_6_Confidence_0.000_Length_1533	324.2	351
EOG09370B65	Complete	SampleTS1_Locus_14363_Transcript_1_2_Confidence_0.667_Length_886	96.5	145
EOG09370B7D	Complete	SampleTS3_Locus_6008_Transcript_4_5_Confidence_0.200_Length_1527	235.5	213

EOG09370BBR	Complete	Sample1_Locus_19788_Transcript_1_1_Confidence_0.333_Length_1516	641.6	340
EOG09370BGO	Complete	Sample6_Locus_20707_Transcript_2_3_Confidence_0.333_Length_881	137	165
EOG09370BJE	Complete	Sample1_Locus_4823_Transcript_3_6_Confidence_0.364_Length_1415	363.8	250
EOG09370BK5	Complete	Sample1_Locus_11386_Transcript_3_6_Confidence_0.667_Length_2134	173.3	361
EOG09370BQO	Complete	Sample6_Locus_13384_Transcript_2_4_Confidence_0.400_Length_1647	191.3	256
EOG09370BYX	Complete	Sample3_Locus_12500_Transcript_5_16_Confidence_0.086_Length_1337	236.4	264
EOG09370C5A	Complete	Sample1_Locus_5040_Transcript_6_59_Confidence_0.077_Length_2422	266.5	439
EOG09370CAN	Missing			
EOG09370CAV	Complete	Sample6_Locus_12402_Transcript_6_6_Confidence_0.125_Length_3559	508.1	526
EOG09370CF1	Complete	SampleTS2_Locus_16414_Transcript_2_4_Confidence_0.750_Length_1911	90.6	177
EOG09370CIV	Complete	SampleTS1_Locus_11785_Transcript_4_5_Confidence_0.400_Length_1437	220.9	204
EOG09370CK6	Missing			
EOG09370CKL	Complete	SampleTS1_Locus_23304_Transcript_2_3_Confidence_0.333_Length_2324	187.7	226
EOG09370CRF	Complete	Sample1_Locus_4733_Transcript_1_5_Confidence_0.286_Length_1406	506.4	372
EOG09370CTU	Complete	SampleTS1_Locus_5002_Transcript_1_4_Confidence_0.400_Length_1662	344.4	363
EOG09370CZ0	Complete	Sample1_Locus_10000_Transcript_3_4_Confidence_0.200_Length_1443	278.3	241
EOG09370CZT	Complete	Sample6_Locus_8448_Transcript_1_3_Confidence_0.667_Length_1466	126	182
EOG09370D13	Complete	Sample6_Locus_6913_Transcript_8_12_Confidence_0.538_Length_2852	347	424
EOG09370DO5	Complete	Sample1_Locus_8822_Transcript_1_5_Confidence_0.024_Length_1715	288.1	312
EOG09370DP4	Fragmented	Sample6_Locus_18310_Transcript_1_7_Confidence_0.111_Length_753	120	158
EOG09370DS4	Complete	SampleTS1_Locus_6708_Transcript_3_10_Confidence_0.579_Length_1242	96.5	125
EOG09370DTG	Complete	Sample1_Locus_6930_Transcript_2_4_Confidence_0.333_Length_1535	119.9	334
EOG09370DV4	Complete	SampleTS1_Locus_3854_Transcript_2_3_Confidence_0.333_Length_1402	349.9	290
EOG09370DX4	Complete	SampleTS2_Locus_12297_Transcript_1_1_Confidence_0.000_Length_1037	223.1	182
EOG09370DXT	Complete	SampleTS1_Locus_1053_Transcript_18_22_Confidence_0.463_Length_2338	86.3	157
EOG09370DYK	Fragmented	Sample3_Locus_7272_Transcript_4_4_Confidence_0.190_Length_655	49.3	55
EOG09370E0Y	Complete	Sample3_Locus_7465_Transcript_6_8_Confidence_0.455_Length_2812	475.5	330
EOG09370E36	Complete	Sample3_Locus_1751_Transcript_3_3_Confidence_0.000_Length_819	107.2	154
EOG09370E4U	Complete	SampleTS1_Locus_9264_Transcript_1_1_Confidence_0.000_Length_1433	324.6	240

EOG09370E4W	Complete	Sample1_Locus_9153_Transcript_1_4_Confidence_0.750_Length_1500	317.1	274
EOG09370E8S	Complete	Sample3_Locus_27250_Transcript_1_1_Confidence_0.000_Length_1764	283.9	299
EOG09370EHZ	Fragmented	SampleTS3_Locus_6897_Transcript_2_2_Confidence_0.333_Length_1282	206.9	233
EOG09370EL3	Complete	SampleTS1_Locus_14130_Transcript_1_1_Confidence_0.000_Length_1456	292.3	289
EOG09370ER5	Complete	Sample6_Locus_988_Transcript_4_5_Confidence_0.400_Length_993	202.6	202
EOG09370F1Q	Complete	SampleTS1_Locus_6795_Transcript_1_1_Confidence_0.000_Length_1133	96.7	119
EOG09370F3T	Complete	SampleTS1_Locus_7773_Transcript_2_2_Confidence_0.000_Length_881	57.8	176
EOG09370F47	Complete	Sample3_Locus_6459_Transcript_3_4_Confidence_0.200_Length_1533	441.4	268
EOG09370FKI	Complete	Sample1_Locus_10278_Transcript_1_3_Confidence_0.667_Length_1067	103.8	253
EOG09370FLD	Complete	SampleTS3_Locus_19797_Transcript_3_3_Confidence_0.000_Length_1329	395.7	316
EOG09370FN1	Complete	SampleTS3_Locus_1849_Transcript_3_5_Confidence_0.600_Length_1324	429.1	282
EOG09370FSS	Complete	Sample6_Locus_6392_Transcript_5_5_Confidence_0.000_Length_1715	306.6	354
EOG09370FVX	Complete	Sample1_Locus_9133_Transcript_2_8_Confidence_0.667_Length_1752	273.6	298
EOG09370FWQ	Complete	SampleTS3_Locus_13170_Transcript_1_1_Confidence_0.000_Length_1469	417.2	311
EOG09370FXE	Complete	Sample1_Locus_67_Transcript_9_10_Confidence_0.125_Length_1441	94.4	138
EOG09370GC2	Fragmented	Sample6_Locus_27805_Transcript_5_5_Confidence_0.000_Length_666	24.5	31
EOG09370GEO	Fragmented	Sample1_Locus_1116_Transcript_18_21_Confidence_0.181_Length_1365	115.1	138
EOG09370GHM	Complete	Sample1_Locus_10825_Transcript_2_3_Confidence_0.333_Length_2164	210.7	211
EOG09370GHP	Fragmented	Sample1_Locus_18114_Transcript_1_3_Confidence_0.333_Length_934	26.5	58
EOG09370GIK	Complete	Sample1_Locus_2710_Transcript_3_6_Confidence_0.429_Length_964	345.4	214
EOG09370GJ7	Complete	Sample3_Locus_13814_Transcript_2_3_Confidence_0.333_Length_1262	224.6	245
EOG09370GK0	Complete	SampleTS3_Locus_10370_Transcript_5_5_Confidence_0.000_Length_1459	198.1	245
EOG09370H3E	Complete	SampleTS1_Locus_4847_Transcript_3_5_Confidence_0.600_Length_1357	360	261
EOG09370H68	Complete	Sample1_Locus_12224_Transcript_1_1_Confidence_0.000_Length_1004	130.5	182
EOG09370HBN	Fragmented	Sample3_Locus_18667_Transcript_1_1_Confidence_0.000_Length_963	123.8	157
EOG09370HD7	Complete	Sample1_Locus_10854_Transcript_1_1_Confidence_0.000_Length_1327	330.6	264
EOG09370HKV	Complete	SampleTS2_Locus_504_Transcript_10_14_Confidence_0.368_Length_1174	115.6	305
EOG09370HVI	Missing			

EOG09370I3J	Complete	SampleTS2_Locus_4841_Transcript_5_10_Confidence_0.692_Length_1164	218.2	221
EOG09370I7R	Complete	Sample6_Locus_10203_Transcript_4_6_Confidence_0.333_Length_998	310.6	192
EOG09370IF5	Complete	Sample1_Locus_17656_Transcript_1_3_Confidence_0.333_Length_1400	324.2	284
EOG09370IH5	Complete	SampleTS1_Locus_4985_Transcript_4_22_Confidence_0.274_Length_1006	241.6	215
EOG09370IMU	Complete	Sample1_Locus_4533_Transcript_5_16_Confidence_0.090_Length_1635	164.2	193
EOG09370INZ	Fragmented	Sample6_Locus_19319_Transcript_1_1_Confidence_0.000_Length_447	46.9	70
EOG09370IUA	Complete	Sample1_Locus_24058_Transcript_2_3_Confidence_0.333_Length_1515	310.7	208
EOG09370J9L	Fragmented	SampleTS1_Locus_7629_Transcript_5_5_Confidence_0.000_Length_788	95.1	161
EOG09370JIK	Complete	Sample6_Locus_2590_Transcript_6_9_Confidence_0.500_Length_1456	219.2	196
EOG09370JW6	Missing			
EOG09370JZ3	Complete	Sample1_Locus_266_Transcript_9_9_Confidence_0.000_Length_1624	193.4	177
EOG09370K15	Complete	SampleTS3_Locus_24254_Transcript_1_1_Confidence_0.000_Length_902	70.9	123
EOG09370K1R	Fragmented	SampleTS2_Locus_3665_Transcript_1_1_Confidence_0.000_Length_1325	99.3	141
EOG09370KD4	Complete	Sample3_Locus_5266_Transcript_2_7_Confidence_0.300_Length_1183	384.9	234
EOG09370KGV	Complete	Sample6_Locus_15085_Transcript_1_1_Confidence_0.000_Length_1486	110.9	89
EOG09370KJ3	Complete	Sample1_Locus_8015_Transcript_1_2_Confidence_0.667_Length_962	222.4	177
EOG09370KM9	Complete	Sample3_Locus_10654_Transcript_5_7_Confidence_0.647_Length_1935	121.1	254
EOG09370KOV	Complete	Sample1_Locus_10314_Transcript_2_12_Confidence_0.069_Length_1259	156.3	179
EOG09370KWF	Complete	SampleTS2_Locus_8315_Transcript_1_1_Confidence_0.000_Length_1215	128.8	180
EOG09370KYZ	Complete	Sample6_Locus_19645_Transcript_2_4_Confidence_0.333_Length_1748	293.6	199
EOG09370L23	Complete	Sample1_Locus_7251_Transcript_6_6_Confidence_0.143_Length_1069	296.1	217
EOG09370L77	Missing			
EOG09370LFI	Complete	SampleTS2_Locus_5458_Transcript_8_9_Confidence_0.500_Length_1344	356.6	234
EOG09370LGH	Complete	Sample1_Locus_8_Transcript_6_11_Confidence_0.612_Length_1512	256.8	283
EOG09370LMP	Complete	Sample6_Locus_16309_Transcript_1_2_Confidence_0.750_Length_1716	288.4	301
EOG09370LQ2	Complete	Sample6_Locus_30085_Transcript_2_3_Confidence_0.500_Length_777	96.4	177
EOG09370LYY	Complete	SampleTS1_Locus_24122_Transcript_3_5_Confidence_0.500_Length_1074	112.2	278
EOG09370M9E	Complete	Sample3_Locus_27863_Transcript_1_1_Confidence_0.000_Length_907	290.1	204
EOG09370MAO	Complete	Sample3_Locus_23245_Transcript_2_3_Confidence_0.333_Length_1042	304.9	241

EOG09370MDU	Complete	SampleTS3_Locus_23743_Transcript_2_3_Confidence_0.500_Length_1152	209.4	183
EOG09370MGF	Complete	SampleTS2_Locus_2228_Transcript_13_14_Confidence_0.486_Length_2575	173.9	263
EOG09370MHI	Complete	Sample1_Locus_28199_Transcript_1_2_Confidence_0.750_Length_954	166.2	183
EOG09370MMK	Complete	SampleTS2_Locus_13332_Transcript_5_6_Confidence_0.222_Length_1565	350.8	233
EOG09370MQ0	Complete	SampleTS2_Locus_6327_Transcript_8_25_Confidence_0.174_Length_1156	252	205
EOG09370MZ4	Complete	Sample1_Locus_26978_Transcript_3_4_Confidence_0.400_Length_962	382.4	226
EOG09370N7W	Complete	Sample1_Locus_12672_Transcript_1_1_Confidence_0.000_Length_1866	398.5	267
EOG09370NBT	Complete	SampleTS2_Locus_9964_Transcript_2_4_Confidence_0.600_Length_1249	237.8	242
EOG09370NBW	Complete	SampleTS3_Locus_9895_Transcript_2_4_Confidence_0.400_Length_1636	122.1	153
EOG09370NHX	Fragmented	Sample3_Locus_11202_Transcript_3_3_Confidence_0.000_Length_613	56.8	96
EOG09370NIG	Complete	Sample1_Locus_11647_Transcript_4_6_Confidence_0.647_Length_3781	151.1	167
EOG09370O06	Complete	Sample1_Locus_8335_Transcript_5_6_Confidence_0.081_Length_1180	182.5	194
EOG09370O51	Complete	Sample1_Locus_7506_Transcript_1_4_Confidence_0.333_Length_951	254.5	196
EOG09370OFR	Complete	Sample3_Locus_19095_Transcript_1_2_Confidence_0.333_Length_1720	294.5	300
EOG09370OGU	Complete	Sample6_Locus_13202_Transcript_1_1_Confidence_0.000_Length_925	157.6	186
EOG09370OGW	Complete	Sample1_Locus_4270_Transcript_3_7_Confidence_0.333_Length_1214	125.2	144
EOG09370OHP	Complete	Sample6_Locus_15392_Transcript_2_4_Confidence_0.333_Length_1277	234.5	205
EOG09370OLY	Complete	SampleTS2_Locus_10705_Transcript_2_4_Confidence_0.400_Length_1376	163.7	149
EOG09370OO3	Complete	Sample1_Locus_11638_Transcript_3_4_Confidence_0.500_Length_1334	211	196
EOG09370OS5	Complete	SampleTS1_Locus_24318_Transcript_1_1_Confidence_0.000_Length_903	92.6	154
EOG09370OUS	Duplicated	Sample6_Locus_13813_Transcript_1_1_Confidence_0.000_Length_1092	229.3	168
EOG09370OUS	Duplicated	SampleTS1_Locus_6359_Transcript_7_7_Confidence_0.294_Length_1274	198.5	173
EOG09370P2W	Duplicated	Sample6_Locus_10877_Transcript_1_3_Confidence_0.667_Length_556	82.1	94
EOG09370P2W	Duplicated	SampleTS2_Locus_1141_Transcript_1_3_Confidence_0.667_Length_647	80.5	94
EOG09370P5Q	Fragmented	Sample6_Locus_12518_Transcript_3_3_Confidence_0.200_Length_959	115.9	139
EOG09370P7I	Complete	Sample6_Locus_23783_Transcript_3_3_Confidence_0.000_Length_864	105	181
EOG09370P9T	Fragmented	Sample3_Locus_17766_Transcript_1_2_Confidence_0.333_Length_694	194.1	157
EOG09370PBD	Fragmented	SampleTS3_Locus_22996_Transcript_4_6_Confidence_0.400_Length_583	264.8	157

EOG09370PLC	Complete	Sample6_Locus_1702_Transcript_13_18_Confidence_0.348_Length_1386	249.5	223
EOG09370PN3	Missing			
EOG09370PYJ	Complete	SampleTS3_Locus_11332_Transcript_1_1_Confidence_0.000_Length_760	282	224
EOG09370QBK	Complete	SampleTS3_Locus_6723_Transcript_6_8_Confidence_0.286_Length_1079	131	138
EOG09370QBR	Fragmented	SampleTS1_Locus_5209_Transcript_1_1_Confidence_0.750_Length_741	145.5	142
EOG09370QCX	Missing			
EOG09370QFD	Complete	SampleTS2_Locus_6656_Transcript_5_10_Confidence_0.565_Length_1070	79.4	154
EOG09370QI4	Complete	SampleTS1_Locus_3459_Transcript_2_4_Confidence_0.714_Length_1228	158.1	189
EOG09370QJ1	Complete	Sample1_Locus_29286_Transcript_1_3_Confidence_0.333_Length_1031	257.1	176
EOG09370QKS	Missing			
EOG09370QMM	Complete	Sample6_Locus_536_Transcript_5_7_Confidence_0.071_Length_1972	177	210
EOG09370QT3	Complete	SampleTS2_Locus_13041_Transcript_7_8_Confidence_0.100_Length_1217	42.4	80
EOG09370QVI	Complete	SampleTS2_Locus_15990_Transcript_2_4_Confidence_0.750_Length_1485	208.6	220
EOG09370R3M	Complete	SampleTS2_Locus_8132_Transcript_2_2_Confidence_0.333_Length_829	268.7	183
EOG09370R67	Complete	Sample3_Locus_10859_Transcript_1_1_Confidence_0.000_Length_749	130.2	107
EOG09370RCD	Complete	Sample1_Locus_14437_Transcript_6_7_Confidence_0.333_Length_2011	292.5	408
EOG09370RCQ	Complete	SampleTS2_Locus_4969_Transcript_19_24_Confidence_0.235_Length_980	270.6	187
EOG09370RCS	Complete	Sample1_Locus_13869_Transcript_1_13_Confidence_0.059_Length_1475	195.8	170
EOG09370RIT	Complete	Sample1_Locus_5002_Transcript_2_3_Confidence_0.333_Length_1136	266.7	134
EOG09370RJN	Complete	Sample3_Locus_13187_Transcript_7_8_Confidence_0.545_Length_1733	302.6	271
EOG09370RRJ	Fragmented	SampleTS1_Locus_6265_Transcript_1_4_Confidence_0.200_Length_1674	164.2	94
EOG09370S0F	Fragmented	Sample6_Locus_24708_Transcript_6_11_Confidence_0.068_Length_327	81.2	65
EOG09370S1Z	Complete	Sample6_Locus_15436_Transcript_1_3_Confidence_0.333_Length_1435	88.8	78
EOG09370S3E	Complete	Sample1_Locus_20404_Transcript_1_2_Confidence_0.333_Length_1142	112.9	156
EOG09370S4T	Complete	Sample3_Locus_10702_Transcript_8_9_Confidence_0.222_Length_709	180.4	131
EOG09370SC9	Complete	Sample1_Locus_20787_Transcript_1_1_Confidence_0.000_Length_670	92.9	122
EOG09370SO4	Complete	Sample6_Locus_25078_Transcript_2_3_Confidence_0.667_Length_1039	49.2	79
EOG09370SON	Fragmented	SampleTS2_Locus_22510_Transcript_4_4_Confidence_0.000_Length_765	132.6	116
EOG09370SPQ	Missing			
EOG09370SV2	Complete	SampleTS1_Locus_22960_Transcript_4_5_Confidence_0.545_Length_1144	150.1	136

EOG09370SYK	Complete	Sample6_Locus_10778_Transcript_11_17_Confidence_0.136_Length_1868	380.5	389
EOG09370T6W	Complete	Sample3_Locus_7656_Transcript_3_3_Confidence_0.000_Length_792	168.1	166
EOG09370T9R	Complete	Sample1_Locus_3476_Transcript_2_10_Confidence_0.077_Length_973	213.5	157
EOG09370TBD	Complete	Sample3_Locus_6085_Transcript_14_15_Confidence_0.065_Length_696	210.6	136
EOG09370TBG	Complete	Sample1_Locus_8940_Transcript_3_4_Confidence_0.400_Length_1113	125.2	154
EOG09370TCX	Complete	Sample1_Locus_25419_Transcript_1_1_Confidence_0.000_Length_509	185.9	136
EOG09370TFO	Duplicated	Sample1_Locus_25057_Transcript_2_6_Confidence_0.727_Length_1459	90.5	234
EOG09370TFO	Duplicated	Sample6_Locus_7235_Transcript_2_4_Confidence_0.500_Length_949	82	190
EOG09370TG2	Complete	SampleTS3_Locus_5573_Transcript_4_5_Confidence_0.333_Length_940	180.6	149
EOG09370TG6	Complete	SampleTS1_Locus_9604_Transcript_1_1_Confidence_0.667_Length_1348	150.7	133
EOG09370TH4	Complete	Sample6_Locus_661_Transcript_11_15_Confidence_0.057_Length_877	142.6	119
EOG09370TP2	Complete	SampleTS1_Locus_7011_Transcript_1_1_Confidence_0.000_Length_1056	202.5	222
EOG09370TS7	Complete	SampleTS2_Locus_12167_Transcript_1_1_Confidence_0.000_Length_1077	119	131
EOG09370TTI	Complete	SampleTS2_Locus_14345_Transcript_1_1_Confidence_0.000_Length_568	79.4	83
EOG09370U0A	Complete	Sample1_Locus_2474_Transcript_1_1_Confidence_0.000_Length_652	195.3	160
EOG09370U71	Complete	Sample1_Locus_3449_Transcript_9_11_Confidence_0.667_Length_1316	198.1	208
EOG09370U9P	Complete	Sample3_Locus_9475_Transcript_2_2_Confidence_0.000_Length_1080	97.7	164
EOG09370UEC	Complete	SampleTS1_Locus_24176_Transcript_5_5_Confidence_0.200_Length_1263	219.7	161
EOG09370UG9	Duplicated	Sample3_Locus_9971_Transcript_6_8_Confidence_0.385_Length_1981	138.2	145
EOG09370UG9	Duplicated	Sample3_Locus_9971_Transcript_8_8_Confidence_0.077_Length_1449	139.2	145
EOG09370UG9	Duplicated	SampleTS2_Locus_11308_Transcript_6_6_Confidence_0.000_Length_879	140.5	146
EOG09370UID	Complete	Sample3_Locus_26583_Transcript_4_6_Confidence_0.556_Length_1236	229.4	130
EOG09370UO4	Complete	Sample3_Locus_6816_Transcript_2_4_Confidence_0.750_Length_1043	205.1	165
EOG09370UR1	Complete	Sample1_Locus_13147_Transcript_1_2_Confidence_0.333_Length_892	141.9	137
EOG09370UR3	Complete	Sample1_Locus_15932_Transcript_1_1_Confidence_0.000_Length_833	229.6	159
EOG09370USH	Complete	Sample3_Locus_2005_Transcript_1_1_Confidence_0.000_Length_978	127.4	146
EOG09370USJ	Complete	Sample6_Locus_8749_Transcript_2_2_Confidence_0.500_Length_914	235.6	183
EOG09370UV4	Fragmented	SampleTS3_Locus_23306_Transcript_1_3_Confidence_0.750_Length_600	141.7	122

EOG09370V9R	Complete	SampleTS1_Locus_18781_Transcript_2_4_Confidence_0.400_Length_1162	66.7	116
EOG09370VFL	Complete	SampleTS3_Locus_4545_Transcript_3_4_Confidence_0.200_Length_1244	214.5	247
EOG09370VLM	Complete	SampleTS1_Locus_8576_Transcript_2_2_Confidence_0.000_Length_656	103.9	112
EOG09370VTP	Fragmented	Sample6_Locus_5591_Transcript_3_6_Confidence_0.000_Length_305	59.8	75
EOG09370VUX	Complete	Sample6_Locus_19866_Transcript_3_3_Confidence_0.000_Length_873	147.3	142
EOG09370W44	Complete	SampleTS2_Locus_1415_Transcript_4_7_Confidence_0.571_Length_968	151.4	125
EOG09370W6N	Complete	Sample3_Locus_2360_Transcript_1_4_Confidence_0.400_Length_677	139.4	138
EOG09370W86	Complete	SampleTS1_Locus_7077_Transcript_1_4_Confidence_0.400_Length_1107	206.2	160
EOG09370WT1	Complete	SampleTS3_Locus_7825_Transcript_2_2_Confidence_0.000_Length_708	146.9	123
EOG09370WWI	Complete	Sample3_Locus_25812_Transcript_3_5_Confidence_0.125_Length_983	147.8	157
EOG09370WYG	Missing			
EOG09370WZX	Complete	SampleTS2_Locus_11551_Transcript_1_1_Confidence_0.000_Length_999	86	149
EOG09370X1T	Complete	Sample6_Locus_19376_Transcript_2_3_Confidence_0.750_Length_511	107.4	96
EOG09370XFG	Complete	SampleTS1_Locus_28942_Transcript_1_2_Confidence_0.333_Length_1054	59.7	107
EOG09370XFJ	Complete	Sample3_Locus_1220_Transcript_6_8_Confidence_0.429_Length_1154	364.8	284
EOG09370XO6	Complete	Sample1_Locus_12634_Transcript_5_6_Confidence_0.167_Length_799	168.1	129
EOG09370Y63	Missing			
EOG09370YC4	Complete	SampleTS3_Locus_9956_Transcript_3_3_Confidence_0.250_Length_794	111.7	159
EOG09370YCN	Complete	Sample1_Locus_9670_Transcript_4_4_Confidence_0.000_Length_614	109.2	115
EOG09370YSI	Complete	Sample3_Locus_9561_Transcript_4_6_Confidence_0.061_Length_1614	187.9	234
EOG09370ZI7	Complete	SampleTS1_Locus_20615_Transcript_2_2_Confidence_0.000_Length_447	91.7	85
EOG09370ZOK	Complete	Sample6_Locus_9715_Transcript_7_10_Confidence_0.312_Length_2211	111.7	289
EOG0937106D	Complete	Sample6_Locus_8723_Transcript_4_5_Confidence_0.200_Length_746	151.9	138
EOG0937106Y	Complete	Sample3_Locus_24653_Transcript_1_2_Confidence_0.500_Length_644	147.6	155
EOG09371088	Complete	Sample3_Locus_10333_Transcript_2_3_Confidence_0.750_Length_926	175.2	140
EOG093710A7	Complete	SampleTS3_Locus_8191_Transcript_4_6_Confidence_0.429_Length_770	63.7	84
EOG093710JH	Complete	Sample1_Locus_24940_Transcript_2_3_Confidence_0.051_Length_716	58.9	72
EOG093710SY	Complete	Sample1_Locus_5469_Transcript_3_3_Confidence_0.000_Length_653	164.9	103
EOG093710T1	Complete	SampleTS1_Locus_6585_Transcript_2_9_Confidence_0.154_Length_824	64.7	85

EOG0937112Y	Complete	Sample6_Locus_13893_Transcript_3_3_Confidence_0.167_Length_1014	140.6	189
EOG093711E9	Complete	Sample1_Locus_3284_Transcript_3_5_Confidence_0.429_Length_669	162.8	110
EOG093711LC	Complete	SampleTS3_Locus_23452_Transcript_2_2_Confidence_0.333_Length_474	69.9	70
EOG093711QY	Fragmented	Sample3_Locus_10114_Transcript_3_3_Confidence_0.000_Length_660	54	54
EOG093711ZO	Complete	Sample1_Locus_24571_Transcript_8_9_Confidence_0.300_Length_768	214.5	170
EOG0937122Q	Duplicated	Sample3_Locus_1719_Transcript_2_5_Confidence_0.571_Length_545	52.9	71
EOG0937122Q	Duplicated	Sample6_Locus_3576_Transcript_6_7_Confidence_0.500_Length_457	45.7	69
EOG0937128O	Fragmented	SampleTS3_Locus_23143_Transcript_1_1_Confidence_0.000_Length_593	81.5	68
EOG0937129K	Complete	Sample1_Locus_8283_Transcript_16_27_Confidence_0.476_Length_2456	95.8	84
EOG093712G8	Fragmented	SampleTS3_Locus_3197_Transcript_1_1_Confidence_0.000_Length_435	93.8	91
EOG093712Q6	Complete	SampleTS2_Locus_11321_Transcript_4_4_Confidence_0.286_Length_668	149.2	72
EOG09371330	Complete	Sample6_Locus_7693_Transcript_9_11_Confidence_0.000_Length_836	107.6	112
EOG0937133J	Complete	Sample6_Locus_4538_Transcript_2_2_Confidence_0.000_Length_588	187.6	111
EOG093713HJ	Complete	Sample1_Locus_2642_Transcript_3_7_Confidence_0.500_Length_846	91.9	84
EOG093713LY	Complete	SampleTS1_Locus_30893_Transcript_1_1_Confidence_0.000_Length_566	130.1	87
EOG09371431	Complete	Sample1_Locus_1163_Transcript_2_3_Confidence_0.500_Length_574	164.2	97
EOG093714JU	Complete	SampleTS2_Locus_9976_Transcript_8_9_Confidence_0.636_Length_459	77.6	82
EOG093714Q2	Missing			
EOG093715EC	Complete	Sample1_Locus_3035_Transcript_1_1_Confidence_0.000_Length_693	92.5	90
EOG09371675	Complete	SampleTS3_Locus_24383_Transcript_1_1_Confidence_0.000_Length_439	122.9	93
EOG0937167R	Complete	Sample6_Locus_613_Transcript_4_5_Confidence_0.400_Length_739	122.6	103
EOG093716T7	Complete	SampleTS2_Locus_7013_Transcript_4_6_Confidence_0.667_Length_1221	155.3	217
EOG093717LU	Complete	SampleTS2_Locus_9380_Transcript_4_5_Confidence_0.429_Length_552	95.8	51
EOG0937183G	Missing			
EOG0937186Q	Complete	Sample1_Locus_21626_Transcript_3_4_Confidence_0.500_Length_1658	108	106
EOG093718E9	Complete	Sample1_Locus_29387_Transcript_1_1_Confidence_0.000_Length_514	109.1	77
EOG093718EG	Missing			
EOG0937192A	Complete	SampleTS1_Locus_13686_Transcript_1_4_Confidence_0.167_Length_930	110.3	132
EOG093719IK	Complete	SampleTS1_Locus_23455_Transcript_1_1_Confidence_0.000_Length_893	117	101

EOG093719M8	Complete	Sample6_Locus_4514_Transcript_2_6_Confidence_0.400_Length_1026	56.5	66
EOG09371BIR	Complete	SampleTS2_Locus_4839_Transcript_2_2_Confidence_0.333_Length_642	74.2	95

Table S4. List of KEGG pathways associated to contigs with assigned enzyme codes in differentially regulated genes. The table contains pathway names, pathway ID, number of enzymes found for each pathway, enzyme codes and sequence names.

Pathway	Pathway ID	#Enzs in Pathway	Enzyme	#Seqs of Enzyme	Seqs
**Biosynthesis of antibiotics**	map01130	112	ec:2.6.1.11 transaminase, ec:6.3.3.1 cycloligase, ec:2.6.1.13 aminotransferase, ec:6.4.1.2 carboxylase, ec:2.1.2.2 formyltransferase, ec:2.6.1.16 transaminase (isomerizing), ec:2.1.2.1 hydroxymethyltransferase, ec:1.14.13.39 synthase (NADPH), ec:6.4.1.3 carboxylase, ec:2.3.1.61 succinyltransferase, ec:4.1.2.13 aldolase, ec:2.7.4.6 kinase, ec:2.7.4.3 kinase, ec:4.1.1.49 carboxykinase (ATP), ec:3.5.2.6 penicillinase, ec:2.3.1.94 synthase, ec:4.1.1.39 carboxylase, ec:1.3.5.1 dehydrogenase, ec:2.7.1.71 kinase, ec:4.1.1.32 carboxykinase (GTP), ec:4.2.1.9 dehydratase, ec:4.2.1.3 hydratase, ec:4.2.1.2 hydratase, ec:1.17.7.1 synthase (ferredoxin), ec:2.6.1.52 transaminase, ec:2.6.1.57 transaminase, ec:2.6.1.58 transaminase, ec:2.2.1.1	1, 2, 1, 2, 1, 1, 2, 1, 2, 1, 2, 5, 7, 1, 1, 2, 1, 1, 1, 1, 1, 1, 2, 2, 1, 2, 1, 1, 3, 1, 1, 1, 7, 2, 1, 1, 3, 1, 1, 1, 2, 2, 1, 3, 1, 1, 1, 2, 2, 1, 1, 1, 1, 2, 2, 1, 1, 3, 2, 5, 5, 2, 1, 2, 1, 1, 1, 1, 1, 2, 2, 1, 5, 1, 10, 5, 3, 2, 2, 3, 3, 3, 1, 1, 1, 2, 2, 1, 2, 1, 1, 6, 1, 2, 1, 3, 2, 2, 2, 1, 1, 2, 1, 1, 1, 1, 3, 1, 2, 2, 1, 1	SampleTS1_Locus_13173_Transcript_7/8_Confidence_0.190_Length_747, SampleTS1_Locus_2676_Transcript_23/50_Confidence_0.046_Length_813, Sample3_Locus_8866_Transcript_2/4_Confidence_0.714_Length_1157, SampleTS3_Locus_10707_Transcript_4/7_Confidence_0.500_Length_1675, Sample3_Locus_8920_Transcript_2/3_Confidence_0.333_Length_2038, SampleTS1_Locus_1155_Transcript_11/16_Confidence_0.625_Length_2240, SampleTS2_Locus_12481_Transcript_10/11_Confidence_0.194_Length_1103, Sample6_Locus_5350_Transcript_7/9_Confidence_0.333_Length_2351, Sample1_Locus_3932_Transcript_7/11_Confidence_0.400_Length_1725, SampleTS1_Locus_10618_Transcript_3/4_Confidence_0.400_Length_1517, Sample1_Locus_25642_Transcript_6/7_Confidence_0.500_Length_1752, SampleTS1_Locus_1968_Transcript_1/3_Confidence_0.400_Length_530, SampleTS1_Locus_1155_Transcript_11/16_Confidence_0.625_Length_2240, Sample3_Locus_4770_Transcript_12/16_Confidence_0.420_Length_1753, Sample3_Locus_324_Transcript_3/17_Confidence_0.222_Length_1711, Sample6_Locus_28252_Transcript_2/3_Confidence_0.500_Length_808, SampleTS1_Locus_4995_Transcript_3/15_Confidence_0.395_Length_904, SampleTS1_Locus_6152_Transcript_2/7_Confidence_0.375_Length_1167, SampleTS1_Locus_1762_Transcript_13/36_Confidence_0.058_Length_819, Sample1_Locus_8568_Transcript_27/30_Confidence_0.054_Length_2057, Sample1_Locus_24078_Transcript_6/7_Confidence_0.150_Length_1572, Sample3_Locus_10333_Transcript_2/3_Confidence_0.750_Length_926, Sample3_Locus_3294_Transcript_4/6_Confidence_0.429_Length_2049, SampleTS1_Locus_6152_Transcript_2/7_Confidence_0.375_Length_1167, Sample3_Locus_9766_Transcript_1/1_Confidence_0.000_Length_1440, Sample3_Locus_6601_Transcript_5/5_Confidence_0.200_Length_4716, SampleTS1_Locus_1762_Transcript_13/36_Confidence_0.058_Length_819, Sample1_Locus_24078_Transcript_6/7_Confidence_0.150_Length_1572, Sample6_Locus_11174_Transcript_1/1_Confidence_0.000_Length_1998, SampleTS3_Locus_23179_Transcript_2/6_Confidence_0.500_Length_1144, Sample3_Locus_4157_Transcript_2/4_Confidence_0.333_Length_2315, Sample6_Locus_8953_Transcript_13/14_Confidence_0.143_Length_4871, SampleTS1_Locus_4324_Transcript_3/4_Confidence_0.286_Length_1536, SampleTS2_Locus_54_Transcript_4/10_Confidence_0.083_Length_1374, SampleTS2_Locus_1705_Transcript_2/5_Confidence_0.667_Length_1103, Sample6_Locus_11174_Transcript_1/1_Confidence_0.000_Length_1998, Sample1_Locus_2272_Transcript_43/67_Confidence_0.374_Length_2859, SampleTS2_Locus_7424_Transcript_6/8_Confidence_0.286_Length_3139, Sample3_Locus_2252_Transcript_9/13_Confidence_0.647_Length_1708, Sample6_Locus_1413_Transcript_3/3_Confidence_0.000_Length_1802, Sample6_Locus_5736_Transcript_2/4_Confidence_0.400_Length_1660, SampleTS1_Locus_12312_Transcript_2/4_Confidence_0.333_Length_2330, Sample1_Locus_9264_Transcript_1/1_Confidence_0.000_Length_1678, Sample1_Locus_26802_Transcript_1/2_Confidence_0.667_Length_439, Sample6_Locus_13664_Transcript_1/2_Confidence_0.333_Length_862, Sample6_Locus_22798_Transcript_3/3_Confidence_0.250_Length_666, SampleTS1_Locus_16129_Transcript_1/1_Confidence_0.000_Length_1960, SampleTS2_Locus_11692_Transcript_2/7_Confidence_0.333_Length_2130, SampleTS2_Locus_760_Transcript_7/10_Confidence_0.186_Length_1031, Sample6_Locus_13592_Transcript_5/5_Confidence_0.250_Length_879, Sample3_Locus_4596_Transcript_3/6_Confidence_0.400_Length_1157, Sample1_Locus_5305_Transcript_3/12_Confidence_0.143_Length_2221, Sample3_Locus_2031_Transcript_1/1_Confidence_0.000_Length_2262, Sample6_Locus_12247_Transcript_7/9_Confidence_0.429_Length_1396, SampleTS1_Locus_11723_Transcript_6/16_Confidence_0.073_Length_167

			glycolaldehydet<br>ransferase,<br>ec:1.11.1.6<br>equilase,<br>ec:2.2.1.6<br>synthase,<br>ec:4.1.3.27<br>synthase,<br>ec:2.7.2.4<br>kinase,<br>ec:2.7.1.40<br>kinase,<br>ec:2.7.2.11<br>5kinase,<br>ec:3.5.4.6<br>deaminase,<br>ec:6.3.2.6<br>synthase,<br>ec:1.2.1.41<br>dehydrogenase,<br>ec:2.7.1.2<br>glucokinase<br>(phosphorylatin<br>g), ec:2.7.1.1<br>hexokinase type<br>IV glucokinase,<br>ec:4.2.3.5<br>synthase,<br>ec:4.2.3.4<br>synthase,<br>ec:2.6.1.42<br>transaminase,<br>ec:2.6.1.45<br>transaminase,<br>ec:1.2.1.59<br>dehydrogenase<br>(NAD(P)+)<br>(phosphorylatin<br>g), ec:1.1.1.216<br>dehydrogenase<br>(NADP+),<br>ec:4.1.1.1<br>decarboxylase,<br>ec:4.2.1.51<br>dehydratase,<br>ec:6.2.1.5<br>ligase<br>(ADPforming),<br>ec:6.2.1.4<br>ligase<br>(GDPforming),<br>ec:6.2.1.1<br>ligase,<br>ec:5.1.3.3<br>1epimerase,<br>ec:5.1.3.1<br>3epimerase,<br>ec:2.5.1.21<br>synthase,<br>ec:2.3.1.12<br>acetyltransferas<br>e, ec:3.1.3.11<br>hexose<br>diphosphatase,<br>ec:2.3.1.16<br>Cacyltransferas<br>e, ec:2.5.1.29<br>diphosphate<br>synthase,<br>ec:2.7.7.4<br>adenylyltransfe<br>rase,<br>ec:1.1.1.133		9,<br>SampleTS1_Locus_7761_Transcript_5/10_Confidence_0.105_Length_1358<br>,<br>SampleTS2_Locus_14711_Transcript_4/7_Confidence_0.273_Length_1393<br>,<br>Sample6_Locus_2327_Transcript_47/56_Confidence_0.195_Length_2423,<br>Sample6_Locus_22851_Transcript_2/3_Confidence_0.714_Length_1077,<br>SampleTS1_Locus_2696_Transcript_4/6_Confidence_0.545_Length_1155,<br>Sample3_Locus_23456_Transcript_1/1_Confidence_0.000_Length_1406,<br>SampleTS2_Locus_20780_Transcript_2/3_Confidence_0.600_Length_1460<br>, Sample1_Locus_7394_Transcript_5/5_Confidence_0.000_Length_2434,<br>SampleTS2_Locus_20841_Transcript_1/1_Confidence_0.000_Length_1375<br>, Sample3_Locus_23456_Transcript_1/1_Confidence_0.000_Length_1406,<br>Sample1_Locus_3087_Transcript_2/3_Confidence_0.333_Length_2354,<br>SampleTS2_Locus_20780_Transcript_2/3_Confidence_0.600_Length_1460<br>, Sample6_Locus_5095_Transcript_5/8_Confidence_0.300_Length_1241,<br>Sample6_Locus_5095_Transcript_5/8_Confidence_0.300_Length_1241,<br>SampleTS2_Locus_10844_Transcript_3/4_Confidence_0.200_Length_1482<br>, Sample6_Locus_15905_Transcript_2/2_Confidence_0.000_Length_483,<br>Sample3_Locus_8753_Transcript_3/7_Confidence_0.053_Length_1045,<br>Sample1_Locus_18777_Transcript_2/4_Confidence_0.923_Length_1416,<br>Sample3_Locus_5952_Transcript_4/4_Confidence_0.000_Length_2586,<br>Sample6_Locus_15604_Transcript_2/4_Confidence_0.400_Length_1416,<br>SampleTS2_Locus_819_Transcript_16/18_Confidence_0.250_Length_1000<br>,<br>SampleTS2_Locus_12618_Transcript_4/6_Confidence_0.571_Length_1272<br>, Sample6_Locus_997_Transcript_9/17_Confidence_0.200_Length_950,<br>SampleTS1_Locus_13725_Transcript_2/7_Confidence_0.333_Length_1080<br>, Sample3_Locus_4596_Transcript_3/6_Confidence_0.400_Length_1157,<br>Sample6_Locus_164_Transcript_78/296_Confidence_0.027_Length_1952,<br>Sample3_Locus_2152_Transcript_2/3_Confidence_0.333_Length_1481,<br>SampleTS3_Locus_6340_Transcript_1/1_Confidence_0.000_Length_1077,<br>Sample3_Locus_2152_Transcript_2/3_Confidence_0.333_Length_1481,<br>SampleTS3_Locus_6340_Transcript_1/1_Confidence_0.000_Length_1077,<br>SampleTS3_Locus_456_Transcript_2/6_Confidence_0.200_Length_2075,<br>Sample3_Locus_10443_Transcript_10/11_Confidence_0.700_Length_2690<br>, Sample3_Locus_3334_Transcript_1/5_Confidence_0.400_Length_1030,<br>Sample6_Locus_901_Transcript_6/8_Confidence_0.643_Length_1628,<br>SampleTS2_Locus_8836_Transcript_1/2_Confidence_0.667_Length_1729,<br>SampleTS3_Locus_5316_Transcript_1/2_Confidence_0.750_Length_1843,<br>Sample3_Locus_10262_Transcript_4/5_Confidence_0.062_Length_1337,<br>Sample6_Locus_2283_Transcript_7/17_Confidence_0.225_Length_1818,<br>SampleTS2_Locus_1750_Transcript_17/20_Confidence_0.182_Length_156<br>8, Sample6_Locus_901_Transcript_6/8_Confidence_0.643_Length_1628,<br>SampleTS3_Locus_4546_Transcript_3/8_Confidence_0.429_Length_1895,<br>SampleTS3_Locus_3403_Transcript_3/4_Confidence_0.333_Length_1292,<br>SampleTS1_Locus_25259_Transcript_1/2_Confidence_0.333_Length_1099<br>, Sample3_Locus_2806_Transcript_6/14_Confidence_0.167_Length_1197,<br>SampleTS2_Locus_1881_Transcript_15/19_Confidence_0.439_Length_912<br>,<br>SampleTS3_Locus_2330_Transcript_4/4_Confidence_0.000_Length_1686,<br>SampleTS1_Locus_16292_Transcript_4/5_Confidence_0.600_Length_1341<br>, Sample3_Locus_4208_Transcript_8/15_Confidence_0.091_Length_2117,<br>Sample3_Locus_40_Transcript_1/1_Confidence_0.000_Length_1702,<br>Sample3_Locus_14770_Transcript_1/1_Confidence_0.000_Length_1819,<br>SampleTS2_Locus_819_Transcript_16/18_Confidence_0.250_Length_1000<br>,<br>Sample1_Locus_7420_Transcript_10/14_Confidence_0.154_Length_1346,<br>SampleTS2_Locus_12618_Transcript_4/6_Confidence_0.571_Length_1272<br>, Sample6_Locus_997_Transcript_9/17_Confidence_0.200_Length_950,<br>Sample1_Locus_393_Transcript_6/7_Confidence_0.556_Length_1466,<br>Sample6_Locus_7028_Transcript_1/3_Confidence_0.750_Length_1269,<br>SampleTS1_Locus_23304_Transcript_2/3_Confidence_0.333_Length_2324<br>, Sample3_Locus_6377_Transcript_4/4_Confidence_0.125_Length_1830,<br>Sample3_Locus_17004_Transcript_3/5_Confidence_0.286_Length_574,<br>Sample3_Locus_3732_Transcript_3/7_Confidence_0.545_Length_970,<br>Sample6_Locus_16912_Transcript_4/6_Confidence_0.286_Length_1521,<br>Sample6_Locus_164_Transcript_78/296_Confidence_0.027_Length_1952,<br>Sample6_Locus_81_Transcript_6/10_Confidence_0.421_Length_1354,<br>Sample6_Locus_3108_Transcript_12/12_Confidence_0.019_Length_1297,<br>SampleTS3_Locus_4699_Transcript_6/10_Confidence_0.579_Length_1963<br>,<br>SampleTS2_Locus_13350_Transcript_1/3_Confidence_0.778_Length_1614

			reductase, ec:2.7.7.9 uridylyltransferase, ec:1.2.1.12 dehydrogenase (phosphorylating), ec:4.1.1.17 decarboxylase, ec:2.3.1.30 Oacetyltransferase, ec:2.3.1.35 Nacetyltransferase, ec:2.7.1.148 5'diphospho)2CmethylDerythritol kinase, ec:4.2.1.91 dehydratase, ec:2.7.6.1 diphosphokinase, ec:1.17.1.8 reductase, ec:1.8.1.4 dehydrogenase, ec:2.5.1.1 geranyldiphosphate synthase, ec:5.3.1.6 isomerase, ec:5.3.1.9 isomerase, ec:2.7.7.64 uridylyltransferase, ec:3.1.3.3 phosphatase, ec:1.1.1.2 dehydrogenase (NADP+), ec:1.1.1.1 dehydrogenase, ec:4.2.1.20 synthase, ec:2.6.1.5 transaminase, ec:2.6.1.9 transaminase, ec:2.5.1.47 synthase, ec:2.6.1.1 transaminase, ec:2.6.1.4 transaminase, ec:4.2.1.11 hydratase, ec:5.3.3.2 Deltaisomerase, ec:2.5.1.54 synthase, ec:2.5.1.58 farnesyltransferase, ec:4.2.1.17 hydratase, ec:2.7.7.23 diphosphorylase, ec:1.4.1.14 synthase (NADH), ec:1.1.3.15 oxidase, ec:1.1.1.86 reductoisomerase (NADP+),		, SampleTS3_Locus_7085_Transcript_3/3_Confidence_0.000_Length_1349, SampleTS1_Locus_27877_Transcript_2/3_Confidence_0.333_Length_987, Sample3_Locus_9475_Transcript_2/2_Confidence_0.000_Length_1080, SampleTS1_Locus_12696_Transcript_3/3_Confidence_0.000_Length_1743, SampleTS3_Locus_2330_Transcript_4/4_Confidence_0.000_Length_1686, SampleTS1_Locus_16292_Transcript_4/5_Confidence_0.600_Length_1341, Sample3_Locus_4208_Transcript_8/15_Confidence_0.091_Length_2117, Sample3_Locus_40_Transcript_1/1_Confidence_0.000_Length_1702, Sample3_Locus_14770_Transcript_1/1_Confidence_0.000_Length_1819, Sample1_Locus_18291_Transcript_1/1_Confidence_0.000_Length_647, Sample6_Locus_15547_Transcript_5/8_Confidence_0.545_Length_1332, Sample6_Locus_7369_Transcript_3/6_Confidence_0.200_Length_1828, SampleTS1_Locus_2734_Transcript_1/3_Confidence_0.333_Length_2491, SampleTS2_Locus_15764_Transcript_1/1_Confidence_0.000_Length_1068, Sample3_Locus_27117_Transcript_1/3_Confidence_0.250_Length_685, Sample6_Locus_6954_Transcript_4/5_Confidence_0.500_Length_1201, Sample6_Locus_4129_Transcript_8/27_Confidence_0.107_Length_571, SampleTS3_Locus_8149_Transcript_4/10_Confidence_0.118_Length_927, SampleTS1_Locus_6731_Transcript_14/15_Confidence_0.246_Length_2303, SampleTS1_Locus_10087_Transcript_6/7_Confidence_0.167_Length_948, SampleTS2_Locus_4874_Transcript_6/12_Confidence_0.158_Length_1042, Sample6_Locus_1524_Transcript_26/30_Confidence_0.185_Length_3215, SampleTS2_Locus_263_Transcript_1/3_Confidence_0.250_Length_2184, SampleTS3_Locus_400_Transcript_6/29_Confidence_0.422_Length_2479, SampleTS3_Locus_23264_Transcript_1/3_Confidence_0.500_Length_845, SampleTS3_Locus_9447_Transcript_3/3_Confidence_0.000_Length_886, Sample6_Locus_6882_Transcript_6/8_Confidence_0.429_Length_1475, SampleTS1_Locus_10894_Transcript_1/1_Confidence_0.000_Length_652, Sample1_Locus_26802_Transcript_1/2_Confidence_0.667_Length_439, Sample6_Locus_13664_Transcript_1/2_Confidence_0.333_Length_862, Sample1_Locus_26802_Transcript_1/2_Confidence_0.667_Length_439, Sample6_Locus_13664_Transcript_1/2_Confidence_0.333_Length_862, Sample1_Locus_15681_Transcript_1/1_Confidence_0.000_Length_1030, Sample6_Locus_5355_Transcript_5/8_Confidence_0.577_Length_1830, SampleTS2_Locus_2923_Transcript_3/4_Confidence_0.333_Length_1277, Sample1_Locus_26802_Transcript_1/2_Confidence_0.667_Length_439, Sample6_Locus_13664_Transcript_1/2_Confidence_0.333_Length_862, SampleTS2_Locus_6646_Transcript_2/2_Confidence_0.333_Length_1520, Sample6_Locus_2195_Transcript_14/16_Confidence_0.176_Length_1741, SampleTS3_Locus_714_Transcript_7/14_Confidence_0.087_Length_1194, Sample1_Locus_19449_Transcript_1/1_Confidence_0.000_Length_1704, SampleTS1_Locus_3608_Transcript_5/7_Confidence_0.222_Length_1514, Sample6_Locus_5354_Transcript_1/7_Confidence_0.333_Length_1260, Sample3_Locus_8990_Transcript_7/9_Confidence_0.091_Length_1811, SampleTS1_Locus_610_Transcript_52/54_Confidence_0.210_Length_1630, SampleTS2_Locus_2631_Transcript_10/37_Confidence_0.203_Length_869, SampleTS1_Locus_789_Transcript_9/11_Confidence_0.238_Length_2300, Sample3_Locus_5282_Transcript_2/7_Confidence_0.700_Length_1266, Sample3_Locus_4208_Transcript_8/15_Confidence_0.091_Length_2117, SampleTS3_Locus_21707_Transcript_1/2_Confidence_0.333_Length_2460, SampleTS1_Locus_1310_Transcript_14/17_Confidence_0.222_Length_1901, Sample3_Locus_1427_Transcript_1/1_Confidence_0.000_Length_746, Sample1_Locus_2001_Transcript_4/7_Confidence_0.600_Length_1686, SampleTS3_Locus_23038_Transcript_2/3_Confidence_0.333_Length_1864, SampleTS2_Locus_4874_Transcript_6/12_Confidence_0.158_Length_1042, Sample3_Locus_23176_Transcript_2/3_Confidence_0.333_Length_1282, SampleTS1_Locus_5373_Transcript_26/33_Confidence_0.065_Length_1620, Sample6_Locus_12695_Transcript_1/2_Confidence_0.333_Length_2218, SampleTS3_Locus_23264_Transcript_1/3_Confidence_0.500_Length_845, SampleTS2_Locus_1750_Transcript_17/20_Confidence_0.182_Length_1568, Sample3_Locus_17004_Transcript_3/5_Confidence_0.286_Length_574, Sample3_Locus_3732_Transcript_3/7_Confidence_0.545_Length_970, Sample6_Locus_293_Transcript_1/2_Confidence_0.333_Length_875, SampleTS1_Locus_789_Transcript_9/11_Confidence_0.238_Length_2300,

			ec:1.2.1.3 dehydrogenase (NAD+), ec:2.3.1.9 Cacetyltransferase, ec:2.3.1.1 Nacetyltransferase, ec:1.1.1.37 dehydrogenase, ec:1.1.1.35 dehydrogenase, ec:4.4.1.1 gammalyase, ec:1.1.1.44 dehydrogenase (NADP+dependent, decarboxylating), ec:1.1.1.49 dehydrogenase (NADP+), ec:4.3.3.7 synthase, ec:5.4.2.8 mannose phosphomutase, ec:6.3.5.3 synthase, ec:1.14.13.70 14alphademethylase, ec:2.3.3.8 citrate synthase, ec:1.5.1.2 reductase, ec:2.4.2.14 phosphoribosyl diphosphate 5amidotransferase, ec:2.3.3.1 (Si)synthase, ec:1.2.4.2 dehydrogenase (succinyltransferring), ec:1.2.4.1 dehydrogenase (acetyltransferring), ec:1.2.4.4 dehydrogenase (2methylpropanoyltransferring), ec:5.1.3.13 3,5epimerase, ec:5.3.1.24 isomerase		SampleTS3_Locus_1827_Transcript_3/3_Confidence_0.000_Length_1033, SampleTS3_Locus_11808_Transcript_9/11_Confidence_0.565_Length_1006, Sample6_Locus_5355_Transcript_5/8_Confidence_0.577_Length_1830, SampleTS2_Locus_2923_Transcript_3/4_Confidence_0.333_Length_1277, Sample6_Locus_10552_Transcript_5/5_Confidence_0.000_Length_1013, SampleTS1_Locus_3938_Transcript_9/14_Confidence_0.467_Length_2040, SampleTS1_Locus_4380_Transcript_8/12_Confidence_0.400_Length_2044, SampleTS3_Locus_2991_Transcript_5/8_Confidence_0.400_Length_1799, SampleTS1_Locus_10980_Transcript_1/3_Confidence_0.667_Length_1432, Sample1_Locus_10139_Transcript_1/10_Confidence_0.538_Length_1144, Sample6_Locus_17319_Transcript_1/1_Confidence_0.000_Length_1109, Sample1_Locus_10749_Transcript_3/5_Confidence_0.800_Length_2198, SampleTS2_Locus_4266_Transcript_1/3_Confidence_0.333_Length_1522, SampleTS3_Locus_443_Transcript_4/7_Confidence_0.429_Length_1718, SampleTS1_Locus_743_Transcript_11/14_Confidence_0.154_Length_1525, Sample3_Locus_10439_Transcript_3/6_Confidence_0.667_Length_1846, SampleTS2_Locus_4114_Transcript_5/7_Confidence_0.267_Length_2080, Sample6_Locus_6489_Transcript_7/13_Confidence_0.200_Length_1232, SampleTS2_Locus_3113_Transcript_1/3_Confidence_0.667_Length_750, SampleTS3_Locus_20806_Transcript_1/1_Confidence_0.000_Length_3106, Sample3_Locus_1003_Transcript_1/3_Confidence_0.750_Length_1416, SampleTS2_Locus_9103_Transcript_1/2_Confidence_0.667_Length_685, Sample1_Locus_24165_Transcript_4/4_Confidence_0.444_Length_605, SampleTS2_Locus_9103_Transcript_1/2_Confidence_0.667_Length_685, Sample3_Locus_2806_Transcript_6/14_Confidence_0.167_Length_1197, SampleTS1_Locus_12931_Transcript_3/3_Confidence_0.200_Length_1113

**Purine metabolism**	map00230	39	ec:6.3.3.1 cycloligase, ec:2.1.2.2 formyltransferase, ec:2.7.4.8 kinase, ec:2.7.4.6 kinase, ec:2.7.4.3 kinase, ec:3.5.2.5 ec:3.5.2.5 allantoinase, ec:6.3.4.4 synthase, ec:3.6.1.3 adenylpyrophosphatase, ec:3.6.1.6 diphosphate phosphatase, ec:3.6.1.9 diphosphatase, ec:4.1.1.21 carboxylase, ec:3.1.4.17 phosphodiesterase, ec:3.5.4.4 deaminase, ec:1.17.4.1 reductase, ec:2.7.1.40 kinase, ec:3.5.4.6 deaminase, ec:6.3.2.6 synthase, ec:3.1.4.35 phosphodiesterase, ec:2.7.1.25 kinase, ec:2.7.1.20 kinase, ec:3.6.1.13 diphosphatase, ec:3.6.1.15 phosphatase, ec:2.7.7.4 adenylyltransferase, ec:3.6.1.21 diphosphatase, ec:3.6.1.29 dinucleosidetriphosphatase, ec:2.7.7.6 RNA polymerase, ec:2.7.7.7 DNA polymerase, ec:2.7.6.5 diphosphokinase, ec:2.7.6.1 diphosphokinase, ec:1.7.3.3 urate hydroxylase, ec:4.6.1.1 cyclase, ec:4.6.1.2 cyclase, ec:1.1.1.205 dehydrogenase, ec:3.1.7.2	2, 1, 4, 5, 7, 1, 1, 215, 5, 5, 2, 48, 3, 1, 7, 1, 1, 27, 2, 1, 2, 278, 3, 4, 2, 11, 6, 1, 1, 1, 10, 8, 1, 2, 2, 1, 2, 1, 1	SampleTS1_Locus_2676_Transcript_23/50_Confidence_0.046_Length_813, Sample3_Locus_8866_Transcript_2/4_Confidence_0.714_Length_1157, SampleTS2_Locus_12481_Transcript_10/11_Confidence_0.194_Length_1103, SampleTS1_Locus_6152_Transcript_2/7_Confidence_0.375_Le

			3'diphosphatase, ec:6.3.5.3 synthase, ec:2.4.2.14 phosphoribosyl diphosphate 5amidotransferase, ec:2.4.2.7 phosphoribosyltransferase, ec:2.4.2.8 phosphoribosyltransferase, ec:3.5.1.5 ec:3.5.1.5 urease		

**Pyruvate metabolism**	map00620	27	ec:6.4.1.2 carboxylase, ec:2.3.3.13 synthase, ec:4.1.1.49 carboxykinase (ATP), ec:3.6.1.7 acetylphosphatase, ec:4.1.1.31 carboxylase, ec:4.1.1.32 carboxykinase (GTP), ec:4.2.1.2 hydratase, ec:2.7.1.40 kinase, ec:4.1.1.3 decarboxylase, ec:6.2.1.1 ligase, ec:2.3.1.12 acetyltransferase, ec:1.2.1.10 dehydrogenase (acetylating), ec:2.3.1.54 Cacetyltransferase, ec:1.8.1.4 dehydrogenase, ec:3.1.2.6 hydrolase, ec:1.1.2.3 dehydrogenase (cytochrome), ec:1.1.2.4 dehydrogenase (cytochrome), ec:2.7.9.1 phosphate dikinase, ec:1.2.1.3 dehydrogenase (NAD+), ec:2.3.1.9 Cacetyltransferase, ec:1.1.1.37 dehydrogenase, ec:4.4.1.5 lyase, ec:1.1.1.38 dehydrogenase (oxaloacetatedecarboxylating), ec:1.1.1.40 dehydrogenase (oxaloacetatedecarboxylating) (NADP+), ec:2.3.3.9 synthase, ec:1.1.1.28 dehydrogenase, ec:1.2.4.1 dehydrogenase (acetyltransferring)	2, 1, 1, 1, 1, 1, 2, 7, 1, 1, 2, 2, 3, 1, 2, 2, 1, 3, 6, 1, 1, 1, 1, 2, 1, 1, 2	Sample3_Locus_8920_Transcript_2/3_Confidence_0.333_Length_2038, SampleTS1_Locus_1155_Transcript_11/16_Confidence_0.625_Length_2240, Sample1_Locus_7894_Transcript_1/3_Confidence_0.250_Length_2075, Sample6_Locus_11174_Transcript_1/1_Confidence_0.000_Length_1998, SampleTS3_Locus_13938_Transcript_1/2_Confidence_0.333_Length_655, Sample3_Locus_7378_Transcript_3/6_Confidence_0.417_Length_3416, Sample6_Locus_11174_Transcript_1/1_Confidence_0.000_Length_1998, Sample3_Locus_2252_Transcript_9/13_Confidence_0.647_Length_1708, Sample6_Locus_1413_Transcript_3/3_Confidence_0.000_Length_1802, Sample6_Locus_12247_Transcript_7/9_Confidence_0.429_Length_1396, SampleTS1_Locus_11723_Transcript_6/16_Confidence_0.073_Length_1679, SampleTS1_Locus_7761_Transcript_5/10_Confidence_0.105_Length_1358, SampleTS2_Locus_14711_Transcript_4/7_Confidence_0.273_Length_1393, Sample6_Locus_2327_Transcript_47/56_Confidence_0.195_Length_2423, Sample6_Locus_22851_Transcript_2/3_Confidence_0.714_Length_1077, SampleTS1_Locus_2696_Transcript_4/6_Confidence_0.545_Length_1155, SampleTS2_Locus_3140_Transcript_18/19_Confidence_0.323_Length_745, SampleTS3_Locus_456_Transcript_2/6_Confidence_0.200_Length_2075, SampleTS2_Locus_8836_Transcript_1/2_Confidence_0.667_Length_1729, SampleTS3_Locus_5316_Transcript_1/2_Confidence_0.750_Length_1843, SampleTS3_Locus_400_Transcript_6/29_Confidence_0.422_Length_2479, Sample6_Locus_1524_Transcript_26/30_Confidence_0.185_Length_3215, Sample6_Locus_961_Transcript_2/3_Confidence_0.250_Length_1478, Sample1_Locus_27983_Transcript_6/13_Confidence_0.294_Length_763, SampleTS2_Locus_20366_Transcript_1/7_Confidence_0.273_Length_670, SampleTS3_Locus_4699_Transcript_6/10_Confidence_0.579_Length_1963, Sample1_Locus_24616_Transcript_1/1_Confidence_0.000_Length_806, SampleTS3_Locus_23179_Transcript_2/6_Confidence_0.500_Length_1144, Sample3_Locus_1427_Transcript_1/1_Confidence_0.000_Length_746, SampleTS2_Locus_13872_Transcript_6/7_Confidence_0.500_Length_988, Sample6_Locus_3300_Transcript_2/5_Confidence_0.700_Length_1810, SampleTS1_Locus_793_Transcript_3/3_Confidence_0.000_Length_1036, Sample6_Locus_2229_Transcript_3/3_Confidence_0.000_Length_1460, SampleTS2_Locus_3926_Transcript_9/12_Confidence_0.038_Length_2167, SampleTS3_Locus_23038_Transcript_2/3_Confidence_0.333_Length_1864, SampleTS2_Locus_4874_Transcript_6/12_Confidence_0.158_Length_1042, Sample3_Locus_23176_Transcript_2/3_Confidence_0.333_Length_1282, SampleTS1_Locus_5373_Transcript_26/33_Confidence_0.065_Length_1620, Sample6_Locus_12695_Transcript_1/2_Confidence_0.333_Length_2218, SampleTS3_Locus_23264_Transcript_1/3_Confidence_0.500_Length_845, SampleTS2_Locus_1750_Transcript_17/20_Confidence_0.182_Length_1568, Sample6_Locus_293_Transcript_1/2_Confidence_0.333_Length_875, SampleTS3_Locus_10192_Transcript_2/2_Confidence_0.333_Length_1357, SampleTS2_Locus_3140_Transcript_18/19_Confidence_0.323_Length_745, Sample3_Locus_24109_Transcript_4/4_Confidence_0.000_Length_2026, SampleTS2_Locus_3140_Transcript_18/19_Confidence_0.323_Length_745, SampleTS1_Locus_8328_Transcript_7/9_Confidence_0.407_Length_1307, Sample6_Locus_1387_Transcript_42/45_Confidence_0.033_Length_1697, Sample3_Locus_1003_Transcript_1/3_Confidence_0.750_Length_1416, SampleTS2_Locus_9103_Transcript_1/2_Confidence_0.667_Length_685

**Amino sugar and nucleotide sugar metabolism**	map00520	25	ec:2.6.1.16 transaminase (isomerizing), ec:2.7.1.44 galacturonokinase (phosphorylating) Dgalacturonic acid kinase, ec:2.7.1.2 glucokinase (phosphorylating), ec:2.7.1.1 hexokinase type IV glucokinase, ec:2.7.1.6 galactokinase (phosphorylating), ec:2.7.1.4 fructokinase (phosphorylating), ec:5.1.3.2 4epimerase, ec:5.1.3.7 4epimerase, ec:5.1.3.5 4epimerase, ec:4.2.1.76 4,6dehydratase, ec:2.7.7.9 uridylyltransferase, ec:5.3.1.9 isomerase, ec:2.7.7.64 uridylyltransferase, ec:5.4.99.30 mutase, ec:2.7.7.44 uridylyltransferase, ec:2.7.7.27 adenylyltransferase, ec:2.7.7.22 guanylyltransferase (GDP), ec:2.7.7.23 diphosphorylase, ec:2.7.7.10 uridylyltransferase, ec:1.6.2.2 reductase, ec:2.3.1.4 Nacetyltransferase, ec:5.4.2.8 mannose phosphomutase, ec:1.1.1.22 6dehydrogenase, ec:5.1.3.18 3,5epimerase, ec:3.2.1.14 ChiC	1, 1, 1, 1, 2, 1, 1, 1, 1, 1, 5, 1, 5, 1, 1, 1, 1, 1, 1, 3, 1, 1, 2, 1, 2	Sample6_Locus_5350_Transcript_7/9_Confidence_0.333_Length_2351, Sample3_Locus_7508_Transcript_1/2_Confidence_0.333_Length_994, Sample6_Locus_5095_Transcript_5/8_Confidence_0.300_Length_1241, Sample6_Locus_5095_Transcript_5/8_Confidence_0.300_Length_1241, Sample3_Locus_7188_Transcript_3/12_Confidence_0.615_Length_971, Sample3_Locus_7508_Transcript_1/2_Confidence_0.333_Length_994, Sample6_Locus_5095_Transcript_5/8_Confidence_0.300_Length_1241, SampleTS2_Locus_4938_Transcript_4/9_Confidence_0.273_Length_771, SampleTS2_Locus_4938_Transcript_4/9_Confidence_0.273_Length_771, SampleTS2_Locus_4938_Transcript_4/9_Confidence_0.273_Length_771, Sample3_Locus_2806_Transcript_6/14_Confidence_0.167_Length_1197, SampleTS3_Locus_2330_Transcript_4/4_Confidence_0.000_Length_1686, SampleTS1_Locus_16292_Transcript_4/5_Confidence_0.600_Length_1341 , Sample3_Locus_4208_Transcript_8/15_Confidence_0.091_Length_2117, Sample3_Locus_40_Transcript_1/1_Confidence_0.000_Length_1702, Sample3_Locus_14770_Transcript_1/1_Confidence_0.000_Length_1819, SampleTS1_Locus_12696_Transcript_3/3_Confidence_0.000_Length_1743 , SampleTS3_Locus_2330_Transcript_4/4_Confidence_0.000_Length_1686, SampleTS1_Locus_16292_Transcript_4/5_Confidence_0.600_Length_1341 , Sample3_Locus_4208_Transcript_8/15_Confidence_0.091_Length_2117, Sample3_Locus_40_Transcript_1/1_Confidence_0.000_Length_1702, Sample3_Locus_14770_Transcript_1/1_Confidence_0.000_Length_1819, SampleTS1_Locus_24714_Transcript_1/1_Confidence_0.000_Length_794, SampleTS3_Locus_2330_Transcript_4/4_Confidence_0.000_Length_1686, Sample3_Locus_6692_Transcript_2/3_Confidence_0.333_Length_1421, Sample3_Locus_982_Transcript_1/1_Confidence_0.000_Length_2070, Sample3_Locus_4208_Transcript_8/15_Confidence_0.091_Length_2117, SampleTS3_Locus_2330_Transcript_4/4_Confidence_0.000_Length_1686, SampleTS1_Locus_26901_Transcript_2/4_Confidence_0.095_Length_536, SampleTS2_Locus_13872_Transcript_6/7_Confidence_0.500_Length_988, Sample3_Locus_15798_Transcript_2/3_Confidence_0.333_Length_1030, Sample1_Locus_22_Transcript_24/64_Confidence_0.125_Length_1686, Sample1_Locus_10139_Transcript_1/10_Confidence_0.538_Length_1144, SampleTS3_Locus_1975_Transcript_19/64_Confidence_0.064_Length_546 , SampleTS2_Locus_4_Transcript_35/36_Confidence_0.021_Length_1580, Sample3_Locus_22932_Transcript_1/1_Confidence_0.000_Length_1277, Sample3_Locus_17381_Transcript_6/8_Confidence_0.324_Length_685, SampleTS1_Locus_7160_Transcript_3/3_Confidence_0.333_Length_2188

**Glycolysis / Gluconeogenesis**	map00010	21	ec:4.1.2.13 aldolase, ec:4.1.1.49 carboxykinase (ATP), ec:4.1.1.32 carboxykinase (GTP), ec:2.7.1.40 kinase, ec:2.7.1.2 glucokinase (phosphorylating), ec:2.7.1.1 hexokinase type IV glucokinase, ec:1.2.1.59 dehydrogenase (NAD(P)+) (phosphorylating), ec:4.1.1.1 decarboxylase, ec:6.2.1.1 ligase, ec:5.1.3.3 1epimerase, ec:2.3.1.12 acetyltransferase, ec:3.1.3.11 hexose diphosphatase, ec:1.2.1.12 dehydrogenase (phosphorylating), ec:1.8.1.4 dehydrogenase, ec:5.3.1.9 isomerase, ec:1.1.1.2 dehydrogenase (NADP+), ec:1.1.1.1 dehydrogenase, ec:4.2.1.11 hydratase, ec:1.2.1.5 dehydrogenase [NAD(P)+], ec:1.2.1.3 dehydrogenase (NAD+), ec:1.2.4.1 dehydrogenase (acetyltransferring)	2, 1, 1, 7, 1, 1, 3, 1, 1, 1, 2, 2, 5, 1, 1, 10, 5, 1, 1, 6, 2	Sample3_Locus_324_Transcript_3/17_Confidence_0.222_Length_1711, Sample6_Locus_28252_Transcript_2/3_Confidence_0.500_Length_808, Sample6_Locus_11174_Transcript_1/1_Confidence_0.000_Length_1998, Sample6_Locus_11174_Transcript_1/1_Confidence_0.000_Length_1998, Sample6_Locus_12247_Transcript_7/9_Confidence_0.429_Length_1396, SampleTS1_Locus_11723_Transcript_6/16_Confidence_0.073_Length_1679, SampleTS1_Locus_7761_Transcript_5/10_Confidence_0.105_Length_1358, SampleTS2_Locus_14711_Transcript_4/7_Confidence_0.273_Length_1393, Sample6_Locus_2327_Transcript_47/56_Confidence_0.195_Length_2423, Sample6_Locus_22851_Transcript_2/3_Confidence_0.714_Length_1077, SampleTS1_Locus_2696_Transcript_4/6_Confidence_0.545_Length_1155, Sample6_Locus_5095_Transcript_5/8_Confidence_0.300_Length_1241, Sample6_Locus_5095_Transcript_5/8_Confidence_0.300_Length_1241, SampleTS2_Locus_819_Transcript_16/18_Confidence_0.250_Length_1000, SampleTS2_Locus_12618_Transcript_4/6_Confidence_0.571_Length_1272, Sample6_Locus_997_Transcript_9/17_Confidence_0.200_Length_950, Sample3_Locus_4596_Transcript_3/6_Confidence_0.400_Length_1157, SampleTS3_Locus_456_Transcript_2/6_Confidence_0.200_Length_2075, Sample3_Locus_10443_Transcript_10/11_Confidence_0.700_Length_2690, SampleTS2_Locus_8836_Transcript_1/2_Confidence_0.667_Length_1729, SampleTS3_Locus_5316_Transcript_1/2_Confidence_0.750_Length_1843, Sample3_Locus_10262_Transcript_4/5_Confidence_0.062_Length_1337, Sample6_Locus_2283_Transcript_7/17_Confidence_0.225_Length_1818, SampleTS2_Locus_819_Transcript_16/18_Confidence_0.250_Length_1000, Sample1_Locus_7420_Transcript_10/14_Confidence_0.154_Length_1346, SampleTS2_Locus_12618_Transcript_4/6_Confidence_0.571_Length_1272, Sample6_Locus_997_Transcript_9/17_Confidence_0.200_Length_950, Sample1_Locus_393_Transcript_6/7_Confidence_0.556_Length_1466, SampleTS3_Locus_4699_Transcript_6/10_Confidence_0.579_Length_1963, SampleTS1_Locus_12696_Transcript_3/3_Confidence_0.000_Length_1743, Sample6_Locus_15547_Transcript_5/8_Confidence_0.545_Length_1332, Sample6_Locus_7369_Transcript_3/6_Confidence_0.200_Length_1828, SampleTS1_Locus_2734_Transcript_1/3_Confidence_0.333_Length_2491, SampleTS2_Locus_15764_Transcript_1/1_Confidence_0.000_Length_1068, Sample3_Locus_27117_Transcript_1/3_Confidence_0.250_Length_685, Sample6_Locus_6954_Transcript_4/5_Confidence_0.500_Length_1201, Sample6_Locus_4129_Transcript_8/27_Confidence_0.107_Length_571, SampleTS3_Locus_8149_Transcript_4/10_Confidence_0.118_Length_927, SampleTS1_Locus_6731_Transcript_14/15_Confidence_0.246_Length_2303, SampleTS1_Locus_10087_Transcript_6/7_Confidence_0.167_Length_948, SampleTS2_Locus_4874_Transcript_6/12_Confidence_0.158_Length_1042, Sample6_Locus_1524_Transcript_26/30_Confidence_0.185_Length_3215, SampleTS2_Locus_263_Transcript_1/3_Confidence_0.250_Length_2184, SampleTS3_Locus_400_Transcript_6/29_Confidence_0.422_Length_2479, SampleTS3_Locus_23264_Transcript_1/3_Confidence_0.500_Length_845, SampleTS1_Locus_3608_Transcript_5/7_Confidence_0.222_Length_1514, SampleTS1_Locus_5373_Transcript_26/33_Confidence_0.065_Length_1620, SampleTS3_Locus_23038_Transcript_2/3_Confidence_0.333_Length_1864, SampleTS2_Locus_4874_Transcript_6/12_Confidence_0.158_Length_1042, Sample3_Locus_23176_Transcript_2/3_Confidence_0.333_Length_1282, SampleTS1_Locus_5373_Transcript_26/33_Confidence_0.065_Length_1620, Sample6_Locus_12695_Transcript_1/2_Confidence_0.333_Length_2218, SampleTS3_Locus_23264_Transcript_1/3_Confidence_0.500_Length_845, Sample3_Locus_1003_Transcript_1/3_Confidence_0.750_Length_1416, SampleTS2_Locus_9103_Transcript_1/2_Confidence_0.667_Length_685

**Starch and sucrose metabolism**	map00500	21	ec:3.6.1.9 diphosphatase, ec:2.7.1.2 glucokinase (phosphorylating), ec:2.7.1.1 hexokinase type IV glucokinase, ec:2.7.1.4 fructokinase (phosphorylating), ec:3.1.2.21 hydrolase, ec:3.1.3.12 trehalose 6phosphatase, ec:3.6.1.21 diphosphatase, ec:2.7.7.9 uridylyltransferase, ec:3.2.1.2 saccharogen amylase, ec:3.2.1.1 glycogenase, ec:3.2.1.68 debranching enzyme, ec:5.3.1.9 isomerase, ec:3.2.1.58 1,3betaglucosidase, ec:2.7.7.27 adenylyltransferase, ec:3.2.1.21 gentiobiase, ec:2.4.1.1 phosphorylase, ec:2.4.1.18 branching enzyme, ec:2.4.1.15 synthase (UDPforming), ec:2.4.1.14 synthase, ec:2.4.1.12 synthase (UDPforming), ec:2.4.1.25 disproportionating enzyme	5, 1, 1, 1, 1, 2, 4, 5, 2, 4, 1, 1, 1, 1, 2, 4, 2, 2, 1, 1, 1	Sample6_Locus_1849_Transcript_4/6_Confidence_0.429_Length_866, Sample3_Locus_345_Transcript_6/8_Confidence_0.455_Length_1801, SampleTS3_Locus_5573_Transcript_4/5_Confidence_0.333_Length_940, Sample3_Locus_10699_Transcript_3/4_Confidence_0.400_Length_957, Sample3_Locus_27182_Transcript_2/3_Confidence_0.250_Length_1010, Sample6_Locus_5095_Transcript_5/8_Confidence_0.300_Length_1241, Sample6_Locus_5095_Transcript_5/8_Confidence_0.300_Length_1241, Sample6_Locus_5095_Transcript_5/8_Confidence_0.300_Length_1241, Sample6_Locus_10875_Transcript_2/10_Confidence_0.167_Length_1095, SampleTS3_Locus_6160_Transcript_3/3_Confidence_0.167_Length_2057, Sample6_Locus_6071_Transcript_1/2_Confidence_0.333_Length_2182, Sample3_Locus_345_Transcript_6/8_Confidence_0.455_Length_1801, SampleTS2_Locus_5310_Transcript_3/15_Confidence_0.167_Length_1115, Sample3_Locus_734_Transcript_8/12_Confidence_0.176_Length_1222, SampleTS3_Locus_5794_Transcript_5/6_Confidence_0.125_Length_1722, SampleTS3_Locus_2330_Transcript_4/4_Confidence_0.000_Length_1686, SampleTS1_Locus_16292_Transcript_4/5_Confidence_0.600_Length_1341, Sample3_Locus_4208_Transcript_8/15_Confidence_0.091_Length_2117, Sample3_Locus_40_Transcript_1/1_Confidence_0.000_Length_1702, Sample3_Locus_14770_Transcript_1/1_Confidence_0.000_Length_1819, Sample3_Locus_2003_Transcript_3/7_Confidence_0.300_Length_1588, Sample1_Locus_23952_Transcript_4/4_Confidence_0.167_Length_1585, SampleTS3_Locus_2836_Transcript_6/9_Confidence_0.091_Length_1339, SampleTS2_Locus_11527_Transcript_3/7_Confidence_0.625_Length_1438, SampleTS1_Locus_833_Transcript_7/9_Confidence_0.300_Length_1571, SampleTS3_Locus_119_Transcript_5/13_Confidence_0.087_Length_2030, SampleTS3_Locus_2836_Transcript_6/9_Confidence_0.091_Length_1339, SampleTS1_Locus_12696_Transcript_3/3_Confidence_0.000_Length_1743, SampleTS2_Locus_9286_Transcript_1/1_Confidence_0.000_Length_2119, Sample3_Locus_6692_Transcript_2/3_Confidence_0.333_Length_1421, Sample1_Locus_25240_Transcript_4/4_Confidence_0.429_Length_2225, SampleTS2_Locus_9286_Transcript_1/1_Confidence_0.000_Length_2119, Sample3_Locus_982_Transcript_1/1_Confidence_0.000_Length_2070, SampleTS3_Locus_4468_Transcript_6/7_Confidence_0.125_Length_3339, SampleTS3_Locus_4468_Transcript_2/7_Confidence_0.250_Length_1403, SampleTS2_Locus_652_Transcript_7/8_Confidence_0.417_Length_1591, Sample1_Locus_1836_Transcript_6/7_Confidence_0.364_Length_3038, Sample1_Locus_1040_Transcript_6/8_Confidence_0.333_Length_2600, SampleTS3_Locus_6160_Transcript_3/3_Confidence_0.167_Length_2057, Sample6_Locus_6071_Transcript_1/2_Confidence_0.333_Length_2182, Sample6_Locus_836_Transcript_8/21_Confidence_0.226_Length_3574, SampleTS1_Locus_24714_Transcript_1/1_Confidence_0.000_Length_794, Sample6_Locus_6420_Transcript_1/2_Confidence_0.333_Length_2262

**Alanine, aspartate and glutamate metabolism**	map00250	21	ec:2.6.1.12 transaminase, ec:2.6.1.15 transaminase, ec:2.6.1.16 transaminase (isomerizing), ec:2.6.1.19 transaminase, ec:6.3.4.4 synthase, ec:1.4.3.2 oxidase, ec:6.3.1.1 ligase, ec:6.3.1.2 synthetase, ec:2.1.3.2 carbamoyltransferase, ec:2.6.1.44 transaminase, ec:4.1.1.15 decarboxylase, ec:6.3.4.16 synthase (ammonia), ec:1.4.3.16 oxidase, ec:2.6.1.2 transaminase, ec:2.6.1.1 transaminase, ec:1.4.1.14 synthase (NADH), ec:6.3.5.4 synthase (glutaminehydrolysing), ec:6.3.5.5 synthase (glutaminehydrolysing), ec:2.4.2.14 phosphoribosyldiphosphate 5amidotransferase, ec:3.5.1.2 glutaminase I, ec:3.5.1.3 alphaketo acidomegaamidase	3, 1, 1, 1, 1, 1, 2, 2, 1, 6, 1, 1, 1, 3, 3, 2, 2, 2, 1, 2, 1	Sample6_Locus_2195_Transcript_14/16_Confidence_0.176_Length_1741, SampleTS3_Locus_714_Transcript_7/14_Confidence_0.087_Length_1194, Sample1_Locus_19449_Transcript_1/1_Confidence_0.000_Length_1704, Sample6_Locus_22798_Transcript_3/3_Confidence_0.250_Length_666, Sample6_Locus_5350_Transcript_7/9_Confidence_0.333_Length_2351, SampleTS1_Locus_13173_Transcript_7/8_Confidence_0.190_Length_747, Sample3_Locus_13585_Transcript_1/1_Confidence_0.000_Length_1351, SampleTS1_Locus_1041_Transcript_1/5_Confidence_0.400_Length_2190, Sample6_Locus_5745_Transcript_6/10_Confidence_0.065_Length_1149, Sample6_Locus_7334_Transcript_8/13_Confidence_0.571_Length_1873, Sample1_Locus_3256_Transcript_2/10_Confidence_0.182_Length_1415, SampleTS2_Locus_4400_Transcript_6/7_Confidence_0.333_Length_1376, Sample1_Locus_3772_Transcript_1/3_Confidence_0.333_Length_1976, Sample6_Locus_2195_Transcript_14/16_Confidence_0.176_Length_1741, SampleTS3_Locus_714_Transcript_7/14_Confidence_0.087_Length_1194, Sample3_Locus_1833_Transcript_4/6_Confidence_0.500_Length_1265, SampleTS1_Locus_13173_Transcript_7/8_Confidence_0.190_Length_747, Sample1_Locus_19449_Transcript_1/1_Confidence_0.000_Length_1704, Sample6_Locus_15604_Transcript_2/4_Confidence_0.400_Length_1416, SampleTS3_Locus_5084_Transcript_15/17_Confidence_0.206_Length_1751, SampleTS3_Locus_15776_Transcript_1/2_Confidence_0.333_Length_1102, SampleTS1_Locus_1041_Transcript_1/5_Confidence_0.400_Length_2190, Sample6_Locus_2195_Transcript_14/16_Confidence_0.176_Length_1741, SampleTS3_Locus_714_Transcript_7/14_Confidence_0.087_Length_1194, Sample1_Locus_19449_Transcript_1/1_Confidence_0.000_Length_1704, Sample1_Locus_26802_Transcript_1/2_Confidence_0.667_Length_439, Sample6_Locus_13664_Transcript_1/2_Confidence_0.333_Length_862, SampleTS2_Locus_6646_Transcript_2/2_Confidence_0.333_Length_1520, SampleTS3_Locus_21707_Transcript_1/2_Confidence_0.333_Length_2460, SampleTS1_Locus_1310_Transcript_14/17_Confidence_0.222_Length_1901, Sample6_Locus_5745_Transcript_6/10_Confidence_0.065_Length_1149, Sample6_Locus_7334_Transcript_8/13_Confidence_0.571_Length_1873, SampleTS2_Locus_5848_Transcript_1/1_Confidence_0.000_Length_1345, SampleTS3_Locus_15776_Transcript_1/2_Confidence_0.333_Length_1102, Sample3_Locus_10439_Transcript_3/6_Confidence_0.667_Length_1846, Sample6_Locus_3307_Transcript_18/23_Confidence_0.163_Length_735, SampleTS1_Locus_5169_Transcript_10/19_Confidence_0.593_Length_3162, Sample6_Locus_1849_Transcript_4/6_Confidence_0.429_Length_866

**Cysteine and methionine metabolism**	map00270	20	ec:4.1.1.50 decarboxylase, ec:4.4.1.15 desulfhydrase, ec:4.4.1.14 synthase, ec:1.4.3.2 oxidase, ec:2.6.1.57 transaminase, ec:2.7.2.4 kinase, ec:2.6.1.42 transaminase, ec:2.6.1.44 transaminase, ec:1.13.11.54 dioxygenase [iron(II)requiring], ec:2.3.1.30 Oacetyltransferase, ec:1.8.4.13 (S)Soxide reductase, ec:3.5.99.7 deaminase, ec:2.1.1.13 synthase, ec:2.6.1.5 transaminase, ec:3.3.1.1 Sadenosylhomocysteine synthase, ec:2.5.1.47 synthase, ec:2.6.1.1 transaminase, ec:1.1.1.37 dehydrogenase, ec:4.4.1.1 gammalyase, ec:5.3.1.23 isomerase	1, 1, 1, 1, 2, 1, 2, 6, 2, 1, 2, 1, 1, 2, 2, 3, 3, 1, 2, 1	SampleTS1_Locus_23304_Transcript_2/3_Confidence_0.333_Length_2324, SampleTS2_Locus_6805_Transcript_3/5_Confidence_0.429_Length_1160, SampleTS2_Locus_10700_Transcript_2/5_Confidence_0.400_Length_1240, SampleTS1_Locus_1041_Transcript_1/5_Confidence_0.400_Length_2190, Sample1_Locus_26802_Transcript_1/2_Confidence_0.667_Length_439, Sample6_Locus_13664_Transcript_1/2_Confidence_0.333_Length_862, Sample3_Locus_2031_Transcript_1/1_Confidence_0.000_Length_2262, Sample1_Locus_18777_Transcript_2/4_Confidence_0.923_Length_1416, Sample3_Locus_5952_Transcript_4/4_Confidence_0.000_Length_2586, Sample6_Locus_2195_Transcript_14/16_Confidence_0.176_Length_1741, SampleTS3_Locus_714_Transcript_7/14_Confidence_0.087_Length_1194, Sample3_Locus_1833_Transcript_4/6_Confidence_0.500_Length_1265, SampleTS1_Locus_13173_Transcript_7/8_Confidence_0.190_Length_747, Sample1_Locus_19449_Transcript_1/1_Confidence_0.000_Length_1704, Sample6_Locus_15604_Transcript_2/4_Confidence_0.400_Length_1416, Sample3_Locus_25024_Transcript_2/3_Confidence_0.400_Length_705, Sample3_Locus_13790_Transcript_1/1_Confidence_0.000_Length_969, Sample3_Locus_6377_Transcript_4/4_Confidence_0.125_Length_1830, SampleTS3_Locus_3182_Transcript_3/5_Confidence_0.400_Length_494, SampleTS1_Locus_1262_Transcript_63/105_Confidence_0.008_Length_707, SampleTS2_Locus_6805_Transcript_3/5_Confidence_0.429_Length_1160, SampleTS1_Locus_8020_Transcript_2/4_Confidence_0.333_Length_2411, Sample1_Locus_26802_Transcript_1/2_Confidence_0.667_Length_439, Sample6_Locus_13664_Transcript_1/2_Confidence_0.333_Length_862, Sample1_Locus_1471_Transcript_7/9_Confidence_0.333_Length_1784, Sample6_Locus_23233_Transcript_1/1_Confidence_0.000_Length_2733, Sample1_Locus_15681_Transcript_1/1_Confidence_0.000_Length_1030, Sample6_Locus_5355_Transcript_5/8_Confidence_0.577_Length_1830, SampleTS2_Locus_2923_Transcript_3/4_Confidence_0.333_Length_1277, Sample1_Locus_26802_Transcript_1/2_Confidence_0.667_Length_439, Sample6_Locus_13664_Transcript_1/2_Confidence_0.333_Length_862, SampleTS2_Locus_6646_Transcript_2/2_Confidence_0.333_Length_1520, Sample6_Locus_293_Transcript_1/2_Confidence_0.333_Length_875, Sample6_Locus_5355_Transcript_5/8_Confidence_0.577_Length_1830, SampleTS2_Locus_2923_Transcript_3/4_Confidence_0.333_Length_1277, Sample1_Locus_12270_Transcript_7/8_Confidence_0.133_Length_2318

**Glyoxylate and dicarboxylate metabolism**	map00630	19	ec:2.1.2.1 hydroxymethyltransferase, ec:6.4.1.3 carboxylase, ec:4.1.1.39 carboxylase, ec:4.2.1.3 hydratase, ec:6.3.1.2 synthetase, ec:4.1.3.1 lyase, ec:1.11.1.6 equilase, ec:2.6.1.45 transaminase, ec:1.4.7.1 synthase (ferredoxin), ec:5.4.99.2 mutase, ec:2.6.1.4 transaminase, ec:1.1.1.77 reductase, ec:1.1.3.15 oxidase, ec:3.5.1.10 deformylase, ec:2.3.1.9 Cacetyltransferase, ec:1.1.1.37 dehydrogenase, ec:1.1.1.36 reductase, ec:2.3.3.9 synthase, ec:2.3.3.1 (Si)synthase	2, 2, 1, 1, 2, 1, 3, 1, 1, 1, 3, 1, 1, 2, 1, 1, 1, 1, 3	Sample1_Locus_3932_Transcript_7/11_Confidence_0.400_Length_1725, SampleTS1_Locus_10618_Transcript_3/4_Confidence_0.400_Length_1517, SampleTS1_Locus_1968_Transcript_1/3_Confidence_0.400_Length_530, SampleTS1_Locus_1155_Transcript_11/16_Confidence_0.625_Length_2240, SampleTS1_Locus_4324_Transcript_3/4_Confidence_0.286_Length_1536, SampleTS2_Locus_7424_Transcript_6/8_Confidence_0.286_Length_3139, Sample1_Locus_3256_Transcript_2/10_Confidence_0.182_Length_1415, SampleTS2_Locus_4400_Transcript_6/7_Confidence_0.333_Length_1376, Sample6_Locus_3001_Transcript_26/36_Confidence_0.070_Length_1469, SampleTS2_Locus_11692_Transcript_2/7_Confidence_0.333_Length_2130, SampleTS2_Locus_760_Transcript_7/10_Confidence_0.186_Length_1031, Sample6_Locus_13592_Transcript_5/5_Confidence_0.250_Length_879, Sample6_Locus_15604_Transcript_2/4_Confidence_0.400_Length_1416, SampleTS3_Locus_21707_Transcript_1/2_Confidence_0.333_Length_2460, Sample1_Locus_12424_Transcript_3/6_Confidence_0.500_Length_2400, Sample6_Locus_2195_Transcript_14/16_Confidence_0.176_Length_1741, SampleTS3_Locus_714_Transcript_7/14_Confidence_0.087_Length_1194, Sample1_Locus_19449_Transcript_1/1_Confidence_0.000_Length_1704, SampleTS2_Locus_263_Transcript_1/3_Confidence_0.250_Length_2184, Sample3_Locus_1427_Transcript_1/1_Confidence_0.000_Length_746, SampleTS2_Locus_12481_Transcript_10/11_Confidence_0.194_Length_1103, SampleTS1_Locus_27797_Transcript_4/4_Confidence_0.800_Length_878, SampleTS2_Locus_1750_Transcript_17/20_Confidence_0.182_Length_1568, Sample6_Locus_293_Transcript_1/2_Confidence_0.333_Length_875, SampleTS3_Locus_11808_Transcript_9/11_Confidence_0.565_Length_1006, SampleTS1_Locus_8328_Transcript_7/9_Confidence_0.407_Length_1307, SampleTS2_Locus_4114_Transcript_5/7_Confidence_0.267_Length_2080, Sample6_Locus_6489_Transcript_7/13_Confidence_0.200_Length_1232, SampleTS2_Locus_3113_Transcript_1/3_Confidence_0.667_Length_750

**Glycine, serine and threonine metabolism**	map00260	19	ec:2.1.2.1 hydroxymethyltransferase, ec:2.6.1.51 transaminase, ec:2.6.1.52 transaminase, ec:2.7.2.4 kinase, ec:2.7.1.39 kinase, ec:4.2.3.1 synthase, ec:2.6.1.44 transaminase, ec:2.6.1.45 transaminase, ec:1.1.1.103 3dehydrogenase, ec:4.1.2.5 aldolase, ec:2.3.1.29 Cacetyltransferase, ec:1.8.1.4 dehydrogenase, ec:3.1.3.3 phosphatase, ec:1.1.1.1 dehydrogenase, ec:4.2.1.20 synthase, ec:2.6.1.4 transaminase, ec:1.2.1.8 dehydrogenase, ec:4.4.1.1 gammalyase, ec:1.4.3.21 oxidase	2, 1, 1, 1, 1, 2, 6, 1, 3, 2, 1, 1, 1, 5, 3, 3, 1, 2, 1	Sample1_Locus_3932_Transcript_7/11_Confidence_0.400_Length_1725, SampleTS1_Locus_10618_Transcript_3/4_Confidence_0.400_Length_1517, Sample6_Locus_15604_Transcript_2/4_Confidence_0.400_Length_1416, Sample1_Locus_9264_Transcript_1/1_Confidence_0.000_Length_1678, Sample3_Locus_2031_Transcript_1/1_Confidence_0.000_Length_2262, Sample3_Locus_10043_Transcript_1/1_Confidence_0.000_Length_1378, Sample6_Locus_4215_Transcript_12/29_Confidence_0.125_Length_1509, Sample6_Locus_4215_Transcript_14/29_Confidence_0.069_Length_739, Sample6_Locus_2195_Transcript_14/16_Confidence_0.176_Length_1741, SampleTS3_Locus_714_Transcript_7/14_Confidence_0.087_Length_1194, Sample3_Locus_1833_Transcript_4/6_Confidence_0.500_Length_1265, SampleTS1_Locus_13173_Transcript_7/8_Confidence_0.190_Length_747, Sample1_Locus_19449_Transcript_1/1_Confidence_0.000_Length_1704, Sample6_Locus_15604_Transcript_2/4_Confidence_0.400_Length_1416, Sample6_Locus_15604_Transcript_2/4_Confidence_0.400_Length_1416, SampleTS2_Locus_263_Transcript_1/3_Confidence_0.250_Length_2184, SampleTS3_Locus_400_Transcript_6/29_Confidence_0.422_Length_2479, SampleTS1_Locus_4882_Transcript_3/9_Confidence_0.550_Length_1118, Sample6_Locus_6563_Transcript_4/10_Confidence_0.455_Length_1416, SampleTS1_Locus_10618_Transcript_3/4_Confidence_0.400_Length_1517, SampleTS2_Locus_13131_Transcript_7/10_Confidence_0.083_Length_1731, SampleTS3_Locus_4699_Transcript_6/10_Confidence_0.579_Length_1963, Sample1_Locus_18291_Transcript_1/1_Confidence_0.000_Length_647, SampleTS2_Locus_4874_Transcript_6/12_Confidence_0.158_Length_1042, Sample6_Locus_1524_Transcript_26/30_Confidence_0.185_Length_3215, SampleTS2_Locus_263_Transcript_1/3_Confidence_0.250_Length_2184, SampleTS3_Locus_400_Transcript_6/29_Confidence_0.422_Length_2479, SampleTS3_Locus_23264_Transcript_1/3_Confidence_0.500_Length_845, SampleTS3_Locus_9447_Transcript_3/3_Confidence_0.000_Length_886, Sample6_Locus_6882_Transcript_6/8_Confidence_0.429_Length_1475, SampleTS1_Locus_10894_Transcript_1/1_Confidence_0.000_Length_652, Sample6_Locus_2195_Transcript_14/16_Confidence_0.176_Length_1741, SampleTS3_Locus_714_Transcript_7/14_Confidence_0.087_Length_1194, Sample1_Locus_19449_Transcript_1/1_Confidence_0.000_Length_1704, SampleTS1_Locus_5373_Transcript_26/33_Confidence_0.065_Length_1620, Sample6_Locus_5355_Transcript_5/8_Confidence_0.577_Length_1830, SampleTS2_Locus_2923_Transcript_3/4_Confidence_0.333_Length_1277, Sample1_Locus_4359_Transcript_11/14_Confidence_0.314_Length_2540

**Pyrimidine metabolism**	map00240	19	ec:2.7.4.6 kinase, ec:3.5.2.2 hydantoinase, ec:2.7.4.9 kinase, ec:3.6.1.6 diphosphate phosphatase, ec:1.3.5.2 dehydrogenase (quinone), ec:3.6.1.9 diphosphatase, ec:4.1.1.23 decarboxylase, ec:2.7.1.48 kinase, ec:1.17.4.1 reductase, ec:2.1.3.2 carbamoyltrans ferase, ec:2.7.1.21 kinase, ec:4.2.1.70 synthase, ec:2.7.7.6 RNA polymerase, ec:2.7.7.7 DNA polymerase, ec:1.3.1.2 dehydrogenase (NADP+), ec:6.3.5.5 synthase (glutaminehydr olysing), ec:2.4.2.10 phosphoribosylt ransferase, ec:3.5.4.12 deaminase, ec:2.7.4.14 kinase	5, 2, 1, 5, 2, 5, 1, 3, 1, 1, 1, 1, 11, 6, 1, 2, 1, 1, 2	SampleTS1_Locus_4995_Transcript_3/15_Confidence_0.395_Length_904, SampleTS1_Locus_6152_Transcript_2/7_Confidence_0.375_Length_1167, SampleTS1_Locus_1762_Transcript_13/36_Confidence_0.058_Length_819 , Sample1_Locus_8568_Transcript_27/30_Confidence_0.054_Length_2057, Sample1_Locus_24078_Transcript_6/7_Confidence_0.150_Length_1572, SampleTS1_Locus_275_Transcript_59/85_Confidence_0.013_Length_590, Sample6_Locus_10051_Transcript_2/3_Confidence_0.333_Length_1368, Sample6_Locus_661_Transcript_11/15_Confidence_0.057_Length_877, Sample3_Locus_345_Transcript_6/8_Confidence_0.455_Length_1801, Sample6_Locus_7351_Transcript_1/1_Confidence_0.000_Length_1279, SampleTS1_Locus_16626_Transcript_2/3_Confidence_0.500_Length_2047 , SampleTS1_Locus_22896_Transcript_1/1_Confidence_0.000_Length_1681 , Sample3_Locus_734_Transcript_8/12_Confidence_0.176_Length_1222, Sample6_Locus_6815_Transcript_6/12_Confidence_0.385_Length_956, SampleTS2_Locus_1483_Transcript_3/13_Confidence_0.364_Length_1620 , Sample6_Locus_1849_Transcript_4/6_Confidence_0.429_Length_866, Sample3_Locus_345_Transcript_6/8_Confidence_0.455_Length_1801, SampleTS3_Locus_5573_Transcript_4/5_Confidence_0.333_Length_940, Sample3_Locus_10699_Transcript_3/4_Confidence_0.400_Length_957, Sample3_Locus_27182_Transcript_2/3_Confidence_0.250_Length_1010, SampleTS1_Locus_6359_Transcript_7/7_Confidence_0.294_Length_1274, Sample1_Locus_7813_Transcript_4/8_Confidence_0.643_Length_1238, SampleTS2_Locus_12437_Transcript_2/3_Confidence_0.667_Length_1244 , SampleTS1_Locus_1762_Transcript_13/36_Confidence_0.058_Length_819 , SampleTS3_Locus_11402_Transcript_2/3_Confidence_0.333_Length_597, Sample1_Locus_3772_Transcript_1/3_Confidence_0.333_Length_1976, SampleTS3_Locus_6992_Transcript_3/6_Confidence_0.500_Length_986, Sample1_Locus_19788_Transcript_1/1_Confidence_0.333_Length_1516, Sample6_Locus_5179_Transcript_1/5_Confidence_0.400_Length_1830, Sample1_Locus_25958_Transcript_3/6_Confidence_0.286_Length_1845, SampleTS1_Locus_10744_Transcript_1/2_Confidence_0.750_Length_662, Sample6_Locus_7420_Transcript_2/3_Confidence_0.333_Length_873, SampleTS2_Locus_4529_Transcript_4/5_Confidence_0.500_Length_1425, SampleTS1_Locus_8634_Transcript_2/3_Confidence_0.333_Length_1251, Sample6_Locus_18352_Transcript_2/2_Confidence_0.333_Length_583, SampleTS1_Locus_6605_Transcript_10/11_Confidence_0.600_Length_612 , Sample1_Locus_24940_Transcript_2/3_Confidence_0.051_Length_716, Sample3_Locus_2973_Transcript_5/8_Confidence_0.556_Length_3914, Sample6_Locus_5933_Transcript_3/4_Confidence_0.071_Length_879, Sample1_Locus_11973_Transcript_3/4_Confidence_0.500_Length_1566, SampleTS1_Locus_2024_Transcript_1/6_Confidence_0.571_Length_829, Sample3_Locus_5177_Transcript_4/6_Confidence_0.400_Length_1433, SampleTS1_Locus_3621_Transcript_2/4_Confidence_0.143_Length_620, Sample6_Locus_17542_Transcript_6/8_Confidence_0.700_Length_1235, Sample6_Locus_5933_Transcript_3/4_Confidence_0.071_Length_879, SampleTS2_Locus_1483_Transcript_3/13_Confidence_0.364_Length_1620 , SampleTS2_Locus_5848_Transcript_1/1_Confidence_0.000_Length_1345, SampleTS3_Locus_15776_Transcript_1/2_Confidence_0.333_Length_1102 , SampleTS1_Locus_6359_Transcript_7/7_Confidence_0.294_Length_1274, Sample6_Locus_15085_Transcript_1/1_Confidence_0.000_Length_1486, SampleTS1_Locus_6152_Transcript_2/7_Confidence_0.375_Length_1167, Sample1_Locus_24078_Transcript_6/7_Confidence_0.150_Length_1572

**Phenylalanine, tyrosine and tryptophan biosynthesis**	map00400	18	ec:2.7.1.71 kinase, ec:1.4.3.2 oxidase, ec:2.6.1.57 transaminase, ec:2.6.1.58 transaminase, ec:4.1.3.27 synthase, ec:4.2.3.5 synthase, ec:4.2.3.4 synthase, ec:4.2.1.51 dehydratase, ec:2.6.1.78 aminotransferase, ec:2.6.1.79 aminotransferase, ec:4.2.1.91 dehydratase, ec:4.2.1.20 synthase, ec:2.6.1.5 transaminase, ec:2.6.1.9 transaminase, ec:2.6.1.1 transaminase, ec:2.5.1.54 synthase, ec:1.14.16.1 4monooxygenase, ec:5.3.1.24 isomerase	1, 1, 2, 1, 1, 1, 2, 1, 3, 3, 1, 3, 2, 2, 3, 1, 1, 1	SampleTS2_Locus_1705_Transcript_2/5_Confidence_0.667_Length_1103, SampleTS1_Locus_1041_Transcript_1/5_Confidence_0.400_Length_2190, Sample1_Locus_26802_Transcript_1/2_Confidence_0.667_Length_439, Sample6_Locus_13664_Transcript_1/2_Confidence_0.333_Length_862, Sample6_Locus_22798_Transcript_3/3_Confidence_0.250_Length_666, Sample1_Locus_5305_Transcript_3/12_Confidence_0.143_Length_2221, SampleTS2_Locus_10844_Transcript_3/4_Confidence_0.200_Length_1482, Sample6_Locus_15905_Transcript_2/2_Confidence_0.000_Length_483, Sample3_Locus_8753_Transcript_3/7_Confidence_0.053_Length_1045, Sample6_Locus_164_Transcript_78/296_Confidence_0.027_Length_1952, Sample1_Locus_26802_Transcript_1/2_Confidence_0.667_Length_439, Sample6_Locus_13664_Transcript_1/2_Confidence_0.333_Length_862, SampleTS2_Locus_6646_Transcript_2/2_Confidence_0.333_Length_1520, Sample1_Locus_26802_Transcript_1/2_Confidence_0.667_Length_439, Sample6_Locus_13664_Transcript_1/2_Confidence_0.333_Length_862, SampleTS2_Locus_6646_Transcript_2/2_Confidence_0.333_Length_1520, Sample6_Locus_164_Transcript_78/296_Confidence_0.027_Length_1952, SampleTS3_Locus_9447_Transcript_3/3_Confidence_0.000_Length_886, Sample6_Locus_6882_Transcript_6/8_Confidence_0.429_Length_1475, SampleTS1_Locus_10894_Transcript_1/1_Confidence_0.000_Length_652, Sample1_Locus_26802_Transcript_1/2_Confidence_0.667_Length_439, Sample6_Locus_13664_Transcript_1/2_Confidence_0.333_Length_862, Sample1_Locus_26802_Transcript_1/2_Confidence_0.667_Length_439, Sample6_Locus_13664_Transcript_1/2_Confidence_0.333_Length_862, Sample1_Locus_26802_Transcript_1/2_Confidence_0.667_Length_439, Sample6_Locus_13664_Transcript_1/2_Confidence_0.333_Length_862, SampleTS2_Locus_6646_Transcript_2/2_Confidence_0.333_Length_1520, Sample3_Locus_8990_Transcript_7/9_Confidence_0.091_Length_1811, Sample3_Locus_2321_Transcript_7/9_Confidence_0.414_Length_1007, SampleTS1_Locus_12931_Transcript_3/3_Confidence_0.200_Length_1113

**Carbon fixation pathways in prokaryotes**	map00720	18	ec:6.4.1.2 carboxylase, ec:6.4.1.3 carboxylase, ec:1.3.5.1 dehydrogenase, ec:4.1.1.31 carboxylase, ec:4.2.1.3 hydratase, ec:4.2.1.2 hydratase, ec:3.5.4.9 cyclohydrolase, ec:6.2.1.5 ligase (ADPforming), ec:6.2.1.1 ligase, ec:5.4.99.2 mutase, ec:4.2.1.17 hydratase, ec:2.7.9.1 phosphate dikinase, ec:2.3.1.9 Cacetyltransferase, ec:1.1.1.37 dehydrogenase, ec:1.1.1.35 dehydrogenase, ec:1.3.1.6 reductase (NADH), ec:1.5.1.5 dehydrogenase (NADP+), ec:2.3.3.8 citrate synthase	2, 2, 1, 1, 1, 2, 1, 2, 1, 1, 2, 3, 1, 1, 3, 2, 1, 1	Sample3_Locus_8920_Transcript_2/3_Confidence_0.333_Length_2038, SampleTS1_Locus_1155_Transcript_11/16_Confidence_0.625_Length_2240, SampleTS1_Locus_1968_Transcript_1/3_Confidence_0.400_Length_530, SampleTS1_Locus_1155_Transcript_11/16_Confidence_0.625_Length_2240, SampleTS2_Locus_54_Transcript_4/10_Confidence_0.083_Length_1374, Sample3_Locus_7378_Transcript_3/6_Confidence_0.417_Length_3416, SampleTS2_Locus_7424_Transcript_6/8_Confidence_0.286_Length_3139, Sample3_Locus_2252_Transcript_9/13_Confidence_0.647_Length_1708, Sample6_Locus_1413_Transcript_3/3_Confidence_0.000_Length_1802, Sample6_Locus_4997_Transcript_3/6_Confidence_0.429_Length_1455, Sample3_Locus_2152_Transcript_2/3_Confidence_0.333_Length_1481, SampleTS3_Locus_6340_Transcript_1/1_Confidence_0.000_Length_1077, SampleTS3_Locus_456_Transcript_2/6_Confidence_0.200_Length_2075, Sample1_Locus_12424_Transcript_3/6_Confidence_0.500_Length_2400, SampleTS1_Locus_789_Transcript_9/11_Confidence_0.238_Length_2300, Sample3_Locus_5282_Transcript_2/7_Confidence_0.700_Length_1266, SampleTS1_Locus_793_Transcript_3/3_Confidence_0.000_Length_1036, Sample6_Locus_2229_Transcript_3/3_Confidence_0.000_Length_1460, SampleTS2_Locus_3926_Transcript_9/12_Confidence_0.038_Length_2167, SampleTS2_Locus_1750_Transcript_17/20_Confidence_0.182_Length_1568, Sample6_Locus_293_Transcript_1/2_Confidence_0.333_Length_875, SampleTS1_Locus_789_Transcript_9/11_Confidence_0.238_Length_2300, SampleTS3_Locus_1827_Transcript_3/3_Confidence_0.000_Length_1033, SampleTS3_Locus_11808_Transcript_9/11_Confidence_0.565_Length_1006, SampleTS3_Locus_3772_Transcript_3/6_Confidence_0.130_Length_1377, SampleTS1_Locus_13834_Transcript_7/9_Confidence_0.143_Length_2053, Sample6_Locus_4997_Transcript_3/6_Confidence_0.429_Length_1455, SampleTS3_Locus_443_Transcript_4/7_Confidence_0.429_Length_1718

**Propanoate metabolism**	map00640	18	ec:6.4.1.2 carboxylase, ec:2.6.1.18 transaminase, ec:2.6.1.19 transaminase, ec:6.4.1.3 carboxylase, ec:1.2.1.27 dehydrogenase (CoAacylating), ec:6.2.1.5 ligase (ADPforming), ec:6.2.1.4 ligase (GDPforming), ec:6.2.1.1 ligase, ec:1.2.1.18 dehydrogenase (acetylating), ec:2.3.1.54 Cacetyltransferase, ec:5.4.99.2 mutase, ec:2.8.3.8 CoAtransferase, ec:1.8.1.4 dehydrogenase, ec:4.2.1.17 hydratase, ec:1.1.1.77 reductase, ec:1.1.1.202 dehydrogenase, ec:2.3.1.9 Cacetyltransferase, ec:1.2.4.4 dehydrogenase (2methylpropanoyltransferring)	2, 1, 1, 2, 1, 2, 2, 1, 1, 3, 1, 1, 1, 2, 1, 1, 1, 2	Sample3_Locus_8920_Transcript_2/3_Confidence_0.333_Length_2038, SampleTS1_Locus_1155_Transcript_11/16_Confidence_0.625_Length_2240, Sample3_Locus_1833_Transcript_4/6_Confidence_0.500_Length_1265, SampleTS1_Locus_13173_Transcript_7/8_Confidence_0.190_Length_747, SampleTS1_Locus_1968_Transcript_1/3_Confidence_0.400_Length_530, SampleTS1_Locus_1155_Transcript_11/16_Confidence_0.625_Length_2240, SampleTS3_Locus_23038_Transcript_2/3_Confidence_0.333_Length_1864, Sample3_Locus_2152_Transcript_2/3_Confidence_0.333_Length_1481, SampleTS3_Locus_6340_Transcript_1/1_Confidence_0.000_Length_1077, Sample3_Locus_2152_Transcript_2/3_Confidence_0.333_Length_1481, SampleTS3_Locus_6340_Transcript_1/1_Confidence_0.000_Length_1077, SampleTS3_Locus_456_Transcript_2/6_Confidence_0.200_Length_2075, SampleTS3_Locus_23038_Transcript_2/3_Confidence_0.333_Length_1864, Sample6_Locus_961_Transcript_2/3_Confidence_0.250_Length_1478, Sample1_Locus_27983_Transcript_6/13_Confidence_0.294_Length_763, SampleTS2_Locus_20366_Transcript_1/7_Confidence_0.273_Length_670, Sample1_Locus_12424_Transcript_3/6_Confidence_0.500_Length_2400, SampleTS2_Locus_1750_Transcript_17/20_Confidence_0.182_Length_1568, SampleTS3_Locus_4699_Transcript_6/10_Confidence_0.579_Length_1963, SampleTS1_Locus_789_Transcript_9/11_Confidence_0.238_Length_2300, Sample3_Locus_5282_Transcript_2/7_Confidence_0.700_Length_1266, SampleTS2_Locus_263_Transcript_1/3_Confidence_0.250_Length_2184, SampleTS2_Locus_263_Transcript_1/3_Confidence_0.250_Length_2184, SampleTS2_Locus_1750_Transcript_17/20_Confidence_0.182_Length_1568, Sample1_Locus_24165_Transcript_4/4_Confidence_0.444_Length_605, SampleTS2_Locus_9103_Transcript_1/2_Confidence_0.667_Length_685

**Carbon fixation in photosynthetic organisms**	map00710	17	ec:4.1.2.13 aldolase, ec:4.1.1.49 carboxykinase (ATP), ec:4.1.1.39 carboxylase, ec:4.1.1.31 carboxylase, ec:2.2.1.1 glycolaldehydetransferase, ec:1.2.1.59 dehydrogenase (NAD(P)+) (phosphorylating), ec:2.7.1.19 phosphopentokinase, ec:5.1.3.1 3epimerase, ec:3.1.3.11 hexose diphosphatase, ec:1.2.1.13 dehydrogenase (NADP+) (phosphorylating), ec:1.2.1.12 dehydrogenase (phosphorylating), ec:5.3.1.6 isomerase, ec:2.6.1.2 transaminase, ec:2.6.1.1 transaminase, ec:2.7.9.1 phosphate dikinase, ec:1.1.1.37 dehydrogenase, ec:1.1.1.40 dehydrogenase (oxaloacetatedecarboxylating) (NADP+)	2, 1, 1, 1, 1, 3, 1, 1, 2, 1, 5, 2, 3, 3, 3, 1, 2	Sample3_Locus_324_Transcript_3/17_Confidence_0.222_Length_1711, Sample6_Locus_28252_Transcript_2/3_Confidence_0.500_Length_808, Sample6_Locus_11174_Transcript_1/1_Confidence_0.000_Length_1998, SampleTS1_Locus_4324_Transcript_3/4_Confidence_0.286_Length_1536, Sample3_Locus_7378_Transcript_3/6_Confidence_0.417_Length_3416, SampleTS1_Locus_16129_Transcript_1/1_Confidence_0.000_Length_1960 , SampleTS2_Locus_819_Transcript_16/18_Confidence_0.250_Length_1000 , SampleTS2_Locus_12618_Transcript_4/6_Confidence_0.571_Length_1272 , Sample6_Locus_997_Transcript_9/17_Confidence_0.200_Length_950, SampleTS2_Locus_12437_Transcript_2/3_Confidence_0.667_Length_1244 , Sample3_Locus_3334_Transcript_1/5_Confidence_0.400_Length_1030, Sample3_Locus_10262_Transcript_4/5_Confidence_0.062_Length_1337, Sample6_Locus_2283_Transcript_7/17_Confidence_0.225_Length_1818, Sample1_Locus_7420_Transcript_10/14_Confidence_0.154_Length_1346, SampleTS2_Locus_819_Transcript_16/18_Confidence_0.250_Length_1000 , Sample1_Locus_7420_Transcript_10/14_Confidence_0.154_Length_1346, SampleTS2_Locus_12618_Transcript_4/6_Confidence_0.571_Length_1272 , Sample6_Locus_997_Transcript_9/17_Confidence_0.200_Length_950, Sample1_Locus_393_Transcript_6/7_Confidence_0.556_Length_1466, SampleTS1_Locus_27877_Transcript_2/3_Confidence_0.333_Length_987, Sample3_Locus_9475_Transcript_2/2_Confidence_0.000_Length_1080, Sample6_Locus_2195_Transcript_14/16_Confidence_0.176_Length_1741, SampleTS3_Locus_714_Transcript_7/14_Confidence_0.087_Length_1194, Sample1_Locus_19449_Transcript_1/1_Confidence_0.000_Length_1704, Sample1_Locus_26802_Transcript_1/2_Confidence_0.667_Length_439, Sample6_Locus_13664_Transcript_1/2_Confidence_0.333_Length_862, SampleTS2_Locus_6646_Transcript_2/2_Confidence_0.333_Length_1520, SampleTS1_Locus_793_Transcript_3/3_Confidence_0.000_Length_1036, Sample6_Locus_2229_Transcript_3/3_Confidence_0.000_Length_1460, SampleTS2_Locus_3926_Transcript_9/12_Confidence_0.038_Length_2167 , Sample6_Locus_293_Transcript_1/2_Confidence_0.333_Length_875, Sample3_Locus_24109_Transcript_4/4_Confidence_0.000_Length_2026, SampleTS2_Locus_3140_Transcript_18/19_Confidence_0.323_Length_745

**Glycerophospholipid metabolism**	map00564	16	ec:2.7.1.82 kinase, ec:2.7.1.32 kinase, ec:4.2.3.2 phospholyase, ec:2.3.1.15 1Oacyltransferase, ec:3.1.3.27 phosphatidylglycerol phosphate phosphatase, ec:3.1.1.4 A2, ec:3.1.1.5 lecithinase B, ec:2.3.1.51 Oacyltransferase, ec:2.3.1.42 Oacyltransferase, ec:3.1.3.4 phosphatase, ec:1.1.1.8 dehydrogenase (NAD+), ec:2.1.1.103 Nmethyltransferase, ec:1.1.1.94 dehydrogenase [NAD(P)+], ec:2.7.7.14 cytidylyltransferase, ec:2.7.7.15 cytidylyltransferase, ec:1.1.5.3 dehydrogenase	1, 1, 1, 4, 1, 4, 1, 1, 3, 2, 1, 2, 1, 1, 1, 1	Sample3_Locus_24294_Transcript_4/4_Confidence_0.250_Length_1046, Sample3_Locus_24294_Transcript_4/4_Confidence_0.250_Length_1046, SampleTS1_Locus_13173_Transcript_7/8_Confidence_0.190_Length_747, SampleTS1_Locus_7733_Transcript_3/6_Confidence_0.615_Length_1523, Sample6_Locus_335_Transcript_1/5_Confidence_0.214_Length_2401, Sample1_Locus_24278_Transcript_1/2_Confidence_0.667_Length_689, SampleTS2_Locus_5399_Transcript_4/5_Confidence_0.034_Length_1249, SampleTS3_Locus_5284_Transcript_10/12_Confidence_0.450_Length_1002, SampleTS1_Locus_587_Transcript_4/24_Confidence_0.067_Length_950, SampleTS2_Locus_23527_Transcript_1/1_Confidence_0.000_Length_1031, SampleTS1_Locus_11100_Transcript_2/5_Confidence_0.286_Length_1260, SampleTS3_Locus_8220_Transcript_1/4_Confidence_0.667_Length_1081, SampleTS3_Locus_4982_Transcript_5/18_Confidence_0.148_Length_763, SampleTS1_Locus_7733_Transcript_3/6_Confidence_0.615_Length_1523, Sample1_Locus_24278_Transcript_1/2_Confidence_0.667_Length_689, SampleTS2_Locus_5399_Transcript_4/5_Confidence_0.034_Length_1249, Sample6_Locus_335_Transcript_1/5_Confidence_0.214_Length_2401, Sample1_Locus_2193_Transcript_40/42_Confidence_0.262_Length_1445, SampleTS2_Locus_23786_Transcript_1/1_Confidence_0.000_Length_866, Sample3_Locus_18209_Transcript_1/3_Confidence_0.667_Length_1443, SampleTS1_Locus_8588_Transcript_5/7_Confidence_0.286_Length_1322, SampleTS2_Locus_2805_Transcript_2/4_Confidence_0.400_Length_1933, Sample3_Locus_18209_Transcript_1/3_Confidence_0.667_Length_1443, SampleTS3_Locus_256_Transcript_7/9_Confidence_0.444_Length_1142, SampleTS3_Locus_256_Transcript_7/9_Confidence_0.444_Length_1142, SampleTS3_Locus_447_Transcript_3/8_Confidence_0.444_Length_1795

**Valine, leucine and isoleucine degradation**	map00280	15	ec:2.6.1.18 transaminase, ec:6.4.1.3 carboxylase, ec:1.4.3.2 oxidase, ec:1.2.1.27 dehydrogenase (CoAacylating), ec:2.6.1.40 transaminase, ec:2.6.1.42 transaminase, ec:2.3.1.16 Cacyltransferase, ec:5.4.99.2 mutase, ec:1.8.1.4 dehydrogenase, ec:4.2.1.17 hydratase, ec:1.2.1.3 dehydrogenase (NAD+), ec:2.3.1.9 Cacetyltransferase, ec:1.1.1.35 dehydrogenase, ec:1.1.1.31 dehydrogenase, ec:1.2.4.4 dehydrogenase (2methylpropanoyltransferring)	1, 2, 1, 1, 1, 2, 1, 1, 1, 2, 6, 1, 3, 1, 2	Sample3_Locus_1833_Transcript_4/6_Confidence_0.500_Length_1265, SampleTS1_Locus_1968_Transcript_1/3_Confidence_0.400_Length_530, SampleTS1_Locus_1155_Transcript_11/16_Confidence_0.625_Length_2240, SampleTS1_Locus_1041_Transcript_1/5_Confidence_0.400_Length_2190, SampleTS3_Locus_23038_Transcript_2/3_Confidence_0.333_Length_1864, Sample3_Locus_1833_Transcript_4/6_Confidence_0.500_Length_1265, Sample1_Locus_18777_Transcript_2/4_Confidence_0.923_Length_1416, Sample3_Locus_5952_Transcript_4/4_Confidence_0.000_Length_2586, SampleTS2_Locus_1750_Transcript_17/20_Confidence_0.182_Length_1568, Sample1_Locus_12424_Transcript_3/6_Confidence_0.500_Length_2400, SampleTS3_Locus_4699_Transcript_6/10_Confidence_0.579_Length_1963, SampleTS1_Locus_789_Transcript_9/11_Confidence_0.238_Length_2300, Sample3_Locus_5282_Transcript_2/7_Confidence_0.700_Length_1266, SampleTS3_Locus_23038_Transcript_2/3_Confidence_0.333_Length_1864, SampleTS2_Locus_4874_Transcript_6/12_Confidence_0.158_Length_1042, Sample3_Locus_23176_Transcript_2/3_Confidence_0.333_Length_1282, SampleTS1_Locus_5373_Transcript_26/33_Confidence_0.065_Length_1620, Sample6_Locus_12695_Transcript_1/2_Confidence_0.333_Length_2218, SampleTS3_Locus_23264_Transcript_1/3_Confidence_0.500_Length_845, SampleTS2_Locus_1750_Transcript_17/20_Confidence_0.182_Length_1568, SampleTS1_Locus_789_Transcript_9/11_Confidence_0.238_Length_2300, SampleTS3_Locus_1827_Transcript_3/3_Confidence_0.000_Length_1033, SampleTS3_Locus_11808_Transcript_9/11_Confidence_0.565_Length_1006, Sample6_Locus_10552_Transcript_5/5_Confidence_0.000_Length_1013, Sample1_Locus_24165_Transcript_4/4_Confidence_0.444_Length_605, SampleTS2_Locus_9103_Transcript_1/2_Confidence_0.667_Length_685

**AminoacyltRNA biosynthesis**	map00970	15	ec:6.1.1.6 ligase, ec:6.1.1.7 ligase, ec:6.1.1.9 ligase, ec:6.1.1.2 ligase, ec:6.1.1.3 ligase, ec:6.1.1.4 ligase, ec:6.1.1.5 ligase, ec:6.1.1.1 ligase, ec:6.1.1.17 ligase, ec:6.1.1.14 ligase, ec:6.1.1.15 ligase, ec:6.1.1.10 ligase, ec:6.1.1.11 ligase, ec:6.1.1.21 ligase, ec:6.1.1.20 ligase	1, 1, 1, 1, 1, 1, 4, 1, 2, 1, 2, 1, 1, 1, 2	Sample3_Locus_4849_Transcript_2/3_Confidence_0.333_Length_1992, Sample1_Locus_12667_Transcript_5/9_Confidence_0.391_Length_935, Sample1_Locus_8266_Transcript_6/7_Confidence_0.333_Length_1182, Sample3_Locus_7465_Transcript_6/8_Confidence_0.455_Length_2812, SampleTS3_Locus_15910_Transcript_1/1_Confidence_0.000_Length_2059, Sample3_Locus_4481_Transcript_6/9_Confidence_0.333_Length_3617, Sample6_Locus_10979_Transcript_4/6_Confidence_0.067_Length_821, SampleTS1_Locus_1930_Transcript_7/12_Confidence_0.552_Length_1752, SampleTS1_Locus_2851_Transcript_1/3_Confidence_0.667_Length_1262, Sample1_Locus_16131_Transcript_3/4_Confidence_0.667_Length_1151, Sample1_Locus_8266_Transcript_6/7_Confidence_0.333_Length_1182, SampleTS1_Locus_19795_Transcript_1/3_Confidence_0.333_Length_2365, SampleTS1_Locus_12215_Transcript_1/1_Confidence_0.000_Length_1974, SampleTS1_Locus_18025_Transcript_1/1_Confidence_0.000_Length_511, SampleTS1_Locus_19795_Transcript_1/3_Confidence_0.333_Length_2365, SampleTS1_Locus_12215_Transcript_1/1_Confidence_0.000_Length_1974, SampleTS1_Locus_1827_Transcript_15/16_Confidence_0.043_Length_2672, SampleTS2_Locus_13321_Transcript_3/3_Confidence_0.000_Length_1479, Sample3_Locus_1944_Transcript_12/13_Confidence_0.053_Length_1388, Sample3_Locus_2323_Transcript_7/7_Confidence_0.100_Length_1063, Sample1_Locus_1466_Transcript_4/5_Confidence_0.286_Length_2081

**Citrate cycle (TCA cycle)**	map00020	15	ec:2.3.1.61 succinyltransferase, ec:4.1.1.49 carboxykinase (ATP), ec:1.3.5.1 dehydrogenase, ec:4.1.1.32 carboxykinase (GTP), ec:4.2.1.3 hydratase, ec:4.2.1.2 hydratase, ec:6.2.1.5 ligase (ADPforming), ec:6.2.1.4 ligase (GDPforming), ec:2.3.1.12 acetyltransferase, ec:1.8.1.4 dehydrogenase, ec:1.1.1.37 dehydrogenase, ec:2.3.3.8 citrate synthase, ec:2.3.3.1 (Si)synthase, ec:1.2.4.2 dehydrogenase (succinyltransferring), ec:1.2.4.1 dehydrogenase (acetyltransferring)	1, 1, 1, 1, 1, 2, 2, 2, 2, 1, 1, 1, 3, 1, 2	Sample3_Locus_4770_Transcript_12/16_Confidence_0.420_Length_1753, Sample6_Locus_11174_Transcript_1/1_Confidence_0.000_Length_1998, SampleTS2_Locus_54_Transcript_4/10_Confidence_0.083_Length_1374, Sample6_Locus_11174_Transcript_1/1_Confidence_0.000_Length_1998, SampleTS2_Locus_7424_Transcript_6/8_Confidence_0.286_Length_3139, Sample3_Locus_2252_Transcript_9/13_Confidence_0.647_Length_1708, Sample6_Locus_1413_Transcript_3/3_Confidence_0.000_Length_1802, Sample3_Locus_2152_Transcript_2/3_Confidence_0.333_Length_1481, SampleTS3_Locus_6340_Transcript_1/1_Confidence_0.000_Length_1077, Sample3_Locus_2152_Transcript_2/3_Confidence_0.333_Length_1481, SampleTS3_Locus_6340_Transcript_1/1_Confidence_0.000_Length_1077, SampleTS2_Locus_8836_Transcript_1/2_Confidence_0.667_Length_1729, SampleTS3_Locus_5316_Transcript_1/2_Confidence_0.750_Length_1843, SampleTS3_Locus_4699_Transcript_6/10_Confidence_0.579_Length_1963, Sample6_Locus_293_Transcript_1/2_Confidence_0.333_Length_875, SampleTS3_Locus_443_Transcript_4/7_Confidence_0.429_Length_1718, SampleTS2_Locus_4114_Transcript_5/7_Confidence_0.267_Length_2080, Sample6_Locus_6489_Transcript_7/13_Confidence_0.200_Length_1232, SampleTS2_Locus_3113_Transcript_1/3_Confidence_0.667_Length_750, SampleTS3_Locus_20806_Transcript_1/1_Confidence_0.000_Length_3106, Sample3_Locus_1003_Transcript_1/3_Confidence_0.750_Length_1416, SampleTS2_Locus_9103_Transcript_1/2_Confidence_0.667_Length_685

**Porphyrin and chlorophyll metabolism**	map00860	14	ec:3.1.3.73 phosphatase, ec:4.1.1.37 decarboxylase, ec:4.99.1.1 ferrochelatase, ec:1.1.1.294 b reductase, ec:2.1.1.11 protoporphyrin IX methyltransferase, ec:2.5.1.62 synthase, ec:1.14.13.122 oxygenase, ec:5.4.3.8 2,1aminomutase, ec:1.14.13.81 IX monomethyl ester (oxidative) cyclase, ec:6.1.1.17 ligase, ec:2.4.1.17 1naphthol glucuronyltransferase, ec:1.3.3.4 oxidase, ec:1.3.3.3 oxidase, ec:6.6.1.1 chelatase	1, 2, 2, 2, 2, 1, 1, 1, 1, 2, 3, 1, 2, 1	SampleTS3_Locus_12636_Transcript_2/2_Confidence_0.333_Length_583, Sample3_Locus_1663_Transcript_2/3_Confidence_0.333_Length_1482, Sample1_Locus_3190_Transcript_1/2_Confidence_0.667_Length_1501, Sample6_Locus_3528_Transcript_1/1_Confidence_0.000_Length_1092, Sample3_Locus_2162_Transcript_1/1_Confidence_0.000_Length_1682, Sample1_Locus_18339_Transcript_2/2_Confidence_0.250_Length_1368, SampleTS1_Locus_13725_Transcript_2/7_Confidence_0.333_Length_1080, SampleTS2_Locus_975_Transcript_12/17_Confidence_0.385_Length_803, Sample3_Locus_1732_Transcript_10/14_Confidence_0.346_Length_743, SampleTS1_Locus_7393_Transcript_3/4_Confidence_0.200_Length_1678, SampleTS3_Locus_17019_Transcript_1/1_Confidence_0.000_Length_1129, SampleTS1_Locus_18338_Transcript_1/3_Confidence_0.667_Length_1638, Sample1_Locus_24667_Transcript_1/1_Confidence_0.000_Length_1383, SampleTS1_Locus_19795_Transcript_1/3_Confidence_0.333_Length_2365, SampleTS1_Locus_12215_Transcript_1/1_Confidence_0.000_Length_1974, Sample6_Locus_9455_Transcript_3/4_Confidence_0.200_Length_1370, SampleTS1_Locus_4303_Transcript_9/11_Confidence_0.167_Length_1464, Sample6_Locus_8395_Transcript_14/17_Confidence_0.508_Length_1568, contig00106, SampleTS1_Locus_5260_Transcript_12/17_Confidence_0.303_Length_1209, Sample3_Locus_4668_Transcript_5/5_Confidence_0.000_Length_1437, Sample1_Locus_26944_Transcript_1/1_Confidence_0.000_Length_1423

**Butanoate metabolism**	map00650	14	ec:2.6.1.19 transaminase, ec:1.3.5.1 dehydrogenase, ec:2.2.1.6 synthase, ec:5.1.2.3 epimerase, ec:1.2.1.10 dehydrogenase (acetylating), ec:4.1.1.15 decarboxylase, ec:2.3.1.54 Cacetyltransferase, ec:1.1.1.157 dehydrogenase, ec:2.8.3.8 CoAtransferase, ec:4.2.1.17 hydratase, ec:2.3.1.9 Cacetyltransferase, ec:1.1.1.36 reductase, ec:1.1.1.35 dehydrogenase, ec:1.1.99.2 dehydrogenase	1, 1, 1, 1, 2, 1, 3, 1, 1, 2, 1, 1, 3, 1	SampleTS1_Locus_13173_Transcript_7/8_Confidence_0.190_Length_747, SampleTS2_Locus_54_Transcript_4/10_Confidence_0.083_Length_1374, Sample3_Locus_4596_Transcript_3/6_Confidence_0.400_Length_1157, SampleTS1_Locus_789_Transcript_9/11_Confidence_0.238_Length_2300, SampleTS3_Locus_400_Transcript_6/29_Confidence_0.422_Length_2479, Sample6_Locus_1524_Transcript_26/30_Confidence_0.185_Length_3215, SampleTS3_Locus_5084_Transcript_15/17_Confidence_0.206_Length_1751, Sample6_Locus_961_Transcript_2/3_Confidence_0.250_Length_1478, Sample1_Locus_27983_Transcript_6/13_Confidence_0.294_Length_763, SampleTS2_Locus_20366_Transcript_1/7_Confidence_0.273_Length_670, SampleTS3_Locus_1827_Transcript_3/3_Confidence_0.000_Length_1033, SampleTS2_Locus_1750_Transcript_17/20_Confidence_0.182_Length_1568, SampleTS1_Locus_789_Transcript_9/11_Confidence_0.238_Length_2300, Sample3_Locus_5282_Transcript_2/7_Confidence_0.700_Length_1266, SampleTS2_Locus_1750_Transcript_17/20_Confidence_0.182_Length_1568, SampleTS3_Locus_11808_Transcript_9/11_Confidence_0.565_Length_1006, SampleTS1_Locus_789_Transcript_9/11_Confidence_0.238_Length_2300, SampleTS3_Locus_1827_Transcript_3/3_Confidence_0.000_Length_1033, SampleTS3_Locus_11808_Transcript_9/11_Confidence_0.565_Length_1006, Sample6_Locus_3300_Transcript_2/5_Confidence_0.700_Length_1810

**Phenylalanine metabolism**	map00360	14	ec:1.4.3.2 oxidase, ec:2.6.1.57 transaminase, ec:2.6.1.58 transaminase, ec:1.13.11.27 dioxygenase, ec:1.2.1.10 dehydrogenase (acetylating), ec:1.1.1.157 dehydrogenase, ec:2.6.1.5 transaminase, ec:2.6.1.9 transaminase, ec:2.6.1.1 transaminase, ec:4.2.1.17 hydratase, ec:1.2.1.5 dehydrogenase [NAD(P)+], ec:1.14.16.1 4monooxygenase, ec:3.5.1.4 acylamidase, ec:1.4.3.21 oxidase	1, 2, 1, 3, 2, 1, 2, 2, 3, 2, 1, 1, 2, 1	SampleTS1_Locus_1041_Transcript_1/5_Confidence_0.400_Length_2190, Sample1_Locus_26802_Transcript_1/2_Confidence_0.667_Length_439, Sample6_Locus_13664_Transcript_1/2_Confidence_0.333_Length_862, Sample6_Locus_22798_Transcript_3/3_Confidence_0.250_Length_666, SampleTS1_Locus_605_Transcript_11/25_Confidence_0.258_Length_787, SampleTS2_Locus_361_Transcript_3/4_Confidence_0.750_Length_304, SampleTS1_Locus_605_Transcript_3/25_Confidence_0.161_Length_805, SampleTS3_Locus_400_Transcript_6/29_Confidence_0.422_Length_2479, Sample6_Locus_1524_Transcript_26/30_Confidence_0.185_Length_3215, SampleTS3_Locus_1827_Transcript_3/3_Confidence_0.000_Length_1033, Sample1_Locus_26802_Transcript_1/2_Confidence_0.667_Length_439, Sample6_Locus_13664_Transcript_1/2_Confidence_0.333_Length_862, Sample1_Locus_26802_Transcript_1/2_Confidence_0.667_Length_439, Sample6_Locus_13664_Transcript_1/2_Confidence_0.333_Length_862, Sample1_Locus_26802_Transcript_1/2_Confidence_0.667_Length_439, Sample6_Locus_13664_Transcript_1/2_Confidence_0.333_Length_862, SampleTS2_Locus_6646_Transcript_2/2_Confidence_0.333_Length_1520, SampleTS1_Locus_789_Transcript_9/11_Confidence_0.238_Length_2300, Sample3_Locus_5282_Transcript_2/7_Confidence_0.700_Length_1266, SampleTS1_Locus_5373_Transcript_26/33_Confidence_0.065_Length_1620, Sample3_Locus_2321_Transcript_7/9_Confidence_0.414_Length_1007, Sample6_Locus_14763_Transcript_11/12_Confidence_0.182_Length_1117, Sample6_Locus_19655_Transcript_1/3_Confidence_0.667_Length_978, Sample1_Locus_4359_Transcript_11/14_Confidence_0.314_Length_2540

**Tryptophan metabolism**	map00380	13	ec:1.4.3.2 oxidase, ec:1.11.1.6 equilase, ec:3.5.5.1 acetonitrilase, ec:4.2.1.84 hydratase, ec:2.6.1.7 transaminase, ec:4.2.1.17 hydratase, ec:1.2.1.3 dehydrogenase (NAD+), ec:2.3.1.9 Cacetyltransferase, ec:1.1.1.35 dehydrogenase, ec:3.2.1.147 myrosinase, ec:1.2.4.2 dehydrogenase (succinyltransferring), ec:1.14.16.4 5monooxygenase, ec:3.5.1.4 acylamidase	1, 3, 1, 1, 1, 2, 6, 1, 3, 1, 1, 1, 2	SampleTS1_Locus_1041_Transcript_1/5_Confidence_0.400_Length_2190, SampleTS2_Locus_11692_Transcript_2/7_Confidence_0.333_Length_2130, SampleTS2_Locus_760_Transcript_7/10_Confidence_0.186_Length_1031, Sample6_Locus_13592_Transcript_5/5_Confidence_0.250_Length_879, Sample3_Locus_27182_Transcript_2/3_Confidence_0.250_Length_1010, Sample3_Locus_12929_Transcript_1/1_Confidence_0.000_Length_1898, Sample6_Locus_22798_Transcript_3/3_Confidence_0.250_Length_666, SampleTS1_Locus_789_Transcript_9/11_Confidence_0.238_Length_2300, Sample3_Locus_5282_Transcript_2/7_Confidence_0.700_Length_1266, SampleTS3_Locus_23038_Transcript_2/3_Confidence_0.333_Length_1864, SampleTS2_Locus_4874_Transcript_6/12_Confidence_0.158_Length_1042, Sample3_Locus_23176_Transcript_2/3_Confidence_0.333_Length_1282, SampleTS1_Locus_5373_Transcript_26/33_Confidence_0.065_Length_1620, Sample6_Locus_12695_Transcript_1/2_Confidence_0.333_Length_2218, SampleTS3_Locus_23264_Transcript_1/3_Confidence_0.500_Length_845, SampleTS2_Locus_1750_Transcript_17/20_Confidence_0.182_Length_1568, SampleTS1_Locus_789_Transcript_9/11_Confidence_0.238_Length_2300, SampleTS3_Locus_1827_Transcript_3/3_Confidence_0.000_Length_1033, SampleTS3_Locus_11808_Transcript_9/11_Confidence_0.565_Length_1006, SampleTS2_Locus_9286_Transcript_1/1_Confidence_0.000_Length_2119, SampleTS3_Locus_20806_Transcript_1/1_Confidence_0.000_Length_3106, Sample3_Locus_2321_Transcript_7/9_Confidence_0.414_Length_1007, Sample6_Locus_14763_Transcript_11/12_Confidence_0.182_Length_1117, Sample6_Locus_19655_Transcript_1/3_Confidence_0.667_Length_978

**Fructose and mannose metabolism**	map00051	13	ec:2.7.1.105 phosphofructokinase 2, ec:4.1.2.13 aldolase, ec:3.1.3.46 2phosphatase, ec:2.7.1.1 hexokinase type IV glucokinase, ec:2.7.1.4 fructokinase (phosphorylating), ec:3.1.3.11 hexose diphosphatase, ec:3.6.1.21 diphosphatase, ec:5.3.1.6 isomerase, ec:2.7.7.22 guanylyltransferase (GDP), ec:1.1.1.67 2dehydrogenase, ec:5.4.2.8 mannose phosphomutase, ec:1.1.1.14 2dehydrogenase, ec:1.1.1.21 reductase	1, 2, 1, 1, 1, 2, 4, 2, 1, 1, 1, 1, 2	Sample6_Locus_11496_Transcript_4/7_Confidence_0.250_Length_1518, Sample3_Locus_324_Transcript_3/17_Confidence_0.222_Length_1711, Sample6_Locus_28252_Transcript_2/3_Confidence_0.500_Length_808, Sample6_Locus_11496_Transcript_4/7_Confidence_0.250_Length_1518, Sample6_Locus_5095_Transcript_5/8_Confidence_0.300_Length_1241, Sample6_Locus_5095_Transcript_5/8_Confidence_0.300_Length_1241, Sample3_Locus_10262_Transcript_4/5_Confidence_0.062_Length_1337, Sample6_Locus_2283_Transcript_7/17_Confidence_0.225_Length_1818, Sample3_Locus_345_Transcript_6/8_Confidence_0.455_Length_1801, SampleTS2_Locus_5310_Transcript_3/15_Confidence_0.167_Length_1115, Sample3_Locus_734_Transcript_8/12_Confidence_0.176_Length_1222, SampleTS3_Locus_5794_Transcript_5/6_Confidence_0.125_Length_1722, SampleTS1_Locus_27877_Transcript_2/3_Confidence_0.333_Length_987, Sample3_Locus_9475_Transcript_2/2_Confidence_0.000_Length_1080, Sample3_Locus_982_Transcript_1/1_Confidence_0.000_Length_2070, SampleTS3_Locus_26045_Transcript_2/2_Confidence_0.000_Length_444, Sample1_Locus_10139_Transcript_1/10_Confidence_0.538_Length_1144, SampleTS2_Locus_54_Transcript_4/10_Confidence_0.083_Length_1374, Sample6_Locus_15547_Transcript_5/8_Confidence_0.545_Length_1332, SampleTS3_Locus_8149_Transcript_4/10_Confidence_0.118_Length_927

**Methane metabolism**	map00680	13	ec:2.1.2.1 hydroxymethyltransferase, ec:4.1.2.13 aldolase, ec:4.1.1.31 carboxylase, ec:2.6.1.52 transaminase, ec:1.2.1.46 dehydrogenase, ec:2.6.1.45 transaminase, ec:6.2.1.1 ligase, ec:1.1.1.244 dehydrogenase, ec:3.1.3.11 hexose diphosphatase, ec:1.1.1.284 dehydrogenase, ec:3.1.3.3 phosphatase, ec:4.2.1.11 hydratase, ec:1.1.1.37 dehydrogenase	2, 2, 1, 1, 2, 1, 1, 1, 2, 2, 1, 1, 1	Sample1_Locus_3932_Transcript_7/11_Confidence_0.400_Length_1725, SampleTS1_Locus_10618_Transcript_3/4_Confidence_0.400_Length_1517, Sample3_Locus_324_Transcript_3/17_Confidence_0.222_Length_1711, Sample6_Locus_28252_Transcript_2/3_Confidence_0.500_Length_808, Sample3_Locus_7378_Transcript_3/6_Confidence_0.417_Length_3416, Sample1_Locus_9264_Transcript_1/1_Confidence_0.000_Length_1678, SampleTS2_Locus_4874_Transcript_6/12_Confidence_0.158_Length_1042, SampleTS3_Locus_23264_Transcript_1/3_Confidence_0.500_Length_845, Sample6_Locus_15604_Transcript_2/4_Confidence_0.400_Length_1416, SampleTS3_Locus_456_Transcript_2/6_Confidence_0.200_Length_2075, SampleTS2_Locus_263_Transcript_1/3_Confidence_0.250_Length_2184, Sample3_Locus_10262_Transcript_4/5_Confidence_0.062_Length_1337, Sample6_Locus_2283_Transcript_7/17_Confidence_0.225_Length_1818, SampleTS2_Locus_4874_Transcript_6/12_Confidence_0.158_Length_1042, SampleTS3_Locus_23264_Transcript_1/3_Confidence_0.500_Length_845, Sample1_Locus_18291_Transcript_1/1_Confidence_0.000_Length_647, SampleTS1_Locus_3608_Transcript_5/7_Confidence_0.222_Length_1514, Sample6_Locus_293_Transcript_1/2_Confidence_0.333_Length_875

**Glutathione metabolism**	map00480	13	ec:3.5.2.9 (ATPhydrolysing), ec:1.11.1.9 peroxidase, ec:1.17.4.1 reductase, ec:2.5.1.18 transferase, ec:4.1.1.17 decarboxylase, ec:1.8.1.7 reductase, ec:1.1.1.44 dehydrogenase (NADP+dependent, decarboxylating), ec:1.1.1.43 2dehydrogenase, ec:2.3.2.2 glutamyl transpeptidase, ec:1.1.1.49 dehydrogenase (NADP+), ec:1.11.1.11 peroxidase, ec:1.11.1.12 glutathione peroxidase, ec:1.11.1.15 thioredoxin peroxidase	1, 6, 1, 6, 2, 3, 2, 1, 1, 2, 2, 1, 4	Sample3_Locus_15598_Transcript_4/6_Confidence_0.286_Length_1365, SampleTS1_Locus_6640_Transcript_2/7_Confidence_0.357_Length_1361, SampleTS3_Locus_2808_Transcript_2/4_Confidence_0.333_Length_770, Sample6_Locus_795_Transcript_19/29_Confidence_0.175_Length_1113, Sample3_Locus_2715_Transcript_4/6_Confidence_0.286_Length_835, Sample1_Locus_18818_Transcript_1/1_Confidence_0.000_Length_983, Sample6_Locus_1113_Transcript_3/14_Confidence_0.211_Length_520, SampleTS3_Locus_11402_Transcript_2/3_Confidence_0.333_Length_597, SampleTS1_Locus_6640_Transcript_2/7_Confidence_0.357_Length_1361, Sample6_Locus_795_Transcript_19/29_Confidence_0.175_Length_1113, Unigene1242_Br_gene19, SampleTS2_Locus_4937_Transcript_4/7_Confidence_0.444_Length_827, Sample3_Locus_2715_Transcript_4/6_Confidence_0.286_Length_835, SampleTS1_Locus_23227_Transcript_3/3_Confidence_0.000_Length_1280, Sample6_Locus_7028_Transcript_1/3_Confidence_0.750_Length_1269, SampleTS1_Locus_23304_Transcript_2/3_Confidence_0.333_Length_2324, SampleTS2_Locus_3616_Transcript_1/2_Confidence_0.333_Length_1862, Sample6_Locus_711_Transcript_5/8_Confidence_0.500_Length_1354, SampleTS1_Locus_5852_Transcript_11/12_Confidence_0.615_Length_1704, Sample6_Locus_10552_Transcript_5/5_Confidence_0.000_Length_1013, SampleTS1_Locus_3938_Transcript_9/14_Confidence_0.467_Length_2040, SampleTS1_Locus_3938_Transcript_9/14_Confidence_0.467_Length_2040, Sample1_Locus_19449_Transcript_1/1_Confidence_0.000_Length_1704, SampleTS1_Locus_4380_Transcript_8/12_Confidence_0.400_Length_2044, SampleTS3_Locus_2991_Transcript_5/8_Confidence_0.400_Length_1799, SampleTS1_Locus_6669_Transcript_3/3_Confidence_0.200_Length_979, SampleTS1_Locus_1282_Transcript_9/25_Confidence_0.318_Length_1204, Sample1_Locus_18818_Transcript_1/1_Confidence_0.000_Length_983, SampleTS2_Locus_21396_Transcript_1/2_Confidence_0.667_Length_780, SampleTS3_Locus_2808_Transcript_2/4_Confidence_0.333_Length_770, Sample3_Locus_11502_Transcript_4/7_Confidence_0.636_Length_850, Sample3_Locus_13267_Transcript_2/3_Confidence_0.667_Length_841

**Galactose metabolism**	map00052	13	ec:2.7.1.2 glucokinase (phosphorylating), ec:2.7.1.1 hexokinase type IV glucokinase, ec:2.7.1.6 galactokinase (phosphorylating), ec:2.4.1.123 3alphagalactosyltransferase, ec:5.1.3.3 1epimerase, ec:5.1.3.2 4epimerase, ec:2.7.7.9 uridylyltransferase, ec:2.7.7.64 uridylyltransferase, ec:2.7.7.10 uridylyltransferase, ec:2.4.1.82 galactosyltransferase, ec:3.2.1.22 melibiase, ec:3.2.1.23 lactase (ambiguous), ec:1.1.1.21 reductase	1, 1, 2, 1, 1, 1, 5, 5, 1, 1, 1, 1, 2	Sample6_Locus_5095_Transcript_5/8_Confidence_0.300_Length_1241, Sample6_Locus_5095_Transcript_5/8_Confidence_0.300_Length_1241, Sample3_Locus_7188_Transcript_3/12_Confidence_0.615_Length_971, Sample3_Locus_7508_Transcript_1/2_Confidence_0.333_Length_994, SampleTS3_Locus_5338_Transcript_2/2_Confidence_0.000_Length_543, Sample3_Locus_10443_Transcript_10/11_Confidence_0.700_Length_2690, SampleTS2_Locus_4938_Transcript_4/9_Confidence_0.273_Length_771, SampleTS3_Locus_2330_Transcript_4/4_Confidence_0.000_Length_1686, SampleTS1_Locus_16292_Transcript_4/5_Confidence_0.600_Length_1341, Sample3_Locus_4208_Transcript_8/15_Confidence_0.091_Length_2117, Sample3_Locus_40_Transcript_1/1_Confidence_0.000_Length_1702, Sample3_Locus_14770_Transcript_1/1_Confidence_0.000_Length_1819, SampleTS3_Locus_2330_Transcript_4/4_Confidence_0.000_Length_1686, SampleTS1_Locus_16292_Transcript_4/5_Confidence_0.600_Length_1341, Sample3_Locus_4208_Transcript_8/15_Confidence_0.091_Length_2117, Sample3_Locus_40_Transcript_1/1_Confidence_0.000_Length_1702, Sample3_Locus_14770_Transcript_1/1_Confidence_0.000_Length_1819, SampleTS3_Locus_2330_Transcript_4/4_Confidence_0.000_Length_1686, SampleTS2_Locus_3316_Transcript_5/7_Confidence_0.211_Length_3110, SampleTS2_Locus_3316_Transcript_5/7_Confidence_0.211_Length_3110, SampleTS2_Locus_9286_Transcript_1/1_Confidence_0.000_Length_2119, Sample6_Locus_15547_Transcript_5/8_Confidence_0.545_Length_1332, SampleTS3_Locus_8149_Transcript_4/10_Confidence_0.118_Length_927

**Fatty acid degradation**	map00071	12	ec:5.1.2.3 epimerase, ec:6.2.1.3 ligase, ec:2.3.1.16 Cacyltransferase, ec:4.2.1.74 hydratase, ec:1.1.1.1 dehydrogenase, ec:5.3.3.8 isomerase, ec:4.2.1.17 hydratase, ec:1.1.1.211 dehydrogenase, ec:1.2.1.3 dehydrogenase (NAD+), ec:2.3.1.9 Cacetyltransferase, ec:1.1.1.35 dehydrogenase, ec:1.3.3.6 oxidase	1, 4, 1, 1, 5, 1, 2, 1, 6, 1, 3, 2	SampleTS1_Locus_789_Transcript_9/11_Confidence_0.238_Length_2300, SampleTS1_Locus_369_Transcript_24/52_Confidence_0.312_Length_2199, SampleTS1_Locus_24343_Transcript_2/3_Confidence_0.333_Length_1375, Sample1_Locus_5442_Transcript_10/12_Confidence_0.091_Length_2073, Sample3_Locus_31478_Transcript_1/1_Confidence_0.333_Length_620, SampleTS2_Locus_1750_Transcript_17/20_Confidence_0.182_Length_1568, SampleTS1_Locus_789_Transcript_9/11_Confidence_0.238_Length_2300, SampleTS2_Locus_4874_Transcript_6/12_Confidence_0.158_Length_1042, Sample6_Locus_1524_Transcript_26/30_Confidence_0.185_Length_3215, SampleTS2_Locus_263_Transcript_1/3_Confidence_0.250_Length_2184, SampleTS3_Locus_400_Transcript_6/29_Confidence_0.422_Length_2479, SampleTS3_Locus_23264_Transcript_1/3_Confidence_0.500_Length_845, SampleTS1_Locus_789_Transcript_9/11_Confidence_0.238_Length_2300, SampleTS1_Locus_789_Transcript_9/11_Confidence_0.238_Length_2300, Sample3_Locus_5282_Transcript_2/7_Confidence_0.700_Length_1266, SampleTS3_Locus_11808_Transcript_9/11_Confidence_0.565_Length_1006, SampleTS3_Locus_23038_Transcript_2/3_Confidence_0.333_Length_1864, SampleTS2_Locus_4874_Transcript_6/12_Confidence_0.158_Length_1042, Sample3_Locus_23176_Transcript_2/3_Confidence_0.333_Length_1282, SampleTS1_Locus_5373_Transcript_26/33_Confidence_0.065_Length_1620, Sample6_Locus_12695_Transcript_1/2_Confidence_0.333_Length_2218, SampleTS3_Locus_23264_Transcript_1/3_Confidence_0.500_Length_845, SampleTS2_Locus_1750_Transcript_17/20_Confidence_0.182_Length_1568, SampleTS1_Locus_789_Transcript_9/11_Confidence_0.238_Length_2300, SampleTS3_Locus_1827_Transcript_3/3_Confidence_0.000_Length_1033, SampleTS3_Locus_11808_Transcript_9/11_Confidence_0.565_Length_1006, SampleTS2_Locus_1750_Transcript_17/20_Confidence_0.182_Length_1568, Sample3_Locus_6833_Transcript_1/1_Confidence_0.000_Length_1688

**Arginine and proline metabolism**	map00330	12	ec:2.6.1.13 aminotransferase, ec:4.1.1.50 decarboxylase, ec:1.14.13.39 synthase (NADPH), ec:2.7.3.2 kinase, ec:2.7.2.11 5kinase, ec:1.2.1.41 dehydrogenase, ec:1.14.11.2 4dioxygenase, ec:4.1.1.17 decarboxylase, ec:2.6.1.1 transaminase, ec:1.2.1.3 dehydrogenase (NAD+), ec:1.5.1.2 reductase, ec:3.5.1.4 acylamidase	1, 1, 1, 1, 2, 3, 6, 2, 3, 6, 1, 2	SampleTS3_Locus_10707_Transcript_4/7_Confidence_0.500_Length_1675, SampleTS1_Locus_23304_Transcript_2/3_Confidence_0.333_Length_2324, Sample1_Locus_25642_Transcript_6/7_Confidence_0.500_Length_1752, SampleTS2_Locus_24331_Transcript_1/2_Confidence_0.500_Length_2444, Sample3_Locus_23456_Transcript_1/1_Confidence_0.000_Length_1406, SampleTS2_Locus_20780_Transcript_2/3_Confidence_0.600_Length_1460, Sample3_Locus_23456_Transcript_1/1_Confidence_0.000_Length_1406, Sample1_Locus_3087_Transcript_2/3_Confidence_0.333_Length_2354, SampleTS2_Locus_20780_Transcript_2/3_Confidence_0.600_Length_1460, SampleTS3_Locus_1508_Transcript_1/3_Confidence_0.333_Length_933, SampleTS3_Locus_1313_Transcript_8/10_Confidence_0.615_Length_1118, SampleTS1_Locus_10911_Transcript_1/1_Confidence_0.000_Length_1788, SampleTS2_Locus_3141_Transcript_2/5_Confidence_0.500_Length_1713, Sample1_Locus_19782_Transcript_4/6_Confidence_0.200_Length_831, Sample3_Locus_2709_Transcript_17/22_Confidence_0.089_Length_783, Sample6_Locus_7028_Transcript_1/3_Confidence_0.750_Length_1269, SampleTS1_Locus_23304_Transcript_2/3_Confidence_0.333_Length_2324, Sample1_Locus_26802_Transcript_1/2_Confidence_0.667_Length_439, Sample6_Locus_13664_Transcript_1/2_Confidence_0.333_Length_862, SampleTS2_Locus_6646_Transcript_2/2_Confidence_0.333_Length_1520, SampleTS3_Locus_23038_Transcript_2/3_Confidence_0.333_Length_1864, SampleTS2_Locus_4874_Transcript_6/12_Confidence_0.158_Length_1042, Sample3_Locus_23176_Transcript_2/3_Confidence_0.333_Length_1282, SampleTS1_Locus_5373_Transcript_26/33_Confidence_0.065_Length_1620, Sample6_Locus_12695_Transcript_1/2_Confidence_0.333_Length_2218, SampleTS3_Locus_23264_Transcript_1/3_Confidence_0.500_Length_845, SampleTS1_Locus_743_Transcript_11/14_Confidence_0.154_Length_1525, Sample6_Locus_14763_Transcript_11/12_Confidence_0.182_Length_1117, Sample6_Locus_19655_Transcript_1/3_Confidence_0.667_Length_978

**Tyrosine metabolism**	map00350	12	ec:1.4.3.2 oxidase, ec:2.6.1.57 transaminase, ec:2.6.1.58 transaminase, ec:3.7.1.2 betadiketonase, ec:1.13.11.27 dioxygenase, ec:1.1.1.1 dehydrogenase, ec:2.6.1.5 transaminase, ec:2.6.1.9 transaminase, ec:2.6.1.1 transaminase, ec:1.2.1.5 dehydrogenase [NAD(P)+], ec:1.14.16.2 3monooxygenase, ec:1.4.3.21 oxidase	1, 2, 1, 1, 3, 5, 2, 2, 3, 1, 1, 1	SampleTS1_Locus_1041_Transcript_1/5_Confidence_0.400_Length_2190, Sample1_Locus_26802_Transcript_1/2_Confidence_0.667_Length_439, Sample6_Locus_13664_Transcript_1/2_Confidence_0.333_Length_862, Sample6_Locus_22798_Transcript_3/3_Confidence_0.250_Length_666, SampleTS1_Locus_6929_Transcript_8/20_Confidence_0.293_Length_1407 , SampleTS1_Locus_605_Transcript_11/25_Confidence_0.258_Length_787, SampleTS2_Locus_361_Transcript_3/4_Confidence_0.750_Length_304, SampleTS1_Locus_605_Transcript_3/25_Confidence_0.161_Length_805, SampleTS2_Locus_4874_Transcript_6/12_Confidence_0.158_Length_1042 , Sample6_Locus_1524_Transcript_26/30_Confidence_0.185_Length_3215, SampleTS2_Locus_263_Transcript_1/3_Confidence_0.250_Length_2184, SampleTS3_Locus_400_Transcript_6/29_Confidence_0.422_Length_2479, SampleTS3_Locus_23264_Transcript_1/3_Confidence_0.500_Length_845, Sample1_Locus_26802_Transcript_1/2_Confidence_0.667_Length_439, Sample6_Locus_13664_Transcript_1/2_Confidence_0.333_Length_862, Sample1_Locus_26802_Transcript_1/2_Confidence_0.667_Length_439, Sample6_Locus_13664_Transcript_1/2_Confidence_0.333_Length_862, Sample1_Locus_26802_Transcript_1/2_Confidence_0.667_Length_439, Sample6_Locus_13664_Transcript_1/2_Confidence_0.333_Length_862, SampleTS2_Locus_6646_Transcript_2/2_Confidence_0.333_Length_1520, SampleTS1_Locus_5373_Transcript_26/33_Confidence_0.065_Length_1620, Sample3_Locus_2321_Transcript_7/9_Confidence_0.414_Length_1007, Sample1_Locus_4359_Transcript_11/14_Confidence_0.314_Length_2540

**Folate biosynthesis**	map00790	11	ec:1.1.1.184 reductase (NADPH), ec:4.1.99.22 3',8cyclase, ec:6.3.2.12 synthase, ec:2.6.1.85 synthase, ec:4.6.1.17 pyranopterin monophosphate synthase, ec:2.7.7.75 adenylyltransferase, ec:2.10.1.1 molybdotransferase, ec:1.1.1.21 reductase, ec:1.14.16.4 5monooxygenase, ec:1.14.16.1 4monooxygenase, ec:1.14.16.2 3monooxygenase	1, 2, 1, 1, 2, 2, 1, 2, 1, 1, 1	Sample6_Locus_15547_Transcript_5/8_Confidence_0.545_Length_1332, Sample1_Locus_18859_Transcript_2/4_Confidence_0.600_Length_1538, SampleTS1_Locus_832_Transcript_11/13_Confidence_0.615_Length_1939, Sample6_Locus_15388_Transcript_2/3_Confidence_0.333_Length_1168, SampleTS3_Locus_14492_Transcript_3/7_Confidence_0.111_Length_1768, Sample1_Locus_18859_Transcript_2/4_Confidence_0.600_Length_1538, SampleTS1_Locus_832_Transcript_11/13_Confidence_0.615_Length_1939, SampleTS3_Locus_4837_Transcript_2/4_Confidence_0.750_Length_1516, Sample3_Locus_26982_Transcript_2/2_Confidence_0.000_Length_647, SampleTS3_Locus_4837_Transcript_2/4_Confidence_0.750_Length_1516, Sample6_Locus_15547_Transcript_5/8_Confidence_0.545_Length_1332, SampleTS3_Locus_8149_Transcript_4/10_Confidence_0.118_Length_927, Sample3_Locus_2321_Transcript_7/9_Confidence_0.414_Length_1007, Sample3_Locus_2321_Transcript_7/9_Confidence_0.414_Length_1007, Sample3_Locus_2321_Transcript_7/9_Confidence_0.414_Length_1007

**Glycerolipid metabolism**	map00561	11	ec:2.3.1.15 1Oacyltransferase, ec:2.3.1.20 Oacyltransferase, ec:3.1.1.3 lipase, ec:2.3.1.51 Oacyltransferase, ec:3.1.3.4 phosphatase, ec:1.1.1.2 dehydrogenase (NADP+), ec:1.1.1.202 dehydrogenase, ec:1.2.1.3 dehydrogenase (NAD+), ec:3.2.1.22 melibiase, ec:1.1.1.21 reductase, ec:2.4.1.46 synthase	4, 2, 1, 1, 2, 10, 1, 6, 1, 2, 2	SampleTS1_Locus_7733_Transcript_3/6_Confidence_0.615_Length_1523, Sample6_Locus_335_Transcript_1/5_Confidence_0.214_Length_2401, Sample1_Locus_24278_Transcript_1/2_Confidence_0.667_Length_689, SampleTS2_Locus_5399_Transcript_4/5_Confidence_0.034_Length_1249, Sample1_Locus_18933_Transcript_2/3_Confidence_0.714_Length_599, Sample3_Locus_18000_Transcript_1/1_Confidence_0.000_Length_845, Sample6_Locus_7047_Transcript_8/10_Confidence_0.182_Length_2472, SampleTS1_Locus_7733_Transcript_3/6_Confidence_0.615_Length_1523, Sample1_Locus_2193_Transcript_40/42_Confidence_0.262_Length_1445, SampleTS2_Locus_23786_Transcript_1/1_Confidence_0.000_Length_866, Sample6_Locus_15547_Transcript_5/8_Confidence_0.545_Length_1332, Sample6_Locus_7369_Transcript_3/6_Confidence_0.200_Length_1828, SampleTS1_Locus_2734_Transcript_1/3_Confidence_0.333_Length_2491, SampleTS2_Locus_15764_Transcript_1/1_Confidence_0.000_Length_1068, Sample3_Locus_27117_Transcript_1/3_Confidence_0.250_Length_685, Sample6_Locus_6954_Transcript_4/5_Confidence_0.500_Length_1201, Sample6_Locus_4129_Transcript_8/27_Confidence_0.107_Length_571, SampleTS3_Locus_8149_Transcript_4/10_Confidence_0.118_Length_927, SampleTS1_Locus_6731_Transcript_14/15_Confidence_0.246_Length_2303, SampleTS1_Locus_10087_Transcript_6/7_Confidence_0.167_Length_948, SampleTS2_Locus_263_Transcript_1/3_Confidence_0.250_Length_2184, SampleTS3_Locus_23038_Transcript_2/3_Confidence_0.333_Length_1864, SampleTS2_Locus_4874_Transcript_6/12_Confidence_0.158_Length_1042, Sample3_Locus_23176_Transcript_2/3_Confidence_0.333_Length_1282, SampleTS1_Locus_5373_Transcript_26/33_Confidence_0.065_Length_1620, Sample6_Locus_12695_Transcript_1/2_Confidence_0.333_Length_2218, SampleTS3_Locus_23264_Transcript_1/3_Confidence_0.500_Length_845, SampleTS2_Locus_3316_Transcript_5/7_Confidence_0.211_Length_3110, Sample6_Locus_15547_Transcript_5/8_Confidence_0.545_Length_1332, SampleTS3_Locus_8149_Transcript_4/10_Confidence_0.118_Length_927, Sample3_Locus_4295_Transcript_2/4_Confidence_0.750_Length_1683, Sample3_Locus_16636_Transcript_4/5_Confidence_0.364_Length_994

**Pentose and glucuronate interconversions**	map00040	11	ec:5.1.3.1 3epimerase, ec:2.7.7.9 uridylyltransferase, ec:2.7.7.64 uridylyltransferase, ec:1.1.1.2 dehydrogenase (NADP+), ec:2.7.7.44 uridylyltransferase, ec:1.1.1.57 reductase, ec:1.1.1.14 2dehydrogenase, ec:1.1.1.19 reductase, ec:1.1.1.22 6dehydrogenase, ec:1.1.1.21 reductase, ec:2.4.1.17 1naphthol glucuronyltransferase	1, 5, 5, 10, 1, 1, 1, 1, 2, 2, 3	Sample3_Locus_3334_Transcript_1/5_Confidence_0.400_Length_1030, SampleTS3_Locus_2330_Transcript_4/4_Confidence_0.000_Length_1686, SampleTS1_Locus_16292_Transcript_4/5_Confidence_0.600_Length_1341, Sample3_Locus_4208_Transcript_8/15_Confidence_0.091_Length_2117, Sample3_Locus_40_Transcript_1/1_Confidence_0.000_Length_1702, Sample3_Locus_14770_Transcript_1/1_Confidence_0.000_Length_1819, SampleTS3_Locus_2330_Transcript_4/4_Confidence_0.000_Length_1686, SampleTS1_Locus_16292_Transcript_4/5_Confidence_0.600_Length_1341, Sample3_Locus_4208_Transcript_8/15_Confidence_0.091_Length_2117, Sample3_Locus_40_Transcript_1/1_Confidence_0.000_Length_1702, Sample3_Locus_14770_Transcript_1/1_Confidence_0.000_Length_1819, Sample6_Locus_15547_Transcript_5/8_Confidence_0.545_Length_1332, Sample6_Locus_7369_Transcript_3/6_Confidence_0.200_Length_1828, SampleTS1_Locus_2734_Transcript_1/3_Confidence_0.333_Length_2491, SampleTS2_Locus_15764_Transcript_1/1_Confidence_0.000_Length_1068, Sample3_Locus_27117_Transcript_1/3_Confidence_0.250_Length_685, Sample6_Locus_6954_Transcript_4/5_Confidence_0.500_Length_1201, Sample6_Locus_4129_Transcript_8/27_Confidence_0.107_Length_571, SampleTS3_Locus_8149_Transcript_4/10_Confidence_0.118_Length_927, SampleTS1_Locus_6731_Transcript_14/15_Confidence_0.246_Length_2303, SampleTS1_Locus_10087_Transcript_6/7_Confidence_0.167_Length_948, SampleTS3_Locus_2330_Transcript_4/4_Confidence_0.000_Length_1686, SampleTS3_Locus_26045_Transcript_2/2_Confidence_0.000_Length_444, SampleTS2_Locus_54_Transcript_4/10_Confidence_0.083_Length_1374, SampleTS3_Locus_8149_Transcript_4/10_Confidence_0.118_Length_927, SampleTS3_Locus_1975_Transcript_19/64_Confidence_0.064_Length_546, SampleTS2_Locus_4_Transcript_35/36_Confidence_0.021_Length_1580, Sample6_Locus_15547_Transcript_5/8_Confidence_0.545_Length_1332, SampleTS3_Locus_8149_Transcript_4/10_Confidence_0.118_Length_927, Sample6_Locus_9455_Transcript_3/4_Confidence_0.200_Length_1370, SampleTS1_Locus_4303_Transcript_9/11_Confidence_0.167_Length_1464, Sample6_Locus_8395_Transcript_14/17_Confidence_0.508_Length_1568

**betaAlanine metabolism**	map00410	10	ec:2.6.1.18 transaminase, ec:2.6.1.19 transaminase, ec:3.5.2.2 hydantoinase, ec:1.2.1.18 dehydrogenase (acetylating), ec:4.1.1.15 decarboxylase, ec:4.2.1.17 hydratase, ec:1.2.1.5 dehydrogenase [NAD(P)+], ec:1.2.1.3 dehydrogenase (NAD+), ec:1.3.1.2 dehydrogenase (NADP+), ec:1.4.3.21 oxidase	1, 1, 2, 1, 1, 2, 1, 6, 1, 1	Sample3_Locus_1833_Transcript_4/6_Confidence_0.500_Length_1265, SampleTS1_Locus_13173_Transcript_7/8_Confidence_0.190_Length_747, SampleTS1_Locus_275_Transcript_59/85_Confidence_0.013_Length_590, Sample6_Locus_10051_Transcript_2/3_Confidence_0.333_Length_1368, SampleTS3_Locus_23038_Transcript_2/3_Confidence_0.333_Length_1864, SampleTS3_Locus_5084_Transcript_15/17_Confidence_0.206_Length_1751, SampleTS1_Locus_789_Transcript_9/11_Confidence_0.238_Length_2300, Sample3_Locus_5282_Transcript_2/7_Confidence_0.700_Length_1266, SampleTS1_Locus_5373_Transcript_26/33_Confidence_0.065_Length_1620, SampleTS3_Locus_23038_Transcript_2/3_Confidence_0.333_Length_1864, SampleTS2_Locus_4874_Transcript_6/12_Confidence_0.158_Length_1042, Sample3_Locus_23176_Transcript_2/3_Confidence_0.333_Length_1282, SampleTS1_Locus_5373_Transcript_26/33_Confidence_0.065_Length_1620, Sample6_Locus_12695_Transcript_1/2_Confidence_0.333_Length_2218, SampleTS3_Locus_23264_Transcript_1/3_Confidence_0.500_Length_845, SampleTS2_Locus_1483_Transcript_3/13_Confidence_0.364_Length_1620, Sample1_Locus_4359_Transcript_11/14_Confidence_0.314_Length_2540

**Pantothenate and CoA biosynthesis**	map00770	10	ec:3.5.2.2 hydantoinase, ec:3.6.1.9 diphosphatase, ec:4.2.1.9 dehydratase, ec:2.2.1.6 synthase, ec:6.3.2.5 ligase (CTP), ec:2.7.1.33 kinase, ec:2.6.1.42 transaminase, ec:2.7.7.3 adenylyltransferase, ec:1.1.1.86 reductoisomerase (NADP+), ec:1.3.1.2 dehydrogenase (NADP+)	2, 5, 1, 1, 1, 1, 2, 1, 1, 1	SampleTS1_Locus_275_Transcript_59/85_Confidence_0.013_Length_590, Sample6_Locus_10051_Transcript_2/3_Confidence_0.333_Length_1368, Sample6_Locus_1849_Transcript_4/6_Confidence_0.429_Length_866, Sample3_Locus_345_Transcript_6/8_Confidence_0.455_Length_1801, SampleTS3_Locus_5573_Transcript_4/5_Confidence_0.333_Length_940, Sample3_Locus_10699_Transcript_3/4_Confidence_0.400_Length_957, Sample3_Locus_27182_Transcript_2/3_Confidence_0.250_Length_1010, Sample1_Locus_2272_Transcript_43/67_Confidence_0.374_Length_2859, Sample3_Locus_4596_Transcript_3/6_Confidence_0.400_Length_1157, SampleTS2_Locus_6327_Transcript_8/25_Confidence_0.174_Length_1156, SampleTS1_Locus_877_Transcript_3/8_Confidence_0.375_Length_2543, Sample1_Locus_18777_Transcript_2/4_Confidence_0.923_Length_1416, Sample3_Locus_5952_Transcript_4/4_Confidence_0.000_Length_2586, SampleTS1_Locus_19274_Transcript_4/5_Confidence_0.038_Length_1457, Sample1_Locus_2001_Transcript_4/7_Confidence_0.600_Length_1686, SampleTS2_Locus_1483_Transcript_3/13_Confidence_0.364_Length_1620

**Arginine biosynthesis**	map00220	10	ec:2.6.1.11 transaminase, ec:1.14.13.39 synthase (NADPH), ec:6.3.1.2 synthetase, ec:2.3.1.35 Nacetyltransferase, ec:6.3.4.16 synthase (ammonia), ec:2.6.1.2 transaminase, ec:2.6.1.1 transaminase, ec:2.3.1.1 Nacetyltransferase, ec:3.5.1.2 glutaminase I, ec:3.5.1.5 ec:3.5.1.5 urease	1, 1, 2, 2, 1, 3, 3, 2, 2, 1	SampleTS1_Locus_13173_Transcript_7/8_Confidence_0.190_Length_747, Sample1_Locus_25642_Transcript_6/7_Confidence_0.500_Length_1752, Sample1_Locus_3256_Transcript_2/10_Confidence_0.182_Length_1415, SampleTS2_Locus_4400_Transcript_6/7_Confidence_0.333_Length_1376, Sample3_Locus_17004_Transcript_3/5_Confidence_0.286_Length_574, Sample3_Locus_3732_Transcript_3/7_Confidence_0.545_Length_970, SampleTS3_Locus_15776_Transcript_1/2_Confidence_0.333_Length_1102 , Sample6_Locus_2195_Transcript_14/16_Confidence_0.176_Length_1741, SampleTS3_Locus_714_Transcript_7/14_Confidence_0.087_Length_1194, Sample1_Locus_19449_Transcript_1/1_Confidence_0.000_Length_1704, Sample1_Locus_26802_Transcript_1/2_Confidence_0.667_Length_439, Sample6_Locus_13664_Transcript_1/2_Confidence_0.333_Length_862, SampleTS2_Locus_6646_Transcript_2/2_Confidence_0.333_Length_1520, Sample3_Locus_17004_Transcript_3/5_Confidence_0.286_Length_574, Sample3_Locus_3732_Transcript_3/7_Confidence_0.545_Length_970, Sample6_Locus_3307_Transcript_18/23_Confidence_0.163_Length_735, SampleTS1_Locus_5169_Transcript_10/19_Confidence_0.593_Length_3162, SampleTS3_Locus_10659_Transcript_7/8_Confidence_0.389_Length_1749

**Nitrogen metabolism**	map00910	10	ec:4.2.1.1 anhydrase, ec:6.3.1.2 synthetase, ec:3.5.5.1 acetonitrilase, ec:1.4.7.1 synthase (ferredoxin), ec:6.3.4.16 synthase (ammonia), ec:1.7.99.1 reductase, ec:1.7.1.1 reductase (NADH), ec:1.7.1.3 reductase (NADPH), ec:4.2.1.104 cyanate lyase, ec:1.4.1.14 synthase (NADH)	1, 2, 1, 1, 1, 2, 4, 1, 1, 2	Sample6_Locus_4308_Transcript_3/6_Confidence_0.429_Length_1128, Sample1_Locus_3256_Transcript_2/10_Confidence_0.182_Length_1415, SampleTS2_Locus_4400_Transcript_6/7_Confidence_0.333_Length_1376, Sample3_Locus_27182_Transcript_2/3_Confidence_0.250_Length_1010, SampleTS3_Locus_21707_Transcript_1/2_Confidence_0.333_Length_2460 , SampleTS3_Locus_15776_Transcript_1/2_Confidence_0.333_Length_1102 , SampleTS2_Locus_20766_Transcript_5/7_Confidence_0.250_Length_1030 , Sample3_Locus_2283_Transcript_4/7_Confidence_0.091_Length_449, SampleTS1_Locus_26901_Transcript_2/4_Confidence_0.095_Length_536, SampleTS1_Locus_16209_Transcript_2/6_Confidence_0.308_Length_976, SampleTS1_Locus_11635_Transcript_3/3_Confidence_0.000_Length_825, SampleTS3_Locus_14467_Transcript_1/1_Confidence_0.000_Length_1733 , SampleTS2_Locus_13872_Transcript_6/7_Confidence_0.500_Length_988, SampleTS2_Locus_4384_Transcript_2/13_Confidence_0.653_Length_812, SampleTS3_Locus_21707_Transcript_1/2_Confidence_0.333_Length_2460 , SampleTS1_Locus_1310_Transcript_14/17_Confidence_0.222_Length_1901

**Terpenoid backbone biosynthesis**	map00900	10	ec:1.17.7.1 synthase (ferredoxin), ec:1.1.1.216 dehydrogenase (NADP+), ec:2.5.1.29 diphosphate synthase, ec:2.7.1.148 5'diphospho)2C methylDerythritol kinase, ec:2.5.1.85 diphosphate synthase [geranylgeranyl diphosphate specific], ec:2.5.1.84 diphosphate synthase [geranyldiphosphate specific], ec:2.5.1.1 geranyldiphosphate synthase, ec:5.3.3.2 Deltaisomerase, ec:2.5.1.58 farnesyltransferase, ec:2.3.1.9 Cacetyltransferase	2, 1, 1, 1, 2, 2, 2, 1, 2, 1	Sample6_Locus_5736_Transcript_2/4_Confidence_0.400_Length_1660, SampleTS1_Locus_12312_Transcript_2/4_Confidence_0.333_Length_2330, SampleTS1_Locus_13725_Transcript_2/7_Confidence_0.333_Length_1080, Sample6_Locus_901_Transcript_6/8_Confidence_0.643_Length_1628, Sample6_Locus_16912_Transcript_4/6_Confidence_0.286_Length_1521, SampleTS2_Locus_13350_Transcript_1/3_Confidence_0.778_Length_1614, SampleTS3_Locus_7085_Transcript_3/3_Confidence_0.000_Length_1349, SampleTS2_Locus_13350_Transcript_1/3_Confidence_0.778_Length_1614, SampleTS3_Locus_7085_Transcript_3/3_Confidence_0.000_Length_1349, SampleTS2_Locus_13350_Transcript_1/3_Confidence_0.778_Length_1614, SampleTS3_Locus_7085_Transcript_3/3_Confidence_0.000_Length_1349, Sample6_Locus_5354_Transcript_1/7_Confidence_0.333_Length_1260, SampleTS1_Locus_610_Transcript_52/54_Confidence_0.210_Length_1630, SampleTS2_Locus_2631_Transcript_10/37_Confidence_0.203_Length_869, SampleTS2_Locus_1750_Transcript_17/20_Confidence_0.182_Length_1568
**Pentose phosphate pathway**	map00030	10	ec:4.1.2.13 aldolase, ec:2.2.1.1 glycolaldehydetransferase, ec:5.1.3.1 3epimerase, ec:3.1.3.11 hexose diphosphatase, ec:2.7.6.1 diphosphokinase, ec:5.3.1.6 isomerase, ec:5.3.1.9 isomerase, ec:1.1.1.44 dehydrogenase (NADP+dependent, decarboxylating), ec:1.1.1.43 2dehydrogenase, ec:1.1.1.49 dehydrogenase (NADP+)	2, 1, 1, 2, 1, 2, 1, 2, 1, 2	Sample3_Locus_324_Transcript_3/17_Confidence_0.222_Length_1711, Sample6_Locus_28252_Transcript_2/3_Confidence_0.500_Length_808, SampleTS1_Locus_16129_Transcript_1/1_Confidence_0.000_Length_1960, Sample3_Locus_3334_Transcript_1/5_Confidence_0.400_Length_1030, Sample3_Locus_10262_Transcript_4/5_Confidence_0.062_Length_1337, Sample6_Locus_2283_Transcript_7/17_Confidence_0.225_Length_1818, Sample6_Locus_81_Transcript_6/10_Confidence_0.421_Length_1354, SampleTS1_Locus_27877_Transcript_2/3_Confidence_0.333_Length_987, Sample3_Locus_9475_Transcript_2/2_Confidence_0.000_Length_1080, SampleTS1_Locus_12696_Transcript_3/3_Confidence_0.000_Length_1743, Sample6_Locus_10552_Transcript_5/5_Confidence_0.000_Length_1013, SampleTS1_Locus_3938_Transcript_9/14_Confidence_0.467_Length_2040, SampleTS1_Locus_3938_Transcript_9/14_Confidence_0.467_Length_2040, SampleTS1_Locus_4380_Transcript_8/12_Confidence_0.400_Length_2044, SampleTS3_Locus_2991_Transcript_5/8_Confidence_0.400_Length_1799

**Sulfur metabolism**	map00920	10	ec:1.8.7.1 sulfite reductase (ferredoxin), ec:3.6.2.1 adenosine 5phosphosulfate sulfohydrolase, ec:2.7.1.25 kinase, ec:2.7.7.4 adenylyltransferase, ec:2.7.7.5 adenylyltransferase (ADP), ec:2.3.1.30 Oacetyltransferase, ec:1.13.11.18 dioxygenase, ec:1.8.1.2 sulfite reductase (NADPH), ec:2.5.1.47 synthase, ec:1.8.99.2 reductase	2, 1, 2, 3, 1, 1, 1, 1, 3, 1	Sample3_Locus_6284_Transcript_3/6_Confidence_0.300_Length_2211, Sample6_Locus_4302_Transcript_2/16_Confidence_0.036_Length_613, SampleTS2_Locus_12297_Transcript_1/1_Confidence_0.000_Length_1037, Sample3_Locus_6616_Transcript_4/6_Confidence_0.286_Length_793, Sample1_Locus_521_Transcript_109/121_Confidence_0.005_Length_964, SampleTS3_Locus_4546_Transcript_3/8_Confidence_0.429_Length_1895, SampleTS3_Locus_3403_Transcript_3/4_Confidence_0.333_Length_1292, SampleTS1_Locus_25259_Transcript_1/2_Confidence_0.333_Length_1099, SampleTS2_Locus_12297_Transcript_1/1_Confidence_0.000_Length_1037, Sample3_Locus_6377_Transcript_4/4_Confidence_0.125_Length_1830, Sample1_Locus_24616_Transcript_1/1_Confidence_0.000_Length_806, Sample6_Locus_4302_Transcript_2/16_Confidence_0.036_Length_613, Sample1_Locus_15681_Transcript_1/1_Confidence_0.000_Length_1030, Sample6_Locus_5355_Transcript_5/8_Confidence_0.577_Length_1830, SampleTS2_Locus_2923_Transcript_3/4_Confidence_0.333_Length_1277, Sample1_Locus_3645_Transcript_14/15_Confidence_0.067_Length_1362

**Drug metabolism other enzymes**	map00983	9	ec:3.5.2.2 hydantoinase, ec:2.7.1.48 kinase, ec:2.7.1.21 kinase, ec:3.1.1.1 aliesterase, ec:1.1.1.205 dehydrogenase, ec:1.3.1.2 dehydrogenase (NADP+), ec:2.4.2.10 phosphoribosyltransferase, ec:2.4.1.17 1naphthol glucuronyltransferase, ec:2.4.2.8 phosphoribosyltransferase	2, 3, 1, 22, 1, 1, 1, 3, 1	SampleTS1_Locus_275_Transcript_59/85_Confidence_0.013_Length_590, Sample6_Locus_10051_Transcript_2/3_Confidence_0.333_Length_1368, Sample1_Locus_7813_Transcript_4/8_Confidence_0.643_Length_1238, SampleTS2_Locus_12437_Transcript_2/3_Confidence_0.667_Length_1244, SampleTS1_Locus_1762_Transcript_13/36_Confidence_0.058_Length_819, SampleTS3_Locus_6992_Transcript_3/6_Confidence_0.500_Length_986, SampleTS3_Locus_4982_Transcript_5/18_Confidence_0.148_Length_763, SampleTS2_Locus_16974_Transcript_1/1_Confidence_0.000_Length_814, Sample6_Locus_7047_Transcript_8/10_Confidence_0.182_Length_2472, Sample3_Locus_4061_Transcript_6/10_Confidence_0.500_Length_1879, Sample1_Locus_25249_Transcript_3/5_Confidence_0.400_Length_1933, SampleTS1_Locus_587_Transcript_4/24_Confidence_0.067_Length_950, Sample6_Locus_21506_Transcript_3/6_Confidence_0.286_Length_1572, Sample6_Locus_6377_Transcript_17/31_Confidence_0.105_Length_889, SampleTS2_Locus_23527_Transcript_1/1_Confidence_0.000_Length_1031, SampleTS2_Locus_1401_Transcript_25/29_Confidence_0.228_Length_1385, SampleTS2_Locus_6522_Transcript_9/11_Confidence_0.500_Length_1378, SampleTS2_Locus_5980_Transcript_2/3_Confidence_0.333_Length_1195, SampleTS3_Locus_8220_Transcript_1/4_Confidence_0.667_Length_1081, SampleTS1_Locus_5610_Transcript_2/3_Confidence_0.333_Length_1211, Sample6_Locus_26172_Transcript_1/1_Confidence_0.000_Length_814, Sample3_Locus_18908_Transcript_2/3_Confidence_0.333_Length_666, SampleTS1_Locus_11100_Transcript_2/5_Confidence_0.286_Length_1260, Sample6_Locus_7693_Transcript_9/11_Confidence_0.000_Length_836, SampleTS1_Locus_11345_Transcript_4/5_Confidence_0.692_Length_960, Sample6_Locus_1702_Transcript_13/18_Confidence_0.348_Length_1386, SampleTS2_Locus_2263_Transcript_6/8_Confidence_0.429_Length_1743, SampleTS1_Locus_12215_Transcript_1/1_Confidence_0.000_Length_1974, Sample3_Locus_18801_Transcript_1/3_Confidence_0.600_Length_1111, SampleTS2_Locus_1483_Transcript_3/13_Confidence_0.364_Length_1620, SampleTS1_Locus_6359_Transcript_7/7_Confidence_0.294_Length_1274, Sample6_Locus_9455_Transcript_3/4_Confidence_0.200_Length_1370, SampleTS1_Locus_4303_Transcript_9/11_Confidence_0.167_Length_1464, Sample6_Locus_8395_Transcript_14/17_Confidence_0.508_Length_1568, Sample3_Locus_3267_Transcript_2/5_Confidence_0.400_Length_1108

**Fatty acid biosynthesis**	map00061	9	ec:6.4.1.2 carboxylase, ec:2.3.1.85 synthase, ec:1.1.1.100 reductase, ec:3.1.2.21 hydrolase, ec:6.2.1.3 ligase, ec:2.3.1.39 Smalonyltransferase, ec:2.3.1.179 synthase II, ec:3.1.2.14 hydrolase, ec:2.3.1.41 synthase I	2, 5, 1, 1, 4, 1, 1, 2, 2	Sample3_Locus_8920_Transcript_2/3_Confidence_0.333_Length_2038, SampleTS1_Locus_1155_Transcript_11/16_Confidence_0.625_Length_2240, Sample3_Locus_24964_Transcript_1/1_Confidence_0.000_Length_1088, Sample6_Locus_6899_Transcript_8/13_Confidence_0.389_Length_1994, Sample6_Locus_8953_Transcript_13/14_Confidence_0.143_Length_4871, SampleTS3_Locus_3571_Transcript_8/10_Confidence_0.125_Length_1106, Sample6_Locus_10875_Transcript_2/10_Confidence_0.167_Length_1095, Sample6_Locus_8953_Transcript_13/14_Confidence_0.143_Length_4871, Sample6_Locus_10875_Transcript_2/10_Confidence_0.167_Length_1095, SampleTS1_Locus_369_Transcript_24/52_Confidence_0.312_Length_2199, SampleTS1_Locus_24343_Transcript_2/3_Confidence_0.333_Length_1375, Sample1_Locus_5442_Transcript_10/12_Confidence_0.091_Length_2073, Sample3_Locus_31478_Transcript_1/1_Confidence_0.333_Length_620, SampleTS3_Locus_3571_Transcript_8/10_Confidence_0.125_Length_1106, Sample3_Locus_24964_Transcript_1/1_Confidence_0.000_Length_1088, Sample6_Locus_6899_Transcript_8/13_Confidence_0.389_Length_1994, Sample6_Locus_10875_Transcript_2/10_Confidence_0.167_Length_1095, Sample3_Locus_24964_Transcript_1/1_Confidence_0.000_Length_1088, Sample6_Locus_8953_Transcript_13/14_Confidence_0.143_Length_4871

**Nicotinate and nicotinamide metabolism**	map00760	9	ec:3.6.1.9 diphosphatase, ec:4.1.3.32 lyase, ec:2.7.1.23 kinase, ec:2.7.7.1 adenylyltransferase, ec:3.6.1.22 diphosphatase, ec:1.4.3.16 oxidase, ec:2.7.7.18 adenylyltransferase, ec:1.6.1.1 transhydrogenase (Sispecific), ec:1.6.1.2 transhydrogenase (Re/Sispecific)	5, 1, 1, 1, 1, 1, 2, 4, 4	Sample6_Locus_1849_Transcript_4/6_Confidence_0.429_Length_866, Sample3_Locus_345_Transcript_6/8_Confidence_0.455_Length_1801, SampleTS3_Locus_5573_Transcript_4/5_Confidence_0.333_Length_940, Sample3_Locus_10699_Transcript_3/4_Confidence_0.400_Length_957, Sample3_Locus_27182_Transcript_2/3_Confidence_0.250_Length_1010, Sample6_Locus_3001_Transcript_26/36_Confidence_0.070_Length_1469, SampleTS1_Locus_12062_Transcript_1/1_Confidence_0.000_Length_1207 , SampleTS3_Locus_2424_Transcript_35/36_Confidence_0.000_Length_1314, Sample3_Locus_345_Transcript_6/8_Confidence_0.455_Length_1801, SampleTS1_Locus_1041_Transcript_1/5_Confidence_0.400_Length_2190, SampleTS2_Locus_1249_Transcript_11/12_Confidence_0.300_Length_1726, SampleTS3_Locus_2424_Transcript_35/36_Confidence_0.000_Length_1314, SampleTS3_Locus_5019_Transcript_18/23_Confidence_0.338_Length_1167, SampleTS1_Locus_1464_Transcript_2/3_Confidence_0.333_Length_2125, Sample3_Locus_1308_Transcript_4/13_Confidence_0.107_Length_1527, SampleTS1_Locus_1287_Transcript_1/3_Confidence_0.714_Length_4056, SampleTS3_Locus_5019_Transcript_18/23_Confidence_0.338_Length_1167, SampleTS1_Locus_1464_Transcript_2/3_Confidence_0.333_Length_2125, Sample3_Locus_1308_Transcript_4/13_Confidence_0.107_Length_1527, SampleTS1_Locus_1287_Transcript_1/3_Confidence_0.714_Length_4056

**Ascorbate and aldarate metabolism**	map00053	9	ec:2.7.7.64 uridylyltransferase, ec:2.7.7.44 uridylyltransferase, ec:1.2.1.3 dehydrogenase (NAD+), ec:1.11.1.11 peroxidase, ec:1.1.1.19 reductase, ec:1.1.1.22 6dehydrogenase, ec:2.4.1.17 1naphthol glucuronyltransferase, ec:5.1.3.18 3,5epimerase, ec:1.13.99.1 oxygenase	5, 1, 6, 2, 1, 2, 3, 1, 1	SampleTS3_Locus_2330_Transcript_4/4_Confidence_0.000_Length_1686, SampleTS1_Locus_16292_Transcript_4/5_Confidence_0.600_Length_1341, Sample3_Locus_4208_Transcript_8/15_Confidence_0.091_Length_2117, Sample3_Locus_40_Transcript_1/1_Confidence_0.000_Length_1702, Sample3_Locus_14770_Transcript_1/1_Confidence_0.000_Length_1819, SampleTS3_Locus_2330_Transcript_4/4_Confidence_0.000_Length_1686, SampleTS3_Locus_23038_Transcript_2/3_Confidence_0.333_Length_1864, SampleTS2_Locus_4874_Transcript_6/12_Confidence_0.158_Length_1042, Sample3_Locus_23176_Transcript_2/3_Confidence_0.333_Length_1282, SampleTS1_Locus_5373_Transcript_26/33_Confidence_0.065_Length_1620, Sample6_Locus_12695_Transcript_1/2_Confidence_0.333_Length_2218, SampleTS3_Locus_23264_Transcript_1/3_Confidence_0.500_Length_845, SampleTS1_Locus_6669_Transcript_3/3_Confidence_0.200_Length_979, SampleTS1_Locus_1282_Transcript_9/25_Confidence_0.318_Length_1204, SampleTS3_Locus_8149_Transcript_4/10_Confidence_0.118_Length_927, SampleTS3_Locus_1975_Transcript_19/64_Confidence_0.064_Length_546, SampleTS2_Locus_4_Transcript_35/36_Confidence_0.021_Length_1580, Sample6_Locus_9455_Transcript_3/4_Confidence_0.200_Length_1370, SampleTS1_Locus_4303_Transcript_9/11_Confidence_0.167_Length_1464, Sample6_Locus_8395_Transcript_14/17_Confidence_0.508_Length_1568, Sample3_Locus_22932_Transcript_1/1_Confidence_0.000_Length_1277, Sample3_Locus_12288_Transcript_5/8_Confidence_0.375_Length_1453

**Oxidative phosphorylation**	map00190	8	ec:2.7.4.1 kinase, ec:3.6.1.1 diphosphatase, ec:1.3.5.1 dehydrogenase, ec:3.6.3.6 ATPase, ec:1.9.3.1 oxidase, ec:1.6.5.3 reductase (H+translocating), ec:1.10.2.2 reductase, ec:1.6.99.3 dehydrogenase	1, 2, 1, 3, 3, 7, 2, 10	Sample6_Locus_14073_Transcript_9/19_Confidence_0.260_Length_829, SampleTS2_Locus_5041_Transcript_4/5_Confidence_0.500_Length_756, Sample6_Locus_485_Transcript_4/7_Confidence_0.400_Length_2774, SampleTS2_Locus_54_Transcript_4/10_Confidence_0.083_Length_1374, Sample3_Locus_450_Transcript_9/16_Confidence_0.476_Length_3424, Sample6_Locus_4460_Transcript_7/9_Confidence_0.417_Length_984, SampleTS1_Locus_1128_Transcript_3/3_Confidence_0.000_Length_491, SampleTS3_Locus_1840_Transcript_3/3_Confidence_0.000_Length_1701, SampleTS1_Locus_3847_Transcript_1/1_Confidence_0.000_Length_1612, SampleTS1_Locus_1911_Transcript_1/1_Confidence_0.000_Length_1757, SampleTS1_Locus_9950_Transcript_2/3_Confidence_0.667_Length_1116, SampleTS1_Locus_6265_Transcript_1/4_Confidence_0.200_Length_1674, Sample6_Locus_27578_Transcript_1/1_Confidence_0.000_Length_652, SampleTS2_Locus_12057_Transcript_2/4_Confidence_0.167_Length_2549, SampleTS3_Locus_3197_Transcript_1/1_Confidence_0.000_Length_435, SampleTS1_Locus_1582_Transcript_4/5_Confidence_0.400_Length_1930, SampleTS2_Locus_590_Transcript_3/3_Confidence_0.000_Length_1259, Sample1_Locus_11514_Transcript_9/11_Confidence_0.161_Length_1131, SampleTS2_Locus_3852_Transcript_3/5_Confidence_0.200_Length_807, SampleTS1_Locus_9016_Transcript_1/2_Confidence_0.333_Length_766, SampleTS1_Locus_9950_Transcript_2/3_Confidence_0.667_Length_1116, SampleTS1_Locus_6265_Transcript_1/4_Confidence_0.200_Length_1674, Sample6_Locus_27578_Transcript_1/1_Confidence_0.000_Length_652, SampleTS2_Locus_12057_Transcript_2/4_Confidence_0.167_Length_2549, SampleTS3_Locus_3197_Transcript_1/1_Confidence_0.000_Length_435, SampleTS1_Locus_1582_Transcript_4/5_Confidence_0.400_Length_1930, Sample6_Locus_2760_Transcript_3/6_Confidence_0.100_Length_1949, SampleTS2_Locus_590_Transcript_3/3_Confidence_0.000_Length_1259, SampleTS2_Locus_1483_Transcript_3/13_Confidence_0.364_Length_1620
**Biotin metabolism**	map00780	8	ec:2.8.1.6 synthase, ec:1.1.1.100 reductase, ec:2.3.1.179 synthase II, ec:6.3.4.10 (ATPhydrolysing)] ligase, ec:6.3.4.11 ligase, ec:6.3.4.15 ligase, ec:2.3.1.41 synthase I, ec:2.3.1.47 synthase	1, 1, 1, 1, 1, 1, 2, 2	SampleTS1_Locus_7045_Transcript_3/6_Confidence_0.286_Length_1062, Sample6_Locus_8953_Transcript_13/14_Confidence_0.143_Length_4871, Sample3_Locus_24964_Transcript_1/1_Confidence_0.000_Length_1088, SampleTS1_Locus_10161_Transcript_4/4_Confidence_0.000_Length_1433, SampleTS1_Locus_10161_Transcript_4/4_Confidence_0.000_Length_1433, SampleTS1_Locus_10161_Transcript_4/4_Confidence_0.000_Length_1433, Sample3_Locus_24964_Transcript_1/1_Confidence_0.000_Length_1088, Sample6_Locus_8953_Transcript_13/14_Confidence_0.143_Length_4871, Sample6_Locus_13557_Transcript_4/6_Confidence_0.200_Length_1530, SampleTS2_Locus_13131_Transcript_7/10_Confidence_0.083_Length_1731

**Biosynthesis of unsaturated fatty acids**	map01040	8	ec:1.1.1.100 reductase, ec:2.3.1.16 Cacyltransferase, ec:1.3.1.38 reductase (NADPH), ec:4.2.1.17 hydratase, ec:1.1.1.211 dehydrogenase, ec:1.14.19.3 6desaturase, ec:1.14.19.1 9desaturase, ec:1.3.3.6 oxidase	1, 1, 1, 2, 1, 1, 1, 2	Sample6_Locus_8953_Transcript_13/14_Confidence_0.143_Length_4871, SampleTS2_Locus_1750_Transcript_17/20_Confidence_0.182_Length_1568, SampleTS1_Locus_11137_Transcript_3/4_Confidence_0.500_Length_1746, SampleTS1_Locus_789_Transcript_9/11_Confidence_0.238_Length_2300, Sample3_Locus_5282_Transcript_2/7_Confidence_0.700_Length_1266, SampleTS3_Locus_11808_Transcript_9/11_Confidence_0.565_Length_1006, SampleTS2_Locus_20864_Transcript_2/3_Confidence_0.667_Length_860, SampleTS2_Locus_20864_Transcript_2/3_Confidence_0.667_Length_860, SampleTS2_Locus_1750_Transcript_17/20_Confidence_0.182_Length_1568, Sample3_Locus_6833_Transcript_1/1_Confidence_0.000_Length_1688
**Histidine metabolism**	map00340	8	ec:4.1.1.22 decarboxylase, ec:3.6.1.31 diphosphatase, ec:2.6.1.9 transaminase, ec:4.2.1.19 dehydratase, ec:1.2.1.5 dehydrogenase [NAD(P)+], ec:1.2.1.3 dehydrogenase (NAD+), ec:1.1.1.23 dehydrogenase, ec:3.5.4.19 cyclohydrolase	1, 3, 2, 1, 1, 6, 1, 3	Sample1_Locus_26122_Transcript_1/1_Confidence_0.000_Length_755, Sample6_Locus_2319_Transcript_10/15_Confidence_0.280_Length_1298, SampleTS2_Locus_7249_Transcript_3/5_Confidence_0.706_Length_812, SampleTS1_Locus_7721_Transcript_1/1_Confidence_0.000_Length_1610, Sample1_Locus_26802_Transcript_1/2_Confidence_0.667_Length_439, Sample6_Locus_13664_Transcript_1/2_Confidence_0.333_Length_862, Sample6_Locus_10525_Transcript_3/19_Confidence_0.244_Length_681, SampleTS1_Locus_5373_Transcript_26/33_Confidence_0.065_Length_1620, SampleTS3_Locus_23038_Transcript_2/3_Confidence_0.333_Length_1864, SampleTS2_Locus_4874_Transcript_6/12_Confidence_0.158_Length_1042, Sample3_Locus_23176_Transcript_2/3_Confidence_0.333_Length_1282, SampleTS1_Locus_5373_Transcript_26/33_Confidence_0.065_Length_1620, Sample6_Locus_12695_Transcript_1/2_Confidence_0.333_Length_2218, SampleTS3_Locus_23264_Transcript_1/3_Confidence_0.500_Length_845, SampleTS1_Locus_7721_Transcript_1/1_Confidence_0.000_Length_1610, Sample6_Locus_2319_Transcript_10/15_Confidence_0.280_Length_1298, SampleTS2_Locus_7249_Transcript_3/5_Confidence_0.706_Length_812, SampleTS1_Locus_7721_Transcript_1/1_Confidence_0.000_Length_1610

**Valine, leucine and isoleucine biosynthesis**	map00290	7	ec:2.3.3.13 synthase, ec:4.2.1.9 dehydratase, ec:2.2.1.6 synthase, ec:2.6.1.42 transaminase, ec:4.2.1.33 dehydratase, ec:1.1.1.86 reductoisomerase (NADP+), ec:1.1.1.85 dehydrogenase	1, 1, 1, 2, 1, 1, 1	Sample1_Locus_7894_Transcript_1/3_Confidence_0.250_Length_2075, Sample1_Locus_2272_Transcript_43/67_Confidence_0.374_Length_2859, Sample3_Locus_4596_Transcript_3/6_Confidence_0.400_Length_1157, Sample1_Locus_18777_Transcript_2/4_Confidence_0.923_Length_1416, Sample3_Locus_5952_Transcript_4/4_Confidence_0.000_Length_2586, Sample6_Locus_7492_Transcript_8/10_Confidence_0.105_Length_2216, Sample1_Locus_2001_Transcript_4/7_Confidence_0.600_Length_1686, SampleTS2_Locus_3967_Transcript_1/4_Confidence_0.667_Length_1445
**Aminobenzoate degradation**	map00627	7	ec:3.6.1.7 acetylphosphatase, ec:3.1.3.41 nitrophenyl phosphatase, ec:3.5.5.1 acetonitrilase, ec:4.2.1.84 hydratase, ec:2.8.3.8 CoAtransferase, ec:4.2.1.17 hydratase, ec:3.5.1.4 acylamidase	1, 66, 1, 1, 1, 2, 2	SampleTS3_Locus_13938_Transcript_1/2_Confidence_0.333_Length_655, SampleTS3_Locus_1236_Transcript_8/14_Confidence_0.379_Length_696, SampleTS1_Locus_1994_Transcript_14/23_Confidence_0.470_Length_2041, SampleTS1_Locus_5854_Transcript_5/5_Confidence_0.400_Length_861, Sample3_Locus_4609_Transcript_5/8_Confidence_0.444_Length_1430, SampleTS3_Locus_6160_Transcript_3/3_Confidence_0.167_Length_2057, SampleTS3_Locus_1630_Transcript_4/8_Confidence_0.500_Length_1635, SampleTS2_Locus_4720_Transcript_3/7_Confidence_0.667_Length_1404, SampleTS1_Locus_10554_Transcript_2/2_Confidence_0.000_Length_719, Sample3_Locus_3349_Transcript_2/6_Confidence_0.556_Length_1203, Sample6_Locus_2283_Transcript_7/17_Confidence_0.225_Length_1818, Sample3_Locus_5932_Transcript_3/4_Confidence_0.571_Length_501, SampleTS3_Locus_13512_Transcript_1/3_Confidence_0.333_Length_1321, SampleTS2_Locus_1774_Transcript_4/5_Confidence_0.500_Length_990, SampleTS2_Locus_549_Transcript_10/36_Confidence_0.207_Length_1084, Sample3_Locus_14090_Transcript_2/4_Confidence_0.714_Length_1345, Sample6_Locus_5727_Transcript_3/3_Confidence_0.000_Length_2301, SampleTS3_Locus_5284_Transcript_10/12_Confidence_0.450_Length_1002, SampleTS1_Locus_2055_Transcript_1/2_Confidence_0.667_Length_939, SampleTS2_Locus_8418_Transcript_9/12_Confidence_0.559_Length_1395, Sample3_Locus_3056_Transcript_6/6_Confidence_0.000_Length_1859, Sample3_Locus_13102_Transcript_1/2_Confidence_0.333_Length_1552, SampleTS3_Locus_9298_Transcript_1/10_Confidence_0.238_Length_793, Sample3_Locus_2217_Transcript_22/26_Confidence_0.080_Length_870, SampleTS1_Locus_2823_Transcript_2/10_Confidence_0.444_Length_2044, SampleTS3_Locus_13352_Transcript_4/6_Confidence_0.600_Length_2895, Sample6_Locus_4221_Transcript_5/15_Confidence_0.231_Length_1620, Sample3_Locus_23182_Transcript_4/4_Confidence_0.000_Length_3946, SampleTS2_Locus_4899_Transcript_2/5_Confidence_0.357_Length_658, Sample6_Locus_594_Transcript_17/21_Confidence_0.471_Length_4353, SampleTS1_Locus_4566_Transcript_4/4_Confidence_0.000_Length_2764, SampleTS2_Locus_2599_Transcript_3/9_Confidence_0.091_Length_4008, SampleTS1_Locus_397_Transcript_66/78_Confidence_0.022_Length_711, SampleTS1_Locus_26651_Transcript_1/1_Confidence_0.000_Length_1107, SampleTS3_Locus_12636_Transcript_2/2_Confidence_0.333_Length_583, Sample6_Locus_11896_Transcript_2/5_Confidence_0.500_Length_1410, SampleTS2_Locus_549_Transcript_23/36_Confidence_0.110_Length_766, SampleTS1_Locus_9567_Transcript_1/2_Confidence_0.667_Length_1197, SampleTS2_Locus_13566_Transcript_1/3_Confidence_0.750_Length_1476, Sample6_Locus_6071_Transcript_1/2_Confidence_0.333_Length_2182, Sample6_Locus_11496_Transcript_4/7_Confidence_0.250_Length_1518, Sample3_Locus_5257_Transcript_3/8_Confidence_0.333_Length_2904, Sample6_Locus_11565_Transcript_7/13_Confidence_0.097_Length_1502, Sample1_Locus_27168_Transcript_1/2_Confidence_1.000_Length_653, Sample6_Locus_24750_Transcript_4/9_Confidence_0.222_Length_785, SampleTS2_Locus_23786_Transcript_1/1_Confidence_0.000_Length_866, Sample3_Locus_10262_Transcript_4/5_Confidence_0.062_Length_1337, SampleTS2_Locus_2994_Transcript_7/9_Confidence_0.235_Length_2922, Sample6_Locus_10381_Transcript_4/5_Confidence_0.333_Length_1065, Sample3_Locus_2926_Transcript_2/3_Confidence_0.750_Length_1774,

					SampleTS3_Locus_23494_Transcript_1/1_Confidence_0.000_Length_1156, Sample3_Locus_23102_Transcript_1/2_Confidence_0.667_Length_1067, Sample6_Locus_8346_Transcript_7/13_Confidence_0.118_Length_1292, SampleTS1_Locus_12977_Transcript_4/6_Confidence_0.429_Length_1160, SampleTS1_Locus_6027_Transcript_7/14_Confidence_0.160_Length_504, Sample3_Locus_22654_Transcript_1/1_Confidence_0.000_Length_1356, Sample6_Locus_1159_Transcript_30/32_Confidence_0.027_Length_1920, SampleTS3_Locus_2277_Transcript_3/5_Confidence_0.400_Length_1847, SampleTS1_Locus_30500_Transcript_2/2_Confidence_0.850_Length_548, Sample1_Locus_18291_Transcript_1/1_Confidence_0.000_Length_647, Sample6_Locus_30120_Transcript_2/3_Confidence_0.667_Length_1321, SampleTS2_Locus_3992_Transcript_15/16_Confidence_0.143_Length_1808, SampleTS1_Locus_11573_Transcript_5/10_Confidence_0.211_Length_1117, Sample1_Locus_25057_Transcript_2/6_Confidence_0.727_Length_1459, SampleTS2_Locus_549_Transcript_22/36_Confidence_0.134_Length_768, Sample3_Locus_2632_Transcript_1/1_Confidence_0.000_Length_1846, SampleTS3_Locus_11395_Transcript_5/6_Confidence_0.111_Length_2047, Sample3_Locus_27182_Transcript_2/3_Confidence_0.250_Length_1010, Sample3_Locus_12929_Transcript_1/1_Confidence_0.000_Length_1898, SampleTS2_Locus_1750_Transcript_17/20_Confidence_0.182_Length_1568, SampleTS1_Locus_789_Transcript_9/11_Confidence_0.238_Length_2300, Sample3_Locus_5282_Transcript_2/7_Confidence_0.700_Length_1266, Sample6_Locus_14763_Transcript_11/12_Confidence_0.182_Length_1117, Sample6_Locus_19655_Transcript_1/3_Confidence_0.667_Length_978
**Inositol phosphate metabolism**	map00562	7	ec:2.7.1.127 3kinase, ec:2.7.1.67 4kinase, ec:2.7.1.68 5kinase, ec:3.1.3.36 5phosphatase, ec:1.2.1.18 dehydrogenase (acetylating), ec:2.7.1.137 3kinase, ec:1.13.99.1 oxygenase	1, 4, 3, 1, 1, 1, 1	SampleTS2_Locus_1265_Transcript_3/9_Confidence_0.588_Length_949, Sample1_Locus_23868_Transcript_1/1_Confidence_0.000_Length_3026, Sample6_Locus_8424_Transcript_5/6_Confidence_0.143_Length_3891, SampleTS1_Locus_4904_Transcript_5/8_Confidence_0.381_Length_1961, SampleTS1_Locus_4990_Transcript_6/7_Confidence_0.056_Length_1270, Sample6_Locus_12499_Transcript_26/36_Confidence_0.183_Length_587, Sample6_Locus_16639_Transcript_4/7_Confidence_0.143_Length_2715, Sample1_Locus_24371_Transcript_4/4_Confidence_0.000_Length_805, SampleTS3_Locus_5284_Transcript_10/12_Confidence_0.450_Length_1002, SampleTS3_Locus_23038_Transcript_2/3_Confidence_0.333_Length_1864, SampleTS3_Locus_5967_Transcript_6/11_Confidence_0.091_Length_1939, Sample3_Locus_12288_Transcript_5/8_Confidence_0.375_Length_1453

**Lysine degradation**	map00310	7	ec:2.3.1.61 succinyltransferase, ec:2.1.1.43 Nmethyltransferase, ec:4.2.1.17 hydratase, ec:1.2.1.3 dehydrogenase (NAD+), ec:2.3.1.9 Cacetyltransferase, ec:1.1.1.35 dehydrogenase, ec:1.2.4.2 dehydrogenase (succinyltransferring)	1, 23, 2, 6, 1, 3, 1	Sample3_Locus_4770_Transcript_12/16_Confidence_0.420_Length_1753, SampleTS1_Locus_7466_Transcript_1/2_Confidence_0.333_Length_1169, Sample3_Locus_6969_Transcript_6/8_Confidence_0.762_Length_870, Sample6_Locus_2648_Transcript_9/13_Confidence_0.414_Length_1634, Sample6_Locus_1130_Transcript_35/37_Confidence_0.057_Length_983, Sample6_Locus_2189_Transcript_8/13_Confidence_0.500_Length_5625, SampleTS2_Locus_4573_Transcript_1/3_Confidence_0.667_Length_881, SampleTS3_Locus_12905_Transcript_2/2_Confidence_0.000_Length_914, SampleTS1_Locus_8400_Transcript_9/12_Confidence_0.536_Length_2582, SampleTS1_Locus_15017_Transcript_4/4_Confidence_0.000_Length_1042, SampleTS3_Locus_6812_Transcript_4/4_Confidence_0.000_Length_3723, SampleTS1_Locus_4326_Transcript_3/6_Confidence_0.125_Length_778, SampleTS2_Locus_2512_Transcript_4/10_Confidence_0.222_Length_1103, SampleTS1_Locus_25422_Transcript_1/1_Confidence_0.000_Length_1767, Sample3_Locus_3137_Transcript_7/8_Confidence_0.071_Length_1158, Sample1_Locus_12494_Transcript_4/6_Confidence_0.333_Length_5696, SampleTS1_Locus_9947_Transcript_4/10_Confidence_0.417_Length_1541, SampleTS3_Locus_10906_Transcript_5/5_Confidence_0.000_Length_3210, Sample6_Locus_6056_Transcript_4/7_Confidence_0.278_Length_663, SampleTS3_Locus_1517_Transcript_6/8_Confidence_0.429_Length_1534, Sample3_Locus_12500_Transcript_5/16_Confidence_0.086_Length_1337, SampleTS1_Locus_2952_Transcript_3/7_Confidence_0.444_Length_2999, Sample6_Locus_3327_Transcript_10/12_Confidence_0.364_Length_5452, Sample3_Locus_7031_Transcript_2/6_Confidence_0.333_Length_4210, SampleTS1_Locus_789_Transcript_9/11_Confidence_0.238_Length_2300, Sample3_Locus_5282_Transcript_2/7_Confidence_0.700_Length_1266, SampleTS3_Locus_23038_Transcript_2/3_Confidence_0.333_Length_1864, SampleTS2_Locus_4874_Transcript_6/12_Confidence_0.158_Length_1042, Sample3_Locus_23176_Transcript_2/3_Confidence_0.333_Length_1282, SampleTS1_Locus_5373_Transcript_26/33_Confidence_0.065_Length_1620, Sample6_Locus_12695_Transcript_1/2_Confidence_0.333_Length_2218, SampleTS3_Locus_23264_Transcript_1/3_Confidence_0.500_Length_845, SampleTS2_Locus_1750_Transcript_17/20_Confidence_0.182_Length_1568, SampleTS1_Locus_789_Transcript_9/11_Confidence_0.238_Length_2300, SampleTS3_Locus_1827_Transcript_3/3_Confidence_0.000_Length_1033, SampleTS3_Locus_11808_Transcript_9/11_Confidence_0.565_Length_1006, SampleTS3_Locus_20806_Transcript_1/1_Confidence_0.000_Length_3106
**One carbon pool by folate**	map00670	7	ec:6.3.3.2 cycloligase, ec:2.1.2.2 formyltransferase, ec:2.1.2.1 hydroxymethyltransferase, ec:3.5.4.9 cyclohydrolase, ec:2.1.1.13 synthase, ec:3.5.1.10 deformylase, ec:1.5.1.5 dehydrogenase (NADP+)	2, 1, 2, 1, 1, 2, 1	SampleTS2_Locus_9480_Transcript_1/1_Confidence_0.000_Length_1500, Sample3_Locus_17995_Transcript_3/4_Confidence_0.200_Length_1095, SampleTS2_Locus_12481_Transcript_10/11_Confidence_0.194_Length_1103, Sample1_Locus_3932_Transcript_7/11_Confidence_0.400_Length_1725, SampleTS1_Locus_10618_Transcript_3/4_Confidence_0.400_Length_1517, Sample6_Locus_4997_Transcript_3/6_Confidence_0.429_Length_1455, SampleTS1_Locus_8020_Transcript_2/4_Confidence_0.333_Length_2411, SampleTS2_Locus_12481_Transcript_10/11_Confidence_0.194_Length_1103, SampleTS1_Locus_27797_Transcript_4/4_Confidence_0.800_Length_878, Sample6_Locus_4997_Transcript_3/6_Confidence_0.429_Length_1455

**Cyanoamino acid metabolism**	map00460	7	ec:2.1.2.1 hydroxymethyltransferase, ec:1.14.13.39 synthase (NADPH), ec:6.3.1.1 ligase, ec:3.5.5.1 acetonitrilase, ec:4.4.1.9 synthase, ec:2.3.2.2 glutamyl transpeptidase, ec:3.2.1.21 gentiobiase	2, 1, 2, 1, 2, 1, 2	Sample1_Locus_3932_Transcript_7/11_Confidence_0.400_Length_1725, SampleTS1_Locus_10618_Transcript_3/4_Confidence_0.400_Length_1517, Sample1_Locus_25642_Transcript_6/7_Confidence_0.500_Length_1752, Sample6_Locus_5745_Transcript_6/10_Confidence_0.065_Length_1149, Sample6_Locus_7334_Transcript_8/13_Confidence_0.571_Length_1873, Sample3_Locus_27182_Transcript_2/3_Confidence_0.250_Length_1010, Sample6_Locus_5355_Transcript_5/8_Confidence_0.577_Length_1830, SampleTS2_Locus_2923_Transcript_3/4_Confidence_0.333_Length_1277, Sample1_Locus_19449_Transcript_1/1_Confidence_0.000_Length_1704, Sample1_Locus_25240_Transcript_4/4_Confidence_0.429_Length_2225, SampleTS2_Locus_9286_Transcript_1/1_Confidence_0.000_Length_2119
**Tropane, piperidine and pyridine alkaloid biosynthesis**	map00960	6	ec:2.6.1.57 transaminase, ec:2.6.1.58 transaminase, ec:2.6.1.5 transaminase, ec:2.6.1.9 transaminase, ec:2.6.1.1 transaminase, ec:1.4.3.21 oxidase	2, 1, 2, 2, 3, 1	Sample1_Locus_26802_Transcript_1/2_Confidence_0.667_Length_439, Sample6_Locus_13664_Transcript_1/2_Confidence_0.333_Length_862, Sample6_Locus_22798_Transcript_3/3_Confidence_0.250_Length_666, Sample1_Locus_26802_Transcript_1/2_Confidence_0.667_Length_439, Sample6_Locus_13664_Transcript_1/2_Confidence_0.333_Length_862, Sample1_Locus_26802_Transcript_1/2_Confidence_0.667_Length_439, Sample6_Locus_13664_Transcript_1/2_Confidence_0.333_Length_862, Sample1_Locus_26802_Transcript_1/2_Confidence_0.667_Length_439, Sample6_Locus_13664_Transcript_1/2_Confidence_0.333_Length_862, SampleTS2_Locus_6646_Transcript_2/2_Confidence_0.333_Length_1520, Sample1_Locus_4359_Transcript_11/14_Confidence_0.314_Length_2540

**Phosphatidylinositol signaling system**	map04070	6	ec:2.7.1.127 3kinase, ec:2.7.1.67 4kinase, ec:2.7.1.68 5kinase, ec:3.1.3.36 5phosphatase, ec:2.7.1.137 3kinase, ec:2.7.11.13 kinase C	1, 4, 3, 1, 1, 1	SampleTS2_Locus_1265_Transcript_3/9_Confidence_0.588_Length_949, Sample1_Locus_23868_Transcript_1/1_Confidence_0.000_Length_3026, Sample6_Locus_8424_Transcript_5/6_Confidence_0.143_Length_3891, SampleTS1_Locus_4904_Transcript_5/8_Confidence_0.381_Length_1961, SampleTS1_Locus_4990_Transcript_6/7_Confidence_0.056_Length_1270, Sample6_Locus_12499_Transcript_26/36_Confidence_0.183_Length_587, Sample6_Locus_16639_Transcript_4/7_Confidence_0.143_Length_2715, Sample1_Locus_24371_Transcript_4/4_Confidence_0.000_Length_805, SampleTS3_Locus_5284_Transcript_10/12_Confidence_0.450_Length_1002, SampleTS3_Locus_5967_Transcript_6/11_Confidence_0.091_Length_1939, SampleTS2_Locus_4456_Transcript_5/7_Confidence_0.333_Length_2807
**Benzoate degradation**	map00362	6	ec:2.3.1.16 Cacyltransferase, ec:1.2.1.10 dehydrogenase (acetylating), ec:1.1.1.157 dehydrogenase, ec:4.2.1.17 hydratase, ec:2.3.1.9 Cacetyltransferase, ec:1.1.1.35 dehydrogenase	1, 2, 1, 2, 1, 3	SampleTS2_Locus_1750_Transcript_17/20_Confidence_0.182_Length_1568, SampleTS3_Locus_400_Transcript_6/29_Confidence_0.422_Length_2479, Sample6_Locus_1524_Transcript_26/30_Confidence_0.185_Length_3215, SampleTS3_Locus_1827_Transcript_3/3_Confidence_0.000_Length_1033, SampleTS1_Locus_789_Transcript_9/11_Confidence_0.238_Length_2300, Sample3_Locus_5282_Transcript_2/7_Confidence_0.700_Length_1266, SampleTS2_Locus_1750_Transcript_17/20_Confidence_0.182_Length_1568, SampleTS1_Locus_789_Transcript_9/11_Confidence_0.238_Length_2300, SampleTS3_Locus_1827_Transcript_3/3_Confidence_0.000_Length_1033, SampleTS3_Locus_11808_Transcript_9/11_Confidence_0.565_Length_1006
**Fatty acid elongation**	map00062	6	ec:2.3.1.16 Cacyltransferase, ec:4.2.1.74 hydratase, ec:1.3.1.38 reductase (NADPH), ec:4.2.1.17 hydratase, ec:1.1.1.211 dehydrogenase, ec:1.1.1.35 dehydrogenase	1, 1, 1, 2, 1, 3	SampleTS2_Locus_1750_Transcript_17/20_Confidence_0.182_Length_1568, SampleTS1_Locus_789_Transcript_9/11_Confidence_0.238_Length_2300, SampleTS1_Locus_11137_Transcript_3/4_Confidence_0.500_Length_1746, SampleTS1_Locus_789_Transcript_9/11_Confidence_0.238_Length_2300, Sample3_Locus_5282_Transcript_2/7_Confidence_0.700_Length_1266, SampleTS3_Locus_11808_Transcript_9/11_Confidence_0.565_Length_1006, SampleTS1_Locus_789_Transcript_9/11_Confidence_0.238_Length_2300, SampleTS3_Locus_1827_Transcript_3/3_Confidence_0.000_Length_1033, SampleTS3_Locus_11808_Transcript_9/11_Confidence_0.565_Length_1006

**Isoquinoline alkaloid biosynthesis**	map00950	6	ec:1.4.3.2 oxidase, ec:2.6.1.57 transaminase, ec:2.6.1.5 transaminase, ec:2.6.1.1 transaminase, ec:1.14.16.2 3monooxygenase, ec:1.4.3.21 oxidase	1, 2, 2, 3, 1, 1	SampleTS1_Locus_1041_Transcript_1/5_Confidence_0.400_Length_2190, Sample1_Locus_26802_Transcript_1/2_Confidence_0.667_Length_439, Sample6_Locus_13664_Transcript_1/2_Confidence_0.333_Length_862, Sample1_Locus_26802_Transcript_1/2_Confidence_0.667_Length_439, Sample6_Locus_13664_Transcript_1/2_Confidence_0.333_Length_862, Sample1_Locus_26802_Transcript_1/2_Confidence_0.667_Length_439, Sample6_Locus_13664_Transcript_1/2_Confidence_0.333_Length_862, SampleTS2_Locus_6646_Transcript_2/2_Confidence_0.333_Length_1520, Sample3_Locus_2321_Transcript_7/9_Confidence_0.414_Length_1007, Sample1_Locus_4359_Transcript_11/14_Confidence_0.314_Length_2540
**NGlycan biosynthesis**	map00510	5	ec:2.4.1.101 2betaNacetylglucosaminyltransferase, ec:2.4.1.131 alpha1,2mannosyltransferase, ec:2.4.1.260 alpha1,6mannosyltransferase, ec:2.4.1.141 Nacetylglucosaminyltransferase, ec:2.4.99.1 alpha(2,6)sialyltransferase	1, 1, 1, 1, 14	Sample3_Locus_2392_Transcript_15/22_Confidence_0.229_Length_3092, SampleTS2_Locus_15690_Transcript_1/2_Confidence_0.333_Length_636, Sample3_Locus_21096_Transcript_1/4_Confidence_0.400_Length_1431, Sample3_Locus_19481_Transcript_3/6_Confidence_0.500_Length_863, Sample6_Locus_19814_Transcript_10/10_Confidence_0.483_Length_1044, Sample3_Locus_19889_Transcript_1/1_Confidence_0.000_Length_1128, SampleTS2_Locus_4156_Transcript_29/43_Confidence_0.097_Length_1215, SampleTS2_Locus_5330_Transcript_4/5_Confidence_0.429_Length_1628, SampleTS1_Locus_13929_Transcript_3/7_Confidence_0.091_Length_1486, Sample6_Locus_5906_Transcript_9/11_Confidence_0.333_Length_2015, Sample6_Locus_6485_Transcript_12/18_Confidence_0.490_Length_1417, Sample1_Locus_19212_Transcript_2/4_Confidence_0.333_Length_2192, Sample3_Locus_191_Transcript_86/118_Confidence_0.018_Length_2298, Sample3_Locus_12595_Transcript_4/8_Confidence_0.364_Length_2480, SampleTS1_Locus_993_Transcript_3/9_Confidence_0.083_Length_1910, Sample3_Locus_25591_Transcript_4/4_Confidence_0.000_Length_1038, SampleTS2_Locus_11455_Transcript_4/10_Confidence_0.630_Length_1058, SampleTS1_Locus_21367_Transcript_2/3_Confidence_0.429_Length_1441

**Metabolism of xenobiotics by cytochrome P450**	map00980	5	ec:1.1.1.184 reductase (NADPH), ec:2.5.1.18 transferase, ec:1.1.1.1 dehydrogenase, ec:1.2.1.5 dehydrogenase [NAD(P)+], ec:2.4.1.17 1naphthol glucuronyltransferase	1, 6, 5, 1, 3	Sample6_Locus_15547_Transcript_5/8_Confidence_0.545_Length_1332, SampleTS1_Locus_6640_Transcript_2/7_Confidence_0.357_Length_1361, Sample6_Locus_795_Transcript_19/29_Confidence_0.175_Length_1113, Unigene1242_Br_gene19, SampleTS2_Locus_4937_Transcript_4/7_Confidence_0.444_Length_827, Sample3_Locus_2715_Transcript_4/6_Confidence_0.286_Length_835, SampleTS1_Locus_23227_Transcript_3/3_Confidence_0.000_Length_1280, SampleTS2_Locus_4874_Transcript_6/12_Confidence_0.158_Length_1042, Sample6_Locus_1524_Transcript_26/30_Confidence_0.185_Length_3215, SampleTS2_Locus_263_Transcript_1/3_Confidence_0.250_Length_2184, SampleTS3_Locus_400_Transcript_6/29_Confidence_0.422_Length_2479, SampleTS3_Locus_23264_Transcript_1/3_Confidence_0.500_Length_845, SampleTS1_Locus_5373_Transcript_26/33_Confidence_0.065_Length_1620, Sample6_Locus_9455_Transcript_3/4_Confidence_0.200_Length_1370, SampleTS1_Locus_4303_Transcript_9/11_Confidence_0.167_Length_1464, Sample6_Locus_8395_Transcript_14/17_Confidence_0.508_Length_1568
**Selenocompound metabolism**	map00450	5	ec:2.7.7.4 adenylyltransferase, ec:1.8.1.9 reductase, ec:2.1.1.13 synthase, ec:4.4.1.1 gammalyase, ec:6.1.1.10 ligase	3, 5, 1, 2, 1	SampleTS3_Locus_4546_Transcript_3/8_Confidence_0.429_Length_1895, SampleTS3_Locus_3403_Transcript_3/4_Confidence_0.333_Length_1292, SampleTS1_Locus_25259_Transcript_1/2_Confidence_0.333_Length_1099, SampleTS2_Locus_3616_Transcript_1/2_Confidence_0.333_Length_1862, Sample1_Locus_18476_Transcript_2/2_Confidence_0.000_Length_1075, Sample1_Locus_3593_Transcript_1/6_Confidence_0.250_Length_1874, Sample6_Locus_711_Transcript_5/8_Confidence_0.500_Length_1354, SampleTS1_Locus_5852_Transcript_11/12_Confidence_0.615_Length_1704, SampleTS1_Locus_8020_Transcript_2/4_Confidence_0.333_Length_2411, Sample6_Locus_5355_Transcript_5/8_Confidence_0.577_Length_1830, SampleTS2_Locus_2923_Transcript_3/4_Confidence_0.333_Length_1277, SampleTS1_Locus_1827_Transcript_15/16_Confidence_0.043_Length_2672

**alphaLinolenic acid metabolism**	map00592	5	ec:2.3.1.16 Cacyltransferase, ec:3.1.1.4 A2, ec:1.1.1.1 dehydrogenase, ec:4.2.1.17 hydratase, ec:1.3.3.6 oxidase	1, 4, 5, 2, 2	SampleTS2_Locus_1750_Transcript_17/20_Confidence_0.182_Length_1568, SampleTS1_Locus_587_Transcript_4/24_Confidence_0.067_Length_950, SampleTS2_Locus_23527_Transcript_1/1_Confidence_0.000_Length_1031, SampleTS1_Locus_11100_Transcript_2/5_Confidence_0.286_Length_1260, SampleTS3_Locus_8220_Transcript_1/4_Confidence_0.667_Length_1081, SampleTS2_Locus_4874_Transcript_6/12_Confidence_0.158_Length_1042, Sample6_Locus_1524_Transcript_26/30_Confidence_0.185_Length_3215, SampleTS2_Locus_263_Transcript_1/3_Confidence_0.250_Length_2184, SampleTS3_Locus_400_Transcript_6/29_Confidence_0.422_Length_2479, SampleTS3_Locus_23264_Transcript_1/3_Confidence_0.500_Length_845, SampleTS1_Locus_789_Transcript_9/11_Confidence_0.238_Length_2300, Sample3_Locus_5282_Transcript_2/7_Confidence_0.700_Length_1266, SampleTS2_Locus_1750_Transcript_17/20_Confidence_0.182_Length_1568, Sample3_Locus_6833_Transcript_1/1_Confidence_0.000_Length_1688
**Sphingolipid metabolism**	map00600	5	ec:2.3.1.24 Nacyltransferase, ec:3.1.3.4 phosphatase, ec:2.8.2.11 sulfotransferase, ec:3.2.1.22 melibiase, ec:3.2.1.23 lactase (ambiguous)	2, 2, 1, 1, 1	SampleTS1_Locus_703_Transcript_3/4_Confidence_0.333_Length_1395, Sample3_Locus_4289_Transcript_3/4_Confidence_0.333_Length_3187, Sample1_Locus_2193_Transcript_40/42_Confidence_0.262_Length_1445, SampleTS2_Locus_23786_Transcript_1/1_Confidence_0.000_Length_866, Sample1_Locus_565_Transcript_2/8_Confidence_0.636_Length_882, SampleTS2_Locus_3316_Transcript_5/7_Confidence_0.211_Length_3110, SampleTS2_Locus_9286_Transcript_1/1_Confidence_0.000_Length_2119

**Drug metabolism cytochrome P450**	map00982	5	ec:2.5.1.18 transferase, ec:1.1.1.1 dehydrogenase, ec:1.14.13.8 monooxygenase, ec:1.2.1.5 dehydrogenase [NAD(P)+], ec:2.4.1.17 1naphthol glucuronyltransferase	6, 5, 2, 1, 3	SampleTS1_Locus_6640_Transcript_2/7_Confidence_0.357_Length_1361, Sample6_Locus_795_Transcript_19/29_Confidence_0.175_Length_1113, Unigene1242_Br_gene19, SampleTS2_Locus_4937_Transcript_4/7_Confidence_0.444_Length_827, Sample3_Locus_2715_Transcript_4/6_Confidence_0.286_Length_835, SampleTS1_Locus_23227_Transcript_3/3_Confidence_0.000_Length_1280, SampleTS2_Locus_4874_Transcript_6/12_Confidence_0.158_Length_1042, Sample6_Locus_1524_Transcript_26/30_Confidence_0.185_Length_3215, SampleTS2_Locus_263_Transcript_1/3_Confidence_0.250_Length_2184, SampleTS3_Locus_400_Transcript_6/29_Confidence_0.422_Length_2479, SampleTS3_Locus_23264_Transcript_1/3_Confidence_0.500_Length_845, Sample3_Locus_10285_Transcript_1/4_Confidence_0.667_Length_836, SampleTS1_Locus_25099_Transcript_1/1_Confidence_0.000_Length_629, SampleTS1_Locus_5373_Transcript_26/33_Confidence_0.065_Length_1620, Sample6_Locus_9455_Transcript_3/4_Confidence_0.200_Length_1370, SampleTS1_Locus_4303_Transcript_9/11_Confidence_0.167_Length_1464, Sample6_Locus_8395_Transcript_14/17_Confidence_0.508_Length_1568
**Monobactam biosynthesis**	map00261	4	ec:2.7.2.4 kinase, ec:2.7.7.4 adenylyltransferase, ec:1.17.1.8 reductase, ec:4.3.3.7 synthase	1, 3, 1, 1	Sample3_Locus_2031_Transcript_1/1_Confidence_0.000_Length_2262, SampleTS3_Locus_4546_Transcript_3/8_Confidence_0.429_Length_1895, SampleTS3_Locus_3403_Transcript_3/4_Confidence_0.333_Length_1292, SampleTS1_Locus_25259_Transcript_1/2_Confidence_0.333_Length_1099, Sample6_Locus_3108_Transcript_12/12_Confidence_0.019_Length_1297, SampleTS1_Locus_10980_Transcript_1/3_Confidence_0.667_Length_1432

**Glycosphingo lipid biosynthesis ganglio series**	map00604	4	ec:2.4.99.7 6alphasialyltransferase, ec:2.4.99.4 alpha2,3sialyltransferase, ec:2.4.99.8 alpha2,8sialyltransferase, ec:3.2.1.23 lactase (ambiguous)	1, 10, 3, 1	Sample6_Locus_28634_Transcript_1/2_Confidence_0.333_Length_832, Sample6_Locus_1667_Transcript_21/40_Confidence_0.198_Length_1348, Sample6_Locus_6485_Transcript_12/18_Confidence_0.490_Length_1417, Sample6_Locus_22436_Transcript_2/2_Confidence_0.333_Length_1831, Sample3_Locus_191_Transcript_86/118_Confidence_0.018_Length_2298, Sample3_Locus_19889_Transcript_1/1_Confidence_0.000_Length_1128, SampleTS2_Locus_4156_Transcript_29/43_Confidence_0.097_Length_1215, SampleTS1_Locus_10921_Transcript_5/7_Confidence_0.444_Length_843, Sample6_Locus_28634_Transcript_1/2_Confidence_0.333_Length_832, SampleTS1_Locus_13929_Transcript_3/7_Confidence_0.091_Length_1486, SampleTS1_Locus_6551_Transcript_6/13_Confidence_0.111_Length_1643, Sample6_Locus_6485_Transcript_12/18_Confidence_0.490_Length_1417, Sample6_Locus_14905_Transcript_12/13_Confidence_0.077_Length_1757, Sample6_Locus_28634_Transcript_1/2_Confidence_0.333_Length_832, SampleTS2_Locus_9286_Transcript_1/1_Confidence_0.000_Length_2119
**Styrene degradation**	map00643	4	ec:3.7.1.2 betadiketonase, ec:3.5.5.1 acetonitrilase, ec:4.2.1.84 hydratase, ec:3.5.1.4 acylamidase	1, 1, 1, 2	SampleTS1_Locus_6929_Transcript_8/20_Confidence_0.293_Length_1407, Sample3_Locus_27182_Transcript_2/3_Confidence_0.250_Length_1010, Sample3_Locus_12929_Transcript_1/1_Confidence_0.000_Length_1898, Sample6_Locus_14763_Transcript_11/12_Confidence_0.182_Length_1117, Sample6_Locus_19655_Transcript_1/3_Confidence_0.667_Length_978
**Novobiocin biosynthesis**	map00401	4	ec:2.6.1.57 transaminase, ec:2.6.1.5 transaminase, ec:2.6.1.9 transaminase, ec:2.6.1.1 transaminase	2, 2, 2, 3	Sample1_Locus_26802_Transcript_1/2_Confidence_0.667_Length_439, Sample6_Locus_13664_Transcript_1/2_Confidence_0.333_Length_862, Sample1_Locus_26802_Transcript_1/2_Confidence_0.667_Length_439, Sample6_Locus_13664_Transcript_1/2_Confidence_0.333_Length_862, Sample1_Locus_26802_Transcript_1/2_Confidence_0.667_Length_439, Sample6_Locus_13664_Transcript_1/2_Confidence_0.333_Length_862, Sample1_Locus_26802_Transcript_1/2_Confidence_0.667_Length_439, Sample6_Locus_13664_Transcript_1/2_Confidence_0.333_Length_862, SampleTS2_Locus_6646_Transcript_2/2_Confidence_0.333_Length_1520

**Lysine biosynthesis**	map00300	4	ec:2.6.1.57 transaminase, ec:2.7.2.4 kinase, ec:1.17.1.8 reductase, ec:4.3.3.7 synthase	2, 1, 1, 1	Sample1_Locus_26802_Transcript_1/2_Confidence_0.667_Length_439, Sample6_Locus_13664_Transcript_1/2_Confidence_0.333_Length_862, Sample3_Locus_2031_Transcript_1/1_Confidence_0.000_Length_2262, Sample6_Locus_3108_Transcript_12/12_Confidence_0.019_Length_1297, SampleTS1_Locus_10980_Transcript_1/3_Confidence_0.667_Length_1432
**Ubiquinone and other terpenoidquinone biosynthesis**	map00130	4	ec:1.13.11.27 dioxygenase, ec:2.1.1.95 Omethyltransferase, ec:2.6.1.5 transaminase, ec:2.1.1.295 methyltransferase	3, 1, 2, 1	SampleTS1_Locus_605_Transcript_11/25_Confidence_0.258_Length_787, SampleTS2_Locus_361_Transcript_3/4_Confidence_0.750_Length_304, SampleTS1_Locus_605_Transcript_3/25_Confidence_0.161_Length_805, SampleTS1_Locus_8489_Transcript_1/1_Confidence_0.000_Length_1204, Sample1_Locus_26802_Transcript_1/2_Confidence_0.667_Length_439, Sample6_Locus_13664_Transcript_1/2_Confidence_0.333_Length_862, SampleTS1_Locus_23650_Transcript_3/3_Confidence_0.000_Length_1330
**Steroid biosynthesis**	map00100	4	ec:2.5.1.21 synthase, ec:3.1.1.13 esterase, ec:1.3.1.21 reductase, ec:1.14.13.70 14alphademethylase	1, 1, 1, 1	Sample6_Locus_901_Transcript_6/8_Confidence_0.643_Length_1628, SampleTS2_Locus_2263_Transcript_6/8_Confidence_0.429_Length_1743, Sample6_Locus_2751_Transcript_28/34_Confidence_0.070_Length_1735, SampleTS2_Locus_4266_Transcript_1/3_Confidence_0.333_Length_1522
**Streptomycin biosynthesis**	map00521	4	ec:2.7.1.2 glucokinase (phosphorylating), ec:2.7.1.1 hexokinase type IV glucokinase, ec:1.1.1.133 reductase, ec:5.1.3.13 3,5epimerase	1, 1, 2, 1	Sample6_Locus_5095_Transcript_5/8_Confidence_0.300_Length_1241, Sample6_Locus_5095_Transcript_5/8_Confidence_0.300_Length_1241, Sample3_Locus_2806_Transcript_6/14_Confidence_0.167_Length_1197, SampleTS2_Locus_1881_Transcript_15/19_Confidence_0.439_Length_912, Sample3_Locus_2806_Transcript_6/14_Confidence_0.167_Length_1197
**Various types of Nglycan biosynthesis**	map00513	4	ec:2.4.1.101 2betaNacetylglucosaminyltransferase, ec:2.4.1.131 alpha1,2mannosyltransferase, ec:2.4.1.260 alpha1,6mannosyltransferase, ec:2.4.1.141 Nacetylglucosaminyltransferase	1, 1, 1, 1	Sample3_Locus_2392_Transcript_15/22_Confidence_0.229_Length_3092, SampleTS2_Locus_15690_Transcript_1/2_Confidence_0.333_Length_636, Sample3_Locus_21096_Transcript_1/4_Confidence_0.400_Length_1431, Sample3_Locus_19481_Transcript_3/6_Confidence_0.500_Length_863

**Retinol metabolism**	map00830	4	ec:1.3.99.23 13,14reductase, ec:1.1.1.105 dehydrogenase (NAD+), ec:1.1.1.1 dehydrogenase, ec:2.4.1.17 1naphthol glucuronyltransferase	2, 3, 5, 3	SampleTS1_Locus_3615_Transcript_4/6_Confidence_0.444_Length_1164, Sample6_Locus_636_Transcript_7/7_Confidence_0.000_Length_955, Sample3_Locus_27117_Transcript_1/3_Confidence_0.250_Length_685, Sample6_Locus_6954_Transcript_4/5_Confidence_0.500_Length_1201, SampleTS1_Locus_10087_Transcript_6/7_Confidence_0.167_Length_948, SampleTS2_Locus_4874_Transcript_6/12_Confidence_0.158_Length_1042, Sample6_Locus_1524_Transcript_26/30_Confidence_0.185_Length_3215, SampleTS2_Locus_263_Transcript_1/3_Confidence_0.250_Length_2184, SampleTS3_Locus_400_Transcript_6/29_Confidence_0.422_Length_2479, SampleTS3_Locus_23264_Transcript_1/3_Confidence_0.500_Length_845, Sample6_Locus_9455_Transcript_3/4_Confidence_0.200_Length_1370, SampleTS1_Locus_4303_Transcript_9/11_Confidence_0.167_Length_1464, Sample6_Locus_8395_Transcript_14/17_Confidence_0.508_Length_1568
**C5Branched dibasic acid metabolism**	map00660	3	ec:2.2.1.6 synthase, ec:6.2.1.5 ligase (ADPforming), ec:1.1.1.85 dehydrogenase	1, 2, 1	Sample3_Locus_4596_Transcript_3/6_Confidence_0.400_Length_1157, Sample3_Locus_2152_Transcript_2/3_Confidence_0.333_Length_1481, SampleTS3_Locus_6340_Transcript_1/1_Confidence_0.000_Length_1077, SampleTS2_Locus_3967_Transcript_1/4_Confidence_0.667_Length_1445
**Chloroalkane and chloroalkene degradation**	map00625	3	ec:1.2.1.46 dehydrogenase, ec:1.1.1.1 dehydrogenase, ec:1.2.1.3 dehydrogenase (NAD+)	2, 5, 6	SampleTS2_Locus_4874_Transcript_6/12_Confidence_0.158_Length_1042, SampleTS3_Locus_23264_Transcript_1/3_Confidence_0.500_Length_845, SampleTS2_Locus_4874_Transcript_6/12_Confidence_0.158_Length_1042, Sample6_Locus_1524_Transcript_26/30_Confidence_0.185_Length_3215, SampleTS2_Locus_263_Transcript_1/3_Confidence_0.250_Length_2184, SampleTS3_Locus_400_Transcript_6/29_Confidence_0.422_Length_2479, SampleTS3_Locus_23264_Transcript_1/3_Confidence_0.500_Length_845, SampleTS3_Locus_23038_Transcript_2/3_Confidence_0.333_Length_1864, SampleTS2_Locus_4874_Transcript_6/12_Confidence_0.158_Length_1042, Sample3_Locus_23176_Transcript_2/3_Confidence_0.333_Length_1282, SampleTS1_Locus_5373_Transcript_26/33_Confidence_0.065_Length_1620, Sample6_Locus_12695_Transcript_1/2_Confidence_0.333_Length_2218, SampleTS3_Locus_23264_Transcript_1/3_Confidence_0.500_Length_845

**Geraniol degradation**	map00281	3	ec:2.3.1.16 Cacyltransferase, ec:4.2.1.17 hydratase, ec:1.1.1.35 dehydrogenase	1, 2, 3	SampleTS2_Locus_1750_Transcript_17/20_Confidence_0.182_Length_1568, SampleTS1_Locus_789_Transcript_9/11_Confidence_0.238_Length_2300, Sample3_Locus_5282_Transcript_2/7_Confidence_0.700_Length_1266, SampleTS1_Locus_789_Transcript_9/11_Confidence_0.238_Length_2300, SampleTS3_Locus_1827_Transcript_3/3_Confidence_0.000_Length_1033, SampleTS3_Locus_11808_Transcript_9/11_Confidence_0.565_Length_1006
**Glycosphingolipid biosynthesis globo and isoglobo series**	map00603	3	ec:2.4.99.4 alpha2,3sialyltransferase, ec:2.4.99.8 alpha2,8sialyltransferase, ec:3.2.1.22 melibiase	10, 3, 1	Sample6_Locus_1667_Transcript_21/40_Confidence_0.198_Length_1348, Sample6_Locus_6485_Transcript_12/18_Confidence_0.490_Length_1417, Sample6_Locus_22436_Transcript_2/2_Confidence_0.333_Length_1831, Sample3_Locus_191_Transcript_86/118_Confidence_0.018_Length_2298, Sample3_Locus_19889_Transcript_1/1_Confidence_0.000_Length_1128, SampleTS2_Locus_4156_Transcript_29/43_Confidence_0.097_Length_1215, SampleTS1_Locus_10921_Transcript_5/7_Confidence_0.444_Length_843, Sample6_Locus_28634_Transcript_1/2_Confidence_0.333_Length_832, SampleTS1_Locus_13929_Transcript_3/7_Confidence_0.091_Length_1486, SampleTS1_Locus_6551_Transcript_6/13_Confidence_0.111_Length_1643, Sample6_Locus_6485_Transcript_12/18_Confidence_0.490_Length_1417, Sample6_Locus_14905_Transcript_12/13_Confidence_0.077_Length_1757, Sample6_Locus_28634_Transcript_1/2_Confidence_0.333_Length_832, SampleTS2_Locus_3316_Transcript_5/7_Confidence_0.211_Length_3110

**Phenylpropanoid biosynthesis**	map00940	3	ec:1.2.1.68 dehydrogenase, ec:1.11.1.7 lactoperoxidase, ec:3.2.1.21 gentiobiase	1, 20, 2	SampleTS1_Locus_5373_Transcript_26/33_Confidence_0.065_Length_1620, SampleTS1_Locus_6669_Transcript_3/3_Confidence_0.200_Length_979, SampleTS2_Locus_760_Transcript_7/10_Confidence_0.186_Length_1031, Sample6_Locus_795_Transcript_19/29_Confidence_0.175_Length_1113, SampleTS2_Locus_4937_Transcript_4/7_Confidence_0.444_Length_827, Sample3_Locus_13267_Transcript_2/3_Confidence_0.667_Length_841, Sample1_Locus_18818_Transcript_1/1_Confidence_0.000_Length_983, SampleTS1_Locus_6640_Transcript_2/7_Confidence_0.357_Length_1361, Sample3_Locus_2283_Transcript_4/7_Confidence_0.091_Length_449, SampleTS2_Locus_7247_Transcript_5/9_Confidence_0.318_Length_1257, SampleTS2_Locus_11692_Transcript_2/7_Confidence_0.333_Length_2130, SampleTS3_Locus_2808_Transcript_2/4_Confidence_0.333_Length_770, Sample3_Locus_11502_Transcript_4/7_Confidence_0.636_Length_850, SampleTS2_Locus_20766_Transcript_5/7_Confidence_0.250_Length_1030, SampleTS1_Locus_1282_Transcript_9/25_Confidence_0.318_Length_1204, SampleTS3_Locus_10310_Transcript_1/2_Confidence_0.667_Length_957, SampleTS2_Locus_21396_Transcript_1/2_Confidence_0.667_Length_780, Sample3_Locus_2715_Transcript_4/6_Confidence_0.286_Length_835, SampleTS1_Locus_4034_Transcript_24/26_Confidence_0.200_Length_835, Sample6_Locus_13592_Transcript_5/5_Confidence_0.250_Length_879, Sample6_Locus_1113_Transcript_3/14_Confidence_0.211_Length_520, Sample1_Locus_25240_Transcript_4/4_Confidence_0.429_Length_2225, SampleTS2_Locus_9286_Transcript_1/1_Confidence_0.000_Length_2119

**Caprolactam degradation**	map00930	3	ec:1.1.1.2 dehydrogenase (NADP+), ec:4.2.1.17 hydratase, ec:1.1.1.35 dehydrogenase	10, 2, 3	Sample6_Locus_15547_Transcript_5/8_Confidence_0.545_Length_1332, Sample6_Locus_7369_Transcript_3/6_Confidence_0.200_Length_1828, SampleTS1_Locus_2734_Transcript_1/3_Confidence_0.333_Length_2491, SampleTS2_Locus_15764_Transcript_1/1_Confidence_0.000_Length_1068, Sample3_Locus_27117_Transcript_1/3_Confidence_0.250_Length_685, Sample6_Locus_6954_Transcript_4/5_Confidence_0.500_Length_1201, Sample6_Locus_4129_Transcript_8/27_Confidence_0.107_Length_571, SampleTS3_Locus_8149_Transcript_4/10_Confidence_0.118_Length_927, SampleTS1_Locus_6731_Transcript_14/15_Confidence_0.246_Length_2303, SampleTS1_Locus_10087_Transcript_6/7_Confidence_0.167_Length_948, SampleTS1_Locus_789_Transcript_9/11_Confidence_0.238_Length_2300, Sample3_Locus_5282_Transcript_2/7_Confidence_0.700_Length_1266, SampleTS1_Locus_789_Transcript_9/11_Confidence_0.238_Length_2300, SampleTS3_Locus_1827_Transcript_3/3_Confidence_0.000_Length_1033, SampleTS3_Locus_11808_Transcript_9/11_Confidence_0.565_Length_1006
**Arachidonic acid metabolism**	map00590	3	ec:1.1.1.184 reductase (NADPH), ec:1.11.1.9 peroxidase, ec:3.1.1.4 A2	1, 6, 4	Sample6_Locus_15547_Transcript_5/8_Confidence_0.545_Length_1332, SampleTS1_Locus_6640_Transcript_2/7_Confidence_0.357_Length_1361, SampleTS3_Locus_2808_Transcript_2/4_Confidence_0.333_Length_770, Sample6_Locus_795_Transcript_19/29_Confidence_0.175_Length_1113, Sample3_Locus_2715_Transcript_4/6_Confidence_0.286_Length_835, Sample1_Locus_18818_Transcript_1/1_Confidence_0.000_Length_983, Sample6_Locus_1113_Transcript_3/14_Confidence_0.211_Length_520, SampleTS1_Locus_587_Transcript_4/24_Confidence_0.067_Length_950, SampleTS2_Locus_23527_Transcript_1/1_Confidence_0.000_Length_1031, SampleTS1_Locus_11100_Transcript_2/5_Confidence_0.286_Length_1260, SampleTS3_Locus_8220_Transcript_1/4_Confidence_0.667_Length_1081

**Ether lipid metabolism**	map00565	3	ec:3.1.1.4 A2, ec:3.1.3.4 phosphatase, ec:2.8.2.11 sulfotransferase	4, 2, 1	SampleTS1_Locus_587_Transcript_4/24_Confidence_0.067_Length_950, SampleTS2_Locus_23527_Transcript_1/1_Confidence_0.000_Length_1031, SampleTS1_Locus_11100_Transcript_2/5_Confidence_0.286_Length_1260, SampleTS3_Locus_8220_Transcript_1/4_Confidence_0.667_Length_1081, Sample1_Locus_2193_Transcript_40/42_Confidence_0.262_Length_1445, SampleTS2_Locus_23786_Transcript_1/1_Confidence_0.000_Length_866, Sample1_Locus_565_Transcript_2/8_Confidence_0.636_Length_882
**Riboflavin metabolism**	map00740	3	ec:3.6.1.9 diphosphatase, ec:3.6.1.18 diphosphatase, ec:3.1.3.2 phosphatase	5, 1, 2	Sample6_Locus_1849_Transcript_4/6_Confidence_0.429_Length_866, Sample3_Locus_345_Transcript_6/8_Confidence_0.455_Length_1801, SampleTS3_Locus_5573_Transcript_4/5_Confidence_0.333_Length_940, Sample3_Locus_10699_Transcript_3/4_Confidence_0.400_Length_957, Sample3_Locus_27182_Transcript_2/3_Confidence_0.250_Length_1010, Sample3_Locus_10699_Transcript_3/4_Confidence_0.400_Length_957, Sample3_Locus_3056_Transcript_6/6_Confidence_0.000_Length_1859, SampleTS1_Locus_10554_Transcript_2/2_Confidence_0.000_Length_719

**Thiamine metabolism**	map00730	3	ec:2.7.4.3 kinase, ec:2.8.1.7 desulfurase, ec:3.6.1.15 phosphatase	7, 2, 278	Sample3_Locus_10333_Transcript_2/3_Confidence_0.750_Length_926, Sample3_Locus_3294_Transcript_4/6_Confidence_0.429_Length_2049, SampleTS1_Locus_6152_Transcript_2/7_Confidence_0.375_Length_1167, Sample3_Locus_9766_Transcript_1/1_Confidence_0.000_Length_1440, Sample3_Locus_6601_Transcript_5/5_Confidence_0.200_Length_4716, SampleTS1_Locus_1762_Transcript_13/36_Confidence_0.058_Length_819, Sample1_Locus_24078_Transcript_6/7_Confidence_0.150_Length_1572, Sample1_Locus_3213_Transcript_2/3_Confidence_0.667_Length_1138, SampleTS3_Locus_8776_Transcript_8/10_Confidence_0.567_Length_524, SampleTS3_Locus_13653_Transcript_1/3_Confidence_0.667_Length_1397, Sample6_Locus_2550_Transcript_5/11_Confidence_0.238_Length_1406, SampleTS2_Locus_8844_Transcript_2/4_Confidence_0.200_Length_1216, Sample6_Locus_4222_Transcript_1/1_Confidence_0.000_Length_1621, SampleTS1_Locus_1444_Transcript_2/3_Confidence_0.125_Length_1079, Sample6_Locus_21118_Transcript_1/3_Confidence_0.667_Length_480, Sample3_Locus_4496_Transcript_5/7_Confidence_0.200_Length_1045, SampleTS1_Locus_3295_Transcript_4/7_Confidence_0.500_Length_1706, SampleTS1_Locus_7342_Transcript_9/11_Confidence_0.250_Length_2056, Sample6_Locus_4781_Transcript_1/2_Confidence_0.333_Length_1290, Sample6_Locus_4460_Transcript_7/9_Confidence_0.417_Length_984, Sample1_Locus_25395_Transcript_1/4_Confidence_0.167_Length_1170, Sample6_Locus_4959_Transcript_8/8_Confidence_0.021_Length_1488, Sample6_Locus_381_Transcript_17/31_Confidence_0.109_Length_686, SampleTS1_Locus_3903_Transcript_4/10_Confidence_0.286_Length_1812, Sample6_Locus_9657_Transcript_7/9_Confidence_0.120_Length_2317, SampleTS2_Locus_2038_Transcript_10/15_Confidence_0.125_Length_523, SampleTS1_Locus_8634_Transcript_2/3_Confidence_0.333_Length_1251, Sample6_Locus_10491_Transcript_7/7_Confidence_0.125_Length_4037, SampleTS1_Locus_1405_Transcript_15/33_Confidence_0.062_Length_1213, SampleTS1_Locus_3177_Transcript_5/5_Confidence_0.000_Length_2422, Sample6_Locus_18055_Transcript_5/5_Confidence_0.111_Length_1330, Sample6_Locus_832_Transcript_2/3_Confidence_0.667_Length_879, SampleTS3_Locus_8946_Transcript_7/8_Confidence_0.091_Length_2134, Sample6_Locus_164_Transcript_81/296_Confidence_0.015_Length_2930, SampleTS3_Locus_3584_Transcript_11/17_Confidence_0.115_Length_3782, Sample6_Locus_25982_Transcript_2/4_Confidence_0.667_Length_1624, SampleTS3_Locus_12142_Transcript_3/6_Confidence_0.250_Length_2865, SampleTS2_Locus_1159_Transcript_15/16_Confidence_0.308_Length_665, SampleTS3_Locus_1866_Transcript_14/24_Confidence_0.200_Length_656, SampleTS2_Locus_5494_Transcript_10/17_Confidence_0.417_Length_3230, SampleTS1_Locus_5190_Transcript_5/7_Confidence_0.286_Length_4645, Sample3_Locus_6601_Transcript_5/5_Confidence_0.200_Length_4716, Sample6_Locus_13814_Transcript_7/9_Confidence_0.217_Length_1987, Sample6_Locus_19560_Transcript_2/3_Confidence_0.333_Length_2582, SampleTS3_Locus_19217_Transcript_2/5_Confidence_0.600_Length_2702, Sample6_Locus_18534_Transcript_3/3_Confidence_0.000_Length_1555, Sample6_Locus_4908_Transcript_6/6_Confidence_0.000_Length_1794, Sample6_Locus_10088_Transcript_1/2_Confidence_0.667_Length_1090, SampleTS2_Locus_2452_Transcript_2/8_Confidence_0.364_Length_455, Sample6_Locus_19645_Transcript_2/4_Confidence_0.333_Length_1748, SampleTS2_Locus_21455_Transcript_2/3_Confidence_0.000_Length_1624, Sample3_Locus_1244_Transcript_63/90_Confidence_0.004_Length_1149, Sample6_Locus_33304_Transcript_3/4_Confidence_0.200_Length_839, Sample3_Locus_13999_Transcript_2/3_Confidence_0.333_Length_1333, Sample6_Locus_4130_Transcript_2/6_Confidence_0.375_Length_1684, SampleTS1_Locus_24562_Transcript_5/6_Confidence_0.400_Length_1717, Sample6_Locus_11465_Transcript_4/6_Confidence_0.333_Length_1334, Sample6_Locus_7465_Transcript_1/1_Confidence_0.000_Length_1622, SampleTS2_Locus_4758_Transcript_3/5_Confidence_0.500_Length_584, Sample1_Locus_18570_Transcript_2/2_Confidence_0.000_Length_1230, Sample3_Locus_14815_Transcript_9/11_Confidence_0.071_Length_984, Sample3_Locus_31540_Transcript_1/1_Confidence_0.926_Length_1324, Sample6_Locus_15929_Transcript_2/4_Confidence_0.429_Length_719, Sample6_Locus_5873_Transcript_1/5_Confidence_0.333_Length_908, SampleTS1_Locus_28650_Transcript_1/4_Confidence_0.250_Length_1505

					, SampleTS3_Locus_1540_Transcript_16/37_Confidence_0.097_Length_571, Sample1_Locus_25642_Transcript_6/7_Confidence_0.500_Length_1752, Sample3_Locus_9363_Transcript_11/12_Confidence_0.300_Length_752, SampleTS3_Locus_5309_Transcript_11/20_Confidence_0.050_Length_1454, Sample3_Locus_3218_Transcript_7/8_Confidence_0.375_Length_2978, Sample6_Locus_25706_Transcript_1/1_Confidence_0.000_Length_2115, Sample3_Locus_10951_Transcript_3/4_Confidence_0.333_Length_1105, Sample3_Locus_11069_Transcript_2/8_Confidence_0.182_Length_1280, Sample3_Locus_5454_Transcript_1/7_Confidence_0.273_Length_1345, SampleTS1_Locus_3342_Transcript_18/19_Confidence_0.040_Length_3749, Sample3_Locus_20016_Transcript_6/9_Confidence_0.273_Length_2073, Sample6_Locus_29189_Transcript_1/1_Confidence_0.000_Length_540, SampleTS3_Locus_23452_Transcript_2/2_Confidence_0.333_Length_474, Sample6_Locus_26588_Transcript_2/2_Confidence_0.000_Length_489, SampleTS1_Locus_3223_Transcript_3/36_Confidence_0.029_Length_1583, SampleTS1_Locus_217_Transcript_13/13_Confidence_0.355_Length_910, SampleTS2_Locus_530_Transcript_232/383_Confidence_0.006_Length_1315, SampleTS3_Locus_3667_Transcript_4/12_Confidence_0.364_Length_2296, Sample6_Locus_3232_Transcript_3/5_Confidence_0.545_Length_999, SampleTS1_Locus_8934_Transcript_5/6_Confidence_0.062_Length_3128, Sample3_Locus_15902_Transcript_7/11_Confidence_0.412_Length_3566, Sample3_Locus_23626_Transcript_1/1_Confidence_0.000_Length_981, Sample3_Locus_3989_Transcript_4/4_Confidence_0.000_Length_1366, SampleTS1_Locus_4491_Transcript_2/5_Confidence_0.571_Length_1595, SampleTS1_Locus_3079_Transcript_1/6_Confidence_0.333_Length_2652, Sample3_Locus_4942_Transcript_1/2_Confidence_0.333_Length_2760, Sample3_Locus_1247_Transcript_10/17_Confidence_0.625_Length_2553, Sample3_Locus_25985_Transcript_1/1_Confidence_0.000_Length_1430, SampleTS2_Locus_154_Transcript_3/9_Confidence_0.083_Length_762, SampleTS1_Locus_1128_Transcript_3/3_Confidence_0.000_Length_491, SampleTS3_Locus_4130_Transcript_1/5_Confidence_0.167_Length_1505, SampleTS2_Locus_20730_Transcript_3/3_Confidence_0.000_Length_1446, SampleTS3_Locus_18221_Transcript_1/2_Confidence_0.333_Length_931, SampleTS1_Locus_18532_Transcript_2/4_Confidence_0.333_Length_1303, Sample6_Locus_12309_Transcript_1/1_Confidence_0.000_Length_1290, Sample6_Locus_16160_Transcript_1/2_Confidence_0.333_Length_1514, Sample3_Locus_1698_Transcript_7/9_Confidence_0.111_Length_1910, Sample6_Locus_25656_Transcript_2/3_Confidence_0.500_Length_785, Sample6_Locus_4942_Transcript_5/6_Confidence_0.500_Length_1741, SampleTS1_Locus_9060_Transcript_10/13_Confidence_0.611_Length_2746, SampleTS1_Locus_4296_Transcript_6/8_Confidence_0.333_Length_1626, Sample6_Locus_2597_Transcript_1/1_Confidence_0.000_Length_1284, SampleTS3_Locus_213_Transcript_1/10_Confidence_0.188_Length_1035, Sample3_Locus_5066_Transcript_18/21_Confidence_0.464_Length_2997, Sample6_Locus_8035_Transcript_8/15_Confidence_0.095_Length_4770, Sample6_Locus_12469_Transcript_1/3_Confidence_0.400_Length_1564, Sample6_Locus_9946_Transcript_4/6_Confidence_0.600_Length_2469, Sample6_Locus_4638_Transcript_4/6_Confidence_0.500_Length_2485, Sample3_Locus_4140_Transcript_1/3_Confidence_0.333_Length_1526, SampleTS1_Locus_11450_Transcript_8/18_Confidence_0.222_Length_2439, Sample6_Locus_9985_Transcript_2/5_Confidence_0.143_Length_1859, Sample3_Locus_1352_Transcript_3/6_Confidence_0.385_Length_690, Sample1_Locus_11542_Transcript_4/6_Confidence_0.200_Length_1313, Sample6_Locus_10676_Transcript_1/1_Confidence_0.000_Length_1850, Sample6_Locus_830_Transcript_5/5_Confidence_0.000_Length_1043, SampleTS1_Locus_10413_Transcript_7/8_Confidence_0.500_Length_2083, Sample3_Locus_6079_Transcript_6/7_Confidence_0.286_Length_3325, Sample3_Locus_1341_Transcript_4/6_Confidence_0.500_Length_950, Sample3_Locus_10333_Transcript_2/3_Confidence_0.750_Length_926, SampleTS3_Locus_7380_Transcript_1/1_Confidence_0.000_Length_2585, Sample6_Locus_4473_Transcript_5/6_Confidence_0.583_Length_591, SampleTS3_Locus_8920_Transcript_1/2_Confidence_0.333_Length_1841, Sample6_Locus_1407_Transcript_6/6_Confidence_0.200_Length_957, SampleTS1_Locus_23722_Transcript_4/4_Confidence_0.000_Length_1687, Sample3_Locus_11117_Transcript_5/5_Confidence_0.250_Length_3512, Sample1_Locus_18264_Transcript_1/3_Confidence_0.333_Length_1805, Sample6_Locus_1443_Transcript_1/3_Confidence_0.750_Length_1752,

					SampleTS1_Locus_12078_Transcript_1/1_Confidence_0.000_Length_736, Sample3_Locus_5270_Transcript_3/3_Confidence_0.000_Length_2249, SampleTS2_Locus_5041_Transcript_4/5_Confidence_0.500_Length_756, SampleTS1_Locus_8088_Transcript_1/1_Confidence_0.000_Length_2675, SampleTS1_Locus_13616_Transcript_4/6_Confidence_0.154_Length_858, SampleTS2_Locus_15504_Transcript_2/4_Confidence_0.333_Length_1132, SampleTS2_Locus_22391_Transcript_2/5_Confidence_0.700_Length_1831, SampleTS3_Locus_9956_Transcript_3/3_Confidence_0.250_Length_794, SampleTS3_Locus_5309_Transcript_8/20_Confidence_0.075_Length_1791, Sample6_Locus_25614_Transcript_1/2_Confidence_1.000_Length_1993, Sample6_Locus_18158_Transcript_1/1_Confidence_0.000_Length_973, Sample3_Locus_2946_Transcript_11/14_Confidence_0.115_Length_1840, SampleTS1_Locus_9204_Transcript_11/12_Confidence_0.077_Length_2853, SampleTS2_Locus_21085_Transcript_4/5_Confidence_0.571_Length_1602, Sample6_Locus_9200_Transcript_1/1_Confidence_0.000_Length_2654, SampleTS2_Locus_2441_Transcript_3/5_Confidence_0.500_Length_1920, SampleTS3_Locus_25647_Transcript_1/1_Confidence_0.000_Length_360, Sample3_Locus_4871_Transcript_6/8_Confidence_0.440_Length_1214, Sample6_Locus_3178_Transcript_6/6_Confidence_0.222_Length_1425, Sample3_Locus_1409_Transcript_6/7_Confidence_0.286_Length_3090, Sample6_Locus_6095_Transcript_5/10_Confidence_0.250_Length_1483, Sample3_Locus_1573_Transcript_4/5_Confidence_0.286_Length_2133, Sample3_Locus_1963_Transcript_4/4_Confidence_0.000_Length_950, Sample3_Locus_3435_Transcript_1/2_Confidence_0.333_Length_1240, Sample3_Locus_4668_Transcript_5/5_Confidence_0.000_Length_1437, Sample1_Locus_18669_Transcript_5/17_Confidence_0.008_Length_1835, Sample6_Locus_6095_Transcript_7/10_Confidence_0.000_Length_1419, Sample6_Locus_4583_Transcript_1/1_Confidence_0.000_Length_759, Sample1_Locus_23436_Transcript_1/2_Confidence_0.333_Length_673, SampleTS1_Locus_27154_Transcript_1/1_Confidence_0.000_Length_1250, SampleTS1_Locus_1021_Transcript_1/8_Confidence_0.100_Length_1554, SampleTS3_Locus_6559_Transcript_1/5_Confidence_0.375_Length_3431, Sample3_Locus_6505_Transcript_4/8_Confidence_0.545_Length_2485, Sample1_Locus_23910_Transcript_3/3_Confidence_0.000_Length_1374, Sample3_Locus_5615_Transcript_4/4_Confidence_0.000_Length_2332, Sample6_Locus_3633_Transcript_29/29_Confidence_0.014_Length_1436, Sample3_Locus_542_Transcript_7/8_Confidence_0.333_Length_2614, Sample6_Locus_12915_Transcript_1/2_Confidence_0.333_Length_449, Sample3_Locus_450_Transcript_9/16_Confidence_0.476_Length_3424, Sample3_Locus_23759_Transcript_1/2_Confidence_0.333_Length_870, SampleTS1_Locus_2824_Transcript_1/2_Confidence_0.667_Length_2271, Sample6_Locus_6064_Transcript_22/31_Confidence_0.237_Length_4671, Sample3_Locus_7171_Transcript_1/2_Confidence_0.333_Length_591, Sample6_Locus_655_Transcript_11/16_Confidence_0.278_Length_1559, Sample6_Locus_1856_Transcript_4/5_Confidence_0.200_Length_2451, Sample6_Locus_6979_Transcript_7/9_Confidence_0.133_Length_1066, Sample3_Locus_182_Transcript_7/15_Confidence_0.357_Length_1190, Sample6_Locus_23006_Transcript_3/4_Confidence_0.333_Length_1913, Sample3_Locus_733_Transcript_9/13_Confidence_0.176_Length_669, SampleTS2_Locus_150_Transcript_3/9_Confidence_0.071_Length_2961, Sample6_Locus_22197_Transcript_1/1_Confidence_0.000_Length_722, SampleTS2_Locus_3665_Transcript_1/1_Confidence_0.000_Length_1325, Sample6_Locus_4336_Transcript_6/7_Confidence_0.100_Length_1059, Sample3_Locus_5314_Transcript_7/11_Confidence_0.300_Length_2313, Sample3_Locus_6357_Transcript_4/4_Confidence_0.000_Length_1942, SampleTS1_Locus_7257_Transcript_1/1_Confidence_0.000_Length_2715, SampleTS3_Locus_3402_Transcript_1/2_Confidence_0.333_Length_782, Sample1_Locus_20036_Transcript_3/5_Confidence_0.286_Length_983, Sample3_Locus_11046_Transcript_10/13_Confidence_0.231_Length_2112, SampleTS2_Locus_24342_Transcript_1/1_Confidence_0.000_Length_766, Sample3_Locus_3455_Transcript_2/4_Confidence_0.444_Length_3322, Sample6_Locus_717_Transcript_19/21_Confidence_0.138_Length_5498, Sample6_Locus_1912_Transcript_4/4_Confidence_0.200_Length_866, SampleTS1_Locus_10744_Transcript_1/2_Confidence_0.750_Length_662, Sample6_Locus_5970_Transcript_2/3_Confidence_0.333_Length_1694, SampleTS1_Locus_411_Transcript_4/5_Confidence_0.400_Length_1732, SampleTS1_Locus_3753_Transcript_6/9_Confidence_0.286_Length_4045, SampleTS1_Locus_13697_Transcript_1/1_Confidence_0.000_Length_807, Sample6_Locus_6776_Transcript_2/2_Confidence_0.000_Length_1183,

					SampleTS2_Locus_2611_Transcript_4/5_Confidence_0.143_Length_1901, Sample3_Locus_1962_Transcript_1/3_Confidence_0.333_Length_2237, SampleTS1_Locus_6958_Transcript_5/8_Confidence_0.429_Length_1672, Sample1_Locus_22269_Transcript_4/4_Confidence_0.333_Length_3009, Sample3_Locus_13026_Transcript_4/6_Confidence_0.320_Length_2029, SampleTS2_Locus_923_Transcript_3/4_Confidence_0.400_Length_925, Sample3_Locus_22917_Transcript_6/12_Confidence_0.267_Length_1675, SampleTS3_Locus_19797_Transcript_3/3_Confidence_0.000_Length_1329, SampleTS3_Locus_1460_Transcript_4/5_Confidence_0.600_Length_1005, Sample6_Locus_6166_Transcript_4/5_Confidence_0.400_Length_3519, SampleTS2_Locus_3082_Transcript_1/4_Confidence_0.429_Length_1115, SampleTS1_Locus_102_Transcript_7/24_Confidence_0.073_Length_1592, SampleTS3_Locus_8121_Transcript_3/5_Confidence_0.600_Length_1476, Sample3_Locus_10180_Transcript_4/6_Confidence_0.500_Length_2179, Sample3_Locus_6982_Transcript_5/15_Confidence_0.079_Length_800, Sample6_Locus_4492_Transcript_6/8_Confidence_0.429_Length_1081, SampleTS2_Locus_9225_Transcript_2/2_Confidence_0.000_Length_1735, Sample6_Locus_9272_Transcript_1/1_Confidence_0.000_Length_455, Sample3_Locus_29450_Transcript_1/1_Confidence_0.000_Length_934, Sample3_Locus_315_Transcript_16/20_Confidence_0.074_Length_3675, Sample6_Locus_5614_Transcript_1/1_Confidence_0.000_Length_834, Sample1_Locus_24238_Transcript_3/4_Confidence_0.400_Length_1703, Sample3_Locus_4755_Transcript_1/2_Confidence_0.667_Length_1045, Sample6_Locus_23152_Transcript_1/1_Confidence_0.000_Length_978, Sample3_Locus_8213_Transcript_2/3_Confidence_0.333_Length_1530, Sample3_Locus_7479_Transcript_6/7_Confidence_0.019_Length_895, SampleTS3_Locus_10854_Transcript_2/5_Confidence_0.667_Length_1018, Sample6_Locus_15076_Transcript_3/5_Confidence_0.375_Length_1959, Sample6_Locus_3087_Transcript_12/15_Confidence_0.200_Length_3172, Sample3_Locus_18821_Transcript_3/4_Confidence_0.333_Length_1725, SampleTS3_Locus_11257_Transcript_2/3_Confidence_0.333_Length_2353, SampleTS1_Locus_3593_Transcript_3/9_Confidence_0.333_Length_4391, SampleTS1_Locus_8184_Transcript_3/5_Confidence_0.400_Length_3710, Sample6_Locus_30028_Transcript_1/2_Confidence_0.333_Length_1025, SampleTS3_Locus_1032_Transcript_5/6_Confidence_0.286_Length_1670, Sample3_Locus_1637_Transcript_14/16_Confidence_0.317_Length_2963, SampleTS1_Locus_3499_Transcript_2/4_Confidence_0.333_Length_3331, SampleTS1_Locus_27733_Transcript_1/1_Confidence_0.000_Length_647, Sample6_Locus_5499_Transcript_1/1_Confidence_0.000_Length_545, Sample3_Locus_8292_Transcript_18/19_Confidence_0.222_Length_11337, Sample3_Locus_20305_Transcript_4/9_Confidence_0.417_Length_708, SampleTS2_Locus_7833_Transcript_5/5_Confidence_0.429_Length_1991, Sample3_Locus_975_Transcript_6/34_Confidence_0.159_Length_709, Sample6_Locus_16096_Transcript_4/4_Confidence_0.286_Length_1064, Sample3_Locus_7036_Transcript_25/26_Confidence_0.147_Length_13961, SampleTS1_Locus_24533_Transcript_3/3_Confidence_0.000_Length_1163, Sample3_Locus_1985_Transcript_7/8_Confidence_0.062_Length_1287, Sample6_Locus_18554_Transcript_1/4_Confidence_0.222_Length_1011, SampleTS3_Locus_3563_Transcript_3/8_Confidence_0.500_Length_2008, Sample6_Locus_10878_Transcript_6/8_Confidence_0.273_Length_1218, Sample6_Locus_2442_Transcript_4/16_Confidence_0.038_Length_1846, Sample1_Locus_18633_Transcript_5/7_Confidence_0.333_Length_1633, SampleTS3_Locus_1476_Transcript_12/16_Confidence_0.250_Length_1542, SampleTS1_Locus_28320_Transcript_1/1_Confidence_0.000_Length_695, Sample6_Locus_8847_Transcript_4/5_Confidence_0.200_Length_4092, SampleTS2_Locus_13751_Transcript_1/2_Confidence_0.333_Length_661, SampleTS2_Locus_6386_Transcript_1/2_Confidence_0.667_Length_1527, Sample3_Locus_4090_Transcript_10/14_Confidence_0.353_Length_4125, SampleTS3_Locus_2656_Transcript_1/1_Confidence_0.000_Length_1732, Sample6_Locus_26409_Transcript_1/1_Confidence_0.000_Length_1698, SampleTS1_Locus_1925_Transcript_1/17_Confidence_0.089_Length_540, SampleTS2_Locus_5418_Transcript_1/1_Confidence_0.000_Length_2770, Sample6_Locus_14840_Transcript_1/3_Confidence_0.333_Length_1085, SampleTS1_Locus_22896_Transcript_1/1_Confidence_0.000_Length_1681, SampleTS3_Locus_10197_Transcript_4/10_Confidence_0.056_Length_1030, Sample6_Locus_3002_Transcript_11/25_Confidence_0.257_Length_1088, Sample3_Locus_9057_Transcript_4/5_Confidence_0.200_Length_803,

					Sample6_Locus_6606_Transcript_8/20_Confidence_0.082_Length_1322, SampleTS2_Locus_8111_Transcript_10/12_Confidence_0.231_Length_2831, SampleTS3_Locus_22958_Transcript_1/1_Confidence_0.000_Length_1054, Sample1_Locus_26944_Transcript_1/1_Confidence_0.000_Length_1423, Sample6_Locus_10169_Transcript_6/7_Confidence_0.607_Length_1376, Sample6_Locus_15179_Transcript_8/9_Confidence_0.429_Length_2067, Sample6_Locus_7700_Transcript_3/5_Confidence_0.133_Length_3205, SampleTS2_Locus_16641_Transcript_4/4_Confidence_0.222_Length_618, SampleTS2_Locus_10767_Transcript_2/2_Confidence_0.000_Length_757, Sample1_Locus_18923_Transcript_1/1_Confidence_0.000_Length_2951, Sample3_Locus_4819_Transcript_7/10_Confidence_0.250_Length_2758, Sample1_Locus_28199_Transcript_1/2_Confidence_0.750_Length_954, Sample3_Locus_4289_Transcript_3/4_Confidence_0.333_Length_3187, SampleTS3_Locus_4352_Transcript_2/3_Confidence_0.333_Length_1615, SampleTS3_Locus_18815_Transcript_3/4_Confidence_0.667_Length_1114, Sample6_Locus_28156_Transcript_2/3_Confidence_0.750_Length_396, Sample6_Locus_14369_Transcript_1/1_Confidence_0.000_Length_645, Sample6_Locus_10316_Transcript_3/8_Confidence_0.156_Length_721, SampleTS1_Locus_3702_Transcript_16/16_Confidence_0.120_Length_803
**Lipopolysaccharide biosynthesis**	map00540	2	ec:2.5.1.55 synthase, ec:5.3.1.13 isomerase	1, 4	SampleTS2_Locus_561_Transcript_5/5_Confidence_0.381_Length_1130, SampleTS3_Locus_1536_Transcript_4/6_Confidence_0.200_Length_1265, Sample6_Locus_2624_Transcript_5/10_Confidence_0.059_Length_1316, SampleTS3_Locus_10968_Transcript_8/14_Confidence_0.097_Length_1180, Sample3_Locus_3559_Transcript_2/6_Confidence_0.200_Length_1280

**mTOR signaling pathway**	map04150	2	ec:2.7.11.11 protein kinase, ec:2.7.11.24 protein kinase	3, 6	SampleTS1_Locus_170_Transcript_6/14_Confidence_0.556_Length_3699, Sample6_Locus_5763_Transcript_4/9_Confidence_0.625_Length_2241, SampleTS1_Locus_7915_Transcript_5/6_Confidence_0.074_Length_2827, Sample3_Locus_9819_Transcript_4/6_Confidence_0.300_Length_1186, Sample1_Locus_5473_Transcript_2/5_Confidence_0.400_Length_2142, Sample3_Locus_737_Transcript_6/9_Confidence_0.308_Length_1795, Sample1_Locus_1971_Transcript_3/6_Confidence_0.571_Length_1795, SampleTS1_Locus_5021_Transcript_2/3_Confidence_0.333_Length_1762, Sample6_Locus_21874_Transcript_4/6_Confidence_0.500_Length_1364
**Other types of Oglycan biosynthesis**	map00514	2	ec:2.4.1.109 mannosyltransferase, ec:2.4.99.1 alpha(2,6)sialyltransferase	1, 14	Sample6_Locus_2970_Transcript_1/3_Confidence_0.600_Length_1024, Sample6_Locus_19814_Transcript_10/10_Confidence_0.483_Length_1044, Sample3_Locus_19889_Transcript_1/1_Confidence_0.000_Length_1128, SampleTS2_Locus_4156_Transcript_29/43_Confidence_0.097_Length_1215, SampleTS2_Locus_5330_Transcript_4/5_Confidence_0.429_Length_1628, SampleTS1_Locus_13929_Transcript_3/7_Confidence_0.091_Length_1486, Sample6_Locus_5906_Transcript_9/11_Confidence_0.333_Length_2015, Sample6_Locus_6485_Transcript_12/18_Confidence_0.490_Length_1417, Sample1_Locus_19212_Transcript_2/4_Confidence_0.333_Length_2192, Sample3_Locus_191_Transcript_86/118_Confidence_0.018_Length_2298, Sample3_Locus_12595_Transcript_4/8_Confidence_0.364_Length_2480, SampleTS1_Locus_993_Transcript_3/9_Confidence_0.083_Length_1910, Sample3_Locus_25591_Transcript_4/4_Confidence_0.000_Length_1038, SampleTS2_Locus_11455_Transcript_4/10_Confidence_0.630_Length_1058, SampleTS1_Locus_21367_Transcript_2/3_Confidence_0.429_Length_1441
**Phosphonate and phosphinate metabolism**	map00440	2	ec:2.7.7.14 cytidylyltransferase, ec:2.7.7.15 cytidylyltransferase	1, 1	SampleTS3_Locus_256_Transcript_7/9_Confidence_0.444_Length_1142, SampleTS3_Locus_256_Transcript_7/9_Confidence_0.444_Length_1142
**Taurine and hypotaurine metabolism**	map00430	2	ec:4.1.1.15 decarboxylase, ec:2.3.2.2 glutamyl transpeptidase	1, 1	SampleTS3_Locus_5084_Transcript_15/17_Confidence_0.206_Length_1751, Sample1_Locus_19449_Transcript_1/1_Confidence_0.000_Length_1704

**Polyketide sugar unit biosynthesis**	map00523	2	ec:1.1.1.133 reductase, ec:5.1.3.13 3,5epimerase	2, 1	Sample3_Locus_2806_Transcript_6/14_Confidence_0.167_Length_1197, SampleTS2_Locus_1881_Transcript_15/19_Confidence_0.439_Length_912, Sample3_Locus_2806_Transcript_6/14_Confidence_0.167_Length_1197
**Linoleic acid metabolism**	map00591	2	ec:3.1.1.4 A2, ec:1.14.19.3 6desaturase	4, 1	SampleTS1_Locus_587_Transcript_4/24_Confidence_0.067_Length_950, SampleTS2_Locus_23527_Transcript_1/1_Confidence_0.000_Length_1031, SampleTS1_Locus_11100_Transcript_2/5_Confidence_0.286_Length_1260, SampleTS3_Locus_8220_Transcript_1/4_Confidence_0.667_Length_1081, SampleTS2_Locus_20864_Transcript_2/3_Confidence_0.667_Length_860
**Sesquiterpenoid and triterpenoid biosynthesis**	map00909	2	ec:1.1.1.216 dehydrogenase (NADP+), ec:2.5.1.21 synthase	1, 1	SampleTS1_Locus_13725_Transcript_2/7_Confidence_0.333_Length_1080, Sample6_Locus_901_Transcript_6/8_Confidence_0.643_Length_1628
**Vitamin B6 metabolism**	map00750	2	ec:2.6.1.52 transaminase, ec:4.2.3.1 synthase	1, 2	Sample1_Locus_9264_Transcript_1/1_Confidence_0.000_Length_1678, Sample6_Locus_4215_Transcript_12/29_Confidence_0.125_Length_1509, Sample6_Locus_4215_Transcript_14/29_Confidence_0.069_Length_739
**Mucin type Oglycan biosynthesis**	map00512	2	ec:2.4.99.4 alpha2,3sialyltransferase, ec:2.4.99.3 alpha2,6sialyltransferase	10, 1	Sample6_Locus_1667_Transcript_21/40_Confidence_0.198_Length_1348, Sample6_Locus_6485_Transcript_12/18_Confidence_0.490_Length_1417, Sample6_Locus_22436_Transcript_2/2_Confidence_0.333_Length_1831, Sample3_Locus_191_Transcript_86/118_Confidence_0.018_Length_2298, Sample3_Locus_19889_Transcript_1/1_Confidence_0.000_Length_1128, SampleTS2_Locus_4156_Transcript_29/43_Confidence_0.097_Length_1215, SampleTS1_Locus_10921_Transcript_5/7_Confidence_0.444_Length_843, Sample6_Locus_28634_Transcript_1/2_Confidence_0.333_Length_832, SampleTS1_Locus_13929_Transcript_3/7_Confidence_0.091_Length_1486, SampleTS1_Locus_6551_Transcript_6/13_Confidence_0.111_Length_1643, Sample6_Locus_28634_Transcript_1/2_Confidence_0.333_Length_832
**Other glycan degradation**	map00511	2	ec:3.2.1.24 alphaDmannosidase, ec:3.2.1.23 lactase (ambiguous)	1, 1	Sample6_Locus_6857_Transcript_8/11_Confidence_0.273_Length_3549, SampleTS2_Locus_9286_Transcript_1/1_Confidence_0.000_Length_2119

**Limonene and pinene degradation**	map00903	2	ec:4.2.1.17 hydratase, ec:1.2.1.3 dehydrogenase (NAD+)	2, 6	SampleTS1_Locus_789_Transcript_9/11_Confidence_0.238_Length_2300, Sample3_Locus_5282_Transcript_2/7_Confidence_0.700_Length_1266, SampleTS3_Locus_23038_Transcript_2/3_Confidence_0.333_Length_1864, SampleTS2_Locus_4874_Transcript_6/12_Confidence_0.158_Length_1042, Sample3_Locus_23176_Transcript_2/3_Confidence_0.333_Length_1282, SampleTS1_Locus_5373_Transcript_26/33_Confidence_0.065_Length_1620, Sample6_Locus_12695_Transcript_1/2_Confidence_0.333_Length_2218, SampleTS3_Locus_23264_Transcript_1/3_Confidence_0.500_Length_845
**Insect hormone biosynthesis**	map00981	2	ec:1.1.1.216 dehydrogenase (NADP+), ec:1.2.1.3 dehydrogenase (NAD+)	1, 6	SampleTS1_Locus_13725_Transcript_2/7_Confidence_0.333_Length_1080, SampleTS3_Locus_23038_Transcript_2/3_Confidence_0.333_Length_1864, SampleTS2_Locus_4874_Transcript_6/12_Confidence_0.158_Length_1042, Sample3_Locus_23176_Transcript_2/3_Confidence_0.333_Length_1282, SampleTS1_Locus_5373_Transcript_26/33_Confidence_0.065_Length_1620, Sample6_Locus_12695_Transcript_1/2_Confidence_0.333_Length_2218, SampleTS3_Locus_23264_Transcript_1/3_Confidence_0.500_Length_845
**Neomycin, kanamycin and gentamicin biosynthesis**	map00524	2	ec:2.7.1.2 glucokinase (phosphorylating), ec:2.7.1.1 hexokinase type IV glucokinase	1, 1	Sample6_Locus_5095_Transcript_5/8_Confidence_0.300_Length_1241, Sample6_Locus_5095_Transcript_5/8_Confidence_0.300_Length_1241
**Steroid degradation**	map00984	2	ec:1.3.99.5 4dehydrogenase (acceptor), ec:1.1.1.51 17)betahydroxy steroid dehydrogenase	2, 1	SampleTS2_Locus_1258_Transcript_11/11_Confidence_0.182_Length_1002, SampleTS3_Locus_4932_Transcript_2/7_Confidence_0.100_Length_697, SampleTS3_Locus_11808_Transcript_9/11_Confidence_0.565_Length_1006
**Atrazine degradation**	map00791	2	ec:3.5.1.84 amidohydrolase, ec:3.5.1.5 ec:3.5.1.5 urease	1, 1	Sample6_Locus_14763_Transcript_11/12_Confidence_0.182_Length_1117, SampleTS3_Locus_10659_Transcript_7/8_Confidence_0.389_Length_1749

**T cell receptor signaling pathway**	map04660	2	ec:3.1.3.16 phosphatase, ec:2.7.10.2 proteintyrosine kinase	49, 2	SampleTS3_Locus_1236_Transcript_8/14_Confidence_0.379_Length_696, SampleTS1_Locus_5854_Transcript_5/5_Confidence_0.400_Length_861, Sample3_Locus_4609_Transcript_5/8_Confidence_0.444_Length_1430, SampleTS3_Locus_1630_Transcript_4/8_Confidence_0.500_Length_1635, SampleTS2_Locus_4720_Transcript_3/7_Confidence_0.667_Length_1404, Sample3_Locus_5932_Transcript_3/4_Confidence_0.571_Length_501, SampleTS3_Locus_13512_Transcript_1/3_Confidence_0.333_Length_1321, SampleTS2_Locus_1774_Transcript_4/5_Confidence_0.500_Length_990, SampleTS2_Locus_549_Transcript_10/36_Confidence_0.207_Length_1084, Sample3_Locus_14090_Transcript_2/4_Confidence_0.714_Length_1345, Sample6_Locus_5727_Transcript_3/3_Confidence_0.000_Length_2301, SampleTS3_Locus_5284_Transcript_10/12_Confidence_0.450_Length_1002, Sample3_Locus_3056_Transcript_6/6_Confidence_0.000_Length_1859, Sample3_Locus_13102_Transcript_1/2_Confidence_0.333_Length_1552, Sample1_Locus_1599_Transcript_1/1_Confidence_0.000_Length_563, SampleTS3_Locus_9298_Transcript_1/10_Confidence_0.238_Length_793, SampleTS1_Locus_2823_Transcript_2/10_Confidence_0.444_Length_2044, Sample6_Locus_4221_Transcript_5/15_Confidence_0.231_Length_1620, Sample1_Locus_9346_Transcript_2/6_Confidence_0.500_Length_949, Sample3_Locus_23182_Transcript_4/4_Confidence_0.000_Length_3946, SampleTS2_Locus_4899_Transcript_2/5_Confidence_0.357_Length_658, Sample6_Locus_594_Transcript_17/21_Confidence_0.471_Length_4353, SampleTS1_Locus_4566_Transcript_4/4_Confidence_0.000_Length_2764, SampleTS2_Locus_2599_Transcript_3/9_Confidence_0.091_Length_4008, SampleTS1_Locus_397_Transcript_66/78_Confidence_0.022_Length_711, Sample1_Locus_9441_Transcript_1/3_Confidence_0.333_Length_1512, SampleTS1_Locus_26651_Transcript_1/1_Confidence_0.000_Length_1107, SampleTS2_Locus_549_Transcript_23/36_Confidence_0.110_Length_766, Sample3_Locus_5257_Transcript_3/8_Confidence_0.333_Length_2904, Sample6_Locus_11565_Transcript_7/13_Confidence_0.097_Length_1502, Sample1_Locus_27168_Transcript_1/2_Confidence_1.000_Length_653, Sample6_Locus_24750_Transcript_4/9_Confidence_0.222_Length_785, SampleTS2_Locus_2994_Transcript_7/9_Confidence_0.235_Length_2922, Sample3_Locus_2926_Transcript_2/3_Confidence_0.750_Length_1774, SampleTS3_Locus_23494_Transcript_1/1_Confidence_0.000_Length_1156, Sample3_Locus_23102_Transcript_1/2_Confidence_0.667_Length_1067, Sample6_Locus_8346_Transcript_7/13_Confidence_0.118_Length_1292, SampleTS1_Locus_12977_Transcript_4/6_Confidence_0.429_Length_1160, SampleTS1_Locus_6027_Transcript_7/14_Confidence_0.160_Length_504, Sample3_Locus_22654_Transcript_1/1_Confidence_0.000_Length_1356, Sample6_Locus_1159_Transcript_30/32_Confidence_0.027_Length_1920, SampleTS3_Locus_2277_Transcript_3/5_Confidence_0.400_Length_1847, SampleTS1_Locus_30500_Transcript_2/2_Confidence_0.850_Length_548, Sample6_Locus_30120_Transcript_2/3_Confidence_0.667_Length_1321, SampleTS2_Locus_3992_Transcript_15/16_Confidence_0.143_Length_1808, SampleTS1_Locus_11573_Transcript_5/10_Confidence_0.211_Length_1117, Sample1_Locus_25057_Transcript_2/6_Confidence_0.727_Length_1459, SampleTS2_Locus_549_Transcript_22/36_Confidence_0.134_Length_768, Sample1_Locus_13613_Transcript_6/7_Confidence_0.111_Length_1026, SampleTS2_Locus_1249_Transcript_11/12_Confidence_0.300_Length_1726, Sample6_Locus_17366_Transcript_1/1_Confidence_0.000_Length_2652
**Steroid hormone biosynthesis**	map00140	2	ec:1.1.1.51 17)betahydroxy steroid dehydrogenase, ec:2.4.1.17 1naphthol glucuronyltransferase	1, 3	SampleTS3_Locus_11808_Transcript_9/11_Confidence_0.565_Length_1006, Sample6_Locus_9455_Transcript_3/4_Confidence_0.200_Length_1370, SampleTS1_Locus_4303_Transcript_9/11_Confidence_0.167_Length_1464, Sample6_Locus_8395_Transcript_14/17_Confidence_0.508_Length_1568
**Cutin, suberine and wax biosynthesis**	map00073	2	ec:1.2.1.50 acylprotein thioester reductase, ec:2.3.1.20 Oacyltransferase	1, 2	SampleTS3_Locus_11808_Transcript_9/11_Confidence_0.565_Length_1006, Sample1_Locus_18933_Transcript_2/3_Confidence_0.714_Length_599, Sample3_Locus_18000_Transcript_1/1_Confidence_0.000_Length_845

**Carbapenem biosynthesis**	map00332	2	ec:2.7.2.11 5kinase, ec:1.2.1.41 dehydrogenase	2, 3	Sample3_Locus_23456_Transcript_1/1_Confidence_0.000_Length_1406, SampleTS2_Locus_20780_Transcript_2/3_Confidence_0.600_Length_1460 , Sample3_Locus_23456_Transcript_1/1_Confidence_0.000_Length_1406, Sample1_Locus_3087_Transcript_2/3_Confidence_0.333_Length_2354, SampleTS2_Locus_20780_Transcript_2/3_Confidence_0.600_Length_1460
**Glycosphingo lipid biosynthesis lacto and neolacto series**	map00601	2	ec:2.4.99.4 alpha2,3sialyltr ansferase, ec:2.4.99.8 alpha2,8sialyltr ansferase	10, 3	Sample6_Locus_1667_Transcript_21/40_Confidence_0.198_Length_1348, Sample6_Locus_6485_Transcript_12/18_Confidence_0.490_Length_1417, Sample6_Locus_22436_Transcript_2/2_Confidence_0.333_Length_1831, Sample3_Locus_191_Transcript_86/118_Confidence_0.018_Length_2298, Sample3_Locus_19889_Transcript_1/1_Confidence_0.000_Length_1128, SampleTS2_Locus_4156_Transcript_29/43_Confidence_0.097_Length_121 5, SampleTS1_Locus_10921_Transcript_5/7_Confidence_0.444_Length_843, Sample6_Locus_28634_Transcript_1/2_Confidence_0.333_Length_832, SampleTS1_Locus_13929_Transcript_3/7_Confidence_0.091_Length_1486 , SampleTS1_Locus_6551_Transcript_6/13_Confidence_0.111_Length_1643 , Sample6_Locus_6485_Transcript_12/18_Confidence_0.490_Length_1417, Sample6_Locus_14905_Transcript_12/13_Confidence_0.077_Length_1757 , Sample6_Locus_28634_Transcript_1/2_Confidence_0.333_Length_832
**Caffeine metabolism**	map00232	1	ec:1.7.3.3 urate hydroxylase	1	Sample6_Locus_2272_Transcript_5/6_Confidence_0.200_Length_1048
**Fluorobenzoa te degradation**	map00364	1	ec:4.2.1.84 hydratase	1	Sample3_Locus_12929_Transcript_1/1_Confidence_0.000_Length_1898
**Lipoic acid metabolism**	map00785	1	ec:2.8.1.8 synthase	1	SampleTS3_Locus_7280_Transcript_3/4_Confidence_0.333_Length_1159
**Toluene degradation**	map00623	1	ec:1.1.1.35 dehydrogenase	3	SampleTS1_Locus_789_Transcript_9/11_Confidence_0.238_Length_2300, SampleTS3_Locus_1827_Transcript_3/3_Confidence_0.000_Length_1033, SampleTS3_Locus_11808_Transcript_9/11_Confidence_0.565_Length_100 6
**Monoterpen oid biosynthesis**	map00902	1	ec:1.3.1.81 reductase	1	SampleTS2_Locus_276_Transcript_13/14_Confidence_0.206_Length_1306

**Glycosaminoglycan biosynthesis keratan sulfate**	map00533	1	ec:2.4.99.4 alpha2,3sialyltransferase	10	Sample6_Locus_1667_Transcript_21/40_Confidence_0.198_Length_1348, Sample6_Locus_6485_Transcript_12/18_Confidence_0.490_Length_1417, Sample6_Locus_22436_Transcript_2/2_Confidence_0.333_Length_1831, Sample3_Locus_191_Transcript_86/118_Confidence_0.018_Length_2298, Sample3_Locus_19889_Transcript_1/1_Confidence_0.000_Length_1128, SampleTS2_Locus_4156_Transcript_29/43_Confidence_0.097_Length_1215, SampleTS1_Locus_10921_Transcript_5/7_Confidence_0.444_Length_843, Sample6_Locus_28634_Transcript_1/2_Confidence_0.333_Length_832, SampleTS1_Locus_13929_Transcript_3/7_Confidence_0.091_Length_1486, SampleTS1_Locus_6551_Transcript_6/13_Confidence_0.111_Length_1643
**Th1 and Th2 cell differentiation**	map04658	1	ec:3.1.3.16 phosphatase	49	SampleTS3_Locus_1236_Transcript_8/14_Confidence_0.379_Length_696, SampleTS1_Locus_5854_Transcript_5/5_Confidence_0.400_Length_861, Sample3_Locus_4609_Transcript_5/8_Confidence_0.444_Length_1430, SampleTS3_Locus_1630_Transcript_4/8_Confidence_0.500_Length_1635, SampleTS2_Locus_4720_Transcript_3/7_Confidence_0.667_Length_1404, Sample3_Locus_5932_Transcript_3/4_Confidence_0.571_Length_501, SampleTS3_Locus_13512_Transcript_1/3_Confidence_0.333_Length_1321, SampleTS2_Locus_1774_Transcript_4/5_Confidence_0.500_Length_990, SampleTS2_Locus_549_Transcript_10/36_Confidence_0.207_Length_1084, Sample3_Locus_14090_Transcript_2/4_Confidence_0.714_Length_1345, Sample6_Locus_5727_Transcript_3/3_Confidence_0.000_Length_2301, SampleTS3_Locus_5284_Transcript_10/12_Confidence_0.450_Length_1002, Sample3_Locus_3056_Transcript_6/6_Confidence_0.000_Length_1859, Sample3_Locus_13102_Transcript_1/2_Confidence_0.333_Length_1552, Sample1_Locus_1599_Transcript_1/1_Confidence_0.000_Length_563, SampleTS3_Locus_9298_Transcript_1/10_Confidence_0.238_Length_793, SampleTS1_Locus_2823_Transcript_2/10_Confidence_0.444_Length_2044, Sample6_Locus_4221_Transcript_5/15_Confidence_0.231_Length_1620, Sample1_Locus_9346_Transcript_2/6_Confidence_0.500_Length_949, Sample3_Locus_23182_Transcript_4/4_Confidence_0.000_Length_3946, SampleTS2_Locus_4899_Transcript_2/5_Confidence_0.357_Length_658, Sample6_Locus_594_Transcript_17/21_Confidence_0.471_Length_4353, SampleTS1_Locus_4566_Transcript_4/4_Confidence_0.000_Length_2764, SampleTS2_Locus_2599_Transcript_3/9_Confidence_0.091_Length_4008, SampleTS1_Locus_397_Transcript_66/78_Confidence_0.022_Length_711, Sample1_Locus_9441_Transcript_1/3_Confidence_0.333_Length_1512, SampleTS1_Locus_26651_Transcript_1/1_Confidence_0.000_Length_1107, SampleTS2_Locus_549_Transcript_23/36_Confidence_0.110_Length_766, Sample3_Locus_5257_Transcript_3/8_Confidence_0.333_Length_2904, Sample6_Locus_11565_Transcript_7/13_Confidence_0.097_Length_1502, Sample1_Locus_27168_Transcript_1/2_Confidence_1.000_Length_653, Sample6_Locus_24750_Transcript_4/9_Confidence_0.222_Length_785, SampleTS2_Locus_2994_Transcript_7/9_Confidence_0.235_Length_2922, Sample3_Locus_2926_Transcript_2/3_Confidence_0.750_Length_1774, SampleTS3_Locus_23494_Transcript_1/1_Confidence_0.000_Length_1156, Sample3_Locus_23102_Transcript_1/2_Confidence_0.667_Length_1067, Sample6_Locus_8346_Transcript_7/13_Confidence_0.118_Length_1292, SampleTS1_Locus_12977_Transcript_4/6_Confidence_0.429_Length_1160, SampleTS1_Locus_6027_Transcript_7/14_Confidence_0.160_Length_504, Sample3_Locus_22654_Transcript_1/1_Confidence_0.000_Length_1356, Sample6_Locus_1159_Transcript_30/32_Confidence_0.027_Length_1920, SampleTS3_Locus_2277_Transcript_3/5_Confidence_0.400_Length_1847, SampleTS1_Locus_30500_Transcript_2/2_Confidence_0.850_Length_548, Sample6_Locus_30120_Transcript_2/3_Confidence_0.667_Length_1321, SampleTS2_Locus_3992_Transcript_15/16_Confidence_0.143_Length_1808, SampleTS1_Locus_11573_Transcript_5/10_Confidence_0.211_Length_111

					7, Sample1_Locus_25057_Transcript_2/6_Confidence_0.727_Length_1459, SampleTS2_Locus_549_Transcript_22/36_Confidence_0.134_Length_768, Sample1_Locus_13613_Transcript_6/7_Confidence_0.111_Length_1026
**betaLactam resistance**	map01501	1	ec:3.5.2.6 penicillinase	1	SampleTS3_Locus_23179_Transcript_2/6_Confidence_0.500_Length_1144
**Dioxin degradation**	map00621	1	ec:1.2.1.10 dehydrogenase (acetylating)	2	SampleTS3_Locus_400_Transcript_6/29_Confidence_0.422_Length_2479, Sample6_Locus_1524_Transcript_26/30_Confidence_0.185_Length_3215
**Primary bile acid biosynthesis**	map00120	1	ec:1.1.1.35 dehydrogenase	3	SampleTS1_Locus_789_Transcript_9/11_Confidence_0.238_Length_2300, SampleTS3_Locus_1827_Transcript_3/3_Confidence_0.000_Length_1033, SampleTS3_Locus_11808_Transcript_9/11_Confidence_0.565_Length_1006
**Glycosaminoglycan degradation**	map00531	1	ec:3.2.1.23 lactase (ambiguous)	1	SampleTS2_Locus_9286_Transcript_1/1_Confidence_0.000_Length_2119
**Peptidoglycan biosynthesis**	map00550	1	ec:2.4.1.227 betaNacetylglucosaminyltransferase	1	Sample1_Locus_1062_Transcript_3/5_Confidence_0.286_Length_1312
**Mannose type Oglycan biosynthesis**	map00515	1	ec:2.4.1.109 mannosyltransferase	1	Sample6_Locus_2970_Transcript_1/3_Confidence_0.600_Length_1024
**Benzoxazinoid biosynthesis**	map00402	1	ec:4.1.2.8 lyase	2	SampleTS3_Locus_9447_Transcript_3/3_Confidence_0.000_Length_886, SampleTS1_Locus_10894_Transcript_1/1_Confidence_0.000_Length_652

**Glycosylphosphatidylinositol (GPI)anchor biosynthesis**	map00563	1	ec:2.4.1.198 Nacetylglucosaminyltransferase	1	Sample6_Locus_10518_Transcript_12/15_Confidence_0.059_Length_418
**Synthesis and degradation of ketone bodies**	map00072	1	ec:2.3.1.9 Cacetyltransferase	1	SampleTS2_Locus_1750_Transcript_17/20_Confidence_0.182_Length_1568
**Carotenoid biosynthesis**	map00906	1	ec:1.13.11.51 dioxygenase	1	SampleTS1_Locus_8076_Transcript_4/6_Confidence_0.190_Length_1521
**Phenazine biosynthesis**	map00405	1	ec:4.1.3.27 synthase	1	Sample1_Locus_5305_Transcript_3/12_Confidence_0.143_Length_2221
**Penicillin and cephalosporin biosynthesis**	map00311	1	ec:3.5.2.6 penicillinase	1	SampleTS3_Locus_23179_Transcript_2/6_Confidence_0.500_Length_1144
**Ethylbenzene degradation**	map00642	1	ec:2.3.1.16 Cacyltransferase	1	SampleTS2_Locus_1750_Transcript_17/20_Confidence_0.182_Length_1568
**Aflatoxin biosynthesis**	map00254	1	ec:6.4.1.2 carboxylase	2	Sample3_Locus_8920_Transcript_2/3_Confidence_0.333_Length_2038, SampleTS1_Locus_1155_Transcript_11/16_Confidence_0.625_Length_2240
**Xylene degradation**	map00622	1	ec:1.2.1.10 dehydrogenase (acetylating)	2	SampleTS3_Locus_400_Transcript_6/29_Confidence_0.422_Length_2479, Sample6_Locus_1524_Transcript_26/30_Confidence_0.185_Length_3215
**Naphthalene degradation**	map00626	1	ec:1.1.1.1 dehydrogenase	5	SampleTS2_Locus_4874_Transcript_6/12_Confidence_0.158_Length_1042, Sample6_Locus_1524_Transcript_26/30_Confidence_0.185_Length_3215, SampleTS2_Locus_263_Transcript_1/3_Confidence_0.250_Length_2184, SampleTS3_Locus_400_Transcript_6/29_Confidence_0.422_Length_2479, SampleTS3_Locus_23264_Transcript_1/3_Confidence_0.500_Length_845
**Biosynthesis of 12, 14 and 16membered macrolides**	map00522	1	ec:2.3.1.94 synthase	2	Sample3_Locus_4157_Transcript_2/4_Confidence_0.333_Length_2315, Sample6_Locus_8953_Transcript_13/14_Confidence_0.143_Length_4871
**Diterpenoid biosynthesis**	map00904	1	ec:1.14.11.13 2betadioxygenase	1	Sample6_Locus_445_Transcript_22/46_Confidence_0.251_Length_1324
**DGlutamine and Dglutamate metabolism**	map00471	1	ec:3.5.1.2 glutaminase I	2	Sample6_Locus_3307_Transcript_18/23_Confidence_0.163_Length_735, SampleTS1_Locus_5169_Transcript_10/19_Confidence_0.593_Length_3162
**Glucosinolate biosynthesis**	map00966	1	ec:2.6.1.42 transaminase	2	Sample1_Locus_18777_Transcript_2/4_Confidence_0.923_Length_1416, Sample3_Locus_5952_Transcript_4/4_Confidence_0.000_Length_2586
**Biosynthesis of ansamycins**	map01051	1	ec:2.2.1.1 glycolaldehydetransferase	1	SampleTS1_Locus_16129_Transcript_1/1_Confidence_0.000_Length_1960

Table S5. DEG id, log2 Fold Change, adjusted P value (padj), sequence description and enzyme codes (|LogFC| > 2, padj ≤ 0.01).

id	log2FoldChange	padj	NCBI_NR db description
Sample3_Locus_20852_Transcript_1/3_Confidence_1.000_Length_655	11,00058027	3,54E-56	---NA---
SampleTS1_Locus_12838_Transcript_3/6_Confidence_0.692_Length_1084	9,272699262	6,12E-05	---NA---
Sample3_Locus_8235_Transcript_1/4_Confidence_0.750_Length_1411	9,038398808	4,64E-40	---NA---
SampleTS1_Locus_5177_Transcript_12/12_Confidence_0.410_Length_2433	8,167685601	1,66E-60	protein
Sample1_Locus_24293_Transcript_7/7_Confidence_0.000_Length_1198	7,575327956	4,79E-12	hypothetical protein COCSUDRAFT_66438
SampleTS3_Locus_11672_Transcript_5/5_Confidence_0.417_Length_1173	7,420877852	3,54E-13	---NA---
SampleTS2_Locus_1842_Transcript_1/10_Confidence_0.533_Length_1424	7,034106596	3,68E-28	---NA---
SampleTS3_Locus_1202_Transcript_5/26_Confidence_0.033_Length_352	6,845761329	1,84E-08	---NA---
SampleTS1_Locus_23003_Transcript_3/9_Confidence_0.094_Length_3741	6,844101128	4,32E-64	extracellular ligand-binding receptor
Sample1_Locus_7779_Transcript_5/5_Confidence_0.125_Length_980	6,514125389	1,90E-82	---NA---
Sample3_Locus_17655_Transcript_7/11_Confidence_0.459_Length_815	6,439711887	4,73E-181	abc transporter substrate-binding protein
Sample3_Locus_6_Transcript_2/29_Confidence_0.022_Length_2158	6,231044617	3,72E-15	elmo domain-containing protein 3-like
SampleTS2_Locus_3108_Transcript_9/19_Confidence_0.060_Length_1587	6,185250239	3,30E-05	3 5 -cyclic nucleotide phosphodiesterase
SampleTS3_Locus_4789_Transcript_2/8_Confidence_0.586_Length_584	6,161787176	0,000103912	---NA---
SampleTS3_Locus_1064_Transcript_8/31_Confidence_0.339_Length_1913	6,038403818	3,19E-09	---NA---
Sample1_Locus_4643_Transcript_15/21_Confidence_0.197_Length_332	5,750682616	5,29E-06	---NA---
Sample3_Locus_14002_Transcript_1/1_Confidence_0.000_Length_1044	5,724777188	8,40E-51	rna recognition motif containing family protein
Sample6_Locus_15296_Transcript_1/1_Confidence_0.000_Length_1123	5,722904742	0,001226458	---NA---
SampleTS1_Locus_354_Transcript_3/28_Confidence_0.108_Length_974	5,685002112	1,08E-05	---NA---

Sample1_Locus_5497_Transcript_3/3_Confidence_0.000_Length_1552	5,676357369	0,000206657	---NA---
Sample1_Locus_795_Transcript_3/5_Confidence_0.500_Length_1893	5,423680459	1,75E-18	protein phosphatase 2c family protein
Sample6_Locus_9744_Transcript_1/1_Confidence_0.000_Length_1559	5,374003405	4,63E-08	camk camk1 protein kinase
SampleTS1_Locus_473_Transcript_15/18_Confidence_0.053_Length_496	5,368551796	4,16E-06	---NA---
SampleTS3_Locus_1274_Transcript_12/77_Confidence_0.082_Length_1404	5,253418477	6,29E-05	---NA---
SampleTS1_Locus_388_Transcript_6/6_Confidence_0.111_Length_1488	5,224590955	1,02E-15	phosphoenolpyruvate carboxylase kinase
Sample6_Locus_3042_Transcript_1/1_Confidence_0.000_Length_1466	5,185237355	6,65E-07	---NA---
SampleTS1_Locus_15981_Transcript_1/1_Confidence_0.000_Length_1004	5,101393609	1,74E-08	---NA---
Sample3_Locus_4004_Transcript_7/9_Confidence_0.400_Length_2113	4,98520081	3,76E-92	family 3 adenylate cyclase
Sample1_Locus_402_Transcript_15/20_Confidence_0.122_Length_761	4,930155696	5,29E-210	---NA---
SampleTS1_Locus_18205_Transcript_5/6_Confidence_0.200_Length_1443	4,874903924	0,001287728	---NA---
Sample3_Locus_6151_Transcript_16/22_Confidence_0.102_Length_1626	4,874014209	0	squamosa promoter-binding-like protein 7 isoform x2
Sample6_Locus_4919_Transcript_1/4_Confidence_0.429_Length_528	4,807787641	0,000769722	---NA---
SampleTS2_Locus_4557_Transcript_3/5_Confidence_0.500_Length_885	4,800900533	0,007191059	---NA---
Sample6_Locus_7983_Transcript_4/5_Confidence_0.654_Length_702	4,752754199	1,26E-42	---NA---
SampleTS2_Locus_1815_Transcript_7/9_Confidence_0.462_Length_603	4,745615926	3,25E-05	domon domain containing protein
Sample1_Locus_8729_Transcript_1/3_Confidence_0.714_Length_626	4,69530485	2,87E-181	---NA---
SampleTS1_Locus_10104_Transcript_2/4_Confidence_0.500_Length_1246	4,690470804	4,17E-10	---NA---
SampleTS2_Locus_507_Transcript_73/128_Confidence_0.005_Length_410	4,609547522	1,18E-43	---NA---
SampleTS2_Locus_1041_Transcript_6/9_Confidence_0.091_Length_927	4,552978065	0,000825272	---NA---
Sample3_Locus_61_Transcript_3/13_Confidence_0.000_Length_1058	4,552973485	5,29E-06	---NA---
Sample3_Locus_6097_Transcript_2/4_Confidence_0.500_Length_1588	4,552961366	0,008754617	protein
SampleTS1_Locus_3282_Transcript_21/28_Confidence_0.206_Length_1494	4,523237383	6,03E-42	serine threonine-protein kinase atr-like

SampleTS1_Locus_86_Transcript_74/166_Confidence_0.033_Length_1152	4,485243868	2,07E-77	abc transporter substrate-binding protein
SampleTS3_Locus_1222_Transcript_6/17_Confidence_0.231_Length_654	4,457100747	0,000113333	---NA---
SampleTS3_Locus_247_Transcript_66/83_Confidence_0.046_Length_451	4,454910632	0,001473552	---NA---
Sample3_Locus_12405_Transcript_3/6_Confidence_0.333_Length_1010	4,429346806	0,00E+00	---NA---
SampleTS1_Locus_5902_Transcript_6/10_Confidence_0.467_Length_700	4,418672122	0,003194545	---NA---
SampleTS2_Locus_2823_Transcript_10/10_Confidence_0.405_Length_406	4,37907043	3,83E-10	---NA---
SampleTS1_Locus_9838_Transcript_6/9_Confidence_0.444_Length_1154	4,323308294	1,39E-86	---NA---
Sample6_Locus_4680_Transcript_3/4_Confidence_0.200_Length_733	4,304282891	3,01E-05	---NA---
SampleTS2_Locus_7300_Transcript_3/4_Confidence_0.368_Length_958	4,288937983	1,60E-25	---NA---
SampleTS1_Locus_5159_Transcript_10/13_Confidence_0.378_Length_1753	4,269170702	4,92E-07	f-box lrr-repeat protein 17
SampleTS3_Locus_976_Transcript_24/26_Confidence_0.657_Length_846	4,264471315	0,00022215	---NA---
SampleTS3_Locus_3474_Transcript_2/7_Confidence_0.429_Length_450	4,238536205	0,004028801	---NA---
SampleTS3_Locus_6276_Transcript_4/6_Confidence_0.409_Length_852	4,23449739	0,000107383	---NA---
Sample1_Locus_24078_Transcript_6/7_Confidence_0.150_Length_1572	4,213763115	0,000253496	adenylate kinase
SampleTS2_Locus_530_Transcript_56/383_Confidence_0.013_Length_411	4,207261248	9,54E-14	---NA---
Sample6_Locus_13760_Transcript_5/10_Confidence_0.500_Length_710	4,167980532	1,68E-15	---NA---
Sample6_Locus_4723_Transcript_20/38_Confidence_0.053_Length_830	4,157757745	3,99E-45	---NA---
Sample3_Locus_17568_Transcript_1/1_Confidence_0.000_Length_1894	4,137918794	2,19E-13	solute carrier family 39 protein
Sample1_Locus_5360_Transcript_6/10_Confidence_0.302_Length_4884	3,993533657	1,73E-05	nadph--cytochrome p450 reductase
Sample1_Locus_9671_Transcript_4/10_Confidence_0.414_Length_2545	3,981265506	8,06E-28	importin alpha

SampleTS1_Locus_3421_Transcript_7/10_Confidence_0.383_Length_971	3,968010106	0,005762551	ring finger protein 185
SampleTS3_Locus_609_Transcript_21/29_Confidence_0.484_Length_1719	3,948448823	3,67E-35	myb-like dna-binding partial
SampleTS2_Locus_3140_Transcript_18/19_Confidence_0.323_Length_745	3,947250341	3,81E-13	acylpyruvase mitochondrial-like
Sample3_Locus_5077_Transcript_25/32_Confidence_0.104_Length_1090	3,94069838	1,38E-06	squamosa promoter binding protein
SampleTS1_Locus_676_Transcript_1/12_Confidence_0.138_Length_1355	3,900888479	2,24E-07	---NA---
Sample1_Locus_1934_Transcript_5/10_Confidence_0.467_Length_1095	3,898467813	0,001875472	protein
SampleTS3_Locus_1786_Transcript_19/20_Confidence_0.344_Length_522	3,892102289	6,89E-13	---NA---
Sample1_Locus_12346_Transcript_7/13_Confidence_0.105_Length_1680	3,889242287	2,49E-10	---NA---
Sample1_Locus_8365_Transcript_1/1_Confidence_0.000_Length_1005	3,855520931	0,008311582	---NA---
SampleTS1_Locus_2950_Transcript_5/8_Confidence_0.211_Length_995	3,845756354	0,006967243	---NA---
SampleTS3_Locus_8258_Transcript_4/7_Confidence_0.500_Length_1732	3,839841491	0,001580641	adp-ribosylation factor
SampleTS1_Locus_86_Transcript_39/166_Confidence_0.018_Length_398	3,820443392	7,84E-26	---NA---
SampleTS2_Locus_195_Transcript_54/71_Confidence_0.121_Length_1499	3,816008864	0,001898467	squamosa promoter binding protein
SampleTS2_Locus_8381_Transcript_4/6_Confidence_0.562_Length_1179	3,815990442	0,000454609	---NA---
SampleTS1_Locus_8647_Transcript_11/12_Confidence_0.267_Length_817	3,76028625	2,15E-12	hypothetical protein
SampleTS1_Locus_2668_Transcript_1/1_Confidence_0.333_Length_615	3,736274682	4,01E-16	heat shock protein hsp20
SampleTS2_Locus_695_Transcript_28/28_Confidence_0.108_Length_683	3,700921599	1,18E-12	protein
SampleTS3_Locus_11_Transcript_354/519_Confidence_0.023_Length_1120	3,692519031	1,72E-08	---NA---
Sample1_Locus_6199_Transcript_3/7_Confidence_0.588_Length_1175	3,623361809	0,002045693	nuclear ribonuclease z
SampleTS3_Locus_1176_Transcript_9/16_Confidence_0.101_Length_1170	3,598160952	0,001572143	gag-pol polyprotein

SampleTS2_Locus_195_Transcript_16/71_Confidence_0.058_Length_700	3,58859196	3,98E-06	---NA---
Sample6_Locus_4545_Transcript_7/7_Confidence_0.125_Length_429	3,569359151	3,00E-42	---NA---
SampleTS2_Locus_703_Transcript_26/30_Confidence_0.190_Length_791	3,533919861	2,84E-23	secreted protein
SampleTS2_Locus_1685_Transcript_8/23_Confidence_0.133_Length_874	3,504058254	1,48E-06	---NA---
SampleTS2_Locus_195_Transcript_55/71_Confidence_0.194_Length_1091	3,497820011	0,005095588	squamosa promoter binding protein
SampleTS3_Locus_877_Transcript_5/9_Confidence_0.174_Length_661	3,487927071	9,84E-45	---NA---
SampleTS2_Locus_9696_Transcript_3/6_Confidence_0.200_Length_356	3,468852287	0,001285682	---NA---
Sample6_Locus_5040_Transcript_19/19_Confidence_0.048_Length_789	3,427644893	4,37E-93	secreted protein
SampleTS1_Locus_1262_Transcript_78/105_Confidence_0.012_Length_939	3,390468749	1,04E-10	---NA---
SampleTS3_Locus_11902_Transcript_9/10_Confidence_0.032_Length_770	3,37626144	6,33E-103	---NA---
Sample6_Locus_15577_Transcript_4/6_Confidence_0.364_Length_1541	3,272070898	0,002568998	---NA---
Sample6_Locus_18094_Transcript_4/6_Confidence_0.357_Length_1047	3,211932069	2,59E-06	---NA---
SampleTS1_Locus_3020_Transcript_3/12_Confidence_0.452_Length_526	3,183212706	3,03E-07	---NA---
SampleTS2_Locus_2631_Transcript_10/37_Confidence_0.203_Length_869	3,180996262	5,12E-15	protein farnesyltransferase geranylgeranyltransferase type-1 subunit alpha-like
SampleTS1_Locus_6680_Transcript_1/4_Confidence_0.667_Length_691	3,178578125	1,72E-06	---NA---
SampleTS3_Locus_3474_Transcript_3/7_Confidence_0.714_Length_1365	3,178564289	0,000119025	cysteine hydrolase
Sample3_Locus_5321_Transcript_3/12_Confidence_0.381_Length_1101	3,163350325	1,21E-07	homospermidine synthase
Sample1_Locus_2553_Transcript_8/8_Confidence_0.474_Length_1256	3,148578868	0,003985246	---NA---
Sample1_Locus_2130_Transcript_13/15_Confidence_0.148_Length_731	3,141258685	1,66E-23	hypothetical protein CHLNCDRAFT_53756
SampleTS1_Locus_6044_Transcript_1/2_Confidence_0.333_Length_684	3,121804693	0,000972559	---NA---

SampleTS2_Locus_1645_Transcript_3/6_Confidence_0.583_Length_935	3,093524336	0,001561769	---NA---
SampleTS3_Locus_10254_Transcript_7/14_Confidence_0.227_Length_477	3,083475104	2,67E-05	---NA---
Sample3_Locus_18643_Transcript_2/4_Confidence_0.200_Length_574	3,083472591	0,005577185	---NA---
Sample6_Locus_57_Transcript_17/25_Confidence_0.207_Length_851	3,072336584	0,001011434	---NA---
Sample1_Locus_28735_Transcript_4/4_Confidence_0.000_Length_825	3,069755266	0,008894724	hypothetical protein COCSUDRAFT_20202
SampleTS1_Locus_1262_Transcript_86/105_Confidence_0.017_Length_3863	3,067883509	3,18E-19	protein
Sample1_Locus_3814_Transcript_1/13_Confidence_0.097_Length_852	3,057547642	4,55E-59	---NA---
SampleTS1_Locus_5495_Transcript_4/19_Confidence_0.208_Length_655	3,053038209	0,000359872	---NA---
SampleTS2_Locus_2228_Transcript_13/14_Confidence_0.486_Length_2575	3,045359162	7,81E-14	protein
Sample6_Locus_4581_Transcript_15/18_Confidence_0.479_Length_1534	3,038393915	0,008905321	fas1 domain-containing protein
Sample1_Locus_6002_Transcript_28/30_Confidence_0.082_Length_1834	3,03408767	8,37E-76	mitogen-activated protein kinase 14
Sample3_Locus_6713_Transcript_13/16_Confidence_0.526_Length_1612	3,021812066	6,87E-09	ash1-related protein isoform 1
Sample3_Locus_14476_Transcript_2/6_Confidence_0.688_Length_618	3,01653083	3,43E-05	---NA---
SampleTS2_Locus_7428_Transcript_8/11_Confidence_0.720_Length_720	3,015315157	7,16E-05	---NA---
SampleTS1_Locus_516_Transcript_35/37_Confidence_0.055_Length_1497	2,989306495	1,55E-130	---NA---
SampleTS3_Locus_13701_Transcript_3/3_Confidence_0.000_Length_845	2,985918215	0,000231094	---NA---
Sample1_Locus_1446_Transcript_8/28_Confidence_0.128_Length_416	2,977132471	3,05E-56	---NA---
Sample3_Locus_14059_Transcript_4/5_Confidence_0.538_Length_512	2,974863166	2,29E-13	---NA---
Sample3_Locus_633_Transcript_2/5_Confidence_0.400_Length_2278	2,955934096	0,000114635	histidine partial
SampleTS3_Locus_7941_Transcript_13/15_Confidence_0.387_Length_1160	2,89602593	1,61E-55	---NA---
SampleTS2_Locus_8890_Transcript_3/7_Confidence_0.200_Length_515	2,878736504	0,001045399	---NA---
SampleTS3_Locus_3853_Transcript_12/20_Confidence_0.564_Length_838	2,842964108	2,19E-09	phosphate abc transporter substrate-binding protein

SampleTS2_Locus_3898_Transcript_4/10_Confidence_0.292_Length_604	2,834733281	0,001483127	---NA---
Sample1_Locus_15530_Transcript_1/3_Confidence_0.333_Length_758	2,834379068	0,003885652	---NA---
SampleTS1_Locus_610_Transcript_52/54_Confidence_0.210_Length_1630	2,81778096	6,25E-12	protein farnesyltransferase geranylgeranyltransferase type-1 subunit alpha
SampleTS2_Locus_1897_Transcript_7/15_Confidence_0.260_Length_342	2,803970215	9,42E-36	---NA---
SampleTS1_Locus_12878_Transcript_5/5_Confidence_0.500_Length_1044	2,785632444	0,001478727	---NA---
SampleTS1_Locus_8506_Transcript_4/14_Confidence_0.133_Length_1659	2,785621541	0,000207062	surface antigen protein
Sample1_Locus_9621_Transcript_9/12_Confidence_0.737_Length_852	2,782449619	8,82E-06	conserved unknown protein
Sample3_Locus_562_Transcript_4/6_Confidence_0.200_Length_1219	2,767092725	0,000127957	serine decarboxylase
SampleTS3_Locus_4287_Transcript_13/14_Confidence_0.511_Length_695	2,761202375	8,42E-19	protein
Sample6_Locus_6229_Transcript_18/18_Confidence_0.000_Length_333	2,756532442	1,95E-05	---NA---
Sample3_Locus_8866_Transcript_2/4_Confidence_0.714_Length_1157	2,741990348	0,001796286	phosphoribosylformylglycinamidine cyclo- chloroplastic mitochondrial-like
SampleTS3_Locus_6772_Transcript_12/17_Confidence_0.238_Length_1568	2,740894093	0,000194914	ammonium transporter
SampleTS1_Locus_516_Transcript_31/37_Confidence_0.047_Length_1839	2,738516854	1,37E-08	hypothetical protein THAOC_06802
SampleTS2_Locus_4531_Transcript_10/17_Confidence_0.583_Length_2220	2,716468863	0,003916901	protein phosphatase 2c family protein
SampleTS1_Locus_23448_Transcript_1/1_Confidence_0.000_Length_1309	2,709084072	0,000419326	---NA---
Sample1_Locus_220_Transcript_22/33_Confidence_0.024_Length_646	2,661675765	0,00038967	---NA---
SampleTS3_Locus_1202_Transcript_26/26_Confidence_0.120_Length_1120	2,646073802	0,000781183	hypothetical protein
Sample6_Locus_25087_Transcript_4/5_Confidence_0.025_Length_1072	2,635431594	0,001220222	---NA---
SampleTS1_Locus_9225_Transcript_1/6_Confidence_0.111_Length_948	2,627392983	0,00019496	---NA---
Sample1_Locus_2443_Transcript_1/6_Confidence_0.400_Length_603	2,612464169	0,002038857	probable histone h2a variant 3

SampleTS3_Locus_331_Transcript_2/14_Confidence_0.077_Length_1738	2,591674326	5,75E-24	protein
SampleTS1_Locus_443_Transcript_19/20_Confidence_0.091_Length_2874	2,590468312	2,53E-42	gram domain-containing protein 1c-like isoform x3
Sample1_Locus_3_Transcript_74/103_Confidence_0.006_Length_500	2,568757697	2,51E-27	---NA---
SampleTS3_Locus_137_Transcript_34/48_Confidence_0.006_Length_1072	2,517720051	1,01E-07	probable anion transporter 5
SampleTS3_Locus_13896_Transcript_13/14_Confidence_0.021_Length_1036	2,517088207	1,33E-07	---NA---
SampleTS3_Locus_8946_Transcript_7/8_Confidence_0.091_Length_2134	2,511479159	2,52E-14	abc subfamily abcg
SampleTS1_Locus_275_Transcript_59/85_Confidence_0.013_Length_590	2,510060978	7,90E-43	dihydropyrimidinase-like isoform x1
SampleTS3_Locus_487_Transcript_25/26_Confidence_0.122_Length_1071	2,501129912	7,84E-05	catalase peroxidase hpi
SampleTS1_Locus_6151_Transcript_8/10_Confidence_0.067_Length_1404	2,497085548	2,44E-06	hypothetical protein VOLCADRAFT_106216
SampleTS3_Locus_22036_Transcript_4/4_Confidence_0.556_Length_688	2,444035058	0,003342381	a-macroglobulin complement component family protein
SampleTS1_Locus_5389_Transcript_15/21_Confidence_0.245_Length_680	2,436708951	0,00061285	---NA---
SampleTS1_Locus_6263_Transcript_6/10_Confidence_0.040_Length_1038	2,436061691	1,56E-86	sugar transport protein 13-like
Sample6_Locus_12619_Transcript_13/16_Confidence_0.133_Length_424	2,422122351	0,004952789	---NA---
Sample3_Locus_1362_Transcript_18/18_Confidence_0.222_Length_1478	2,416147762	4,20E-63	myb domain protein
SampleTS3_Locus_148_Transcript_514/701_Confidence_0.006_Length_309	2,393970507	0,008259093	---NA---
SampleTS1_Locus_920_Transcript_114/207_Confidence_0.025_Length_847	2,379499609	0,000177177	hypothetical protein EMIHUDRAFT_357722, partial
SampleTS3_Locus_5673_Transcript_4/8_Confidence_0.200_Length_1545	2,376243061	2,22E-53	---NA---
Sample1_Locus_9463_Transcript_1/13_Confidence_0.500_Length_687	2,357477873	0,009969876	---NA---
Sample3_Locus_4983_Transcript_5/14_Confidence_0.667_Length_804	2,327482771	6,60E-14	---NA---
Sample6_Locus_16581_Transcript_8/12_Confidence_0.467_Length_2159	2,306730516	3,02E-31	b3 domain-containing protein os07g0563300-like

SampleTS1_Locus_51_Transcript_16/22_Confidence_0.205_Length_763	2,285485095	0,004561129	---NA---
SampleTS3_Locus_22037_Transcript_4/5_Confidence_0.500_Length_442	2,284319504	1,16E-15	---NA---
SampleTS2_Locus_3486_Transcript_11/18_Confidence_0.365_Length_1202	2,269655938	6,90E-11	---NA---
Sample6_Locus_585_Transcript_21/34_Confidence_0.265_Length_974	2,246669092	3,06E-48	glycosyl transferase
Sample3_Locus_21605_Transcript_3/4_Confidence_0.250_Length_1123	2,244335936	0,005146243	---NA---
SampleTS1_Locus_12050_Transcript_2/7_Confidence_0.588_Length_496	2,215541987	3,31E-09	---NA---
SampleTS3_Locus_2974_Transcript_5/19_Confidence_0.108_Length_524	2,211975537	3,04E-60	---NA---
Sample6_Locus_7770_Transcript_5/7_Confidence_0.688_Length_1460	2,203382026	9,05E-07	caax prenyl protease 2
Sample3_Locus_3879_Transcript_3/17_Confidence_0.386_Length_704	2,197875876	0,004820274	---NA---
Sample1_Locus_6882_Transcript_13/34_Confidence_0.095_Length_647	2,196511154	5,32E-11	---NA---
SampleTS1_Locus_10121_Transcript_12/12_Confidence_0.238_Length_1237	2,175892875	0,001209132	---NA---
Sample6_Locus_1809_Transcript_20/21_Confidence_0.137_Length_1913	2,170435802	5,15E-72	squamosa promoter-binding protein 1-like
Sample1_Locus_7491_Transcript_4/12_Confidence_0.471_Length_824	2,168819956	3,50E-14	---NA---
Sample1_Locus_8233_Transcript_10/12_Confidence_0.200_Length_942	2,156632758	5,70E-83	rab gtpase-8
Sample3_Locus_3837_Transcript_8/10_Confidence_0.350_Length_1593	2,119734701	8,08E-70	---NA---
SampleTS2_Locus_4645_Transcript_4/6_Confidence_0.650_Length_2189	2,097020904	5,11E-10	---NA---
SampleTS2_Locus_390_Transcript_13/53_Confidence_0.189_Length_597	2,08943898	1,58E-13	---NA---
Sample1_Locus_3216_Transcript_8/22_Confidence_0.061_Length_529	2,085675242	0,003120143	---NA---
Sample3_Locus_17985_Transcript_11/11_Confidence_0.059_Length_915	2,081044045	2,88E-22	reverse transcriptase
Sample1_Locus_4898_Transcript_9/18_Confidence_0.353_Length_1501	2,074501269	3,27E-08	phospholipid scramblase
Sample1_Locus_820_Transcript_7/17_Confidence_0.590_Length_943	2,072226545	2,40E-15	glutathione s-transferase

SampleTS1_Locus_11908_Transcript_4/6_Confidence_0.583_Length_702	2,058457302	9,05E-07	wd-40 repeat-containing protein
Sample1_Locus_9346_Transcript_2/6_Confidence_0.500_Length_949	2,021808088	0,001026853	hypothetical protein VOLCADRAFT_84366
Sample1_Locus_6171_Transcript_31/32_Confidence_0.072_Length_2101	2,007414206	1,57E-51	protein
Sample1_Locus_10546_Transcript_2/3_Confidence_0.333_Length_2690	2,001354941	2,20E-12	kinase domain-containing protein isoform 1
Sample1_Locus_510_Transcript_2/12_Confidence_0.674_Length_501	-2,002214213	0,000237704	---NA---
Sample3_Locus_9600_Transcript_7/7_Confidence_0.350_Length_867	-2,010626488	0,005216032	arginine n-methyltransferase 2
Sample6_Locus_6377_Transcript_8/31_Confidence_0.019_Length_975	-2,022321333	1,95E-13	---NA---
SampleTS3_Locus_8386_Transcript_8/10_Confidence_0.200_Length_553	-2,035893475	1,37E-12	hypothetical protein COCSUDRAFT_53614
Sample3_Locus_7306_Transcript_4/5_Confidence_0.625_Length_572	-2,044460982	2,10E-06	protein
Sample3_Locus_8602_Transcript_7/9_Confidence_0.000_Length_887	-2,04599485	2,28E-10	---NA---
Sample3_Locus_6953_Transcript_9/13_Confidence_0.475_Length_4750	-2,046746686	1,09E-05	xylulose kinase
Sample1_Locus_8793_Transcript_5/12_Confidence_0.469_Length_1642	-2,056542542	0,000939814	---NA---
SampleTS1_Locus_102_Transcript_7/24_Confidence_0.073_Length_1592	-2,064067791	0,000941185	gtp binding protein
SampleTS2_Locus_32_Transcript_9/12_Confidence_0.412_Length_3353	-2,074221988	6,90E-11	chloroplast processing enzyme-like protein
Sample3_Locus_17152_Transcript_4/4_Confidence_0.000_Length_943	-2,074698372	1,59E-14	protein
Sample1_Locus_2956_Transcript_31/33_Confidence_0.039_Length_1166	-2,081134827	0,000521338	---NA---
SampleTS3_Locus_492_Transcript_6/12_Confidence_0.429_Length_1472	-2,096553855	5,07E-24	cysteine proteinase 7
Sample6_Locus_114_Transcript_25/103_Confidence_0.069_Length_1795	-2,097050396	7,08E-16	---NA---
Sample3_Locus_564_Transcript_17/23_Confidence_0.490_Length_2043	-2,099022677	2,61E-08	tubulin tyrosine ligase-like member 5
Sample1_Locus_1235_Transcript_39/59_Confidence_0.206_Length_1120	-2,101639482	0,000236393	peptidase s8
SampleTS1_Locus_7276_Transcript_4/11_Confidence_0.475_Length_666	-2,103386694	0,001306648	cg12276-pa
Sample1_Locus_2584_Transcript_6/14_Confidence_0.500_Length_2401	-2,104250991	0,000284872	membrane protein

Sample6_Locus_6748_Transcript_31/33_Confidence_0.467_Length_2454	-2,125213532	0,000380697	binding protein
Sample6_Locus_16912_Transcript_4/6_Confidence_0.286_Length_1521	-2,125975068	0,003283182	4-diphosphocytidyl-2-c-methyl-d-erythritol kinase
Sample6_Locus_164_Transcript_81/296_Confidence_0.015_Length_2930	-2,131537729	0,00054768	dna annealing helicase and endonuclease zranb3
SampleTS1_Locus_3611_Transcript_4/7_Confidence_0.462_Length_614	-2,139849704	0,000262077	---NA---
SampleTS3_Locus_806_Transcript_24/43_Confidence_0.183_Length_786	-2,142072675	0,000631648	---NA---
SampleTS1_Locus_12176_Transcript_7/17_Confidence_0.314_Length_1653	-2,157206534	1,32E-11	---NA---
Sample6_Locus_4663_Transcript_6/12_Confidence_0.170_Length_579	-2,167511192	0,000315198	---NA---
Sample1_Locus_5968_Transcript_19/21_Confidence_0.286_Length_1498	-2,194782008	4,78E-09	---NA---
Sample3_Locus_1308_Transcript_9/13_Confidence_0.071_Length_370	-2,195842493	3,39E-09	---NA---
SampleTS2_Locus_4824_Transcript_5/6_Confidence_0.273_Length_488	-2,198708593	1,04E-07	---NA---
SampleTS1_Locus_254_Transcript_196/372_Confidence_0.001_Length_443	-2,19882253	8,44E-07	---NA---
SampleTS1_Locus_13851_Transcript_18/18_Confidence_0.024_Length_1396	-2,201924111	0,000149958	---NA---
Sample6_Locus_63_Transcript_47/50_Confidence_0.006_Length_1338	-2,205830977	0,002333506	---NA---
Sample1_Locus_9000_Transcript_6/6_Confidence_0.000_Length_1173	-2,209896637	0,000315198	cytoplasmic protein
SampleTS1_Locus_6361_Transcript_2/3_Confidence_0.143_Length_443	-2,215727952	1,58E-12	---NA---
Sample1_Locus_2707_Transcript_26/33_Confidence_0.394_Length_976	-2,230697099	2,79E-05	---NA---
Sample6_Locus_6789_Transcript_15/28_Confidence_0.060_Length_623	-2,231932516	6,75E-05	---NA---
Sample1_Locus_1235_Transcript_40/59_Confidence_0.388_Length_1852	-2,232761103	0,000850812	peptidase s8
Sample6_Locus_284_Transcript_17/22_Confidence_0.067_Length_1947	-2,233999107	7,63E-10	glycosyl hydrolase
SampleTS3_Locus_14002_Transcript_6/6_Confidence_0.083_Length_934	-2,235105763	8,05E-08	xylosyltransferase 1-like
Sample6_Locus_5920_Transcript_21/36_Confidence_0.090_Length_804	-2,239469172	0,000889011	---NA---

SampleTS1_Locus_3105_Transcript_11/16_Confidence_0.333_Length_1763	-2,250735466	2,44E-09	origin recognition complex subunit 5
Sample3_Locus_11361_Transcript_22/29_Confidence_0.310_Length_825	-2,263280982	0,001485698	sumo-activating enzyme subunit 1a
Sample3_Locus_22_Transcript_30/37_Confidence_0.227_Length_1347	-2,268314269	7,63E-05	protein
SampleTS2_Locus_154_Transcript_3/9_Confidence_0.083_Length_762	-2,277978756	0,005906303	beta tubulin
Sample1_Locus_17297_Transcript_5/12_Confidence_0.429_Length_705	-2,285537041	0,001003556	---NA---
Sample1_Locus_8585_Transcript_6/6_Confidence_0.000_Length_1145	-2,298887546	4,81E-07	short-chain dehydrogenase
Sample1_Locus_10351_Transcript_10/10_Confidence_0.077_Length_635	-2,299476625	6,93E-08	---NA---
Sample1_Locus_2272_Transcript_43/67_Confidence_0.374_Length_2859	-2,309534758	7,16E-05	dihydroxy-acid dehydratase
Sample3_Locus_1335_Transcript_24/25_Confidence_0.041_Length_339	-2,333071121	3,51E-07	---NA---
Sample6_Locus_16758_Transcript_4/8_Confidence_0.500_Length_907	-2,346150962	0,001542052	---NA---
Sample1_Locus_756_Transcript_7/11_Confidence_0.158_Length_3868	-2,34837064	2,81E-05	dna topoisomerase i
Sample3_Locus_4646_Transcript_14/15_Confidence_0.029_Length_1668	-2,352595867	1,54E-10	low quality protein: protein mss51 mitochondrial- partial
SampleTS1_Locus_22837_Transcript_11/14_Confidence_0.526_Length_924	-2,39139727	0,003090512	---NA---
Sample1_Locus_15457_Transcript_19/32_Confidence_0.195_Length_1455	-2,404879208	0,003687535	---NA---
SampleTS2_Locus_12903_Transcript_9/9_Confidence_0.036_Length_1543	-2,412822134	0,009194502	---NA---
Sample6_Locus_2848_Transcript_16/17_Confidence_0.521_Length_1276	-2,416349978	4,54E-06	rhodanese-like protein
SampleTS1_Locus_10387_Transcript_7/8_Confidence_0.100_Length_886	-2,420257487	2,34E-16	---NA---
SampleTS2_Locus_4494_Transcript_52/54_Confidence_0.066_Length_1160	-2,42791585	4,21E-27	flagellar associated protein
Sample3_Locus_1698_Transcript_7/9_Confidence_0.111_Length_1910	-2,428386045	3,18E-11	dna repair helicase xpb2
Sample3_Locus_5595_Transcript_14/17_Confidence_0.357_Length_4068	-2,434689916	5,38E-05	vacuolar protein sorting-associated protein 13
Sample6_Locus_212_Transcript_219/511_Confidence_0.006_Length_869	-2,437344026	0,004951932	---NA---
Sample6_Locus_12426_Transcript_3/4_Confidence_0.400_Length_834	-2,438978198	5,38E-06	peptidyl-prolyl cis-trans isomerase cyp20- chloroplastic-like
Sample3_Locus_95_Transcript_4/17_Confidence_0.131_Length_655	-2,441724945	1,96E-05	---NA---
Sample1_Locus_542_Transcript_8/17_Confidence_0.237_Length_776	-2,445794505	5,04E-07	---NA---
SampleTS2_Locus_4494_Transcript_43/54_Confidence_0.036_Length_2339	-2,44757448	1,44E-32	flagellar associated protein

SampleTS2_Locus_5721_Transcript_5/6_Confidence_0.607_Length_568	-2,452036873	0,003420044	---NA---
Sample1_Locus_698_Transcript_20/51_Confidence_0.176_Length_1608	-2,458266665	0,001473552	lipoxygenase homology domain-containing protein 1-like
Sample3_Locus_6557_Transcript_6/15_Confidence_0.238_Length_1079	-2,472348669	4,28E-06	---NA---
SampleTS1_Locus_4936_Transcript_10/10_Confidence_0.062_Length_1298	-2,495548532	1,00E-22	protein
Sample3_Locus_5271_Transcript_4/11_Confidence_0.609_Length_982	-2,505929498	0,000436114	lipoxygenase homology domain-containing protein 1
Sample1_Locus_5155_Transcript_7/10_Confidence_0.073_Length_908	-2,51231203	0,000263379	protein
Sample6_Locus_9242_Transcript_4/7_Confidence_0.143_Length_489	-2,54340117	5,84E-05	---NA---
Sample1_Locus_12063_Transcript_8/10_Confidence_0.605_Length_1236	-2,54406752	8,81E-05	anaphase-promoting complex subunit 1-like
SampleTS3_Locus_6406_Transcript_2/4_Confidence_0.571_Length_915	-2,546566491	0,000177177	rdx family protein
Sample6_Locus_3169_Transcript_9/10_Confidence_0.095_Length_1673	-2,563047123	7,34E-10	---NA---
Sample1_Locus_891_Transcript_5/17_Confidence_0.605_Length_956	-2,580128926	3,08E-10	---NA---
Sample6_Locus_6014_Transcript_15/22_Confidence_0.088_Length_1106	-2,626940589	0,001444434	---NA---
SampleTS2_Locus_8321_Transcript_17/19_Confidence_0.062_Length_1174	-2,627953921	1,29E-10	---NA---
Sample6_Locus_2327_Transcript_13/56_Confidence_0.080_Length_1002	-2,628934143	6,62E-05	pyruvate kinase
Sample6_Locus_5516_Transcript_4/8_Confidence_0.500_Length_1972	-2,63109958	5,03E-14	signal peptide protein
Sample1_Locus_16475_Transcript_4/9_Confidence_0.667_Length_385	-2,633570838	0,00297646	---NA---
SampleTS1_Locus_1688_Transcript_11/14_Confidence_0.620_Length_1966	-2,637826341	8,67E-11	hypothetical protein AURANDRAFT_72102
SampleTS2_Locus_4463_Transcript_16/21_Confidence_0.644_Length_2034	-2,647681007	3,51E-14	calcineurin-like metallo-phosphoesterase superfamily protein isoform 1
Sample3_Locus_980_Transcript_9/22_Confidence_0.438_Length_635	-2,675859927	0,00049421	---NA---
Sample3_Locus_1064_Transcript_33/42_Confidence_0.288_Length_1479	-2,692183498	0,000160422	calcineurin-like metallo-phosphoesterase-like protein
Sample6_Locus_4215_Transcript_14/29_Confidence_0.069_Length_739	-2,714920494	2,28E-05	threonine synthase
Sample1_Locus_12415_Transcript_7/8_Confidence_0.618_Length_1569	-2,738593424	0,009296495	---NA---
Sample3_Locus_933_Transcript_3/19_Confidence_0.308_Length_408	-2,79092102	6,49E-17	---NA---
Sample3_Locus_6164_Transcript_6/16_Confidence_0.426_Length_1550	-2,827859223	4,17E-07	membrane protein
SampleTS1_Locus_2149_Transcript_13/29_Confidence_0.042_Length_389	-2,848960603	0,000722404	---NA---
Sample1_Locus_681_Transcript_58/104_Confidence_0.018_Length_1409	-2,85121685	6,57E-06	---NA---
Sample6_Locus_1528_Transcript_8/12_Confidence_0.071_Length_642	-2,909010686	8,33E-09	---NA---
Sample3_Locus_10861_Transcript_6/9_Confidence_0.353_Length_1071	-2,918713867	0,000837388	---NA---
SampleTS2_Locus_14071_Transcript_7/7_Confidence_0.000_Length_3360	-2,938889499	0,004501318	3 -cyclic-nucleotide phosphodiesterase

Sample1_Locus_3589_Transcript_4/12_Confidence_0.280_Length_964	-2,944840286	4,45E-32	---NA---
Sample6_Locus_1055_Transcript_5/6_Confidence_0.062_Length_1635	-2,969203727	7,91E-10	tho complex subunit 2-like
Sample1_Locus_33312_Transcript_1/1_Confidence_1.000_Length_501	-3,036442953	0,000223529	---NA---
Sample1_Locus_698_Transcript_45/51_Confidence_0.188_Length_1524	-3,065360527	3,21E-05	lipoxygenase homology domain-containing protein 1
Sample1_Locus_10463_Transcript_11/22_Confidence_0.155_Length_1079	-3,068529817	0,000144037	conserved repeat domain protein
Sample6_Locus_3025_Transcript_27/53_Confidence_0.325_Length_2010	-3,07771913	1,96E-05	alpha- -sialyltransferase st3gal ii
SampleTS1_Locus_24134_Transcript_1/3_Confidence_0.571_Length_859	-3,110005857	0,004689671	---NA---
SampleTS3_Locus_5834_Transcript_5/11_Confidence_0.267_Length_826	-3,113790086	0,000643466	---NA---
Sample1_Locus_12745_Transcript_7/13_Confidence_0.512_Length_1824	-3,128369899	3,01E-05	---NA---
Sample1_Locus_653_Transcript_4/10_Confidence_0.405_Length_667	-3,145336822	0,004820274	hypothetical protein SPRG_08339
SampleTS1_Locus_2195_Transcript_3/4_Confidence_0.500_Length_1846	-3,271468392	0,003465067	protein
Sample3_Locus_4290_Transcript_16/18_Confidence_0.500_Length_1185	-3,303889696	0,003311505	soluble starch synthase iii-2
Sample6_Locus_4476_Transcript_9/27_Confidence_0.306_Length_489	-3,314711495	2,10E-06	chlorophyll a b binding protein
Sample1_Locus_1488_Transcript_6/9_Confidence_0.444_Length_1981	-3,403168234	0,001542052	glutamate dehydrogenase
Sample1_Locus_3352_Transcript_18/24_Confidence_0.140_Length_1042	-3,407045438	0,000615931	phytoene desaturase
Sample6_Locus_44_Transcript_137/137_Confidence_0.029_Length_1637	-3,475066266	1,01E-21	putative nitrilase
Sample1_Locus_324_Transcript_15/16_Confidence_0.121_Length_1009	-3,509208914	0,003347531	---NA---
Sample3_Locus_8914_Transcript_1/1_Confidence_0.000_Length_479	-3,593388687	0,000579114	predicted protein
Sample3_Locus_3585_Transcript_6/10_Confidence_0.282_Length_715	-3,710866346	0,001553804	---NA---
SampleTS3_Locus_4205_Transcript_2/2_Confidence_0.333_Length_1332	-3,721370692	2,34E-12	protein serine threonine kinase
Sample6_Locus_701_Transcript_18/22_Confidence_0.224_Length_862	-3,856418098	0,008697366	---NA---
Sample3_Locus_2316_Transcript_26/28_Confidence_0.104_Length_1684	-3,920785087	1,00E-08	ankyrin repeat protein
Sample1_Locus_4210_Transcript_5/6_Confidence_0.250_Length_669	-4,016889188	6,43E-06	rhodanese-like domain-containing protein chloroplastic-like
Sample1_Locus_5959_Transcript_29/31_Confidence_0.207_Length_845	-4,096133885	3,66E-10	---NA---
Sample3_Locus_2912_Transcript_61/73_Confidence_0.104_Length_766	-4,121681238	5,81E-25	peptidase s8
Sample1_Locus_2960_Transcript_23/25_Confidence_0.186_Length_1034	-4,241456213	0,001561769	hypothetical protein TVSG_00003
SampleTS3_Locus_2111_Transcript_1/31_Confidence_0.279_Length_495	-4,399815735	3,31E-28	---NA---
SampleTS2_Locus_1080_Transcript_7/9_Confidence_0.389_Length_2115	-4,431923342	0,008311582	ammonium transporter
SampleTS2_Locus_11027_Transcript_1/1_Confidence_0.000_Length_1814	-4,502312822	0,000266982	lysine histidine transporter-like 5

Sample1_Locus_2253_Transcript_5/16_Confidence_0.447_Length_1433	-4,50593177	0,005776197	proteins containing btb poz and kelch involved in regulatory signal transduction processes
Sample1_Locus_5058_Transcript_19/34_Confidence_0.167_Length_1811	-5,08638029	0,00057094	---NA---
Sample1_Locus_4388_Transcript_6/11_Confidence_0.257_Length_1023	-5,202202604	7,12E-20	hypothetical protein COCSUDRAFT_64655
Sample1_Locus_8578_Transcript_16/27_Confidence_0.481_Length_1212	-5,412816275	0,000491489	---NA---
Sample1_Locus_9106_Transcript_13/13_Confidence_0.400_Length_1902	-5,435143368	3,55E-93	high affinity phosphate probable
Sample3_Locus_2912_Transcript_60/73_Confidence_0.148_Length_730	-5,621702636	9,51E-13	carbohydrate binding family 6
Sample1_Locus_1524_Transcript_22/24_Confidence_0.000_Length_9683	-5,773785844	8,17E-20	polyketide synthase
SampleTS3_Locus_9040_Transcript_2/2_Confidence_0.500_Length_554	-6,448448404	1,67E-05	---NA---

Table S6. List of nitrilase and nitrilase-like sequences retrieved from public repositories utilised to generate the ML tree.

Species	Enzyme note	Source	Accession Number
Ahnfeltiopsis flabelliformis	-	realDB	-
Arabidopsis thaliana	NIT3	Howden et al. (2009)	-
Arabidopsis thaliana	NIT1	Howden et al. (2009)	-
Arabidopsis thaliana	NIT4	Howden et al. (2009)	-
Arabidopsis thaliana	NIT2	Howden et al. (2009)	-
Arabidopsis thaliana	nitrilase/cyanide hydratase	GenBank	NP_567340
Arachis duranensis	nitrilase-like protein 2		XP_015942307
Asparagus officinalis	nitrilase-like protein 2	GenBank	XP_020250716
Auxenochlorella prototechoides	nitrilase-like protein 1	UniProtKB	XP_011396009
Brachypodium distachyon	nitrilase-like protein 2	eggNOG	XP_003575496
Brassica napus	-	Howden et al. (2009)	-
Brassica rapa	NIT-T1	Howden et al. (2009)	-
Brassica rapa	NIT-T2	Howden et al. (2009)	-
Brassica rapa	NIT-T4	Howden et al. (2009)	-
Chlamydomonas eustigma	hypothetical protein	eggNOG	GAX78444
Chlorella variabilis	hypothetical protein	UniProtKB	XP_005845331
Coccomyxa subellipsoidea C-169	carbon-nitrogen hydrolase	GenBank	XP_005648661
Dunaliella tertiolecta CCMP1320	-	MMETSP	-
Eucheuma denticulatum	-	realDB	-
Gracilaria chouae	-	realDB	-
Grateloupia filicina	-	realDB	-
Lupinus angustifolius	NIT4B	Howden et al. (2009)	-
Lupinus angustifolius	NIT4A	Howden et al. (2009)	-
Marchantia polymorpha	hypothetical protein	GenBank	PTQ36733
Medicago truncatula	carbon-nitrogen hydrolase	GenBank	KEH44534
Micromonas commode	carbon-nitrogen hydrolase	GenBank	XP_002503164
Nicotiana tabacum	NIT4A	Howden et al.	-

		(2009)	
Nicotiana tabacum	NIT4B	Howden et al. (2009)	-
Oryza sativa	-	Howden et al. (2009)	-
Panicum hallii	hypothetical protein	GenBank	PAN21099
Populus trichocarpa	nitrilase-like protein 2	UniProtKB	XP_002316330
Raphidocelis subcapitata	nitrilase	GenBank	GBF91685
Rosa chinensis	nitrilase-like protein 2	GenBank	XP_024176796
Selaginella moellendorffii	nitrilase-like protein 2	UniProtKB	XP_002968641
Setaria italica	nitrilase-like protein 2	GenBank	XP_004962940
Sorgum bicolor	nitrilase-like protein 2	eggNOG	XP_002442180
Tetraselmis striata LANL1001	-	MMETSP	-
Tetraselmis suecica	-	Current study	-
Volvox carteri f. *nagariensis*	hypothetical protein	UniProtKB	XP_002956542
Zea mays	NIT2	Howden et al. (2009)	-
Zea mays	NIT1	Howden et al. (2009)	-
Zea mays	nitrilase-like protein 2	eggNOG	NP_001141322
